# Abstracts from the International Science Symposium on HIV and Infectious Diseases (ISSHID 2019): Infectious diseases: Chennai, India. 12-14 October 2019

**DOI:** 10.1186/s12879-020-05038-y

**Published:** 2020-05-20

## ISSHID: E-poster Presentations Abstract-5 Drug resistance profile of Gram negative and related exotic bacterial isolates from an urban hospital in Chennai, Tamil Nadu, India

### Reenaa Muthu^1^, Gayathri Devanandan^1^, Lalitha Kameswari Sankaranarayanan^1^, Parthiban Rudrapathy^2^

#### ^1^Department of Microbiology, Ethiraj College for Women, Chennai, Tamilnadu, India; ^2^Division of Microbiology, Department of Clinical Laboratory Services and Translational Research, Malabar Cancer Centre, Thalassery, Kerala, India

**Background**: Despite the impressive range of therapies available for treating patients in intensive care, bacterial infections continue to be the major challenge worldwide. Impact of gram negative organisms on clinical disease is well known but exotic bacterial pathogens such as Ralstonia sp are lesser known members of this group. Infections related with exotic pathogens are often reported in mechanically ventilated patients particularly associated with those who are immunocompromised. This study aims to analyse the gram negative pathogens and their antibiotic susceptibility profile from various clinical and environmental samples (blood, urine, IV and fomites) from an urban multi-speciality hospital in Chennai, Tamilnadu, India using standard protocols and the data discussed.

**Methods:** Basic microbiological techniques were used to screen the clinical and hospital environmental specimens for gram negative bacteria and their antibiotic susceptibility testing was performed by standard disc diffusion method according to CLSI guidelines. Bacterial isolates which could not be confirmed by basic phenotypic techniques were identified by using automated bacterial identification system (VITEK-2, Biomerieux) and their antibiogram was noted.

**Results**: A total of 40 clinical samples (39 urine and 1blood) and 60 environmental samples were collected. Among these 16/39 urine samples and 17/60 environmental samples were positive for gram negative bacilli. All the gram negative isolates were resistant to nitrofurantoin used to treat UTI.

**Conclusion**: Gram negative organisms and exotic gram negative pathogens are most often associated with hospital acquired UTIs. All Ralstonia sp isolated showed resistant pattern for nitrofurantoin, one of the antibiotic commonly used to treat urinary tract infections.

## ISSHID Abstract-47 A quick method for designing and screening effective chemically modified siRNA against viruses – A perspective to employ RNAi in antiviral research

Showkat Ahmad Dar, Manoj Kumar

Virology Discovery Unit and Bioinformatics Centre, Institute of Microbial Technology, Council of Scientific and Industrial Research, Chandigarh, India

**Background:** Viral infections have a massive negative impact worldwide as evident from recent outbreaks. For example, dengue is emerging as global health problem affecting around 400 million people per year and nearly half of world population at its infection risk. The siRNAs are one of the natural antivirals and some of them are already FDA approved (Patisiran).

**Methods:** We employed virus specific computational algorithms (VIRsiRNApred and SMEpred) for siRNA designing against dengue virus (DENV2) genome. We selected eleven siRNAs (si1 to si11) based on different criteria like varying inhibition efficacy, off targets and different genomic regions (5′-Untranslated region, Capsid, Pre-Membrane, Envelope and 3′-Untranslated region). The siRNAs were further chemically modified with deoxy-nucleotide at the two 3′overhangs. We cloned these genes in PsiCheck-TM2 plasmid and used dual luciferase assay for knockdown efficacy screening of the siRNAs. We tested the knockdown efficacy of siRNAs at three concentrations, their combinations and their toxicity using MTT assay in HeLa cells. Statistical analysis was done by one-way ANOVA with Tuckey post hoc test using R.

**Results:** The designed siRNAs and their combinations performed according to their prediction efficacies. Also, the two siRNAs from earlier studies (for external validation) showed similar silencing efficacies. The siRNAs showed almost no toxicity compared to the scrambled siRNA.

**Conclusion:** We demonstrate a rapid method to design, test and construct a repertoire of chemically modified siRNAs as antivirals without the use of live viruses or biosafety facilities. Our method also showed similar performance as compared to external live dengue virus.

## ISSHID Abstract-63 Herpes Zoster of the Maxillary Division of Tri-germinal Nerve with superadded Streptoccocus viridans infection , in an Immunosuppressed Individual - A Case Report

### Kiran.M^1^, Madhusudhan.B^2^, Pujita.B^2^

#### ^1^Department of Microbiology, Sree Balaji Medical College & Hospital, Bharath University (BIHER), Chennai, Tamil Nadu, India; ^2^Department of Surgery, BRS Hospitals Pvt. Ltd, Chennai, Tamil Nadu, India

**Background:** Herpes Zoster also known as Shingles, is a unilateral, painful vesicular condition, resulting from reactivation of the latent chicken pox (Varicella-Zoster) virus, present dormant in sensory ganglion of cranial nerves or dorsal root ganglion of spinal nerves. Though a self-limiting condition, it may take weeks to resolve especially in immunosuppressed individuals. There is a potential for developing aseptic meningitis, ocular sequelae, post herpetic neuralgia, disseminated zoster and superadded bacterial infections, which pose a great challenge to treating clinicians.

**Case Report:** We report a case of an 80 year old woman, who came with complaints of burning sensation and multiple painful vesicular lesions on the right half of her face and palate associated with fever of one week duration. She had edema of right eye and an ulcer (2x1cm) below her right lower eyelid. Ophthalmic examination was normal. Radiological and blood parameters were normal, except for neutropenia. Blood and wound swab culture from ulcer showed, presence of Streptococcus viridans. Clinical diagnosis of Herpes Zoster involving Maxillary division of Tri-germinal nerve (right side) was made. Written informed consent was obtained from the guardian of the patient for the publication of this case report and accompanying images.

**Conclusion:** Treatment using antiviral agents, antibiotics, corticosteriods, analgesics, anti-inflammatory drugs and corneal protection with ocular lubricants were started and patient showed good improvement. In case of severe Post herpetic Neuralgia, nerve block injections are suggested. Clinicians must be aware of the various complications and treatment modalities associated with the disease, especially in such immunosuppressed and aged patents.

## ISSHID Abstract-76 Study of expression of Brennan Krohn virus in saliva of HIV seropositive and seronegative children

### Kamaleswari P^1^,Ranganathan K^1^, Uma Devi K Rao^1^, Kiran Kumar^2^

#### ^1^Department of Oral Pathology and Microbiology, Ragas Dental College & Hospital, Chennai, India; ^2^ Director, Genes N Life Health Care, Hyderabad, India

**Background:** Salivary gland disease is defined as an enlargement of the salivary gland and/or complaint of xerostomia and histologically characterized by lymphocytic infiltrate of the salivary gland causing acinar atrophy. It has been reported in the literature that in an immunosuppressed setting, Brennan Krohn Virus(BKV) could infect the salivary gland eliciting a CD8T lymphocyte infiltrate and also be expressed in the saliva. In the setting of immunosuppression and immature immune system, Children infected with Human Immunodeficiency Virus are prone to reactivation of BKV if previously infected by the virus leading to disease progression thereby increasing the morbidity.

**Methods:** This study was approved by the IRB of Ragas Dental College and Hospital. Saliva samples were collected from HIV seropositive and seronegative children after obtaining the consent from their parents/guardian. DNA was extracted from the saliva samples and subjected to Real time polymerase chain reaction to study the expression of BKV; the data collected were analyzed using SPSS 20 software.

**Results:** Among the 30 HIV seropositive and seronegative children 27%(8/30) of them had detectable BKV load, 25%(2/8) crossed the threshold level of 200 copies/ml from both groups. We assessed the salivary flow rate and found that there was a decreased salivary flow rate(p=0.05) among the HIV seropositive children who crossed the threshold level of BKV.

**Conclusion:** In this study we detected and quantified BKV in the saliva of both HIV seropositive and seronegative children, indicating that saliva is a potential source of BKV infection. **"**

## ISSHID Abstract-81 Seroprevalance of Hepatitis B in pregnant females - a decade after introduction of Hepatitis B vaccine in Universal Immunization Program of India

### Shilpee Kumar, Rojaleen Das

#### Department of Microbiology, VMMC & Safdarjung Hospital, New Delhi, India

**Background:** In India, Introduction of HBV vaccine was pilot-tested in 2002 and expanded to the entire country in 2011 under the Universal Immunization Program (UIP). It has been a decade since Hepatitis B vaccine has been introduced in UIP. The study was conducted to analyze the change in seroprevalence status of Hepatits B in females of reproductive age group.

**Methods:** This was a retrospective observational study. The cohort enrolled for the study were pregnant females who attended the antenatal clinic of VMMC & Safdarjung Hospital in 2019. The blood sample was collected as a part of routine screening for HBV, HIV and syphilis. The samples were tested for HBsAg by qualitative indirect ELISA.

**Results:** A total of 5513 pregnant females were enrolled in the study and 89 were seropositive for HBsAg. The seroprevalence was found to be 1.6% and highest in the age group of 30-35 year (2.7%). Studies before 2011 have shown seroprevalence of 2.3–6.3%.

**Conclusion:** The seroprevalence of HBsAg has decreased over the years. However as India is the second most populated country, the seroprevalence of 1.6% amounts to nearly 4 million women in reproductive age infected with HBV and putting 4 Lakhs babies at risk of acquiring HBV by vertical transmission yearly. Vaccine delivery assessment is required to identify the gaps that may help in strengthening the national viral hepatitis control program of India.

## ISSHID Abstract-101 Marine brown algal compounds inhibits HSV-2, an invitro analysis

### Manikannan Mathaiyan, Lavanya Babu, Krupakar Parthasarathy

#### Center for Drug Discovery and Development, Col.Dr.Jeppiaar Research Park, Sathyabama Institute of Science and Technology, Chennai, India

**Background:** Herpes Simplex Virus-2 (HSV-2) belongs to the family Herpesviridae, a causative agent of genital herpes. Wild-type HSV-2 may cause severe infections in neonates and HSV-2-infected individuals are placed at approximately three fold higher risk for acquiring human immunodeficiency virus. Vaccines against HSV-2 remain a challenging approach and also drug resistance is increasing. In this context, it is important to identify newer HSV inhibitors. In this study we aimed to identify the inhibitors from marine brown algae, Sargassum species.

**Methods:***Sargassum swartzii*, a Marine brown algae were collected from south coastal of Tamilnadu. Crude extracts were prepared by standard extractions procedure and phytochemicals present in the crude extracts were separated. Vero cells 2.5x105/well were infected with stranded HSV-2 strain and cytopathic effect (CPE) was calculated. 50- 200μg of phenolic compounds and flavonoids treated with HSV-2 infected vero cells for up to 72 hours. Percentage of inhibition was calculated based on number of plaques reduction. Acyclovir used as a drug positive control.

**Results:** Number of plaques was calculated as 1.7x105/ml PFU/ml. Among the different conc. of test compounds tested 200μg/ml of flavonoids have shown less no of plaques. At tested 200μg/ml virus yield decreased by 90 fold indicates that plaques reduction was due to virus inhibition mediated by flavonoids antiviral property. This suppression is not due to cytotoxic effects of compounds, it was confirmed by MTT assay. Up to 500μg/ml of flavonoids were non toxic to vero cells.

**Conclusion:** Flavonoids are the principle compounds potentially inhibit HSV-2 in invitro condition.

## ISSHID Abstract-116 Early diagnosis and drug resistance detection of Pulmonary Tuberculosis by TRUENAT and LPA in a tertiary care hospital

### D.Shireesha, A.Surekha, A.Renuka Devi, B.Nagajyothi, J.Vijayalakshmi

#### Dept of Microbiology, Kurnool, 518002, A.P.

**Background:** Tuberculosis is one of the most important diseases in india. Early diagnosis and detection of drug resistance is the only way in controlling the spread of tuberculosis. Newer diagnostic modalities have been extremely useful in achieving the same. so there is a need to know the prevalence of tuberculosis in our geographical area(kurnool A.P.) and to stratify the patients based on the variables like age ,sex, and drug resistance with help of TRUENAT and LPA .

**Methods:** This is a prospective study done during the study period from march 2019 to June 2019.All the samples obtained during the study period were tested according to the RNTCP guidelines by using TRUENAT and line probe assay.

**Results:** A total of 1221 sputum samples were processed during the study period.Out of 1221 samples 244 (19.9%)of the samples were positive for tuberculosis. Positivity rate was higher in males(69.2%) compared to females(30.7%).Rifampicin resistance was detected in 73(29.9%) of the cases by TRUENAT.Line probe assay has detected isoniazid resistance in 30(12.29%) of the positive samples out of which H monoresistance was observed in 18(7.3%) samples. Fluoroquinolone resistance and injectable aminoglycoside resistance was detected in 14 cases(5.7%).Bedaquline was started for 8 cases of DRTB.

**Conclusion:** TRUENAT and LPA assays have been extremely useful in the diagnosis and identification of drug resistance of suspected tuberculosis cases.

## ISSHID Abstract-124 Significant mutations in the genome of dengue virus serotype-1support its continuous circulation from 2014 to 2019 in Hyderabad, India

### Musturi Venkataramana

#### Department of Biotechnology & Bioinformatics, School of Life Sciences, University of Hyderabad, Gachibowli, Hyderabad, Telangana, India

**Background:** India is hyper-endemic for dengue virus infections circulating all four serotypes. But there are reports indicating only one or two of the serotypes dominates for certain period of time in few regions and causes significant morbidity and mortality. The reason for this uniqueness is not known. Hence this study was undertaken in order to understand the dynamics of dengue virus circulation in Hyderabad, India from 2014 to 2019.

**Methods:** Viral RNA was isolated from the dengue virus infected serum samples using the Qiagen viral isolation kit. cDNA was synthesized using the reverse primer of the pre-Membrane region which was further amplified (511bp) by using the forward primer. Serotyping was carried by using the above template with serotype specific primers as described in earlier reports.

**Results:** The data suggested the circulation of all four serotypes with equal proportion during the year 2014. 60% of the infections were co-infections with more than one serotype and caused severe dengue ie either dengue hemorrhagic (DHF) or dengue shock syndrome. But over the time, dengue virus serotype-1 emerged as single serotype circulating alone in 2019 in Hyderabad. The nucleotide sequence analysis indicated the significant mutations throughout the genome which might played crucial role in better adaptation and hence in circulating for the past five years.

**Conclusion:** The adaptive mutations acquired by the dengue virus serotype-1, might played crucial role in better adaptation and hence in circulating for the past five years continuously circulating in Hyderabad, India.

## ISSHID Abstract-135 CD4 COUNT- A measure of drug resistance in HIV patients who are on 1st line ART in a tertiary care hospital

### A.Praveen, A.Surekha, A.Renuka Devi, B.Shanthi Reddy, R.Hymavathi

#### Kurnool, A.P,518002

**Background:** Measuring CD4 (T- Cell) count in HIV patients gives us a measure of the response to ART and also provides an assessment of disease progress. Virus mutations were noticed in patients who failed ART treatment. Other factors contributing to ART resistance were poor medication compliance, genetic differences in drug metabolism, improper nutrition, and poor socioeconomic status.

**Methods:** A retrospective observational study was conducted to examine the 1st line ART resistance using CD4 count among ART users in government general hospital, Kurnool starting from the year 2014 to 2019. All the ART users were adults aged between 20-60 years. Patients who came for ART treatment, CD4 count was monitored every 6 months for a period of 5 and half years

**Results:** A descriptive analysis of the data of 7,560 patients who were on ART was conducted. Out of 7,560 patients only 150 patients had shown resistance to 1st line ART i,e CD4 count <100cells/mm3. Among the 150 patients, 95 patients had a CD4 count <100cells/mm3 after 60 months on ART, 32 patients had a CD4 count <100 cells/mm3 after 24 months on ART and remaining 23 patients had a CD4 <100cells/mm3 after 12months on ART indicating resistance to 1st line ART. It was observed males showed predominance over females in ART resistance.

**Conclusion:** This study shows that measuring CD4 count can be useful in the early detection of 1st line ART resistance .This could also help us to identify and monitor poor treatment adherence in HIV patients.

## ISSHID Abstract-136 Re-emergence of diphtheria in Northwest-zone of Tamilnadu

### Mythili Pradeep, S Rajesh, M Kavitha, T Sundararajan, R Vidhyarani, S Deepa

#### Department of Microbiology, GMKMC,Salem,Tamilnadu

**Background:** Diphtheria is one of the most threatful infectious disease especially in children with high fatality rate. Diphtheria has re-emerging probably due to pockets of unimmunisation and non-adherence to booster doses. From 2011-2015, India had the largest number of reported cases (18,350) followed by Indonesia ( 3203 ).

**Methods:** This is a descriptive study, conducted in GMKMCH Salem.21 clinically suspected diphtheria cases had been admitted in our tertiary care centre during the period of oct-2018 to Aug-2019. Age group of the patients under 12 years.Specimens ( throat swabs ) were collected from diphtheria suspected cases and processed for staining and culture followed by establishment of pathogenicity with toxigenicity testing in reference lab.

**Results:** Total number of cases studied- 21.Among 21 cases,5(3 males,2 females with mean age of 5 years who had missed DPT booster doses) were positive for direct gram stain and Albert stain.Preliminary report was given to the clinician immediately following which diphtheria antitoxin was administered to the patients. Culture results were positive.Serum samples and PTA isolates were sent to the reference lab (CMC Vellore) for toxigenicity testing and molecular characterization. All the 4 samples were toxic stains and TOX A gene positive (RT-PCR).Out of 5, 4 patients died.

**Conclusion:** In the era of vaccination, diphtheria is re-emerging mainly because of lack of booster dose vaccination coverage. Booster doses are needed to ensure continuous protection.Ring immunisation to be done to prevent outbreak. Public should be instructed to vaccinate their children at appropriate age with correct doses.

## ISSHID Abstract-143 Leprosy- is it really eliminated in India?

### Cordelia Babitha, Maitreyi Parachuri, Murugan Sundaram

#### Department of Dermatology, Sri Ramachandra Institute of Higher Education and Research, Porur, Chennai, India

**Background:** Leprosy is one of the oldest disease known to man, continues to be a major public health problem in India. Multidrug therapy has helped to achieve the elimination status of less than 1 case per 10,000 in December 2005. However there is a recent rise in the number of new cases being recorded. The objective of this study is to present the newly diagnosed leprosy cases between January 2019 to July 2019 in our hospital.

**Methods:** In this case series 22 patients with clinical features suggestive of leprosy presented to the dermatology OPD between January 2019 to July 2019. All the patients were subjected to physical examination,slit skin smear and biopsy.

**Results:** Among the 22 cases, 6 were female and 16 male and were categorized into Ridley-Jopling classification with 15 patients in borderline, 5 in lepromatous and 2 in tuberculoid spectrum. Type 1 reaction was seen in 4 patients and type 2 in 2 patients. Patients were started on WHO-MDT , those on reaction were managed with glucocorticoids. Screening and counseling of all household contacts were done.

**Conclusion:** India accounts for 60 % of new cases being reported despite the prevalence being reduced from 5 million cases in 1998 to less than 2,00,000 in 2016. Long incubation period, lack of awareness, fear and stigma accounts for majority of hidden cases. Important measures like house to house surveys, screening of children and elderly, chemoprophylaxis to contacts and research of transmission and treatment will help to truly eliminate leprosy one day.

## ISSHID Abstract-145 Are the Antenatal women susceptible to Rubella and measles? -A Seroprevalence study

### Sherly Antony^1^, George Varghese^1^, Bilsa P M^2^, Reshma Mary Joseph^3^, Vindhuja Udayan^2^, Reeshma R Nair^1^, Reshmi K Pillai^1^, Kavya C Ravi^1^, Philip Mathew^4^, Mercy John Idikula^1^

#### ^1^Department of Microbiology, Pushpagiri Institute of Medical Sciences and Research Centre, Thiruvalla, Kerala, India; ^2^Department of Medical Microbiology, Union Christian College, Aluva, Kerala, India; ^3^Department of Medical Microbiology, School of Health Sciences, University of Calicut, Malappuram, Kerala, India; ^4^Department of Community Medicine, Pushpagiri Institute of Medical Sciences and Research Centre, Thiruvalla, Kerala, India

**Background:** Globally, MMR vaccine coverage has reduced the load of measles, mumps and rubella. However a recent surge in measles cases is observed. USA reporting its maximum cases ever since 1992, threatening its elimination status. In India, IDSP noted high case burden in the last three years which may be just the tip of the iceberg. As India, aims to eliminate measles and rubella by 2020, the susceptibility of the antenatal women in whom the infection can have adverse consequences was looked into.

**Methods:** IgG antibodies to rubella and measles were assayed by ELISA (Euroimmun, Germany) on 75 sera received from Antenatal clinic. The seronegativity (95% confidence interval), cross-tabulated with age status using tests of proportions, Chi Square Test and Odds Ratio for association between age and seronegativity were analysed.

**Results:** For measles, 59 participants were seropositive and 2 borderline while for rubella 60 were positive and 1 borderline. Seronegativity with 95% confidence interval for IgG to measles and rubella was 18.7 % (CI=9.7 to 27.7%). Most seronegatives were aged between 19-24 years. However, the association between age and seronegativity were not statistically significant. (p= 0.061 measles and 0.98 rubella).

**Conclusion:** Kerala state despite its good health indices has 18.7% seronegativity to measles and rubella which could be clinically significant. It represents 7,453 mothers of total antenatal population in the state (37,264). Hence to achieve WHO’s elimination target of 2020, it is of high priority that women in child bearing age are added to the existing MR vaccine campaign

## ISSHID Abstract-155 Candida Biofilms: A study of biofilm formation among various Candida species at a tertiary care hospital

### Manohar. M. Reddy, A. Renuka Devi, A. Surekha, B. Shanthi Reddy, R. Hymavathi

#### Department of Microbiology, Kurnool Medical College, Kurnool AP India

**Background:** Biofilms are formed when microorganisms attach irreversibly to inert and living surfaces. Since the surfaces are non shedding, these surfaces provide an ideal place for colonization by biofilm forming microorganism.

**Methods:** Samples of urine, pus, blood, cerebrospinal fluid, body fluids, oral and ear swabs from patients were processed in the microbiology laboratory. Different candida species grown on culture media were identified, isolated and inoculated into microtitre plate wells. Biofilm production in microtitre wells was detected by measuring their optical density with an ELISA reader.

**Results:** A total of 64 various candida species were isolated from all samples. Among the various candida species isolated, 24 were *Candida albicans* and 17 formed biofilms, 21 were Candida tropicalis and 14 formed biofilms, 13 were *Candida krusei* and 11 formed biofilms, 4 were *Candida parapsilosis* and 3 formed biofilms, 2 were *Candida glabrata* and none formed biofilms.

**Conclusion:** A thorough knowledge of biofilm formation of candida species will help us in an early diagnosis and treatment of candida infection, preventing its further spread and improving patient outcome.

## ISSHID Abstract-158 Structural analysis of NS2B/NS3 proteases of flaviriridae and novel inhibitor design

### Ramakrishnan C^1^, Velmurugan D^2^, Gromiha MM^1,3^

#### ^1^Department of Biotechnology, Bhupat and Jyoti Mehta School of Biosciences, Indian Institute of Technology Madras, Chennai - 600 036, INDIA; ^2^Centre of Advanced Study in Crystallography and Biophysics, University of Madras, Chennai, India; ^3^Advanced Computational Drug Discovery Unit, Tokyo Tech World Research Hub Initiative (WRHI), Institute of Innovative Research, Tokyo Institute of Technology, 4259 Nagatsuta-cho, Midori-ku, Yokohama, JAPAN

**Background:** Dengue, West Nile and Zika viruses belong to flaviviridae family and cause life threatening fevers. These three viruses express the polyprotein encoded by 5'-C-prM-E-NS1-NS2A-NS2B-NS3-NS4A-NS4B-NS5-3' gene for their life cycle in the host cells in common. This polyprotein is cleaved into the active protein components with the aid of protease activity of NS2B/NS3pro complex. Thus, the NS2B/NS3 is considered as a potent target for designing inhibitors against these viruses.

**Methods:** We have performed a structural analysis of NS2B/NS3pro complex using molecular modeling and dynamics simulations and explained the structure-function relationship of this complex based on the similarity and differences in the structural features. Further, we have employed screening of protease inhibitor chemical space using high-throughput virtual screening.

**Results:** We have obtained the structures of substrate peptide (GLKR’GGAK) bound NS2B/NS3 protease of DENV, WNV and Zika virusus. These complex structures represent the model for the protease activity in cleaving the substrate peptide and it is robust for designing high affinity and low risk compounds with specific as well as non-specific protease inhibitors. We also present some of the compounds selected from the in silico studies as promising inhibitors against DENV and WNV proteases.

**Conclusion:** Our results provide a strong platform for structure/ensemble-based screening of large chemical libraries and also to prepare NS2B/NS3pro focussed compound libraries. Further, NS2B/NS3pro specific/non-specific inhibitors can also be designed based on our pipeline. Together, our computational method helps designing of potent antiviral drugs, efficiently and rapidly.

## ISSHID Abstract-164 Seroprevalence of Hepatitis C Virus infection in haemodialysis patients in a tertiary care hospital

### G. Mounika, A. Renuka Devi, A. Surekha, B. Nagajyothi, J.Vijaylakshmi

#### Department of Microbiology, Kurnool Medical College, Kurnool AP India

**Background:** Hepatitis C virus is an RNA virus and a known cause of infection transmitted in patients undergoing haemodialysis. The source of infection stems from improper washing and disinfecting equipment used in haemodialysis. Infection can also occur due to improper hygiene practises while handling the dialysis equipment and patient. Transmission through contaminated blood while treating anaemic patients is another source of HCV infection. HCV infection has been implicated in the pathogenesis of glomerulonephritis leading to worsening of the already compromised kidney. Therefore prevention of HCV transmission and early diagnosis and treatment of HCV is very essential.

**Methods:** This is a prospective observational study conducted for 3 months done to identify HCV patients on hemodialysis in the geographical location ( Kurnool, AP). Blood samples were collected and screened for HCV antibodies by ELISA for all patients who came for hemodialysis. Ethical clearance was obtained and prior consent from patients was taken before testing.

**Results:** Among 139 patients who were on hemodialysis 104 patients were males and 35 patients were females with a mean age of 51±15 years. 4 patients were identified as having HCV infection. All 4 patients were old cases of HCV infection. No new cases were found in the time period of the study

**Conclusion:** This study looks at the disease burden and rate of transmission of HCV in hemodialysis patients. Use of proper protocols and infection control practices prevents the incidence of Hepatitis-C infection in haemodialysis patients.

## ISSHID Abstract-168 Role of antimicrobial peptides against the biofilm formation in MRSA strains

### Thenmozhi Ramalingam^1^, Beema Shafreen Rajamohamed^2^, Thajuddin Nooruddin^1^

#### ^1^Department of Microbiology, Bharathidasan University, Tiruchirappalli, Tamilnadu, India; ^2^Department of Biotechnology, Alagappa University, Karaikudi, Tamilnadu, India

**Background:** Biofilm formation imposes a major threat in clinical manifestations. Still Methicillin Resistant *Staphylococcus aureus* (MRSA) is a greater challenge to the physicians in controlling its emergence. Apart from controlling MRSA biofilms by antibiotics, we have approached with a novel antimicrobial peptide (AMP), Chensinin-1 (SPDC-1) against its biofilm formation.

**Methods:** About fifty patients and their nasal swabs has been taken for the study. The isolated MRSA isolates were subjected to the study of biofilm forming potential by crystal violet assay. These biofilm formers were treated with SPDC-1.

**Results:** Out of fifty patients screened, eighteen turned to be positive for MRSA. From the 18 MRSA strains 14 formed biofilms at varying degrees. N7 and N23 two best biofilm formers have been chosen for further studies on par with MRSA ATCC 33591. The biofilms appear to be thick and very dense in their microcolony formation, which was supported by Light microscopy, Scanning Electron microscopy and Confocal microscopy. The biofilm formers were treated with SPDC-1at various concentrations from 5 to 20 μM. At least concentration of 5 μM the SPDC-1 showed efficient disintegration of biofilm MRSA strains. Further assays also supported the efficiency of AMP against biofilm forming MRSA strains.

**Conclusion:** Hence this study showed the efficacy of antimicrobial peptide against biofilm forming MRSA stains. This AMP has no side effects and emergence of resistance to AMPs has not been reported so far.

## ISSHID Abstract-188 Evaluation of diagnostic efficiency of nested PCR in the diagnosis of Herpes simplex virus-2 (HSV-2) infection

### Deepa V, Rayvathy B

#### Department of Microbiology, Dr. ALM Post Graduate Institute of Basic Medical Sciences, University of Madras, Taramani, Chennai- 600 113, India

**Background:** Herpes simplex virus 2, causes genital ulcer disease (GUD) and the patients present with genital pain and sores. The diagnosis of HSV 2 infection is primarily clinical as Tzanck smear lacks sensitivity and specificity. Serology helps in screening but unreliable for diagnosis and culture is difficult to perform in all laboratories. So, molecular methods are much promising for efficient diagnosis. Hence, this study proposes to use a nested PCR assay for the diagnosis of HSV-2 infection from genital swabs of patients with GUD and compares its diagnostic efficiency with that of real-time PCR.

**Methods:** A total of 40 genital ulcer swab samples collected from GUD patients attending a tertiary Sexually transmitted infections (STI) clinic were included in this study. DNA was extracted using the Nucleospin virus kit and nested PCR assay was performed using specific primer sets targeting POL gene (163-bp) of HSV-2. The same samples were analyzed using real time-PCR with specific probe and primers targeting the glycoprotein G gene of HSV-2.

**Results:** 23/40 samples were positive by nested PCR and 27/40 samples were positive by real-time PCR. It was found that nested PCR had a sensitivity and specificity of 55.5% and 46.1% respectively. The positive and negative predictive values were 68.2% and 33.3% respectively.

**Conclusion:** Thus, the evaluation of diagnostic efficiency of nested PCR in HSV -2 diagnosis shows that it is not a good diagnostic test as it has poor sensitivity and specificity.**"**

## ISSHID Abstract-229 Detection of Non typeable *Haemophilus influenza* by P6 (OMP) gene from Respiratory samples

### D.Danis Vijay^1^ S.Jayanthi^1^, N.Meenakshi, S.H.ShifaMeharaj^1^, A.Sujhithra^3^, J.Perumal^1^

#### ^1^Department of Microbiology, Chettinad Hospital and Research Institute , Chettinad Academy of Research & Education, Kelambakkam, Tamilnadu, India; ^2^Department of Respiratory Medicine, Chettinad Hospital and Research Institute, Chettinad Academy of Research & Education, Kelambakkam, Tamilnadu, India; ^3^Department of Allied Health Science, Chettinad Academy of Research & Education, Kelambakkam, Tamilnadu, India

**Background:** Nasopharyngeal NTHI colonization leads to the risk of respiratory tract infections. Although NTHI related diseases can be successfully treated with the commonly used β lactam antibiotics such as ampicillin or amoxicillin, resistance among the bacteria is rising increasingly through different mechanisms such as β lactamase or through modified penicillin - binding protein with low affinity for β lactams. Aim:To identify the P6 gene from the respiratory samples and to differentiate the Typeable and Non-typeable Haemophilus influenza.

**Methods:** The prospective bacteriological survey of 75 sputum and nasopharyngeal swab were screened for P6 gene suspected with history of respiratory tract infection. Simultaneous phenotypic and genotypic confirmation were done for typeable and Nontypeable Haemophilus influenza by 16SrRNA and P6 gene and study was done for period of six months from January 2019 to June 2019.

**Results:** Nearly 75 respiratory samples (63 were sputum sample and 12 Nasopharyngeal swab) were screened for presence of H.influenza. Phenotypic confirmation was done following isolation of the organisms and 15 showed cultures positive. Among the 75 samples, 10 nasopharyngeal swab and 5 sputum samples were positive for P6 gene.

**Conclusion:** The outer membrane protein P6 gene is considered as the good genetic marker for detection of the *Haemophilus influenza* at the earliest. Molecular detection tests could complement culture-based tests by strengthening their surveillance.

## ISSHID Abstract-242 Pluronic based Niosomal formulation for topical delivery of anti-infective drug secnidazole

### Udaya Sakthi M, Ramyadevi D, Vedha Hari BN

#### Department of Pharmaceutical Technology, School of Chemical & Biotechnology, SASTRA Deemed University, Thanjavur, Tamil Nadu, India

**Background:** Topical anti-infective therapy by conventional dosage forms has limitations such as short residence time, degradation of the drug at mucosal surfaces, etc. To overcome these limitations and enhance the therapeutic efficacy, vesicular systems were considered as they permeate effectively through skin dermis. With this background, the objective of our study was to prepare niosomes of Secnidazole with Pluronic F127 owing to its non-ionic surfactant property and explore its use in topical delivery of anti-infective agents.

**Methods:** Secnidazole niosomes were prepared by the Modified ether injection method and the Thin-film hydration method in the presence and absence of cholesterol. The physicochemical properties of the developed noisomes were evaluated through particle size analysis, thermogravimetric analysis combined with differential scanning calorimetric analysis, X-ray diffraction studies, drug content, skin permeation, Transmission electron microscope, confocal laser scanning microscopy and in-vitro drug release studies.

**Results:** The formulations devoid of cholesterol showed required niosomes formation and were considered for further evaluations. The vesicle size of optimized formulation was found to be 396 nm with the PDI value of 0.5. The interaction studies revealed high drug stability. The niosomes demonstrated the faster release of Secnidazole due to the presence of Pluronic surfactant. The Confocal Laser microscopic study confirmed the spherical nature and improved skin permeability of the Secnidazole niosomes.

**Conclusion:** Secnidazole niosomes prepared by the thin-film hydration method were stable for 90 days without any particle aggregation or sedimentation. Hence, Pluronic F127 based Secnidazole niosomes could be an efficient carrier for topical anti-infective therapy.

## ISSHID Abstract-251 Altered systemic levels of acute phase and antimicrobial proteins and post treatment modulation in tuberculous lymphadenitis

### Gokul Raj Kathamuthu^1,2^, Kadar Moideen^1^, Rathinam Sridhar^3^, Dhanaraj Baskaran^2^, Subash Babu^1,4^

#### ^1^National Institutes of Health-NIRT-International Center for Excellence in Research, Chennai, India; ^2^National Institute for Research in Tuberculosis (NIRT), Chennai, India; ^3^Government Stanley Medical Hospital, Chennai, India; ^4^Laboratory of Parasitic Diseases, National Institute of Allergy and Infectious Diseases, National Institutes of Health, Bethesda, Maryland, USA

**Background:** Pulmonary tuberculosis is characterized by elevated levels of acute phase (APPs) and antimicrobial proteins (AMPs). However, data on the association of APPs and AMPs with tuberculous lymphadenitis (TBL) is scarce.

**Methods:** We have examined the systemic levels of APPs and AMPs in TBL (n=44) and latent tuberculosis (LTB, n=44) individuals at baseline and in TBL before and after the completion of anti-tuberculosis treatment (ATT, n=44). We measured the plasma levels of alpha-2-macroglobulin (⍺-2MG), serum amyloid A (SAA), C-reactive protein (CRP), haptoglobin (Hp), human beta defensin 2 (HBD-2), granulysin, cathelicidin (LL-37) and human neutrophil peptide1-3 (HNP1-3) by ELISA. We also examined the association of these proteins with TBL lymph node (LN) size and multiple (M) versus single (S) LN involvement. Finally, we have also examined the pre and post-treatment modulation of APP and AMPs in TBL. The statistically significant differences were analysed using GraphPad PRISM 8.

**Results:** TBL individuals exhibit increased plasma levels of APPs (⍺-2MG, SAA1, CRP, elevated (HBD-2) and diminished (granulysin, HNP1-3) AMPs when compared to LTB individuals. In contrast, none of these proteins was significantly associated with LN size and multiple versus single LN involvement indicating a lack of association with disease severity. Following ATT, the systemic levels of ⍺-2MG, CRP, Hp, HBD-2 were significantly diminished and granulysin and HNP1-3 were significantly elevated when compared to pre-treatment levels.

**Conclusion:** Thus, altered levels of APPs and AMPs at baseline corroborates the presence of systemic inflammation and enhanced immune activation and their reversal after ATT indicates modulation after treatment in TBL disease.

## ISSHID Abstract-283 Identification of ligand receptor interaction across blood brain barrier in dengue pathogenesis

### Durga Bethala, Shashikant Vaidya

#### Haffkine Institute for Training, Research and Testing, Mumbai

**Background:** Clinical evidences suggests that blood brain barrier (BBB) may be compromised during DENV infection; however, it is not clear whether the damage is due to the infection or to the inflammatory response generated by the BBB cells. Other neurotropic flaviviruses such as Japanese encephalitis virus (JEV) are also known for neurological damage, it uses the bloodstream to enter nerve tissue where it infects cells of the neurovascular unit. While the rabies virus chooses the retro-grade axonal route to infect the neuronal cells. Both JEV and RABV affect the cells from neurovascular unit in different ways resulting into BBB damage. Our aim is to identify receptors binding to DENV envelope protein (DEP), compared with other neurotropic viruses like JEV and RABV using in silico model. This information will allow us to understand impact of dengue virus on our complex nervous system.

**Methods:** DEP sequence was submitted in SWISS-MODEL software the energy minimized stable structure was developed. This structure was further subjected to protein-protein interaction with brain endothelial membrane proteins using PATCHDOCK and FIREDOCK softwares. The structure with lowest binding energy score was selected to identify important amino acids at the interface binding region.

**Results:** On the basis of lowest energy score plasma lemma vesicle associated protein, annexin A6 and vacuolar protein sorting associated protein-13C has shown higher interaction to dengue envelope protein as compared to other viruses.

**Conclusion:** We can compare the in-silico data of dengue envelope protein interaction with brain endothelial membrane receptor with the in-vitro study

## ISSHID Abstract-285 Pulmonary tuberculosis: a rare occurrence presenting as diffuse alveolar hemorrhage

### Raghav raj J, Anantha Subramanian

#### Kauvery Hospital, Chennai, India

**Background:** pulmonary tuberculosis is commonly encountered in daily practice with varied presentation. Diffuse alveolar hemorrhage is an exceedingly rare manifestation of pulmonary tuberculosis with few case reports.

**Case report:** A 38 year old female presented with hemoptysis, progressive dyspnea for seven days. On examination her respiratory rate was 32/min with room air saturation was 88%. On systemic examination she had crepitations. Her chest xray showed diffuse nonhomogeneous infiltrates with reticular shadows. Her baseline blood investigations were normal with a exception of hemogram A computed tomography thorax was done on 1st day of admission, which showed bilateral diffuse ground glass opacity. In view of hemoptysis, radiological and laboratory (anemia) picture, a provisional diagnosis of diffuse alveolar hemorrhage was made and she was started on intravenous methyl prednisone. work up for autoimmune/coagulation disorders was done with ANA, ANCA ,ANTI-GBM, APLA antibodies, PT/APTT being non contributory. She was taken up for bronchoscopy following clinical stabilization and Bronchoalveolar lavage was taken, which showed Acid Fast Bacilli, Genexpert Positive and cytology revealing hemosiderin laden macrophages. She was initiated on anti tuberculosis therapy, was discharged with the same and tapering dose of steroids. On follow up she had no episodes of hemoptysis and radiological clearance. Consent was obtained from the patient for the publication of her data

Discussion: DAH is a medical emergency, It often occurs with vasculitis, autoimmune disorders, drugs. Pulmonary tuberculosis has been associated with it less often. The present case highlights the possibility of TB being a differential diagnosis and the improvement following therapy.

## ISSHID Abstract-309 Comparison of Multi-drug resistant Tuberculosis prevalence in HIV and non-HIV infected patients in a tertiary care center

### R.Monica, PAT.Jagatheeswary

#### Department of Microbiology, Saveetha Medical College, Thandalam, Chennai, India

**Background:** Multidrug-resistant tuberculosis is one of the most important HIV-related opportunistic infections. The study aims to assess the prevalence of multi-drug resistant Mycobacterium species from suspected pulmonary tuberculosis (PTB) cases amongst newly diagnosed HIV positive patients and compare it with non-HIV patients attending a tertiary care center.

**Methods:** A retrospective study was conducted from January 2018 to July 2019. Sputum samples were collected from patients who were suspected to have PTB and 261 of them were found to sputum positive for acid-fast bacilli (AFB) by Fluorochrome and Ziehl- Neelson staining. The sputum samples of these patients were subjected to the detection of Mycobacterium spp. by Cartridge-based nucleic acid amplification test(CBNAAT) and also checked for rifampicin and isoniazid(RIF) resistance. Serum samples were obtained from these patients and tested for antibodies to HIV by ELISA and CLIA methods.

**Results:** Among the 3265 patients screened for PTB, 261(7.9%) were found to be positive for AFB. All of these 261(7.99%) patients were screened by CBNAAT and were found to be positive for Mycobacterium tuberculosis and 5(4.21%) were found to have genes coding to RIF resistance. Among this 261, 3(2.68%) patients were found to be reactive for antibodies to HIV and also found to have gene coding for RIF resistance.

**Conclusion:** In developing countries with a high prevalence of tuberculosis in the general population, the possibility of incidental tuberculosis in patients with HIV should always be considered. Universal Drug Susceptibility Testing by molecular methods should be followed especially in HIV co-infected TB patients in tertiary care centers.**"**

## ISSHID Abstract-312 Novel disease target identification in *Mycobacterium tuberculosis* H37Rv by insilico subtractive genomics approach

### Srinivasan.S^1^, Sasikala.S^1^, Saradhai.P^1^,Mumtaj.P^2^

#### ^1^PG & Research Department of Microbiology and Biotechnology, Presidency College(Aut), Chennai, Tamilnadu, india; ^2^Bioinformatics Infrastructure Facility centre of DBT, Presidency College(Aut), Chennai, Tamilnadu, India

**Background:** Multi-drug resistant (MDR) and extensively-drug resistant (XDR) strains has emerged all over the world including India. *Mycobacterium tuberculosis* causes Tuberculosis a highly infectious disease and has developed resistance against the first line and second line drugs

**Methods:** Complete proteome sequences of Mycobacterium tuberculosis H37Rv, retrieved from NCBI, were subjected to BLAST-P, against DEG and in NCBI server for the screening of essential genes and proteins, respectively. Non homologous sequences were selected.

Non homologue essential proteins sub cellular localization was identified by P SORT server and metabolic pathway analysis was done by KEGG Automatic Annotation Server (KAAS). Comparative analysis of the metabolic pathways of the host and pathogen was performed by using KEGG pathway database. Modeling of the target proteins were performed using Swiss Modeller and were validated using PROCHECK. Non bonded interactions between different atoms types were measured by ERRAT program. RMSD and RMSF were calculated for modelled structures. Functional active-sites were identified through CASTp.

**Results:** By subtractive genomics approach, 947 non homologous genes were identified. About 250 membrane localized proteins were identified. Three dimensional model of drug targets were generated through identified templates along with fold fitting. Fold recognition was done through GenThreader and LOMETS server. Generated 3D model of target proteins were checked by Ramachandran plot through PROCHECK programme.

**Conclusion:** Use of the DEG database is more useful method for identification of essential genes. The present study has thus led to the identification of membrane proteins that can be targeted for effective drug designing.

## ISSHID Abstract-315 Dengue In Pregnancy: A Clinical Dilemma In Diagnosis

### Nidhi Sharma, Archana Prakash, Shanthi Ethiraj, Hussaina Sheikh

#### Saveetha Institute of Medical and Technical Sciences, Chennai, 602105, India

**Background:** The vector of dengue fever (caused by 4 closely related serotypes of Flavivirus), A. aegypti is endemic in South East Asia. Dengue in pregnancy has a mortality of 20%. The plasma leak from intra vascular to extravascular compartment results in shock that progress as uncompensated shock (Dengue Shock syndrome). Second cause of mortality is hemorrhagic manifestations (Dengue Hemorrhagic fever).

**Methods:** Eleven pregnant women with dengue(confirmed with Dengue ELIZA IgG and IgM and NS1 Ag) were treated by intravenous normal saline (loading and maintenance). Intravenous dextrose, intramuscular injections, steroids, NSAIDS and Cariprill were avoided. Paracetamol infusions and platelet transfusions were given. Daily blood counts, input output charting, amniotic fluid index and nonstress test was done. Induction of labor was avoided.

**Results:** The mean duration of febrile phase, critical phase and recovery phase was 4.54+1.16, 4.64+1.49 and 7.64+0.88 days (mean total duration of illness was 16.91+2.31). There was a significant fall in platelet count on day 4(t= 5.41,SD= 0.517,p<. 0001) There was no significant fall in leukocytes (t= 0.220,SD= 1.35,p=0.83). There was no significant rise in hematocrit (t=-0.904,SD=2.12,p=0.38). The mean pulse pressure was 34+8.1mm Hg. Tourniquet sign was positive in 8/11 patients.

**Conclusion:** Dengue fever in pregnancy is associated with thrombocytopenia. There is no leucopenia (relative leukocytosis of pregnancy). There is hemoconcentration and reduced pulse pressure due to decreased intravascular volume and leaky capillaries, however these changes cannot be detected in pregnancy (physiological hemodilution and increased intravascular volume). Management is antipyretics, strictly monitored intravenous therapy with platelet transfusion reserved for critical phases.

## ISSHID Abstract-337 Quorum Sensing Analysis of Pseudomonas aeruginosa and Effect of Bacteriocin against Biofilm Formation in Pseudomonas aeruginosa

### S.Priya Dharsini1, G.Bhuvaneshwari2, M.Kalyani3

#### Department of Microbiology, Saveetha Medical College and Hospital, Thandalam, Kanchipuram Dist, Tamilnadu, India

**Background:***Pseudomonas aeruginosa* is the commonest causative agent of Hospital Acquired Infection (HAI). It shows higher resistance to disinfectants and antibiotics due to production of Biofilm. There are various reviews on antibacterial effect of herbal extracts and nanoparticles against this superbug. Thus, this study aims to prove the effectiveness of bacteriocin against *P.aeruginosa* which may help in benefiting health care centre.

**Methods:** 100 strains of *P.aeruginosa* were collected from Saveetha hospital, Department of Microbiology for a time period of 6 months . Strains were identified by conventional methods. Bacteriocin was extracted by ammonium sulphate precipitation method and efficiency was checked by paper disc diffusion assay. Biofilm formation and quorum sensing analysis was performed by Microtitre plate method and Thin Layer Chromatography (TLC) respectively.

**Results:** In this study, 91% of *P.aeruginosa* strains were strong, 8% were intermediate and 1% were weak biofilm producers. From TLC analysis, 67% of the strains produced Acyl Homoserine Lactone molecules. Out of which, 49% has shown unknown analytes of Retardation factor (Rf) value greater than 1. The Rf values identified were 3 unsubstituted C4 (5%), 3 unsubstituted C6 (4%), 3 oxo C8 (3%), 3 oxo C4 (3%), 3 oxo C6 (2%), 3 oxo C1 (1%). Biofilm production before and after bacteriocin exposure was proved significant by paired t-test.

**Conclusion:** Quorum sensing molecules were confirmed to play a major role in biofilm formation. As bacteriocin was effective in controlling the biofilm formation, it can be incorporated in any surface disinfectant which helps in controlling transmission of superbugs.

## ISSHID Abstract-339 Biofilm formation, Antifungal susceptibility profiling and Polymerase chain reaction analysis of Candida albicans isolated from various clinical samples in tertiary care hospital

### Rajeswari.M.R^1,2^, Kalyani.M^2^, Hanumanthappa.A.R^1^ Vijayaraghavan. R^2^, Lava R^1^

#### ^1^Department of Microbiology, J. J .M. Medical College, Davangere, Karnataka, India; ^2^Saveetha Medical College, SIMATS, Chennai, Tamilnadu, India

**Background:** Candida species have emerged as main pathogens in both invasive and mucosal infections and show resistance to antifungal drugs. Knowledge of resistance to conventional antifungals and newer antifungal agents could support in better disease management and hence this study was carried out

To determine the biofilm formation, antifungal susceptibility and to find out the genotypic distribution of Candida albicans in a tertiary care center

**Methods:** This cross sectional study was done from July 2017 to June 2018 at J.J.M.Medical college Davangere. After getting Institutional Ethical Committee approval, 126 C.albicans were recovered from all clinical samples received in Microbiology laboratory. Biofilm formation was done by visual tube method. Antifungal susceptibility to Amphotericin B, Fluconazole, Voriconazole, Flucytosine and Caspofungin were performed by E – Test and genotyping was done by amplifying DNA fragment located in the 25S rDNA bearing the potential Group I introns.

**Results:** All 126 C. albicans isolates from different clinical samples were grouped into three genotypes by 25S rDNA. 68% belonged to genotype A, 22% to C and 10% to genotype B. Biofilm formation was observed more in Genotype A(69.8%) Resistance to fluconazole was observed in 16.3% of genotype A, 7.7% to B, and 3.7% to C. All strains belonging to genotype B and C were sensitive to other four antifungals except Genotype A

**Conclusion:** This study clearly shows that Genotype A is more virulent and resistant to antifungal drugs. Genotyping definitely helps in understanding the epidemiology, virulence and drug resistance of Candida species.

## ISSHID Abstract-346 Detection and typing of circulating Influenza A and B virus by real time reverse transcriptase polymerase chain reaction (RT PCR) at a tertiary care centre in Chennai

### Krithika Gopalakrishnan^1^, Divya Katta^1^, Ramya Barani^1^, Sudhabharathi Reju^1^, Monika Mani^1^, Preetam Arthur^2^, Shuba S^3^, Usha Rani G^4^, Padma Srikanth^1^

^1^Department of Microbiology, Sri Ramachandra Institute of Higher Education and Research, Chennai, India; ^2^Department of General Medicine, Sri Ramachandra Institute of Higher Education and Research, Chennai, India; ^3^Department of Paediatrics, Sri Ramachandra Institute of Higher Education and Research, Chennai, India; ^4^Department of Obstetrics & Gynaecology, Sri Ramachandra Institute of Higher Education and Research, Chennai, India

**Background:** Influenza accounts for 114667 cases and 8543 deaths in India over past decade. There were periodical occurrences of influenza in Chennai. Real time PCR is gold standard test recommended for influenza diagnosis. This study aims to determine Influenza A, B and H1N1 in participants with influenza-like illness (ILI) attending tertiary care center during recent outbreak.

**Methods:** This is prospective study conducted from 2018-2019. A total of 1755 throat swabs collected from participants with ILI. RNA was extracted using QIAamp Viral RNA Mini kit. Real time PCR was performed to detect Pandemic Influenza A/H1N1/2009 using ABI/HT/FAST/7900 (Applied Biosystems, USA). Influenza A negative samples tested for Influenza B by real time PCR using Artus Infl/H1LC/RG RT-PCR kit (Qiagen, Germany).

**Results:** Among 1755 samples analyzed, detection rate of H1N1 and Influenza A were 33% (n=575) and 13% (n=225) respectively. Paediatric age-group positivity rate was 27% for H1N1 and 25% for Influenza A. H1N1 detection rate in women of reproductive age was 41%. Increased detection rate was seen from October to December for H1N1 and September to December for influenza A. Among samples tested negative for Influenza A (n=80), the detection rate of Influenza B was 18%. Fever (90%) was the predominant symptom followed by cough (88%), sore throat (51%), chills &rigor (45%).

**Conclusion:** Co-circulation of Influenza A and B apart H1N1 depicts need for its detection for timely treatment and preventing complications. Increased positivity rate of H1N1 and influenza A in children and pregnant women indicates need for change in vaccination policy**"**

## ISSHID Abstract-347 Nuclear factor kappa B p65 detection by real-time PCR among HIV-1 infected individuals and healthy controls

### Sivasubramaniyan.G^1^, Rajesh Kannangai^2^, Ganesh Venkatraman^3^, Gopalsamy^1^, Sudha Bharathi^1^, Ramya Barani^1^, Monika Mani^1^, Padma Srikanth^1^

#### ^1^Department of Microbiology, Sri Ramachandra Institute of Higher Education and Research, Porur, Chennai; ^2^Department of Clinical Virology, Christian Medical College, Vellore; ^3^Department of Human Genetics, Sri Ramachandra Institute of Higher Education and Research, Porur, Chennai

**Backgroun**d: Nuclear factor-kappa B (NF-κB p65) is the transcription factor which helps in the replication of the HIV-1 virus by binding the site of NF-κB p65 in long terminal repeat (LTR) region of the virus. The aim of the study is to standardize and identify the expression of NF kappa B p65 by Real-time PCR among HIV-1 infected individuals and compare with viral load

**Methods:** HIV-1 RNA levels done using Artus HI virus-1 RG PCR kit (Qiagen, Germany). PBMC were separated from EDTA blood using Ficoll-Hypaque. RNA extraction was done using RNase mini kit (Qiagen, USA). cDNA was converted (Invitrogen, USA) and estimated using Nanodrop. Relative quantification of NF-κB p65 was done by using Power SYBR Green Master Mix kit (Thermo Fisher).

**Results:** A total of eight samples were included in the study, four were HIV-1 infected individuals and 4 were controls. Among HIV-1 participants, three had high plasma HIV-1 RNA level (>1000 copies/ml) and one had low HIV-1 RNA (650 copies/ml). Increased NF-κB p65 expression was observed in participant with high plasma RNA level (2-∆∆Ct range16-73) in contrast to participant with low plasma RNA level (2-∆∆Ct-6). We observed increased level of NF-κB p65 expression in a participant who had co-infection with CMV despite low HIV-1 RNA plasma level.

**Conclusion:** This is the first study to report detection of NF-κB p65 by SYBR green assay among HIV-1 individuals. Estimation of NF-κB p65 by real time PCR provides future prospective therapy of HIV-1 to suppress viral load by NF-κB inhibitors.

## ISSHID Abstract-380 Early detection of CMV by real-time quantitative PCR targeting immediate gene-ppUL83 in patients with active infection

### Ezhilarasi . V^1^, Yazhini . P^1^, Ramya Barani^1^, Sudha Bharathi R^1^ , Jayakumar. M^2^ , Padmasani. L, Padma Srikanth^1^

#### ^1^Department of Microbiology, Sri Ramachandra Institute of Higher Education and Research, Chennai, Tamil Nadu, India; ^2^Department Nephrology, Sri Ramachandra Institute of Higher Education and Research, Chennai, Tamil Nadu, India

**Background:** CMV belongs to herpes group of DNA virus, causes childhood infection and establishes latency in cells and organs. Though several genes (glycoprotein B (gB) and H (gH) DNA polymerase gene UL54, UL 126 etc) used for diagnosis, detection of active CMV using immediate (ppUL83)gene is useful to initiate early treatment. The aim of this study is early detection of CMV DNA by real-time qPCR.

**Methods:** Study participants who were suspected for CMV were enrolled. Blood/saliva/urine were collected and DNA were extracted by Qiagen DNA kit and quantitative real-time PCR was performed by TaqMan chemistry targeting ppUL83 using appropriate controls. The lower detection limit is <50 copies/ml. Amplification and analysis were done in SDS software (V2: 2.2; ABI 7900 HT).

**Results:** A total of 308 participants were enrolled (January 2016 to August 2019). Among the total, 57.5 % (n=177) were male and 42.5% (n=131) were female. About 52% of participants were 18-60 years, 21% (n=66) in 1-18 years and 20% with <1 year. Majority of CMV observed in pediatric patients 50% (n=20/40), post renal transplant recipients-PRTR 42% (n=40/95), general medicine 40% (n=10/25), hematology 38% (n=3/8), gastroenterology 33% (n=10/30), pulmonology 29% (n=2/7),both neurology & rheumatology 23% (n=3/13) and neonatology 19% (n=14/75). Patients who tested positive were initiated with appropriate treatment.

**Conclusion:** CMV causes infection in immunocompromised and PRTR’s. In our study, majority of CMV noted in pediatrics & PRTR’s. Early detection of active CMV using active gene like ppUL83 can prevent end organ disease and other outcomes by initiating preemptive therapy

## ISSHID Abstract-383 Diagnostic Analysis of *Neisseria gonorrhoeae* (NG) using Foldscope microscope: An inexpensive and point-of-care solution for screening microbes

### Uma Chaudhry, Sujata Adhana and Rajnish Kumar

#### Bhaskaracharya College of Applied Sciences, University of Delhi, Sector-2, Phase-I, Dwarka, New Delhi-110075, INDIA

**Background:** Foldscope Microscope, developed by Dr. Manu Prakash at Stanford University, has seen tremendous applications in the recent past. Being a relatively cost-effective (under $1.00) microscope made of paper, it could offer an alternative for diagnostic roles. It would be especially advantageous in resource poor regions of the world. We procured several foldscopes and assembled them (**“**fold on the dotted lines**”**) taking help from our under-graduate students. We analysed their application as a diagnostic tool for detecting N. gonorrhoeae, which causes a sexually transmitted disease - Gonorrhoea. There are large number of morbidity cases of patients infected by the organism.

**Methods:** Pure cultures of NG were procured from Safdarjung Hospital, New Delhi, India and evaluated using Gram Staining procedures. The idea is to standardise a diagnostic method which could be used at home as handy as a pregnancy detection kit.

**Results:** Preliminary studies clearly indicated presence of gram negative diplococci, characteristics feature of NG, using foldscopes. Further validation is required using patient samples directly. Speaking with physicians at the hospital, it was realized that most of the patients were asymptomatic and would consult doctor only when serious symptoms were observed.

**Conclusion:** The assay would help asymptomatic patients to evaluate themselves routinely for any infection. If the entire approach proves to be effective, it could help in developing powerful new way of routinely and almost immediately identifying contaminating microbes at the bedside. Moreover, successful imaging using cell phone would allow results to be shared with their physicians in a timely manner.

## ISSHID Abstract-387 Gut microbiota and its influence on Type 2 Diabetes Mellitus

### Shwathi Suresh Kumar^1^, Sribal Selvarajan^1^, Premalatha Pushpanathan^1^, Krishna G. Seshadri^2^, Padma Srikanth^1^

#### ^1^Department of Microbiology, Sri Ramachandra Institute of Higher Education and Research, Chennai, India; ^2^Department of Endocrinology, Sri Ramachandra Institute of Higher Education and Research, Chennai, India

**Background:** In India, 72 million people suffer from Type 2 diabetes mellitus (T2DM). This study was conducted to identify the composition of gut microbiota among T2DM and healthy controls and to correlate with Monocyte chemoattractant protein‑1 (MCP‑1) levels.

**Methods:** Stool and blood samples along with their clinical details and diet history were collected from healthy controls (n=10) and T2DM (n=10). Bacterial DNA was extracted from stool samples and amplified using fusion primers. Metagenomic analysis was performed using Ion Torrent Sequencing platform. Human MCP1 enzyme linked immunosorbent assay was performed on 17 serum samples.

**Results:** Phylum Proteobacteria-73% was abundant in T2DM and Firmicutes-69% in controls. The mean MCP1 levels among T2DM (256.5+106.1pg/ml) were significantly higher than controls (89.03+42.1 pg/ml) [P=0.0013]. Escherichia-13%, Serratia-10%, Citrobacter-9%, Faecalibacterium-7%, Enterobacter-6% (gram negative bacteria) were abundant in T2DM while Faecalibacterium-23%, Eubacterium-16%, Clostridium-10%, Vibrio-7% (gram positive bacteria) were predominant among controls. Escherichia was found to be increased 25 folds with MCP-1levels >200 than in ≤200. Escherichia (37%) and Vibrio (18%) were predominant among T2DM vegetarian and non-vegetarian participants respectively. In addition, we observed the shift in the genera towards gram negative bacteria (GNB) preponderance among post reproductive control women.

**Conclusion:** The higher gylcemic index associated with increased MCP-1 levels revealed the predominance of Escherichia.

## ISSHID Abstract-395 Role of CD4 count in PLHIV

### Thentral , Kavitha , Rajesh , Sundarajan , Vidhya Rani, Deepa , Neelaveni, Nirmala, Anandashankari, Thirunavukarasu

#### Microbiology, Dr.MGR University, Salem, India

**Background:** CD4 cell count has been essential component of HIV treatment and care programme since it provides the measurement of patients immune status, risk for opportunistic infections. It remains an important test for diagnostic decision making particularly in patient with advanced disease

**Methods:** This is a Cross sectional study in the department of Microbiology GMKMCH , Salem from April 2019 to August 2019. The persons who have been newly diagnosed with HIV infection in ICTC are tested for CD4 count by FACS Calibur . Those patients on ARTare tested for CD4 count at six months interval. An analysis of counts in naive and patient with ART was done. A correlation between viral load and CD4 count has been done in male patient with CD4 <100

**Results:** A total of 143 patients was diagnosed with HIV as per NACO guidelines, 30(20% ) had CD4 count below 100, 51 (35%) between 100 - 500, 29(20%) above 500 all the ANC (6) have CD4 around 500 . In patients on ART (916) , 11(1.2%) had CD4 below 100 ,283(31%) between 100 - 500 , 582(64%) above 500. In males on ART with CD4 count less than 100 , viral load varied from <20 - >2,28,989copies/ml

**Conclusion:** CD4 count in HIV infected , antiretroviral naive were below 500 in 79% of cases . The decrease in CD4 count during infection ,driven by immune activation not always correlates with viral load. So CD4 count along with viral load can be used for monitoring the treatment**"**

## ISSHID Abstract-402 Dissection of sensitivity and resistance of Chandipura virus to human complement

### Umerali Kunnakkadan, Reshma Koolaparambil Mukesh, Joydeep Nag, Nisha Asok Kumar, Sreenath Muraleedharan Suma, John B Johnson

#### Pathogen Biology lab, Rajiv Gandhi Centre for Biotechnology, Trivandrum

**Background:** The complement system, a potent innate immune barrier faced by animal viruses, can facilitate virus neutralization or the inverse, if the virus can override complement. Chandipura virus (CHPV), a vector-borne rhabdovirus causing fatal paediatric encephalitis, following local infection, disseminates to the central nervous system via blood. The study unravels the effect of human complement on CHPV.

**Methods:** CHPV was cultured and titrated in Vero cells. Complement susceptibility was tested with normal human serum (NHS) or heat-inactivated NHS by either varying concentrations or time followed by plaque assay. Complement dependency and pathways responsible for CHPV neutralization were dissected with respective complement-depleted/reconstituted NHS. Complement reconstitution was confirmed by haemolytic assays with rabbit or sheep RBC’s against NHS. Mechanism of neutralization and evasion were assessed with Transmission Electron Microscopy (TEM, Jeol) and sucrose gradient ultracentrifugation (Beckman)

**Results:** The concentration and time-dependent neutralization of CHPV by NHS (not HI) was complement dependent as CHPV was resistant to C3-depleted serum. Neutralization was classical pathway- dependent as EGTA treated serum, C4, and C1q-depleted sera had no effect contrary to Factor B-depleted serum. As CHPV was sensitive to C8 depleted NHS, terminal complement pathway had no role in neutralization. A marked shift in sucrose gradient suggested the mechanism of neutralization as aggregation, further validated by TEM. CHPV grown in HeLa cells were relatively less sensitive to NHS due to the presence of CD46 and CD55.

**Conclusion:** Although sensitive to complement, CHPV possesses mechanisms to evade complement.

## ISSHID Late Abstract-10 New-age microbicidal compounds from Cyanobacteria

### Damodharan Rajesh, Sannyasi Elumalai

#### Department of Biotechnology, University of Madras, Guindy Campus, Chennai, India

**Background:** In the past recent decades, antibiotic resistance is the alarming issue in the field of medical treatment. Many pathogenic microbes have developed multi-drug resistance against single and combination of antibiotics. Therefore, search for a novel and potential natural compound would be a solution to this global issue. Cyanobacteria are oxygenic, photoautotrophic prokaryotes evolved approximately 3.5 billion years ago. In recent years, cyclic and non-cyclic, non-ribosomal polypeptides and polyketides from cyanobacteria have promising microbicidal activity including antibacterial, antifungal and antiviral activities.

**Methods:** In our study, a symbiotic cyanobacterium has been isolated from the coralloid roots of a Cycad plant Cycas beddomei Dyer collected from the East Deccan dry evergreen forests. The cyanobacterium was identified based on the 16S rRNA gene sequencing based molecular characterization and gene sequence was submitted to NCBI and accession number was retrieved.

**Results:** The cyanobacterial polypeptides such as Spirulan, Dynemicin A, Esperamicin A, and Antiviridin exhibit potent antiviral activity on deadly human pathogenic viruses. Similarly, Ramolpanin, Enduracidin, Vancomycin, Tryocidine A, Bacitracin, and Surfactin possess strong antibiotic activities against human pathogenic bacteria. In our study, a cyanobacterium was isolated from the coralloid roots of Cycas plant followed by molecular identification. Based on the 16S rRNA sequencing it was identified as Desmonostoc muscorum (C.Agardh ex Bornet & Flahault) Hrouzek & Ventura and the NCBI GenBank accession number (MN381810) was obtained.

**Conclusion:** Therefore, cyanobacteria are considered as a novel source and cost-effective model for the production of novel anti-microbicidal compounds for the treatment of modern age diseases.

## ISSHID Late Abstract-13 Effect of probiotic curd on cardiovascular disease (CVD) risk factors and body mass composition in People living with HIV(PLWH) - A pilot study

### Amruthrao P^1^,Shivaprakash M^2^,Madhavi G^2^,Sree vennelarao^3^, MD.Khaleel Pasha^4^, V Srinivas^5^. R. Hemalatha^6^

#### ^1^Clinical Division,NIN, Hyderabad, India; ^2^Microbiology,NIN,Hyderabad, India; ^3^Pharmacy,Osmania university, Hyderabad,INDIA; ^4^Gastroenterology,Osmania General Hospital, Hyderabad,India; ^5^ART center,Osmania General Hospital, Hyderabad,India; ^6^Health Research, NIN, Hyderabad,India

**Background:** Probiotic food supplementation has been demonstrated to have promising results in improving the immune status i.e. CD4 cell profile in HIV subjects.

**Methods:** In present study we have selected about 100 patients (Males & Females) who were identified for HIV and were on ART were recruitedwith the age range of 20-50 in Osmania General Hospital, Hyderabad. The anthropometry parameters were recorded at baseline and after three months .Simultaneously blood samples were collected for analysis of CD4 cell counts, serum, ADA and Cholesterol levels. They were supplemented with indigenously prepared Probiotic curd .which contained the blend of *Lactobacillus bulgaricus*, *Streptococcus thermophilus* and *Bifidobacterium bifidum* daily for three months along with their regular diet.

**Results:** We have observed that all the anthropometric parameters were found to be significally increased. Further upon analysis of blood samples we could observe that there was 0.08 log increase in the CD4 cell counts with an average 5 point decrease in the supplementation group of HIV and a decrease in cholesterol levels from 185 to 158 mg/ml which was significant at 5% level. Similarly a significant decrease in ADA levels from 33.68± 19.44 to 24.94± 8.4 i.e. reverted back from abnormal to normal levels (15-25 U/dl) in these subjects after supplementation.

**Conclusion:** Our study has shown that the Probiotic curd supplementation was found to be effective on HIV subjects as there was an improvement in the anthropometric parameters and enhancement of CD4 cell counts with decrease in cholesterol and ADA levels and in these subjects.

## ISSHID Late Abstract-2 Biomarkers of aging in people living with HIV on long-term suppressive antiretroviral therapy

### Hemalatha Babu^1^, Kannan Thiruvengadam^1^, Vijila Sundaraj^2^, Murugesan S^1^, Madheswaran A^1^, Sangeetha A^1^, Karthika C^1^, Srikanth P Tripathy^1^, Luke Elizabeth Hanna^1^

#### ^1^Department of HIV/AIDS, National Institute for Research in Tuberculosis (ICMR), Chennai, India; ^2^Government Hospital of Thoracic Medicine (GHTM), Chennai, India

**Background:** Emerging data indicates that people living with HIV (PLHIV) show evidence of premature aging despite successful antiretroviral treatment (ART). Much of thisevidence is based on biomarkers of age-associated disordersidentified through high-throughput -omics studies. This pilot study attempted to identify easy-to-detect biomarkers associated with increased risk of aging-associated conditions in PLHIV.

**Methods:** The study was based on a retrospective case record review of 60 HIV-1 infected individuals on uninterrupted cART with the standard first line regimen for at least five years (median 6 years), and having stable CD4 counts >500cells/μL and undetectable viral load from Chennai, India. Longitudinal data on haematological and biochemical parameters obtained at 6-monthly intervals were analyzed and results are presented aspercentage frequency.

**Results:** The study identified higher number of abnormal events in Total Leukocyte Count (TLC) 74.5%, Mean Corpuscular Volume (MCV)- 80.6%, Alanine aminotransferase (ALT)- 31.6% andAlkaline phosphatase (ALP)- 39.7% in PLHIV. Some of the common opportunistic infections seen in this cohort included upper respiratory tract infection,oral ulcer, reproductive tract infectionand pelvic infection.

**Conclusions:** The biomarkers identified in this study are associated with aging-related diseases like cardiovascular disease, metabolic syndrome, cancer and hepatic disorder. Increased TLC has been associated with cardiovascular disease and longevity, increased ALT with metabolic syndrome and hepatic disorder, and decreased ALP with micronutrient deficiency and systemic inflammation. Validation of these findings in a larger cohort can help identify suitable biomarkers to assess risk of aging-associated diseases and morbidities in PLHIV.

## ISSHID Late Abstract-3 Comparative analysis of the performance of Quantiferon-TB Gold Plus and Quantiferon-TB Gold In–Tube Assays in a high TB prevalence setting

### Evangeline Ann Daniel, Sathyamurthi P, Saranathan R, Kannan T, Brindha B, Anuja R, Monica M, Hema Rajapushpam M, Luke Elizabeth Hanna, Srikanth Prasad Tripathy

#### Department of HIV/AIDS, National Institute for Research in Tuberculosis (ICMR), Chennai, India

**Background:** Interferon-γ release assay is a better alternative to TST for diagnosis of Latent Tuberculosis Infection (LTBI). Studies from low TB prevalence countries found good agreement between QuantiFERON-TB Gold Plus (QFT-Plus) QuantiFERON Gold In-Tube (QFT-GIT) assays, but there is little data comparing performance of the assays in a high-prevalence setting. This study aimed to compare the performance of QFT-Plus and QFT-GIT assays and evaluate additional cytokines as biomarkers for improving LTBI diagnosis.

**Methods:** QFT-GIT and QFT-Plus tests were performed on samples from 20 healthcare workers from Chennai. A Luminex-based multiplex assay was used to measure 45 cytokines/chemokines in quantiFERON supernatants. Receiver Operating Characteristic (ROC) curves of the significant cytokines were constructed to determine optimum cut-off levels for differentiating LTBI-positive and negative groups.

**Results:** A high degree of correlation was observed in the performance of QFT-Plus and QFT-GIT assays, as well as between the QFT-GIT TB tube and each of the two QFT-Plus TB tubes, and between the QFT-Plus TB1 and TB2 tubes (R >0.95). We identified 10 analytes that were significantly elevated in the antigen-stimulated tubes. ROC analysis revealed a good discriminative ability for IP-10, IL-2 and Granzyme B with sensitivity of 0.88-1 and specificity of 0.95- 1.

**Conclusion:** Observations revealed similar performance of both assays. The most robust TB antigen specific responses seem to be elicited in the QFT-Plus TB1 tube. IP-10, IL-2 and Granzyme B were identified as additional biomarkers that could be explored for their utility in LTBI diagnosis either individually or in combination with IFN-γ.

## ISSHID Late Abstract-5 Genetic diversity in the envelope gene of HIV -1 in plasma and PBMC of Tire-3 neutralizers

### K. Lucia Precilla^1^, Narayanaiah Cheedarla^2^, Ramesh Karunaianantham^1^, Anbalagan Selvaraj^1^, Hemalatha Haribabu^1^, Manohar Nesakumar^1^, R. K. Syed Nisar^1^, Kannan Muthuramalingam^1^, Srikanth Prasad Tripathy^1^, Luke Elizabeth Hanna^1^

#### ^1^Department of HIV/AIDS, National Institute for Research in Tuberculosis, Chennai, India; ^2^Department of Microbiology and Immunology, Emory University, Atlanta, USA

**Background:** HIV compartmentalisation and its impact on viral diversity is known to play an important role in HIV pathogenesis. We analyzed the genetic diversity between envelope sequences of HIV-1 obtained from plasma and peripheral blood mononuclear cells (PBMC) of Tire-3 neutralizers, in order to determine the impact of immune pressure on the viral sequences in the plasma and cells.

**Methods:** Proviral DNA and viral RNA were extracted from PBMC and paired plasma of 8 broad clade neutralizers. The full-length HIV-1 envelope gene (gp160) was PCR amplified and sequenced using an ABI 3100 Genetic Analyzer. The nucleotide sequences were analysed using SeqScape software, v2.5. Construction of phylogenetic tree and evolutionary analysis were performed using maximum likelihood algorithm and MEGA 7.

**Results:** Phylogenetic analysis revealed that the genetic distance between 7 of the 8 pairs of sequences was <0.05 with sequence identity ranging from 88 - 98%. One lone pair had a genetic distance of 0.2 and sequence identity of 70%. Phylogenetic tree constructed with the sequences showed that the plasma viral sequence of this unique sample did not cluster with its corresponding proviral DNA sequence.

**Conclusion:** We did not observe any significant correlation between genetic diversity of the viruses in the PBMC or plasma and the neutralization potency or type and number of epitope specificities in the samples. Careful analysis of the sequences of the unique sample with the greatest genetic distance showed the presence of APOBEC3G-mediated hypermutations in the proviral DNA.

## ISSHID: Oral Presentations Abstract-8 In vitro anti-tubercular and anti-HIV activity of silver nano particles synthesized using *Piper nigrum*

### Ruby Veronica N^1^, Karthik S^1^, Jerrine Joseph^2^, Rajasekar T^2^ , Radhakrishnan M^2^,Azger Dusthackeer V.N^3^, Mary Shamya A^2^, Luke Elizabeth Hanna^3^, Wilson Aruni^4,5^

#### ^1^Department of Biotechnology, School of Bioengineering, SRM Institute of Science and Technology, Kattankulathur, Tamil Nadu, India; ^2^Centre for Drug Discovery and Development, Sathyabama Institute of Science and Technology, Rajiv Gandhi Road, Chennai, Tamil Nadu, India; ^3^National Institute for Research in Tuberculosis, Chetpet, Chennai, Tamil Nadu, India; ^4^Sathyabama Institute of Science and Technology, Rajiv Gandhi Road, Chennai, Tamil Nadu, India; ^5^School of Medicine, California University of Science and Medicine, US Department of Veteran affairs, Loma Linda CA, USA

**Background:** In 2017 alone, around 10.4 million people fell ill with TB and 1.7 million died due to this disease. Among them, 53 million lives were saved in the year 2000 to 2017 through TB diagnosis and treatment. Multidrug-resistant (MDR) TB and extremely drug-resistant (XDR) TB are more commonly reported in patients particularly affected with HIV co infection.

**Methods:** In this study, we used three different medicinal plants (*Piper nigrum, Mangifera indica, Moringa oleifera*) for the green synthesis of nanoparticle and tested their in vitro efficacy towards the TB and HIV. The silver nanoparticles synthesized by biological method were characterized by UV-Vis spectroscopy, FT-IR, XRD and SEM.

**Results:** The silver nanoparticles formation were confirmed by colour change and UV spectrum and FT-IR spectrum was used to confirm the presence of functional group in the biomolecules. Spherical shape of the silver nanoparticles were determined by SEM. The crystalline nature was analysed by XRD. In this study Piper nigrum silver nanoparticles (AgNPs) have shown superlative activity of 98.5% Relative light unit (RLU ) reduction against M. tuberculosis H37Rv at 250 μg/ml concentration than the other two plants. IC50 values of silver nanoparticles were 14.33 μg/ml after 48 hours cell lysis post infection towards TZM-bl cells.

**Conclusion:** The synthesis of silver nanoparticle using medicinal plants by green synthesis approach has several advantages like eco-friendliness, efficiency, cost effectiveness and biocompatibility. In this green synthesis of *Piper nigrum* silver nanoparticles have potential for biomedical application particularly as anti-TB and anti- HIV agents.

## ISSHID Abstract-10 Estimation of Drug Dosage for Antiretroviral Therapy Based on Bacterial Foraging Guided Fractional Particle Swarm Optimization Technique

### Damodharan Rajesh^1^, Sannyasi Elumalai^1^, Gopal Rajesh Kanna^2^, Kasilingam Saravanan^2^

#### ^1^Department of Biotechnology, University of Madras, Guindy Campus, Chennai, India; ^2^Department of Plant Biology and Plant Biotechnology, Presidency College, Chennai, India

**Background:** In the past recent decades, antibiotic resistance is the alarming issue in the field of medical treatment. Many pathogenic microbes have developed multi-drug resistance against single and combination of antibiotics. Therefore, search for a novel and potential natural compound would be a solution to this global issue. Cyanobacteria are oxygenic, photoautotrophic prokaryotes evolved approximately 3.5 billion years ago. In recent years, cyclic and non-cyclic, non-ribosomal polypeptides and polyketides from cyanobacteria have promising microbicidal activity including antibacterial, antifungal and antiviral activities.

**Methods:** In our study, a symbiotic cyanobacterium has been isolated from the coralloid roots of a Cycad plant Cycas beddomei Dyer collected from the East Deccan dry evergreen forests. The cyanobacterium was identified based on the 16S rRNA gene sequencing based molecular characterization and gene sequence was submitted to NCBI and accession number was retrieved.

**Results:** The cyanobacterial polypeptides such as Spirulan, Dynemicin A, Esperamicin A, and Antiviridin exhibit potent antiviral activity on deadly human pathogenic viruses. Similarly, Ramolpanin, Enduracidin, Vancomycin, Tryocidine A, Bacitracin, and Surfactin possess strong antibiotic activities against human pathogenic bacteria. In our study, a cyanobacterium was isolated from the coralloid roots of Cycas plant followed by molecular identification. Based on the 16S rRNA sequencing it was identified as Desmonostoc muscorum (C.Agardh ex Bornet & Flahault) Hrouzek & Ventura and the NCBI GenBank accession number (MN381810) was obtained.

**Conclusion:** Therefore, cyanobacteria are considered as a novel source and cost-effective model for the production of novel anti-microbicidal compounds for the treatment of modern age diseases.

## ISSHID Late Abstract-11 Generation and characterization of HIV-1 chimeric viruses to define functional role of alterations in viral envelope sequence with time in natural infection to guide subtype C based immunogen design

### Ayushman Dobhal^1^, Nitesh Mishra^1^,Shaifali Sharma^1^, Sanjeev Kumar^1^, Ravinder Singh^2^, Bimal Kumar Das^2^, Rakesh Lodha^3^, Sushil K Kabra^3^, Kalpana Luthra^1^

#### ^1^Department of Biochemistry, All India Institute of Medical Sciences, New Delhi, India; ^2^Department of Microbiology, All India Institute of Medical Sciences, New Delhi, India; ^3^Department of Pediatrics, All India Institute of Medical Sciences, New Delhi, India

##### **Correspondence:** Kalpana Luthra

**Background:** HIV-1 envelope glycoproteins are targets of neutralizing antibodies and represent a key component for immunogen design. Within HIV-1 infected individuals, roughly 20% individuals generate broadly neutralizing antibodies against the envelope glycoprotein, a trimer of heterodimers of gp120 and gp41. The mapping of epitopes on viral envelopes vulnerable to immune evasion will aid in defining targets of vaccine immunogens and understanding the coevolution of plasma antibodies and viral variants in HIV-1 infected individuals provides key information for vaccine strategies. Herein, we report a HIV-1 infected pediatric elite neutralizer, AIIMS_330, who developed potent plasma bnAbs against HIV-1. In the timeframe in which AIIMS_330 gained significant increment in plasma potency, the viral variants developed resistance to V2-glycan targeting bnAbs.

**Methods:** Examination of autologous envelopes isolated in the timeframe of 90-month to 117-month from AIIMS_330 and chimeric viruses generated by swapping the V1V2 loops between viruses showed the association of V1V2 loop lengthening with development of neutralization to autologous plasma antibodies.

**Results:** The loop increments in the evolving viruses gained many potential N-linked glycosylation sites (PNGS). Of particular interest, the V1V2 loop lengthening did not provide resistance to V3-glycan targeting bnAbs as previously known. The epitopes were highly conformational as proved by a series of experiments.

**Conclusion:** Our data extend our knowledge on neutralizing epitopes associated with virus escape and potentially contribute to immunogen design.

## ISSHID Abstract-34 Identification of viral envelope glycoproteins with potential to serve as clade C based immunogens from HIV-1 infected pediatric elite neutralizers

### Nitesh Mishra^1^, Ayushman Dobhal^1^, Shaifali Sharma^1^, Muzamil Ashraf Makhdoomi^1,2,^ Bimal Kumar Das^3^, Rakesh Lodha^4^, Sushil K Kabra^4^, Kalpana Luthra^1^

#### ^1^Department of Biochemistry, All India Institute of Medical Sciences, New Delhi, India; ^2^Department of Biochemistry, Government College for Women, Cluster University Srinagar, Srinagar, India; ^3^Department of Microbiology, All India Institute of Medical Sciences, New Delhi, India; ^4^Department of Pediatrics, All India Institute of Medical Sciences, New Delhi, India

##### **Correspondence:** Kalpana Luthra

**Background:** The human immunodeficiency virus-1(HIV-1) envelope glycoprotein gp160 (envs), a trimer of heterodimer containing gp120 and gp41 subunits, mediates viral entry, and is the sole target of broadly neutralizing antibodies (bnAbs). A protective HIV-1 vaccine capable of inducing such bnAbs while inhibiting the induction of non-neutralizing antibodies (nnAbs) is needed, but few candidates, with BG505.SOSIP.664 being the prominent one, capable of doing so are known. Elite neutralizers are top 1% of HIV-1 infected individuals with highly potent plasma antibodies capable of neutralizing diverse strains of HIV-1. Envs from such elite neutralizers offer prominent candidates capable of inducing such bnAbs.

**Methods:** Herein, we describe two HIV-1 envs, 329.14.B1 and 330.16.E6, isolated from two pediatric elite neutralizers AIIMS_329 and AIIMS_330. TZM-bl cell-based neutralization assays were performed for 329.14.B1 and 330.16.E6 pseudoviruses using bnAbs, nnAbs, heterologous and autologous plasma.

**Results:** 329.14.B1 and 330.16.E6 pseudoviruses were susceptible to all major known bnAbs targeting the key sites of vulnerability and were capable of binding bnAbs in their native forms. Heterologous plasmas from HIV-1 infected infants potently neutralized both the pseudoviruses, suggesting antibodies capable of recognizing these envelopes exist in HIV-1 infected infants of Indian origin. Envs were resistant to soluble CD4 and non-neutralizing antibodies. Glycan shield mapping showed both envs with intact glycan shield, offering another advantage as completely glycan shielded viruses are associated with accelerated neutralization breadth development.

**Conclusion:** 329.14.B1 and 330.16.E6 HIV-1 clade C envelopes have the desirable features to be evaluated further as potential templates for clade C based immunogens.

## ISSHID Abstract-50 Out-of-pocket medical expenditure and health care seeking behavior among newly diagnosed persons living with Tuberculosis in Hyderabad and Bengaluru cities, in South India

### Rajaram SP^1^ , A Singarajipura^2^, A Rajesham^3^, M Manjula^2^, S Reuben^4^, S Amar^4^, KH Prakash^1^, S Shastri^2^, K Karthikeyan^1^, W Reynold^1^

#### ^1^Karnataka Health Promotion Trust, THALI, Bengaluru, India; ^2^Lady Willingdon State TB Centre, RNTCP, Bengaluru, India; ^3^ Commissionerate of Health and Family Welfare, RNTCP, Hyderabad, India; ^4^USAID India, Tuberculosis and Infectious Diseases, New Delhi, India

**Background:** India's End TB National Strategic plan envisages 'zero costs' as one of its goals. We examined the out-of-pocket expenditure and its association with health care seeking prior to TB treatment initiation.

**Methods:** A study was conducted under Tuberculosis Health Action Learning Initiative (THALI) funded by USAID among new sputum positive (NSP) adults who were diagnosed and initiated on TB treatment. We dichotomized the out-of-pocket expenditure around the median split and conducted multivariate logistic regression analysis, that included only the variables that had p-value ≤10% in the bi-variate analysis.

**Results:** Of the 451 NSP adults interviewed, 227 were from Bengaluru and 224 from Hyderabad. The median out-of-pocket expenditure before TB treatment initiation was Rs.1300 (US$ 18.6, IQR: Rs.400-Rs.2700) (Males Rs.1050 (US$ 15.0, IQR: Rs.360-2500); Females Rs.1635(US$ 23.4, IQR: Rs.520- Rs.3150). The median costs incurred included, consultation fee with median Rs.150 (US$ 2.1, IQR: Rs.20-Rs.300), laboratory tests Rs.250 (US$ 3.6, IQR: Rs.0-Rs.1100) and medicines Rs.500 (US$ 7.1, IQR: Rs.100-Rs.1120). Five or more visits (AOR: 9.44 95%CI: 4.67-19.09) to health care provider and who visited only a private doctor (AOR: 6.68; 95% CI: 0.99-45.28) were the factors that significantly influenced the high out-of-pocket expenditure.

**Conclusion:** To achieve the goal of ‘zero costs’, it is essential to create demand from the community for appropriate diagnostic tests and to engage with private sector to reduce costs due to increase in volumes. This will shorten the care seeking pathways thereby reducing both the number of consultations and the out-of-pocket expenditure

## ISSHID Abstract-61 Economic Evaluation and Budget Impact Analysis of Human Papilloma Virus (HPV) Vaccine for the Prevention of Cervical Cancer in India

### Yuvaraj Krishnamoorthy, Salin K Eliyas, Sharan Murali, Tanveer Rehman, Manikandanesan Sakthivel, Palanivel Chinnakali

#### Department of Preventive and Social Medicine, Jawaharlal Institute of Postgraduate Medical Education and Research (JIPMER), Puducherry - 605008, India

**Background:** Cervical cancer (CaCx) is the second most common cancer among women in India. Human Papilloma Virus (HPV) vaccination has been recommended by World Health Organization as an effective preventive strategy. Before its introduction in India, there is need to generate evidence on cost-effectiveness of this vaccine. Hence, we aimed to evaluate the impact and cost-effectiveness of a projected national quadrivalent HPV immunization program in India.

**Methods:** The study developed a model using UNIVAC software to compare CaCx disease and cost burden with and without such a program using Incremental Cost Effectiveness Ratio (ICER) for a hypothetical cohort of 10 year old adolescent girls from 2019 to 2028. Model inputs included measures of disease and cost burden, vaccine performance, coverage and cost. Program impact was expressed in terms of decadal number of CaCx cases, hospitalizations, deaths and disability-adjusted life-years (DALYs) averted. Sensitivity analysis was performed for ICERs in different vaccination scenarios.

**Results:** Vaccination would be cost-saving at the GAVI Alliance price of $4.50 per dose, which would result in an expenditure of $499 (INR 34,142) for averting per DALY which was less than one-time GDP per capita of India. Even with the most unfavourable scenario at $9.50 per dose, vaccination would be highly cost-effective with ICER $1250 (INR 85,527). Probabilistic sensitivity analysis found the ICER to be $678 (INR 46,390) with 95%CI: $524-$886.

**Conclusion:** A national quadrivalent HPV immunization program in India would prevent substantial morbidity, mortality and highly cost-effective at a range of vaccine prices.

## ISSHID Abstract-96 Role of stem cell-like memory T cells (TSCM) and memory T cell subsets in the host immune response to HIV

### Sivasankaran Munusamy Ponnan^1^, Kannan Thiruvengadam^1^, Hemalatha Babu^1^, Sujitha Kathirvel^1^, Manikannan Mathaiyan^1^, Thongadi Ramesh Dinesha^2^, Shanmugam Saravanan^2^, Kailapuri Gangatharan Murugavel^2^, Srikanth Prasad Tripathy^1^, Luke Elizabeth Hanna^1^

#### ^1^National Institute for Research in Tuberculosis (Indian Council of Medical Research), Chennai, India; ^2^YRG Center for AIDS Research and Education, Chennai, India

##### **Correspondence:** Luke Elizabeth Hanna

**Background:** TSCM cells are a unique subset of T cells that possess self-renewal capability and can differentiate into memory or effector T cell subsets. The role of TSCM cells in HIV infection and antiviral response has not been completely elucidated. The present study aimed to explore the role of TSCM cells and memory T cells subsets in the immune response to HIV exposure and infection.

**Methods:** The study enrolled 35 HIV exposed seronegative women (ESN), 10 HIV infected women on cART, 10 HIV infected women naive to ART, and 35 HIV unexposed uninfected healthy females (UH). Blood was obtained from all subjects and multicolour flowcytometry was performed to enumerate the proportion of TSCM cells and memory T cell subsets in the different groups. Samples were stained with the corresponding cocktail of monoclonal antibodies, acquired on a BD FACS Aria flowcytometer, and data was analyzed using Flowjo, v 10.5 (Treestar).

**Results:** We found significantly higher frequencies of EM and TE cells in the ESN group, particularly in the CD8+ compartment (p<0.05). Interestingly, the frequency of CD8+ TSCM was significantly higher in the ESN group than the HIV+ART+ and UH groups (p < 0.001). We also observed an increase in the frequency of CD4+ TSCM cells the ESN group, but the increase was not significant.

**Conclusion:** This observation indicates the potential contribution of TSCM, EM and TE cells to the early control of HIV infection. Hence, strategies to induce CD8+ TSCM cells would be highly beneficial for the prevention/control of HIV infection.

## ISSHID Abstract-98 Genetic diversity of *Mycobacterium tuberculosis* complex isolated from previously treated sputum smear positive patients with pulmonary tuberculosis

### Anagoni Srikar^1^, Venkataramana^1^, Alladi mohan^2^, PVGK Sarma^3^, Abhijit Chaudhury^1^

#### ^1^Department of Microbiology, SVIMS University, Tirupati, India; ^2^Department of Medicine, SVIMS University, Tirupati, India; ^3^Department of biotechnology, SVIMS University, Tirupati, India

**Background:***M. tuberculosis* drug resistance is a serious problem, especially among previously treated patients. We aimed to investigate the pattern of drug resistance and strain diversity among previously treated patients with sputum smear-positive pulmonary tuberculosis in a South Indian population.

**Methods:** A total of 97 patients with sputum smear positivity and who had previously undergone treatment were selected. Sputum was cultured and drug susceptibility testing was done for first line tuberculosis drugs. Spoligotyping was performed on genomic DNA using standard protocol.

**Results:** The 97 patients belonged to all age groups (10-90 years) with a mean age of 50 yrs; most common being in 61-70 yrs range(28.9%). Females were 40 (41.24%) and males 57 (58.76%). The isolates showed highest (48.4%) and lowest (6.2%) resistance to isoniazid and ethambutol respectively. Mono-resistance to isoniazid 14 (14.43%) was highest among all drugs. Significant difference (p<0.0001) was found between rifampicin and isoniazid resistant pattern. Spoligotyping results showed 34 different Spoligotyping patterns. Out of 97 isolates, 76 belonged to well defined lineages and 7 clusters; while the remaining 21 were singletons. The distribution of most common lineages were: Beijing-12 (12.37%), CAS1-Delhi- 9 (9.27%), EAI3-Ind – 20 (20.61%), EAI5- 13 (13.40%), and 7 each of Unknown and Orphan.

**Conclusion:** Mono-resistance to isoniazid was very high among the previously treated patients. The occurrence of 12.4% Beijing strains in South India is an important finding. Spoligotyping can be very useful to delineate the lineages of MTBC in circulation in a given geographic region.

## ISSHID Abstract-106 Role of hyperglycemia on macrophage effector function in pathophysiology of pulmonary tuberculosis

### Archana Singh^1^, Sudhasini Panda^1^, Diravyaseelan M^1^, Kalpana Luthra^1^,Naval Kishore^2^, Anant Mohan^3^, Neeraj Gupta^4^

#### ^1^Department of Biochemistry, All India Institute of Medical Sciences, New Delhi, India; ^2^Department of Medicine, All India Institute of Medical Sciences, New Delhi, India; ^3^Department of Pulmonary Medicine, All India Institute of Medical Sciences, New Delhi, India; ^4^Department of Pulmonary Medicine, VMMC and Safdarjung Hospital, New Delhi, India

**Background:** Epidemiological studies shows diabetes mellitus (DM) patients are more susceptible to tuberculosis (TB), but the underlying mechanism is unclear. Since macrophages are first line of defense against TB infection, it was hypothesized that hyperglycemia might cause innate immune dysregulation in terms of macrophage function and hence increased TB susceptibility.

**Methods:** Monocytes were isolated from 30 individuals from four groups namely “Active TB with uncontrolled Type 2 DM (HbA1c>7.5), Active TB without type 2 DM, Uncontrolled Type 2 DM only (HbA1c>7.5) and healthy individuals” and differentiated into macrophages. Recognition receptors namely CD14, CD11b, MARCO, TLR2 and CD206 were estimated using flow cytometry. Phagocytosis activity was measured using FITC labelled BCG using flow cytometry. ROS levels were estimated using DCFDA kit.

**Results:** Altered levels of recognition receptors were found in TB patients having diabetes (low CD11b and MARCO, high CD206) with p<0.01. Phagocytosis activity was also reduced in TB patients having diabetes compared to TB and diabetes alone (p<0.05). ROS levels were higher in DM and TB having DM compared to other groups (p<0.01), also ROS levels were significantly higher in TB having DM compared to DM group.

**Conclusion:** The altered levels of receptors in TB patients having diabetes might suggest immune dysregulation under hyperglycemia condition. This might be one of the factors for reduced phagocytosis activity in TB patients under hyperglycemic condition. Higher ROS levels in diabetic TB milieu points towards *M. tuberculosis* resistance against ROS. However, the result needs to be validated in more representative samples like alveolar macrophages.

## ISSHID Abstract-144 Analysis of genetic diversity of HIV-1 nef gene from individuals with HIV associated dementia

### Sushama Jadhav, Vijay Nema

#### ICMR-National AIDS Research Institute, Pune, Maharashtra, India

**Background:** HIV-1 Nef is a multifunctional accessory protein expressed early after infection. Nef polymorphisms is associated with cellular functions leading to neurological complications. Understanding the genetic diversity of HIV-1 nef gene can reflect its association with cellular factors for developing HIV associated dementia (HAD)

**Methods:** HIV-1 nef gene was amplified by PCR from HIV-1 positive plasma of five unlinked study patients from each group of with and without HIV associated dementia based on IHDS Score (International HIV dementia scale). PCR products were purified and sequenced using Genetic analyzer. HIV BLAST tool and seqscape software used for subtype and genetic diversity analysis respectively.

**Results:** Out of 10, 9 HIV strains are subtype C and one is BC recombinant. In non-dementia group (IHDS score > 9.5), ), there was a mixture of lysine (K) and arginine (R) at position 71 while in second group with dementia (IHDS score > 9.5), there is threonine (T) necessary for SH3 binding as nef interacts with the SH3 domain of Src tyrosine kinase and other signaling molecules. It is destabilizing signature position showing association in dementia patients. Furthermore, highly conserved FPD amino acid at 122-124 position is exposed as a loop and interacts with human thioesterase, influencing nef mediated endocytosis and downregulation of CD4 and MHC1. Additionally, conserved P73, R78, D87, T118 amino acids are essential for downregulation of CD4

**Conclusion:** Thus, genetic variation in nef gene translating into the change of functioning along with specific cellular counterparts might be the vital players in HAD development.

## ISSHID Abstract-154 Inhibition of Bacteria by Bimetallic nanoparticles derived from *Eucalyptus globulus*

### Ramavath Bharath Naik^1^, Ashok Kumar^1^, Elanchezhiyan Manickan^1^

#### ^1^Department of Medical Microbiology, Dr.ALM PGIBMS, University of Madras, Taramani Campus, Chennai

**Background:** Biosynthesis of gold (Au), Silver (Ag), Bimetal (Ag-Au) nanoparticles have attracted significant interest due to their environmental friendly, biocompatible and their potential application.

**Methods:** In this study, we investigated the synthesis of gold(Au), Silver(Ag), bimetal(Ag-Au) nanoparticles using leaf aqueous extract of Eucalyptus globulus, and also antibacterial activity has been studied using gram-negative and gram-positive organisms. The synthesized nanoparticles were characterized using UV-vis Spectrophotometry, FTIR, XRD, SEM, and TEM techniques.

**Results:** The characterization of nanoparticles was confirmed the nanoparticles crystalline in nature, surface morphology and responsible reduction biomolecules. The antibacterial activities were studied (Filter paper disk diffusion method) against Staphylococcus aureus, Pseudomonas aeroginosa, Streptococci feacalis, Proteus and E.coli. The Eucalyptus globulus leaf extract mediated Ag-Au bimetallic nanoparticles show high rate of antibacterial activity among the bacterial species when compared to Ag, Au nanoparticles.

**Conclusion:** The present finding discloses the Eucalyptus globulus mediated bimetallic nanoparticles had the best antibacterial activity.

## ISSHID Abstract-198 Detection of uncultivable newer bacterial species associated with persistent chronic infections while investigating the bacterial diversity in microbiome of used clinical devices

### Ashutosh kumar Amar^1^, Kalaivani Ramakrishnan^2^ Jagadish Menon^3^, K. Prashanth^1^

#### ^1^Department of Biotechnology, School of Life Sciences, Pondicherry University, Pondicherry, India; ^2^Department of Microbiology, Mahatma Gandhi Medical College and Research Institute, Sri Balaji Vidyapeeth – Deemed University, Pondicherry, India; ^3^Department of Orthopaedics, Jawaharlal Institute of Postgraduate Medical Education and Research, Pondicherry, India

**Background:** Chronic persistent device-related infections often tend to give culture negative results in microbiological investigation. The 16SrRNA gene sequence analysis and next generation sequencing (NGS) of metagenome was used to detect bacteria causing device-related infections that are culture negative

**Methods:** Around fifteen different device samples were collected from same number of patients and were parallelly subjected to microbiological culture, Sanger sequencing of 16SrRNA gene and NGS of device metagenome.

**Results:** Culture and 16SrRNA gene analysis yielded identical results only in four cases. Discrepancies were linked to eleven false-negative culture results when compared to sequencing results. Sequencing of 16SrRNA gene amplicons in 15 patients with severe infections identified a maximum of two bacterial species per sample and very rarely three species. DNA of some of the rare species identified through Sanger sequencing from the clinical samples are *Achromobacter xylosoxidans, Acinetobacter bouvetii, Comomonas spp. Cronobacter sakazakii, Herbaspirillum* spp., Rothia spp. and Sphingobacteriales bacterium. NGS of 16SrDNA microbiome from four device clinical samples was able to identify the presence of many rarely described human pathogens such as *Rothia mucilaginosa, Streptococcus infantis, Gemella haemolysans, Meiothermus silvanus, Schlegelella aquatica, Roseomonas pecuniae, Serratia nematodiphila* and *Enterobacter asburiae* along with common nosocomial pathogens such as *Stenotrophomonas maltophilia* and *A.baumannii*. This investigation detected rare bacterial species such as *M. silvanus*, *S. aquatic* and *S*. *nematodiphila* that are never reported in human infection.

**Conclusion:** Our results reveal that culture free, holistic metagenomic approach using NGS could be better method to identify the pathogens in culture-negative cases of chronic device-related infections

## ISSHID Abstract-206 Imidazolidinones “SPS-CHKN-KG” against Chikungunya virus

### Sharada P Swain^1,2^, Soma Chattopadhyay^3^

#### ^1^Department of Medicinal chemistry, Kinase green Industry Pvt Ltd, Bijaya Chandrapur, Paradeep, Odisha, India; ^2^School of Health Science, University of Petroleum and Energy Studies, Bidholi, Dehradun, India; ^3^Institute of Life Science, Nalco Square, Bhubaneswar, India

**Background:** Chikungunya fever has been reported in more than 40 countries of Asia, Africa, Europe and the Americas. The symptoms of the disease includes muscle/joint pain, joint swelling, headache, nausea, fatigue and rash. Following the fever, strong joint pain or stiffness occurs, which usually lasts weeks or months, but may last for years. The 2005 Indian epidemic, one of the most severe of the recent outbreaks, which affected 1.3 million people. There is no specific antiviral drug treatment for Chikungunya virus infection. The treatment is primarily at relieving the symptoms, which involves use of anti-pyretics, optimal analgesics in allopathy. Hence, there is urgent need for development of new drugs for Chikungunya treatment.

**Methods:** The desired imidazolidinones were synthesized and characterized by NMR and Mass spectroscopic data. The inbition studies was performed against Chikungunya virus in Vero cells. The inhibitory concentarion IC50 values were calculated.

**Results:** The compounds inhibited the Chikungunya virus with good potency. The compound SPS-CHKN-KG inhibited the virus with excellent potency (IC50 = 3.2μM).

**Conclusion:** The novel imidazolidinone analogs exhibited excellent in vitro inhibition activity against Chikungunya virus. The further in vitro studies are under progress to get EC50, CC50 and SI data.

## ISSHID Abstract-208 Transposon Tn6168 carrying insertion element ISEc28 trigger over expression of armA causing aminoglycoside resistance in *Acinetobacter baumannii*

### K. Prashanth, Ajit R. Sawant, Rajagopalan Saranathan, Sudhakar Pagal

#### Department of Biotechnology, School of Life Sciences, Pondicherry University, Pondicherry, India

**Background:** Nosocomial pathogen *Acinetobacter baumannii* is known for its propensity to acquire resistance genes through horizontal gene transfer. Mobilization of resistance determinants are usually with the help of mobile genetic elements like transposons, IS-elements and resistance islands. This study intended to detect the prevalence of aminoglycoside modifying enzyme (AME) genes and 16SrRNA methylase armA mediated aminoglycoside resistance mechanism in clinical isolates of *A. baumannii*.

Methods: MIC determination for aminoglycosides was performed by agar dilution method and PCR screening was used to detect AME genes. Strain PKAB07 that was genome sequenced (GenBank-ID: CP006963.1) was explored for the presence of IS-elements by IS-Finder.

Results: In silico analysis of genome of PKAB07 revealed presence of two resistance islands namely AbGRI1-1 and AbGRI3, in which transposon Tn6279 was part of AbGRI3. Besides, 23 IS-elements were found in PKAB07, wherein it was observed that ISEc28 is always present upstream of armA gene. In AbGRI3, armA is flanked by ISEc29 downstream and a disrupted ISEc28 upstream. The organization of ISEc28-armA-ISEc29 is widespread and highly conserved in many Gram-negative bacteria. Cloning of armA and armA along with upstream ISEc28 in *E. coli* using pUC19 vector performed to check the differential effects with respect to aminoglycoside resistance. The *E*. *coli* clone of armA along with ISEc28 showed high level resistance towards all the aminoglycoside antibiotics tested whereas the clone having armA alone remained susceptible to aminoglycosides.

Conclusion: Our study showed that ISEc28 behaving as a strong promoter leading to over expression of armA resulting in elevated aminoglycoside resistance

## ISSHID Abstract-210 Antibacterial & Antibiofilm potential of Chitosan Biopolymer (Medium Molecular Weight) Against Multi Drug Resistant (MDR) Bacterial Isolates of Diabetic & Non Diabetic Wound Ulcers

### Pradeepa Venkatachalam, P.Suganthi, M.Anandhi, Swathi M, Aneesha S

#### Department of Microbiology, Dr.ALM PGIBMS, University of Madras, Taramani, Chennai, India

**Background:** The spectrum of microbial flora and their multi drug resistance (MDR) were the leading cause of therapeutic challenges in diabetic patients. Biofilm is considered to be one of the important reasons for MDR. Hence, the present study was aimed to investigate the anti-biofilm activity of Chitosan biopolymer on MDR biofilm producing pathogens.

**Methods:** A total of 51 isolates, diabetic 21(41%) and non-diabetic 30(59%) were included in our study. Antibiotic sensitivity test (AST) and MIC for the isolates was performed as per CLSI guidelines.Standard microtitre plate method using crystal violet dye was used to estimate the biofilm production of the MDR isolates. Anti-biofilm property of chitosan biopolymer was determined using Biofilm Iinhibition Assay. Standard Broth Dilution method was employed to determine the antibacterial effect (MIC &MBC) of chitosan biopolymer for the MDR isolates.

**Results:***Staphylococcus aureus* (52%) and *Klebseila* sps (30%) was found to be the common pathogen in diabetic and non-diabetic wound ulcers, respectively. 59% of the total isolates in our study were observed as MDR. Among the MDR isolates, 66% were characterized as strong biofilm producers. Chitosan biopolymer showed at good anti-biofilm activity against MDR isolates. Chitosan biopolymer found to have antibacterial activity with a MIC value of 64 μg/ml and MBC value of 128 μg/ml for MDR isolates.

**Conclusion:** Antibacterial and anti-biofilm activity of chitosan found to be a promising antibacterial for MDR pathogens. However, further clinical studies may warrant the use of chitosan biopolymer as an alternative therapeutics for diabetic wound ulcers. "

## ISSHID Abstract-214 Molecular epidemiology of acute respiratory viral infections among children in Chennai, South India

### Prabu D^1^, Karunya D^1^, Sree Nethra B^1^, Kiruthika K^1^, Vidhya NM^1^, Anusha H^1^, Kalpana Sivasambo^2^, Sarath Balaji^2^, ElilarasiRagunathan^2^, Suganya Mahalingam^2^

#### ^1^Department of Microbiology, Dr.ALM PGIBMS, University of Madras, Taramani, Chennai, India; ^2^Department of Pediatric Pulmonology, Institute of Child Health and Hospital for Children, Egmore, Chennai, India

**Background:** Viruses are the frequent cause of acute respiratory infections and account for high morbidity and mortality. Despite being a major cause of death in developing countries like India, the role of respiratory viruses in childhood ARI is poorly investigated. This study was done to determine the burden of common viral etiologies of ARI among children in and around Chennai region.

**Methods**: A total of 101 specimens (55Nasopharyngeal aspirates and 46 nasal swabs) were collected from children (aged 0 to 10 years) with symptoms of ARI. RNA was extracted from the specimens and reverse transcription was performed. The cDNA was subjected to detection of human respiratory syncytial virus, influenza viruses, human metapneumovirus, human rhinovirus and human parainfluenza viruses by conventional and real time RT-PCR.

**Results:** Among the enrolled children, 54.5% were diagnosed with LRTI and 45.5% had URTI. HRSV was the most frequent virus, detected in 29.5% (53.3% of RSV A and 46.7% of RSV B) of the children followed by human parainfluenza virus (16.8%; 94.1% of HPIV 3 and 5.8% of HPIV 4). Influenza B virus (Yamagata lineage) and human rhinovirus were detected in one sample each (1%). Influenza A virus, human metapneumovirus and human parainfluenza viruses types 1 and 2 were not detected in any of the samples.

**Conclusion:** HRSV contributed to majority of the ARI among children in this study. Follow-up of viral ARI over a long period allows better understanding of the epidemiology and role of respiratory viruses in ARI.

## ISSHID Abstract-243 Switching off the functional relation between HIV-1 Integrase and LEDGF/p75 using in silico approach

### Umesh Panwar^1^, Sanjeev Kumar Singh^1^

#### ^1^Computer Aided Drug Design and Molecular Modelling Lab, Department of Bioinformatics, Alagappa University, Karaikudi, Tamil Nadu, India

**Background:** Since the century begins, viruses have remained a global health concern. Currently, 36.9 million people globally were living with HIV in 2017 & 35.4 million people have died from AIDS-related illnesses. Within three major viral enzymes (HIV-1 protease, HIV-1 reverse transcriptase and HIV-1 integrase [IN]), Integrase is an essential enzyme of the viral replication in HIV,-1 catalyzes the integration of viral genetic material with the host genome. Integrase strongly depends on the binding of host cellular cofactors for the replication process. Among them, the host factor lens epithelium-derived growth factor (LEDGF/p75) contributes to the regulation of gene expression by tethering the HIV-1 pre-integration complex to the chromatin via direct involvement with IN. Thus, the importance of the protein-protein interaction between HIV-1 IN and LEDGF/p75 has been drawn the interest in the prediction of the potent inhibitor to combat the viral infection.

**Methods:** Herein, in silico platform of E-pharmacophore based screening with the implementation of molecular docking, ADME/T, binding free energy and dynamics simulation following with PCA strategies has been applied to develop a potent inhibitor to diminish the virus-host relation.

**Results:** The outcome represented that the identified molecule with acceptable pharmacological properties and higher binding affinity is able to inhibit the IN-LEDGF/p75 relation.

**Conclusion:** We anticipated these results provide the small molecule may be future therapeutic agents to inhibit the protein-protein interaction of integrase with improved activity and stability and increase the life span of infected people.

## ISSHID Abstract-253 Rapid detection of common viruses in cerebrospinal fluid by high resolution melt analysis

### Helen Hencida Thangamony^1,2,^ Ravindran Kumar^2^, Chinnappan Palaniappan Thangavelu^2^, Mani Mariappa^2^, Berlin Grace Viswanathan Mariammal^3^, Kavitha Subbiah^3^, Dheepa Muthusamy^4^ & Kootallur Narayanan Brahmadathan^2^

#### ^1^Department of Biotechnology, School of Agriculture and Biosciences, Karunya Institute of Technology and Sciences, Karunya Nagar, Coimbatore, Tamil Nadu, India; ^2^Division of Molecular Biology, Microbiological Laboratory Research & Services Pvt Ltd, Coimbatore, Tamil Nadu, India; ^3^Department of Biotechnology, School of Agriculture and Biosciences, Karunya Institute of Technology and Sciences, Karunya Nagar, Coimbatore, Tamil Nadu, India; ^4^Department of Microbiology, Government Medical College & ESI Hospital, Coimbatore, Tamil Nadu, India

**Background:** High resolution melt analysis (HRMA) done on a PCR platform is a sensitive tool for the rapid identification of microbes. This study describes the standardization of HRMA for the detection of 5 common viruses, Enterovirus (EV), HSV-1, HSV-2, VZV, and CMV in CSF.

**Methods:** 950 consecutive CSF from suspected cases of meningoencephalitis were subjected to HRMA on a PCR platform using pathogen specific primers and standard protocols. Primers were checked for various parameters using known concentrations of DNA / RNA specific to the framed panel. The melt profile and Tm values were analyzed by HRM Software. All positive samples were tested with a commercial kit (Fast Track Diagnostics, Luxembourg) and analyzed through statistical calculators.

**Results:** The primers were specific to each virus with no cross reactions while the melt curves and Tm were distinguishable from one another. Time to detection was 3 hours and LOD were 125, 88, 162, 76 and 35 copies/mL for EV, HSV-1, HSV-2, VZV and CMV respectively (p<0.0001). Seventy two (7.6%) of the 950 samples were positive for EV (n= 13), HSV-1 (30), HSV-2 (3), VZV (9) and CMV (17). One sample identified both EV and CMV while another one identified HSV-1 and CMV. 70 CSF were positive by both HRMA and the kit. Two samples positive for EV by the kit with low viral loads were negative by HRMA.

**Conclusion:** This in-house standardized HRMA is an ideal, cost-effective and highly sensitive test for the rapid detection of the five most common agents causing Meningoencephalitis.

## ISSHID Abstract-265 Detection of *Neisseria gonorrhoeae* in clinical samples – the leap ahead from conventional methods to the molecular approach

### Ketan Priyadarshi, Uneza Husain

#### Department of Microbiology, Institute of Medical Sciences, Banaras Hindu University, Varanasi, Uttar Pradesh, India

**Background:** Prompt and accurate diagnosis of STIs is necessary. NAAT is the test of choice for gonorrhea. CDC advocates supplemental testing with PCRs having different targets, creating a need for Multiplex PCR assays. The study aimed to compare a novel in-house Multiplex Nested PCR (MNPCR) targeting two genes simultaneously for the detection of gonococci from clinical samples with culture isolation and microscopy.

**Methods:** 116 symptomatic subjects were included. Urethral swabs from males(n=12) and endocervical swabs from females(n=104) were subjected to Gram staining and gonococcal culture on chocolate agar and Modified Thayer-Martin media, with AST by Kirby-Bauer disc diffusion using CDS & CLSI guidelines and MIC by E-test. DNA extraction was done using the phenol-chloroform method. MNPCR was performed targeting porA pseudogene and opa gene using novel designed primers. Amplicon detected by agarose gel electrophoresis using UV transilluminator.

**Results:** Culture was positive in 41.67%(5/12) males, in whom GNDC was also seen on Gram stain, but none in females. A total of 33.62%(39/116) subjects [29.8%(31/104)females and 66.7%(8/12) males] were positive for Neisseria gonorrhoeae in MNPCR. 3/12 males and 31/104 females were detected only by PCR. Considering PCR as standard, sensitivity, specificity, PPV and NPV of culture and Gram stain was 12.82%, 100%, 100%, 69.37% in total, and 62.5%, 100%, 100%, 57.14% in males. Incorporation of two different gene targets improved detection.

**Conclusion:** PCR based diagnosis of *Neisseria gonorrhoeae* infections should be encouraged. This novel MNPCR may be used in management of both symptomatic and asymptomatic cases of gonorrhea, particularly among high-risk groups.

## ISSHID Abstract-269 Adherence to Isoniazid Preventive Therapy and its Determinants among people living with HIV

### Vikas Prabhu, Basavaprabhu Achappa

#### Dept. of Internal Medicine, Kasturba Medical College Mangalore, Karnataka, India

**Background:** Isoniazid preventive therapy (IPT) is a preventive therapy measure and a key public health intervention in mitigating the risk of TB in HIV. However, despite its implementation, adherence to IPT and its barriers remain unknown to a large extent. Investigating into adherence rates and the factors influencing it can help better address this life saving intervention.

**Methods:** A descriptive longitudinal study was conducted over two years and a total of 320 participants were included. Adherence was measured by self-report of intake of tablets at the end of the therapy along with the monthly ART documentation. A standardized semi-structured questionnaire was used to collect all the relevant information. Questionnaire covered aspects on TB treatment and care, HIV related stigma, medical provider relations, social support and socioeconomic status.

**Results:** Mean adherence rates were 83.75%. Of 320 patients, 268 were adherent and 19 were non adherent. Isoniazid related side effects were reported by 50; 33 of whom were withdrawn. Prior history of TB was noted in 43(13.43%). A detailed analysis across different variables among the adherent and non-adherent groups was done.

**Conclusion:** The level of adherence to IPT was high.A list of facilitators and barriers were identified. Same is essential for gauging the current situation and which can help to design and deliver appropriate public health measures to address it.

## ISSHID Abstract-277 In vitro anti-HIV-1 activity of sulphated polysaccharide from three different marine macroalgae (seaweeds)

### Rajesh Kanna Gopal^1^, Preethy P. Raj^2^ and Elumalai Sanniyasi^2^

#### ^1^Department of Plant Biology and Plant Biotechnology, Presidency College (Autonomous), Chennai, India; ^2^Department of Biotechnology, University of Madras, Guindy Campus, Chennai, India

**Background:** Highly active antiretroviral therapy (HAART), is the only remedial measure for HIV-AIDS treatment as recognized by WHO. About 44 antiretroviral drugs have been approved by FDA. However, they are associated with life-threatening side effects and drug resistance in first-line treatment. Sulfated polysaccharides (SP) from marine macroalgae are direct inhibitor of retroviruses, and considered as a “new generation antiretroviral drug”. We have investigated a SP, fucoidan from three macroalgae, for in vitro anti-HIV-1 activity.

**Methods:** Fucoidan was extracted from three macroalgae *Padina pavonica* (PP), *Stoechospermum marginatum* (StM) and *Spatoglossum macrodontum* (SpM). Characterized by RP-HPLC and FT-IR, purified by ion-exchange chromatography and evaluated for anti-HIV-1 activity by gag p24 assay.

**Results:** Total yield of crude fucoidan was 9.58 %, 26.2 % and 6.44 % in PP, StM and SpM respectively. Fucose (sugar moiety) in fucoidan was used as standard for the confirmation of fucoidan in our extracts by RP-HPLC. Similarly, FT-IR confirms the sulfate groups. The total fucoidan obtained by ion-exchange purification was 0.713 μg/ml, 0.871 μg/ml and 1.045 μg/ml from PP, StM and SpM respectively. The maximum inhibition of HIV-1 were 95.65 % in PP, 85.65 % in StM and 89.56 % in SpM. The estimated IC50 values were 0.295 μg/ml, 0.346 μg/ml and 0.002 μg/ml in PP, StM and SpM respectively.

**Conclusion:** Our study concludes that the fucoidan extracts of all the three macroalgae exhibits promising HIV-1 inhibiting activity. Furthermore, extensive research work is essential to develop this bioactive compound for clinical treatment of HIV patients.

## ISSHID Abstract-288 Interaction of HIV-1 and HIV-2 Capsid variants with huTRIM5α SPRY domain and its association with pathogenesis

### Veena V Ramalingam^1^, Subramanian Suganya^2^, G John Fletcher^1^, Priscilla Rupali^3^, George Varghese^3^, Susanne Pulimood^4^, Lakshmanan Jeyaseelan^5^, Balaji Nandagopal^2^, Gopalan Sridharan^2^, Rajesh Kannangai^1^

#### ^1^Department of Clinical Virology, Christian Medical College, Vellore, Tamil Nadu, India; ^2^Sri Sakthi Amma Institute of Biomedical Research, Sri Narayani Hospital and Research Centre, Sripuram, Vellore, Tamil Nadu, India; ^3^Department of Infectious Diseases, Christian Medical College, Vellore, Tamil Nadu, India; ^4^Department of Dermatology, Christian Medical College, Vellore, Tamil Nadu, India; ^5^Department of Biostatistics, Christian Medical College, Vellore, Tamil Nadu, India

**Background:** HIV-2 is less pathogenic and highly susceptible to host factor huTRIM5α (tripartite motif-containing protein) compared to HIV-1. This study was aimed to examine amino acid variations in the HIV-1 and 2 capsid gene that influences the susceptibility to TRIM5α.

**Methods:** This study was approved by the Institutional Review Board (Min. No. 8798). The HIV-1 and 2 capsid sequences contained variations as evidenced by sequencing of strains from treatment naive individuals. Molecular docking was done using ClusPro and Hex docking programs. HIV-1 and 2 RNA quantification was carried out using an Abbott real-time and Exavir RT activity assay respectively.

**Results:** The sequence analysis on HIV-1 (n=59) & HIV-2 (n=14) capsid gag gene identified 41 (17.8%) and 8 (4.5%) SNPs respectively. The variations in the HIV-2 capsid gene was significantly lower than HIV-1 (p=0.02). The molecular docking analysis showed HIV-1 reference strain used V1 loop while, HIV-2 used V3 loop of huTRIM5α for interaction. HIV-1 strains with A116T SNP used V3 loop of huTRIM5α for binding but there was no difference in susceptibility. HIV-2 strains with V81A SNP used V1 loop which is shown to be associated with the reduced activity of huTRIM5α as evidenced by increased HIV-2 viral load.

**Conclusion:** This is the first study which documents the difference in the usage of the loop between the two HIV types for interaction with huTRIM5α. Variations in the HIV capsid protein result in an alteration in the binding to the restriction factor huTRIM5α thereby; it may affect progression of the disease.

## ISSHID Abstract-322 Cloning of FliC Gene from *Salmonella enterica* serovar Typhi in the Expression Vector pT1NX and Evaluation of its Expression in *Lactobacillus rhamnosus*

### Richard P^1^, Nirmala P^1^ and Dinesh Kumar^2^

#### ^1^PG and Research Department of Microbiology, Mohamed Sathak college of Arts and Science, Chennai-600119, India; ^2^Swim Biopharma, Vinayagapuram, Chennai-600117, India

**Background:** Flagellin is the major protein constituent of Salmonella sp. flagella, and a mammalian toll-like receptor 5 (TLR5) agonist. Previous research indicates that antibodies against the FliC protein induced in mice models can provide protection against Salmonella challenge. In the present study, we cloned the flagellin gene (FliC) of *Salmonella enterica* serovar Typhi into pT1NX plasmid vector and evaluated its expression in *Lactobacillus rhamnosus* (MTCC 1408).

**Methods:** The complete sequence of FliC gene was constructed through cDNA from Salmonella enterica serovar Typhi. The constructed gene was cloned into pT1NX plasmid vector using NaeI and SpeI restriction enzymes. L. rhamnosus cells were transiently transformed with pT1NX-FliC vector by electroporation. DNA sequencing and agarose gel electrophoresis methods were used to reconfirm the positive colony which holds pT1NX-FliC gene. The recombinant FliC protein (rFliC) was separated using SDS-PAGE gel.

**Results:** The full protein-coding region of the *Salmonella enterica* serovar Typhi FliC gene containing 1518 nucleotides was successfully cloned into *L*. *rhamnosus*. Cloning of the FliC gene was confirmed by DNA sequencing and agarose gel electrophoresis. The rFliC protein was secreted in the culture medium in low quantity. The results indicated that the rFliC containing 506 amino acids with a molecular mass of 53 kDa in SDS-PAGE gel.

**Conclusion:** The findings of the present study indicated that *L*. *rhamnosus* is able to express Salmonella FliC protein. Recombinant FliC-expressing *L. rhamnosus* will be studied in preclinical models to test the in vivo efficacy and safety profiles as an oral typhoid vaccine."

## ISSHID Abstract-330 Incidence of cytomegalovirus drug resistance among allogeneic stem cell transplant recipients from a tertiary care centre

### Santhosh Kumar Duraisamy^1^, Shoba Mammen^1^, Rajesh Kannangai^1^, Aby Abraham^2^, Vikram Mathews^2^, Biju George^2^, Alok Srivastava^2^, Asha Mary Abraham^1^

#### ^1^Department of Clinical Virology, Christian Medical College, Vellore -632004, Tamil Nadu, India; ^2^Department of Clinical Hematology, Christian Medical College, Vellore -632004, Tamil Nadu, India

**Background:** CMV reactivation is a major cause of post-transplantation morbidity and mortality among stem cell transplant recipients. Ganciclovir is employed as first line antiviral therapy. In these patients cidofovir and foscarnet are used in whom resistance to ganciclovir develops.

**Aim:** To determine the incidence of CMV drug resistance (UL97 & UL54 gene) among allogeneic stem cell transplant recipients admitted to a tertiary care hospital in South India.

**Materials & Methods:** Blood samples were collected in K2-EDTA tubes from patients (n=178) admitted under the Department of Hematology. Post-transplant CMV reactivation was monitored using an accredited in-house real time polymerase chain reaction (PCR). In-house conventional PCRs targeting the UL54 (DNA polymerase) and UL97 (viral kinase) genes of CMV were developed to screen patients with increasing viral load for more than 7-14 days even after antiviral treatment.

**Results:** Of 178 patients recruited, 86(48.31%) recipients had CMV reactivation post-transplantation, 9(10.4%) of whom recovered spontaneously. The rest (n=77) were given intravenous ganciclovir. Drug resistance was suspected in 4/77(5.19%) patients. UL97 did not show any significant mutations. The following mutations were found in the UL54 gene: L655S, A688V, G874R, T1122A, V1164A.

**Conclusion:** Mutations in the UL97 gene are the most frequent cause for drug resistance with ganciclovir treatment. However, no mutations were observed in the current study. Mutations in the UL54 region usually lead to multi-drug resistance. In the current study, two novel mutations (T1122A and V1164A) were seen in 3/4 patients with ganciclovir resistance. However, further studies are needed to validate this finding.

## ISSHID Abstract-349 Treatment response to direct acting antivirals in HCV infected patients-experience from a tertiary care centre

### Suresh P^1^, Raghavendran A^1^, Karthick N^1^, Calvin JD^1^, Abraham P^1^

#### ^1^Department of Clinical Virology, Christian Medical College, Vellore, India – 632004

**Background:** Hepatitis C Virus (HCV) infection is estimated to affect 185 million population in worldwide with India having a prevalence of 0.09-5.2%. The aim of this study was to assess treatment response against currently available direct acting antivirals among HCV infected patients.

**Methods:** A total of 199 patients were measured for HCV RNA virus load using Abbott Real-time PCR at baseline followed by sustained virological response (SVR) measured 3 months after completion of direct acting antiviral therapy (DAA). Among the 199 patients 174 were treated with exclusively DAA (SOF+LDV, SOF+DAC, SOF+VEL, SOF+RBV) and 25 patients were started on DAA therapy due to failure of prior treatment. These 25 patients were previously treated with Peg-IFN+ribavirin or SOF+RBV or IFN free DAAs.

**Results:** Among 174 patients who received exclusively DAA therapy, 169/174 (97.13%) patients were achieved SVR. Among there 5/174 (2.87%) patients did not achieve SVR. Out of 25 patients who failed SVR on previous therapy, 22/25 (88%) patients were achieved SVR. Among there 3/25 (12%) patients did not achieve SVR. Overall, 191 of 199 patients attained for SVR, 8/199 (4.1%) patients failed to achieve SVR. The median viral load for these 8 relapsed patients was 5.3 log IU/mL.

**Conclusion:** In our study 191/199 (95.9%) patients attained a very high rate of SVR against DAA therapy. However, patients who failed to achieve SVR need to be assessed for the resistance associated substitutions in the target region of these DAAs.

## ISSHID Abstract-353 Drug Resistant Extrapulmonary Tuberculosis : Tip of the Iceberg?

### Emma Manuel, S. Thasneem Banu, K. G. Venkatesh, U. Umadevi, J. Euphrasia Latha

#### Institute of Microbiology Madras Medical College, Chennai

**Background:** India contributes to 24% of the global burden of Tuberculosis with almost 2 million new cases added every year. Of the 1.5 million cases reported to the Revised National Tuberculosis Control Programme (RNTCP) , 10-15% Extrapulmonary (EPTB) . EPTB constitutes 15-20% of all cases of Tuberculosis in immunocompetent patients and upto 50% in people living with HIV and AIDS (PLHA). This study aims to estimate the prevalence of Extrapulmonary Tuberculosis in PLHA and determine the drug resistance pattern of the isolates obtained.

**Methods:** This is a one year ( March 2018- February 2019 ) long cross sectional study done at Institute of Microbiology Madras Medical College on 1000 Extrapulmonary samples obtained from PLHA with clinical/radiological suspicion of EPTB. The samples were subjected to AFB microscopy, CBNAAT and liquid culture (MGIT 960) by Standard Protocol and Phenotypic Drug Susceptibility Testing was done using MGIT 960.

**Results:** Of the 1000 Extrapulmonary samples received, 6% were positive by AFB Microscopy, 19% we're positive by CBNAAT and 16% were positive by liquid culture which were subjected to Drug Susceptibility Testing using MGIT and 19% of the isolates we're Multidrug Resistant (MDR). The specimen wise MDR prevalence was found to be 33% in lymph nodes, 30% in CSF and 17% in Urine and nil among pus , fluids and biopsy specimen.

**Conclusion:** There is a need to increase the access to culture and DST for Extrapulmonary specimen in high burden settings to know the extent of the Iceberg.

## ISSHID Abstract-362 Single nucleotide polymorphism (T40I) in different clinical strains of glycoprotein E (ORF 68) of Varicella Zoster virus (VZV) in a resource limited setting

### Ilakkiya Arumugam^1^, Padma Srikanth^1^, Monika Mani^1^, Ramya Barani^1^, Gopalsamy Sarangan^1^, Ganesh P^2^, Madhavan K^3^, Padmasani Venkat Ramanan^4^, Ravi Mahalingam^5^, Maria Acena Nagel^6^

#### ^1^Department of Microbiology, Sri Ramachandra Institute of Higher Education and Research, Chennai, India; ^2^Department of Medical Gastroenterology, Sri Ramachandra Institute of Higher Education and Research, Chennai, India; ^3^Department of General Medicine, Sri Ramachandra Institute of Higher Education and Research, Chennai, India; ^4^Department of Paediatrics, Sri Ramachandra Institute of Higher Education and Research, Chennai, India; ^5^Department of Neurology, University of Colorado School of Medicine, Aurora, Colorado, USA; ^6^Department of Neurology & Ophthalmology, University of Colorado School of Medicine, Aurora, Colorado, USA

**Background:** Varicella Zoster virus (VZV) has long been considered to have a stable genome. Identification of a genetically variant strain has led to reconsider the presumption of VZV immutability. The study was aimed to identify mutations in open reading frame-68(ORF-68), a highly conserved gene that expresses only during active infection among clinical strains of VZV causing meningoencephalitis, inflammatory bowel disease and typical varicella.

**Methods:** The study was approved by Institution-ethics-committee. DNA was extracted from colon biopsy (IBD(n=15), control group(n=10), cerebrospinal fluid(n=40) and vesicular fluid(n=1) by DNA blood mini kit (Qiagen, Germany). Conventional PCR was performed using specific primers to amplify ORF-68(1876bp). The amplified products were sequenced using Big-dye terminator kit, ABI-GA3730 (V3.2,Applied Biosystems, USA). The bidirectional sequences were analyzed against standard sequences-blastn(https://blast.ncbi.nlm.nih.gov/Blast.cgi). Multiple sequence alignment was carried out for three sequences against VZV-Dumas prototype and structure prediction was done by Swiss model.

**Results:** The male-female ratio was 1:1; mean age of the participants was 17+ 15.7 (range from 3-59years). Among 56 samples and 10 controls tested for VZV ORF-68, 16.07%(n=9) were positive. None of our control group tested positive. Among nine tested positive, 33.3%(n=3) (vesicular fluid, CSF and colon biopsy) were sequenced and confirmed as Human herpesvirus-3. There was aminoacid substitution T40I in all three samples. Further confirmation was done by structure prediction using Swiss model.

**Conclusion:** VZV ORF-68 has a large non-conserved N-terminal end (aminoacid 1to188) which is required for replication of the virus. Mutation in this domain may lead to altered cell-to-cell spread, secondary envelopment and skin infection.

## ISSHID: Poster Presentations Abstract-6 Prevalence of syphilis among HIV infected people attending integrated counseling and testing center- a single center study

### Srirama BR, Mothi SN, Gururaja KS, Swamy VHT, Dayananda AK, Shyam S, Sagarika N

#### Ashakirana Charitable Trust Hospital, Mysore, India

**Background:** Despite the previous reports suggesting a decline in the prevalence of syphilis, majority of the recent reports from the developed nations including United States of America, Canada, Great Britain and Australia have observed an upsurge in its incidence. [3] A clear overview of the recent changes in the prevalence of syphilis is essential in targeting the efforts for future interventions to prevent HIV infection.

**Methods:** Cross sectional retrospective study. The study was done by chart review of registers of the ICTC attached to Ashakirana hospital from April 2012 to March 2015. People with Rapid plasma reagin (RPR) test reactive in titers 1/8 or above was diagnosed as having syphilis. Point prevalence of Syphilis in each year was calculated and comparison was done among the 3 years. Demographic factors were taken in percentage

**Results:** The point prevalence of Syphilis was 5.6% in 2012-13, 9.43% in 2013-14 and 10.2% in 2014-15. The sex distribution was 58.8% males and 41.2% females in 2012-13. 68% males and 32% females in 2013-14 and 68.57% males and 31.43% females in 2014-15. The age distribution was 18 – 40 years 64% , 41-60 years 35.29% and >60 years 0% in 2012-13, 18 – 40 years 52 % 41 -60 years 48% and >60 years 0% in 2013-14 and 18 – 40 years 42.85% 41 -60 years 54.28% and >60 years 2.85% in 2014-15.

**Conclusion:** We recommend that in addition to the initial testing, a routine test for syphilis to be established yearly in HIV infected population

## ISSHID Abstract-7 Incidence of Drug Resistance Mutations (DRMs) in HIV-1 vertically infected children attendees of Govt. General Hospital, Vijayawada, Andhra Pradesh, India

### N. Baratha Jyothi^1^, Sunita K^1^ , P Nitya Jeeva Prada^2^

#### ^1^Department of Zoology, Maris Stella College, Vijayawada-8; ^2^Department of Zoology, Acharya Nagarjuna University, Guntur, India

**Background:** More than 50 reverse transcriptase mutations are associated with nucleoside reverse transcriptase inhibitor resistance including M184V, thymidine analog mutations (TAMs), mutations associated with non-thymidine analog containing regimens, multi-nucleoside resistance mutations, and several recently identified accessory mutations.

**Methods:** The present article deals with the profile of HIV infected children and adolescents in HAART era and Pre-HAART era who were attending the ART centre, Govt. General Hospital, Vijayawada. The national programme will collect Dried Blood Spots (DBS) samples from all patients who are sent for HIV Drug resistance genotyping.

**Results:** Out of 66 subjects, tested for genotyping, 9 (12.1%) subjects have shown DRMs. For NRTIs, the most frequent mutation detected was K70KR in 5 patients; M184V observed in 4 patients; M184MV, D67N and D67DN in 3 patients; T69N and K219E in 2 patients; V751, M41L, Q151LM, T215F, V215I and T69G in 1 patient out of 11 NRTIs –exposed patients. For NNRTIs the most frequent mutations detected were K103N, G190AG and 498AG observed in 3 patients; V90I in 2 patients; P225H, Y188FHLY, K101EK, K103R, Y181C, Y181CY, V108I, V179D, K103KN and G190S in 1 patient out of 11 patients.

**Conclusion:** Our study reinforces the finding that, a less frequency of resistance mutations to ARV’s in subjects with virologic failure on HAART in the subjects. This supports the need for surveillance of resistance patterns to help guide therapeutic approaches, to limit transmission of these variants, and to guide the introduction of new drugs in HIV children.

## ISSHID Abstract-9 Increasing Chikungunya cases in Uttar Pradesh: Report of the first outbreak in a decade

### Ahmad Ozair, Danish N. Khan, Shantanu Prakash, Amit Bhagat, Anil Verma, Arjumand Faruqi, Suruchi Shukla, Amita Jain

#### Department of Microbiology, King George’s Medical University, Lucknow, India

**Background:** Chikungunya virus, a category-C NIAID pathogen, re-emerged in India after three decades in 2006. Fewer than a hundred laboratory-confirmed cases per million were reported in Uttar Pradesh (UP), the most populous Indian state with over 200 million inhabitants, in this upsurge and fewer so were reported annually up until 2011. We observed a sudden rise in cases of arthralgia with fever, classical of chikungunya, at UP’s nodal referral centre and sought to investigate demographical, clinical and serological trends of chikungunya affliction in UP.

**Methods:** We performed a retrospective analysis on all clinically-suspect chikungunya cases tested for anti-chikungunya IgM antibodies from October 2012 to December 2017 at King George’s Medical University, using ELISA-based kit.

**Results:** 23.7% of the 3240 cases included were seropositive. The greatly varied clinical presentations seen could be broadly divided into four groups with seropositivity being utmost in classic arthralgia patients (40%), trailed by those displaying fever of unknown origin (22%), encephalitis (13%) and fever with rash (12%). We saw a twenty-fold jump in number of cases (total;seropositive) in 2016 (1389;412) and 2017 (1619;341), compared to those in 2012-2015. Phylogenetic analysis of causative strain established that it was the same as the one causing concurrent outbreak in adjoining state of New Delhi.

**Conclusion:** Based on Central Bureau of Health Intelligence’s data from 2006-2011 and our own from 2012-2017, we find that chikungunya outbreak occurred in UP for the first time in a decade in 2016, that too as a part of large-scale upsurge across Northern India.

## ISSHID Abstract-15 Transfusion Transmissible HBV - Is blood safety compromised in developing countries?

### Krishnamoorthy Radhakrishnan, RavindraPrasad Thokala, Ashwin Anandan

#### Department of Transfusion Medicine, Sri Ramachandra Medical College & Research Institute, Sri Ramachandra Institute Of Higher Education and Research, Chennai, Tamilnadu, India

**Background:** Transfusion transmissible viruses cause infectious complications in recipients of blood transfusion. Screening of donated blood for HBsAg, antiHCV and Anti-HIV 1&2 are mandatory in India. Whereas Nucleic acid testing (NAT) and Core antibody testing (Anti-HBc) for Hepatitis B virus (HBV) infection are standard requirements of the American Association of Blood Banks (AABB), they are not mandated in developing countries like India; hence there exists a risk of transfusion transmissible HBV in developing countries. Anti-HBc serves as a surrogate marker for detecting occult Hepatitis B infections. This study was carried out with an objective of estimating prevalence of Anti-HBc among voluntary blood donors at our institution.

**Methods:** Informed consent was obtained from blood donors to screen their donated units for transfusion transmissible infections (TTI) as per National Blood Transfusion Council (NBTC) guidelines. Enzyme Linked Immunosorbent Assay (ELISA) for Anti-HBc (total) was carried out. Descriptive statistics was used to estimate prevalence (January 2018 to June 2019).

**Results:** During the study period, the prevalence of Anti-HBc was 6.90% and prevalence of HBsAg was 0.73% among voluntary blood donors; there was no correlation with age, gender, socio-economic status of blood donors.

**Conclusion:** Infectious disease testing carried out on blood donor samples should have 100% sensitivity since the blood bank is required to perform screening tests only. Mutant HBV strains pose a threat to the blood supply. NAT and Anti-HBc testing of donor samples will help achieve near zero risk of transfusion transmissible HBV albeit at the cost of shrinking blood donor pool.

## ISSHID Abstract-22 Cortical venous thrombosis due to herpes zoster – a rare case report

### Sureshkumar. K, Geetha.T, Ramesh.D

#### Coimbatore Medical College Hospital, Coimbatore, India

**Background:** The neurological complications of varicella zoster seen in less than 1% cases, which include cerebellitis,encephalitis and cerebral venous thrombosis are rarely reported. We present such a rare case of cortical venous thrombosis subsequent to varicella infection. The mechanisms underlying cerebral vascular events after VZV infection could be vasculitis, thrombosis due to direct endothelial damage and acquired protein S deficiency

Case report: 42years old male patient Farmer by occupation got admitted with history of headache over occipital region, vomiting for past 1 week and fever 2 weeks back. Multiple skin lesion over face and trunk for past 12 days. On examination Patient had multiple hyperpigmented scar lesions over face and trunk. Fundus examination shows both eye disc margins blurred and both eye established papilloedema .ESR –60 mm/hr. varicella IgM antibody positive. CT brain shows Thrombosis of superior saggital, right transverse and sigmoid sinuses extending into right internal jugular vein. MRI shows Thrombosis of superior saggital sinus, right transverse sinus, sigmoid sinus and bilateral frontoparietal cortical veins noted. He was treated with anti edema , anti epileptics, and anti coagulants The patient responded well and had an uneventful recovery. Consent was obtained from the patient for publication of their data

**Conclusion:** Cerebral venous sinus thrombosis is a rare neurological complication associated with primary varicella zoster infection. Early diagnosis and management can help prevent associated morbidity and mortality and lead to better outcome. The physician should have high index of suspicion for hypercoagulability in patients following varicella infection and institute appropriate therapy.

## ISSHID Abstract-26 Retrospective analysis of Co-Infection due to HIV/ HBV and HIV/HCV in cancer patients from regional cancer hospital in Ahmedabad, Gujarat

### Foram M Patel, Parijath N Goswami, Pankaj V Raval, Hiren A Patel

#### Department of Microbiology, The Gujarat Cancer & Research Institute, Ahmedabad, 380016 Gujarat, India

**Background:** There is very limited information available on the prevalence of co-infection of HIV/HBV and HIV/HCV. Hepatitis co-infection with HIV is associated with high morbidity and mortality. The objective of study was to assess the HBV and HCV prevalence in HIV reactive patients.

**Methods:** Retrospective analysis was done from June’13 to June’19. All the cancer patients were subjected for screening of HIV, HCV and HBV by ELISA(Qualisa –Tulip, India) on ELISA system (Euphoria,Tulip Group ). Pre & post counseling and written consent were taken as per NACO guidelines. All positive samples were tested twice according to the kit protocol. For HIV, reactive samples were tested with three different principle methods (ELISA, ICT and ELFA).

**Results:** During study period, total of 2, 07,179 samples were screened for HIV, HBsAg and HCV infection in cancer patients. HIV prevalence was 0.89%(1800/207179). HIV infection was more prevalent amongst male(64.22%) in 41-50 yrs. Surgical patients(48.28%) was having highest HIV prevalence having Head and Neck malignancy(28.33%). Amongst HIV reactive, co-infection rate of HIV/HBV was 2.17 %( 39/1800), HIV/HCV was 1.33 %( 24/1800) and One patients was having triple infection of HIV/HBV/HCV(0.06%). HIV/HBV(29/39) and HIV/HCV(14/24) were more found in male of 41-50 yrs. of age. HIV/HBV was more common in surgical patients(19/39) having gastrointestinal tumors(13/39), while HIV/HBV was prevalent in medical patients(14/24) having haematological malignancy(6/24).

**Conclusion:** HIV infection was more prevalent in male of middle age having solid tumors. Co-infection has more male preponderance during age of 41-50 yrs. HIV/HBV was mostly found in gastrointestinal tumors, while HIV/HCV in haematological malignancy.

## ISSHID Abstract-30 Role of hybrid chemokines in modulating CCR5 receptor function and HIV-1 entry inhibition

### A Deepu_1_, A Aravind^2^, K Santhosh Kumar^3^

#### ^1^Department of Botany, Mar Thoma College, Tiruvalla, India; ^2^College of Pharmaceutical Sciences, Thiruvananthapuram, India; ^3^Department of Pathogen Biology, Rajiv Gandhi Centre for Biotechnology, Thiruvananthapuram, India

**Background:** The CC chemokine receptor 5 (CCR5) plays a very pivotal role in facilitating the entry of human immunodeficiency virus-1 (HIV-1) into host cells. Ligands interacting with CCR5 could thus function both as effective HIV-1 entry inhibitors and as valuable tools for studying receptor biology. Use of natural CCR5 ligands for therapeutic purposes has often been hampered by the potential side effects due to the high degree of promiscuity evident in chemokine system. In the present study we designed a hybrid chemokine by attaching the N terminus of the most potent agonist of CCR5 to the core region of the viral macrophage inflammatory protein-II to obtain a ligand with high affinity for CCR5.

**Methods:** The hybrid chemokine was synthesized by Fmoc solid phase peptide synthesis and structural characterization was done using CD spectroscopy. The Interaction of hybrid chemokine with CCR5 was analyzed by calcium efflux studies, chemotaxis assay and receptor internalization studies. Antiviral potency was evaluated by p24 antigen assay.

**Results:** The hybrid chemokine displayed similar structural pattern to natural chemokines in CD spectra and elicited chemotactic activity, induced calcium efflux and caused receptor internalization in CCR5 positive cells. It exhibited anti HIV-1 activity by reducing the production of p24 antigen in cell culture system infected with virus.

**Conclusion:** The outcome of this study encourages the pursuance of the concept of hybrid chemokines both as a valuable therapeutic tool in preventing the cellular entry of HIV-1 and as a viable option to decipher the nuances of chemokine receptor biology.

## ISSHID Abstract-31 Functional characterization of streptomycin adenylyltransferase from *Serratia marcescens*: An experimental approach to understand the Antibiotic Resistance mechanism

### Dhamodharan Prabhu^1^, Mathimaran Amala^1^, Poopandi Saritha^1^, Sundaraj Rajamanikandan^2^, Malaisamy Veerapandiyan^1^, Jeyaraman Jeyakanthan^1^

#### ^1^Structural Biology and Biocomputing Lab, Department of Bioinformatics, Alagappa University, Science Campus, Karaikudi – 630004, Tamil Nadu, India; ^2^Complex Systems Division, Beijing Computational Science Research Center, ZPark II, Haidian, Beijing 100193, People’s Republic of China

**Background:***Serratia marcescens*, a gram negative opportunistic pathogen, is the main causative agent for the acquired nosocomial infections in the intensive-care units of hospital environments. Intrinsically, it posses several antibiotic resistance mechanisms to counter attack the antibiotics for the survival. The emerging new resistance mechanisms threaten the entire world and impeding the treatment procedures. Controlling antibiotic resistance is mandatory and therefore we investigated the hypothetical protein for its potential role in fighting against antibiotic resistance.

**Methods:** The target gene cloned into pET28a+ expression vector was transformed into E. coli BL21(DE3)pLysS cells; further purified using Ni-NTA affinity and Size Exclusion Chromatography. Minimum Inhibitory Concentrations of different aminoglycoside antibiotics were determined using the Ezy MIC™ Strips. The mechanism of the protein was characterized by Chromatographic adenylation assay and Malachite Green assay.

**Results:** The purified protein was confirmed by western blotting, MALDI-TOF analysis and identified as aminoglycoside nucleotidyltransferases protein. The protein expressed in E. coli cells has shown resistance to streptomycin (>256 MCG) and sensitive to ampicillin, amikacin, erythromycin, gentamycin, kanamycin and tobramycin. The enzyme catalyzed adenylated-streptomycin was absorbed at 260 nm wavelength. The adenylation process of streptomycin was detected by capturing the release of pyrophosphate from ATP and absorbance measured at 620 nm.

**Conclusion:** The functional assays confirmed the hypothetical protein is Streptomycin adenylyltransferase, which utilizes the ATP to modify the streptomycin by adenylation process. Mechanistic information of the protein provides deeper knowledge for designing better inhibitors to overcome antibiotic resistance.

## ISSHID Abstract-33 A nonlinear autoregressive exogenous model for patient specific modeling of the CD4 cell count and HIV-1 viral load interaction dynamics

### V. Rajinikanth1, K. Kamalanand2, B. Thayumanavan3 and P. Mannar Jawahar4

#### ^1^Department of Electronics and Instrumentation Engineering., St. Joseph’s College of Engineering, Chennai 600 119, India; ^2^Department of Instrumentation Engineering, MIT Campus, Anna University, Chennai 600044, India; ^3^Department of Oral Pathology, Sathyabama University Dental College and Hospital, Chennai 600119, India; ^4^Karunya Institute of Technology and Sciences, Coimbatore 641 114, India

**Background:** Mathematical modeling of HIV-1 infection dynamics is an important task since the developed model provides valuable information on the progression of the infection as well as the drug dosage required for an optimal planning of ART therapy. Further, the identification of the mathematical relationship between the CD4 cell count and HIV-1 viral load can provide an useful means for patient specific estimation of HIV-1 viral load in resource limited settings.

**Methods:** In this work, a patient specific mathematical model based on the nonlinear autoregressive exogenous model (NARX) structure was developed with CD4 cell count as the input variable and HIV-1 viral load as the output variable. The nonlinearity utilized in the model is a tree partition with 15 units. Further, the Gauss-Newton technique was utilized for the identification of model parameters.

**Results:** The developed model structure was found to be efficient in modeling the patient specific interaction between the CD4 cell count and the HIV-1 viral load. The estimated Mean Squared Error (MSE) in the developed model was very low with a value of 5.312e-07. Further, the accuracy of the developed model was 95.5%.

**Conclusion:** Since the developed NARX model is dynamic and time dependent, it can be utilized to study the dynamics of the interaction between the CD4 cell count and HIV-1 viral load. Further, the model can also be used to estimate the HIV-1 viral load from CD4 cell count measurements.

## ISSHID Abstract-39 Prevalence of Hepatitis C Viral Infection among Beta-Thalassemia Patients: A Study from the Centre for Excellence in Thalassemia and other Blood Disorders

### Sravanireddy Vinjamuri^1^, Pravallika Taniklla^2^, Venkataramana Kandi^3^

#### ^1^II MBBS students, Prathima Institute of Medical Sciences, Karimnagar, Telangana, India; ^2^II MBBS students, Prathima Institute of Medical Sciences, Karimnagar, Telangana, India; ^3^Associate Professor, Department of Microbioogy, Prathima Institute of Medical Sciences, Karimnagar, Telangana, India

**Background:** Hepatitis C virus (HCV) is a single-stranded RNA virus, which is frequently transmitted through blood transfusions, contact with infected blood or blood products, and vertical transmission. Injectable drug abusers and transplant recipients are predisposed to HCV infection. It causes acute hepatitis, which may progress to chronic hepatitis and in severe untreated cases, patients may develop cirrhosis and hepatocellular carcinoma. Since there is no vaccine available against HCV infection, prevention remains the mainstay, at least among the susceptible population.

**Methods:** A case-control study was conducted at the center for excellence in thalassemia and other blood disorders attached to the Prathima institute of medical sciences, tertiary care teaching hospital at Karimnagar, Telangana, India. Blood samples of 100 beta-thalassemia patients and 100 age-matched controls were screened for antibodies against HCV by an Enzyme-linked immunosorbent assay (ELISA), a rapid immunochromatographic method, and the chemiluminescence Assay using the Abbot.

**Results:** Of the 100 cases of beta-thalassemia 28 (28%) were HCV positive and all the controls were negative for HCV infection. The prevalence among the patients attending the hospital was found to mere 0.19%.

**Conclusion:** HCV infection among the beta-thalassemia patients was abnormally high as compared to the control group. Thalassemia patients were predisposed to HCV infection and other blood-borne viral infections. Thorough screening of blood prior to transfusion is warranted. HCV infection may further increase the morbidity and mortality of beta-thalassemia patients and other patients with blood disorders who acquire the infection due to frequent blood transfusions.

## ISSHID Abstract-40 Physagulin-F Inhibits HIV-1 Replication through Reverse Transcriptase Inhibition

### Estari Mamidala, Swapna Gurrapu

#### Infectious Diseases Research Lab, Department of Zoology, Kakatiya University, Warangal-506009, (Telangana State), India

**Background:** People living with HIV/AIDS ofen choose traditional or complementary and alternative medicine to complement or replace conventional treatment. The objective of the current study is to isolate HIV-1 reverse transcriptase inhibitor from Physalis angulata fruits.

**Methods:** The crude extracts were prepared from dried areal parts P. angulata with using non-polar to polar solvents by maceration method and isolated Physagulin-F by using column chromatography and HPLC. Peripheral Blood Mononuclear Cells (PBMCs) isolated from healthy donors by ficoll–hypaque density gradient centrifugation method. Cell viability test was performed on Physagulin-F by MTT assay using PBMC and HIV-1 RT inhibition activity was determined by HIV-1 Reverse Transcriptase (p66) Capture ELISA.

**Results:** In the HIV-1 reverse transcriptase assay, isolated compound pysagulin-F showed good inhibitory activity (84%) at 800 μg/ml and MTT assay confirmed that the isolated Physagulin-F had no cytotoxic activity and IC50 values higher than 200 μg/ml.

**Conclusion:** The physagulin of Physalis plant has great potential for developing useful drugs. Extraction of important biologically-active phytochemicals from this plant will certainly be helpful in protecting and treating various viral diseases in human beings.

## ISSHID Abstract-41 A surprise caller visiting a rare location

### Archana.B^1^, S.Rajendiran^1^, N.Palaniappan^2^

#### ^1^Department of Pathology, SRIHER, Porur, Chennai; ^2^Department of Obstetrics & Gynecology, SRIHER, Porur, Chennai

**Background:** Cytomegalovirus (CMV) is a member of Herpesviridae, a family of enveloped, double-stranded DNA viruses having complex genomes.CMV infection is usually asymptomatic in immunocompetent individuals but is capable of resurfacing during periods of decreased immunity. In immunocompromised patients, CMV infection can cause life-threatening consequences.Females, though by a small margin, are at a higher risk of CMV infection than males.While CMV infection is known to cause abortions in pregnant women, it is very rare and uncommon in the female genital tract (FGT).

**Case report:** A 50year old diabetic patient presented to the gynecology department with abdominal pain since 5 months.Ultrasound of the abdomen revealed a fibroid uterus. Vaginal hysterectomy with bilateral salpingectomy was performed. Grossly, the specimen showed a leiomyoma; however, rest of the uterus, cervix and tubes looked absolutely unremarkable. Surprisingly, on microscopy, cervix, endometrium, myometrium and tubes showed dense acute on chronic inflammation studded with numerous organisms with owl’s eye inclusions resembling CMV in the epithelial, endothelial and mesenchymal cells. Immunohistochemistry for CMV confirmed these organisms.Serology also revealed that the patient was HIV positive.

**Conclusion:** Though infection with CMV has been reported elsewhere like the colon, retina and oesophagus; few studies have reported CMV primarily in the FGT. In immunocompromised patients, infection with CMV has to be considered as it may result in significant morbidity and may be associated with pelvic inflammatory disease and cervical dysplasia. Recognition of CMV may help clinicians institute appropriate optimum management, follow up the patient and screen the family members too.

## ISSHID Abstract-42 Development of Machine-learning tool to classify Plasmodium species based on its shape and texture features

### N. Sri Madhava Raja^1^, S. Arunmozhi^2^, Inthumathy Jayabalan^3^ and B. Ajay Rajasekaran^4^

#### ^1^Department of Electronics and Instrumentation Engineering., St. Joseph’s College of Engineering, Chennai 600 119, India; ^2^Department of Electronics and Communication Engineering, ManakulaVinayagar Institute of Technology, Puducherry, India; ^3^Birchwood Surgery, Letchworth Garden City, Hertfordshire, UK; ^4^Newcastle University Medicine Malaysia, 79200 GelangPatah, Johor, Malaysia

**Background:** In malaria diagnosis, the identification of parasite (plasmodium) plays a vital role in treatment planning. The drug selection and dose level also varies based on the type of the species and its infection level. The proposed work aims to implement a Machine-Learning Technique (MLT) for evaluation and classification of the plasmodium recorded using a digital microscope with the thin blood smear.

**Methods:** This work proposes a MLT to process and classify the microscopic pictures collected from malaria infected patients. A series of techniques, such as image-enhancement, segmentation, texture/shape feature extraction, dominant feature selection and classification are implemented. A morphology based segmentation of plasmodium is implemented in initial phase. Later, the shape and texture features are extracted using feature mining technique. Further, feature selection is applied to identify the dominant feature set. Finally a Multi-Class-Classifier (MCC) based on Neural-Network is implemented to classify the pictures.

**Results:** The developed system was found to be efficient in classifying the species based on its texture and shape measures. The obtained output provides an overall classification accuracy of 96.83%. Further, a relative assessment with the similar technique in the literature confirms that, proposed technique offers better precision, sensitivity and specificity.

**Conclusion:** The proposed MLT is efficient and works well on microscopic image database. In future, it can be considered to examine the clinical level blood smear images. Further, the developed method can be used as an assisting tool for the doctors during the mass screening process; which will help to minimize the diagnosis burden.

## ISSHID Abstract-51 Epidemiological analysis of pandemic influenza A (H1N1) pdm09 in Central India- 2019

### Senthil Raja^1,2^, Deepa Sankari^2^, K.N.Brahmadathan^2^, C.P.Thangavelu^2^, M.Mani^2^

#### ^1^Microbiological Laboratory, First floor Yashwant Plaza, Opposite Indore Railway Station Indore, Madhya Pradesh 452001; ^2^Microbiological Laboratory, 12A Cowley Brown Road, R.S.Puram, Coimbatore - 641002

**Background:** Influenza A viruses cause continuing local or global outbreaks with severe consequences on human health and the global economy. The novel strain of influenza A virus – H1N1 2009 had caused pandemic disease among human probably owing to little or no preexisting immunity to the new strain. This is a retrospective analysis of the impact of H1N12009 on the population of Central India during the pandemic period of Feb- 2019- April 2019.

**Methods:** Influenza A assay set primers and probes were used for the detection of H1N1 by RotorGene Q® Real time RT PCR during the period, Feb- 2019- April 2019. Positive H1N1 samples were sequenced and submitted in NCBI for genetic characterization.

**Results:** A total of 667 samples were tested. Out of which 249 (37.33 %) were positive for H1N1 pdm09. Of the 249 positives, 9 (1.35 %) were pediatric ( 55 years. In Central India, western Madhya Pradesh was the worst affected. The samples were collected from cities such as Indore (71.36%), Ujjain (12.44%), Dewas (4.95%), Dhar (4.65%), Ratlam (1.8%), Raisen (1.65%), Harda (0.9%) Sehore (0.75%), Chhatarpur (0.45%), Shajapur (0.3%), Bhopal (0.3%), Mandla (0.3%), Sagar (0.15%) and overall positivity in Indore were observed as (26.39%), Ujjain (5.85%), Dewas (1.95%), Dhar (1.35%), Ratlam (1.2%), Raisen (0.45%), Sehore (0.45%), & Shajapur (0.15%). A total of 53 patients died of which 56.6% were male.

**Conclusion:** Laboratories should be equipped with facilities and expertise for identification of H1N1 at all times.

## ISSHID Abstract-52 A study on the distribution of Human Immunodeciency Virus (HIV) and Syphilis infection and associated risk factors among the Irula Tribal Community of Tamil Nadu State, India

### Elangovan Ramya^1, 4^, Joseph C. Daniel^2^, P.V.Geetha^3^, R.Usha^1^, Natesan Manikandan^4^, P.Kamaraj^4^, J.Nagaraj^4^, C.P.Venil^5^

#### ^1^Department of Microbiology, Karpagam Academy of Higher Education, Coimbatore, Tamil Nadu, India; ^2^Department of Microbiology, St.Josephs College of Arts and Science, Cuddalore, Tamil Nadu, India; ^3^Department of Microbiology, Sri Ramachandra Institute of Higher Education and Research, Chennai, Tamil Nadu, India; ^4^ICMR-National Institute of Epidemiology, Chennai, Tamil Nadu, India; ^5^Department of Biotechnology, Anna University Regional Campus-Coimbatore, Coimbatore, Tamil Nadu, India

**Background:** An extensive study on the distribution of Human Immunodeficiency Virus and Syphilis infection among the Irula tribal community from Cuddalore, Kanchipuram, Thiruvallur and Villupuram Districts of Tamil Nadu.

**Methods:** Medical camps were organized for the Irula tribal population. A pretested proforma was used to obtain the possible behavioral risk factors. 372 blood samples were collected non-randomly (above18 years). All the samples were tested for antibodies to HIV by ELISA and also tested for antibodies to *Treponema pallidum* by qualitative RPR.

**Results**: Seropositivity of HIV was 1.34% and Syphilis was 1.08%. Of the study samples, 20% had dual HIV and Syphilis Co-infection. In this, males had a much higher prevalence of both infections than females (HIV: 2.54% Vs. 0.79%, Syphilis: 2.54% Vs. 0.39%, Co-infected: 0.84% Vs. 0.00%). The presence of HIV antibodies was significantly associated with Alcoholism (P=0.028), Intravenous drug abuse (adjusted odds ratio [OR] =5.95; 95% Confidence Interval [CI] =0.96-36.74), Series of Injection (OR=5.74; 95% CI=0.94-34.99) and Sexual behaviour (OR=10.12; 95% CI =1.12-91.57). Syphilis infection was most significantly associated with Jaundice in the family (P=0.002), Alcoholism (P=0.05) and independently significant association with Sexual Behaviour (OR=7.51; 95% CI=0.77-73.06).

**Conclusion:** The distribution of HIV and Syphilis infection was observed among the Irula tribal population with various risk factors such as sexual behavior, alcoholism, intravenous drug abuse and series of injections. In addition, we found that History of jaundice was placed a major role in the transmission of syphilis infection.

## ISSHID Abstract-53 Fatal ameobic meningo-enchephalitis in immunocompetent host in resource limited setting in India

### Sangmesh Chavanda^1^, Manisha Gupta^2^, Devashish Ruikar^1^, Ghansham Darak^1^, Umesh Kanade^3^

#### ^1^Department of Medicine, Aashirwad Criti Care, Latur, Maharastra, India; ^2^SGPGI, Lucknow, Uttar Pradesh, India; ^3^Deparment of Pathology, GMC, Latur, Maharastra, India

**Background:** We present a rare case of *N.fowleri* living amoeba meningitis in immunocompetent person

**Methods:** 54 years male immunocompetent who used soiled expired eye drops for symptomatic relief for itching which was prescribed by some general practitioner long back.patient had fulminant course of disease expired within almost 4 days of onset of disease

**Results:** Diagnosis was made on basis of clinical signs of meningism; CSF examination revealed neutrophilic predominance meningitis with simple wet mount light microscopy showed amoebic motility, pcr studies was not possible due to resource limited setting.

**Conclusion:** While *N. fowleri* infection appears to be quite rare compared to other diseases, the clinical manifestations of primary amoebic meningoencephalitis are devastating and nearly always fatal. Due to the rarity of *N. fowleri* infections in humans, there are no clinical trials to date that assess the efficacy of one treatment regimen over another. Most of the information regarding medication efficacy is based on either case reports or in vitro studies. Secondly simple test of wet mount in light microscopy has diagnostic values in resource limited settings.

## ISSHID Abstract-57 Plasma TNF-α is associated with decrease of blood platelet levels in dengue infection

### Jaisheela Vimali Irudhayaraj^1^, Amudhan Murugesan^2^, Esaki M. Shankar^1^

#### ^1^Division of Infectiology and Medical Microbiology, Department of Life Sciences, School of Life Sciences, Central University of Tamil Nadu, Thiruvarur, Tamilnadu, India; ^2^Department of Microbiology, Government Theni Medical College & Hospital, Theni, Tamilnadu, India

**Background:** Dengue virus infection is a vector-borne disease of tropical and sub-tropical region with protean manifestations affecting ~50 million people globally. Clinical manifestations vary from dengue fever, dengue haemorrhagic fever (DHF) and dengue shock syndrome in which exhaustion of T-cells accounts for viral persistence and disease severity. Dengue virus infection results in increased production of cytokines by infected monocytes, B lymphocytes and mast cells. CD4+ and CD8+ T-cells are likely targeted by DENV, particularly during secondary infection when serotypes cross-react with bystander CD4+ and CD8+ T cells. Cross-reactive memory T-cells play an important role in the pathogenesis of secondary dengue (SD) by altering the cytokine profiles. In the present study we have assessed the clinical and cytokine profile in primary and secondary dengue cases.

**Methods:** Serum samples from patients of Govt. Theni Medical college was collected and tested for plasma levels of TNF-α, IFN-γ, GM-CSF, IL-13, IL-12p70, IL-10, IL-5, IL-4 and IL-2 cytokines in primary and secondary dengue infection and correlated with the levels of blood platelet. All statistical analyses were done using GraphPad Prism 6 software (La Jolla, California, USA).

**Results:** Assessment of cytokines and their correlation with disease revealed that four cytokines IL-2, TNF-α, IL-4, IL-10 were significantly elevated in secondary dengue infection whereas IL-13 remained unaltered during primary and secondary infection. Decreased blood platelet levels were associated with increase in the serum concentrations of TNF-α among DENV-infected individuals.

**Conclusion:** TNF- α could play a pivotal role in enhancing dengue pathogenesis inspite of decreased platelet count."

## ISSHID Abstract-58 A Rare case of Allergic Fungal Rhinosinusitis due to *Myriodontium keratinophilum*

### Yamini Anandan, Anupma Jyoti Kindo

#### Department of Microbiology, Sri Ramachandra Institute of Higher Education and Research, Chennai, India

**Background:** To report a rare case of allergic fungal rhinosinusitis due to *Myriodontium keratinophilum*, which has not been previously reported.

**Methods:** A 41 year old female patient presented with history of nasal block and nasal discharge, following which she developed a pale glistening polypoidal mass in the left nasal cavity. The polypoidal mass with fungal debris was removed by functional endoscopic sinus surgery under general anaesthesia and sent for fungal culture, smear such as gram stain, KOH mount, lacto-phenol cotton-blue mount, Sabouraud Dextrose Agar to identify the type of growth, to able to diagnose the agent causing the fungal rhinosinusitis.

**Results:** Culture on SDA revealed the growth of white mould at 25 degree Celsius incubation. Lacto-phenol cotton-blue mount from the polypoidal mass and fungal debris revealed fungal hyphae with non-dichotomous branching and produced a single conidia on denticles along the side of the hyphae. Slide culture was put up to identify the fungus, it revealed conidial arrangement consistent with that of Myriodontium Keratinophilum species. Consent has been obtained from the patient for the publication of this case study.

**Conclusion:** This case report lays emphasis on a thorough microbiological work up, to provide insightful information, thereby expanding our knowledge and spawning new research organisms which may not have been reported earlier. We hereby report a culture proven case of allergic fungal rhinosinusitis due to a very rare species, Myriodontium keratinophilum.

## ISSHID Abstract-59 Outbreak of Mumps in suburban Chennai- a report

### Quartzita Melofer M

#### Department of Community Medicine, SRM Medical College Hospital and Research Centre, Kattankulathur, Kancheepuram District, Tamilnadu, South India

**Background:** An outbreak of Mumps was recorded in Kattankulathur, located in sub urban region of Chennai between February 2019 and April 2019. The index case was a 6 year old male child who presented with bilateral parotitis in a group home for children.

**Methods:** 54 Patients of all age groups and both genders attending SRM Urban Health and Training Centre dispensary, Kattankulathur between February and April 2019 with clinical signs and symptoms of mumps in accordance with standard definition were included for the study.

**Results:** 52 children were affected, 44 hostilities, 10 day scholars and 2 adults. 32 were boys and 20 were girls, 2 women. Age distribution was between 2-53 years, mean age being 10 years. Immunization status of 16.4 % (9 orphan children) was unknown, of the remaining, 83.6 % (n= 46) were not vaccinated for Mumps completely, 4 children had received one dose of MMR vaccine at 18 months. 3 boys had complication of Otitis Media and Orchitis. Sleeping in close contact dormitory, common lunch hall and communal bathing unit were contributors to the spread of the disease. The children were isolated in the hospital on campus and the regional health centre was notified and surveillance of the surrounding area was started. Follow up after 2 months was uneventful.

**Conclusion:** This report highlights the need for routine vaccination against mumps in early childhood to prevent the shift of disease pattern to adult population where there is higher risk of complications.

## ISSHID Abstract-62 Chikungunya induced Rheumatoid arthritis via vimentin citrullination and PADI4 susceptibility in South Indian Tamil RA population

### V Malini, N Shettu

#### P.G. and Research Department of Zoology, Pachaiyappa's College, Chennai, Tamil Nadu, India

**Background:** Chikungunya virus (CHIKV) infection is a known risk factor for Rheumatoid Arthritis (RA), nonetheless, the precise mechanism is still elusive. Citrullination is a cornerstone of RA and PADI4 genetic variants are consistently proved to be a prominent RA risk allele among Asians. Symptom similarities between RA and CHIKV arthritis was intriguing to investigate whether the basic biochemical events and genetic susceptibility of RA could also be a mediator of CHIKV induced RA.

**Methods:** Blood samples and CHIKV history collected from 207 RA cases satisfying 2010 ACR criteria visiting rheumatology clinic and 186 age, sex & ethnicity matched controls in Chennai, India with informed consent. RF, anti-CCP, anti-Sa, anti-CEP-1, CRP, ESR and DAS28 were analyzed. All cases and controls genotyped for PADI4_92 (rs874881), PADI4_104 (rs1748033) and PADI4_94 (rs2240340) by Sanger's direct sequencing. CHIKV infected RA cases and CHIKV infected controls were compared.

**Results:** Alarmingly, out of 95 CHIKV infected RA, 76 (80%) developed RA within a year following infection. CHIKV infected RA cases illustrated significantly higher frequency (p=0.01) and anti-Sa level (p=0.04) than 112 non-infected RA cases with trivial difference in other parameters. All studied polymorphisms demonstrated to be a susceptible allele for CHIKV infected RA (p <0.05).

**Conclusion :** This is the first report that CHIKV may contribute to RA development via citrullination of vimentin. Further, PADI4 poses risk loci for the initiation and exacerbation of CHIKV induced RA in Tamil population. This finding offers a prototype for future studies aiming to disentangle RA complexity for developing targeted therapy

## ISSHID Abstract-70 Involvement of microRNA in regulation of pro-inflammatory genes in HIV1 TAT activated astrocytes

### Kamini Khatak, Durairaj D, Nivedita Chatterjee

#### L&T Department of Ocular Pathology, Vision Research Foundation, Chennai, India

**Background:** Glial cells in the Central Nervous System (CNS) are responsible for immune response and inflammation triggered by stress factors and pathogens. MicroRNAs are short non coding RNA involved in the post transcriptional process. miRNA networks play critical role in many physiological conditions and can change disease conditions. Here we look at global miRNA profile in human primary astrocytes on being activated with recombinant HIV1 TAT protein. We also test whether endogenous anti-inflammatory agents, the endocannabinoid AEA affect miRNA profile in astrocytes.

**Methods:** Primary human astrocytes were cultured and treated with HIV1 TAT protein and AEA. Subsequent analysis was done by Real Time PCR, miRNA global microarray, DCFDA, and appropriate antagomirs/ mimics.

**Results:** Global microRNA profiling in HIV1 TAT activated astrocytes showed significant changes in many miRNAs. Implicated pathways included AKT1, MAPK and Hippo. miR- 192-5p was suppressed in a dose dependent manner but not miR-543, in activated cells. Endocannabinoid AEA reverses these changes for both miRNAs. HIV1 TAT treatment increases the expression of inflammation genes, IL-18, IL-1β, ASC and NLRP3. Using antagomirs/mimics of miR192 and miR 543, we show that these regulate only specific inflammatory components.

**Conclusion:** HIV1 TAT, a coat protein shed by HIV increases the inflammatory cascade. miRNA study in viral infection can give better understanding about the regulation in disease progression. Role of miRNA in activated astrocytes can act as many targets for therapeutic benefit in controlling disease progression.

## ISSHID Abstract-71 Quantitative proteomic analysis of an AmBisome resistant *Leishmania donovani* strain reveals multiple metabolic and signalling processes affected in drug resistance

### Rimi Mukherjee, Ashish Kumar, Tanvir Bamra, Taj Shafi, Manjay Kumar, Kavita Bharati, Abhik Sen, Pradeep Das

#### Department of Molecular Biology, RMRI, Patna, India

**Background:** Background: Leishmaniasis is a neglected tropical disease, caused by the intracellular parasite Leishmania. Visceral Leishmaniasis caused by *Leishmania donovani*, is the most serious systemic form that can be fatal if untreated. The current drug of choice is single dose AmBisome. The treatment efficacy of VL is being increasingly challenged by drug resistance. Therefore, the aim of this study was to compare the proteomic profiles in clinical isolates of AmBisome resistant and sensitive L. donovani strains, to identify potential markers of resistance.

**Methods:** Resistant and sensitive *L. donovani* clinical isolates were obtained from parasite bank of RMRI. These parasites were confirmed by PCR and characterized by drug sensitivity test. Total protein from the parasites was quantified for their identity by liquid chromatography and mass spectrometry.

**Results:** Total of 669 (26.7%) out of 2503 proteins was found to be differentially expressed in the comparative LC-electrospray ionization MS/MS analysis (12.9% up-regulated; 13.8% down-regulated in resistant strain). Functional grouping of the differentially expressed proteins depicted the following: - Energy metabolism (9.9% Up-regulated, 6.9% Down-regulated); Protein folding & degradation (3.7% Up-regulated, 2.5% Down-regulated); Cytoskeleton & Cell membrane (10% Up-regulated, 10.8% Down-regulated); Redox signalling (1.5% Up-regulated, 0% Down-regulated); RNA/DNA metabolism (15% Up-regulated, 9.8% Down-regulated). The detailed functional analysis is underway.

**Conclusion:** Analysis of the proteome data revealed certain groups of proteins whose expression was highly altered in the resistant strain like those grouped under DNA/RNA metabolism, cell cytoskeleton, redox signalling and energy metabolism, which likely play an important role in conferring drug resistance to the parasites.

## ISSHID Abstract-72 Molecular typing of *S. enterica Ser.typhi* isolates from South India

### Valinderjeet Randhawa, Ravinder Kaur, Meenakshi Singh

#### Dept. of Microbiology, Lady Hardinge Medical College, New Delhi

**Background:** The current study was to evaluate Pulsed field gel electrophoresis(PFGE) as a typing tool for S. enterica Ser. Typhi isolates from South India. Till recently dependence was on phenotypic based techniques as phage typing to characterize isolates involved in outbreaks.

**Methods:** The PFGE was done by PulseNet protocol CDC,Atlanta. The interpretation of banding pattern was done by method of Tenover .The reproducibility of the technique was confirmed.The isolates were identified with a capital letter X followed by a numeral.These isolates were also phage typed.

**Results:** Four hundred and thirty three isolates collected from nine collaborating centers were subjected to testing. The band number varied between 9-17, with sizes in the range of 22Kb to 650Kb .Thirty five different profiles were obtained which belonged to 10 different groups. The Discriminatory index of the technique was 0.97 and typeability was 100%.X15, X17 and X25 were the predominant types. The discriminatory index of phage typing was 0.25 and the predominant phage type was E1.

**Conclusion:** PFGE is an excellent typing system with high typeability, reproducibility and discriminatory power. It is more discriminatory than the conventional phage typing and can be used to delineate the isolates from the outbreaks.

## ISSHID Abstract-73 *Pythium insidiosum* corneal ulcer - a case report

### K Vishnu Priya^1^, S Thilagavathy^2^, R Varun Prasanna^3^

#### ^1^Department of Microbiology, Dhanalakshmi Srinivasan Medical College and Hopital, Perambalur, Tamil Nadu, India; ^2^Department of Microbiology, Kauvery Hospital, Tiruchirapalli, Tamil Nadu, India; ^3^Department of Medicine, Kauvery Hospital, Tiruchirapalli, Tamil Nadu, India

**Background:***Pythium insidiosum*, an emerging pseudofungi of aquatic origin, inhabits moist soil environment such as irrigation channels, rice paddies etc. It is an oomycete that causes pythiosis insidiosi, an uncommon life threatening disease. Various clinical forms of human pythiosis are - vascular, ocular, cutaneous or subcutaneous and disseminated forms. We present a case of ocular pythiosis identified by gene sequencing.

**Case Report:** A 62 year old male farmer was admitted with complaints of right eye pain with pus discharge and breathlessness. He is a known case of leprosy, diabetes mellitus and hypertension. Ophthalmologist opinion was sought and diagnosed as panophthalmitis with corneal abscess for which the patient underwent right evisceration and orbital implant was placed. The intraocular contents were sent to microbiology lab. The patient was started with IV. Liposmal amphotericin B, Atropine eye drops and Natamycin eye drops empirically. Gram stain showed moderate epithelial cells, occasional pus cells and no bacteria. KOH wet mount showed sparsely septate broadly branching hyphae. Culture on Sabouraud Dextrose Agar showed light brown coloured fungal colony with no conidia. Lactophenol Cotton Blue Mount showed sparsely septate, broadly branching hyphae. The isolate was identified as *Pythium insidiosum* by gene sequencing. Consent has been taken from the next of kin of the patient for publication of this data as the patient had expired.

**Conclusion:** Human pythiosis is often misdiagnosed as it creates confusion with zygomycosis entailing improper and delayed specific treatment leading to fatal consequences. Hence, prompt identification is necessary as most isolates of pythium are ignored as lab contaminants.

## ISSHID Abstract-74 Morphological spectrum of Histoplasma in HIV and non-HIV patient- Report of two cases

### Abinaya Devi, Jayalakshmy, LillykuttyPothen, Sankar

#### Department of Pathology, GMC, Kottayam, Kerala, India

**Background:** Histoplasmosis is a fungal infection caused by *Histoplasma capsulatum* and is acquired by inhalation of dust particles from soil contaminated with bird or bad droppings. Disseminated histoplasmosis can develop in 4.2% of immunocompetent and 55.2% of immunosuppressed persons. We present two cases of histoplasmosis in a HIV and Non-HIV patient with a view to describe and differentiate the tissue reactions between the two.

**Case presentation:** Case1: A 33year old retro-positive male, presented with multiple ulcerations, erythematous papule, scaling and crusting over scalp, arm, forearm and lowerlimb. Histopathology showed dermis with dense inflammation composed of sheets of histiocytes, a few lymphocytes, plasma cells and neutrophils. No definite granuloma identified. Fungal spores with a halo are noted inside the histiocytes.

**Case2:** A 49 year old immunocompetent male, presented with ulceroproliferative lesion involving posterior part of the right alveolar ridge. He also had a hyperpigmented papule with central erosion over right nasal alae and an ulcer over the right retromolar trigone and bilateral vocal cords. Histopathology of these lesions showed chronic suppuration and well-defined granuloma composed of histiocytes and giant cells. Many intracellular and extra cellular fungal spores with halo are seen.

GMS was positive for fungal spores in both cases and diagnosis of histoplasmosis was suggested. Both the patients have given consent for the publication of their personal data

**Conclusion:** The histological reaction varies according to the severity, phase of the infection and the host's immune system. The organisms were seen both intra-cellularly and extra-cellularly with granuloma formation in immune-competent individual, whereas the organisms were seen only intra-cellularly with no granuloma in retropositive individual.

## ISSHID Abstract-75 Analysis of HIV-1 Integrase gene by applying an easy cost-effective in-house assay using plasma and Dried Blood Spot (DBS) specimens

### Sannoong Hu, Ajit Patil, Devidas Chaturbhuj, Swarali Kurle

#### HIV Drug Resistance Laboratory, National AIDS Research Institute, Pune, 411026, India

**Background:** The National AIDS Control Program of India has approved use of Integrase strand transfer inhibitors (INSTIs) as third line regimen. Resistance mutations have been identified for INSTIs. Easy and cost-effective methods are needed for integrase resistance analysis. Data on Integrase resistance in India is limited. This urges research into INSTI drug resistance genotyping, prevalence and mechanisms.

**Methods:** An in-house RT-Nested PCR protocol was standardized for multiple HIV-1 subtypes. Integrase gene was amplified and sequenced from 20 plasma and Dried Blood Spots (DBS) specimens from different HIV-1 subtypes (including B, C, B/D, A/E and F). The sequence data was analyzed using various tools like MEGA, REGA, JPHMM and HIVDRDB program for subtype analysis and interpretation of resistance mutations.

**Results:** The standardized in-house assay could successfully amplify and sequence integrase gene from both plasma and DBS specimens. Integrase sequences showed high level of conservation across all samples. Sequence analysis of integrase gene using REGA and JPHMM identified two unique recombinants (G/AE and C/A1) within the integrase sequence. INSTI resistance analysis showed absence of major mutations while two accessory mutations were noted, E157Q in subtype C and G163R/K in subtype F as well as subtype C.

**Conclusion:** The RT-Nested PCR protocol could successfully amplify the integrase gene from plasma and DBS specimens from different subtypes. This can be useful for analysis of resistance to INSTIs across various HIV-1 subtypes in INSTI naive and failed individuals. Presence of accessory mutations in INSTI naive samples warrants nation-wide larger studies to understand INSTI resistance.

## ISSHID Abstract-78 The Prevalence and Pattern of Superficial Fungal Infections among patients visiting dermatology OPD of a tertiary care hospital in Warangal

### G.V. Padmaja, Efshana Jabeen, Anushya

#### Kakatiya Medical College, KNRUHS,Warangal,Telangana, India

**Background:** Superficial fungal infections are most common in tropical and subtropical countries. Superficial fungal infections of the skin are one of the most common dermatological conditions seen in clinical practice. It affects 20-25% of the population.

Materials: and **Methods:** In this study, 1473 Suspected superficial fungal infection cases were identified among approximately 3500 patients screened during August 2017 to August 2019.

The collected samples (skin, nail, and hair) were subjected to direct microscopy with potassium hydroxide and cultured on Sabourauds dextrose agar,after the fungal culture growth Lacto Phenol Cotton Blue mount was done to identify the fungal species.

**Results:** The prevalence of superficial fungal infection was 26.2% (386/1473), dermatophytosis was 63.7% (246/386), and non-dermatophytosis was 36.3% (140/386). Among the isolated dermatophytes, Trichophyton rubrum was the commonest species (68%). and Candida (70%) the commonest non-dermatophytes species. Tinea corporis was the commonest (58%) clinical presentation. Culture examination highlighted dermatophytes 68% Trichophyton rubrum, 19% Trichophyton mentagrophyte, 6% Trichophyton tonsurans, 2% Trichophyton violaceum , 2% Microsporum gypseum, , 1.5% Microsporum canis, 0.9% Trichophyton verrucosum 0.5% Epidermophyton floccosum and non-dermatophytes 38%Candida albicans , 32% Candida non albicans 10% Aspergillus niger , 7% Aspergillus flavus , 4% Aspergillus fumigatus 2% Alternaria,2% Curvularia,0.5%cladosporium

**Conclusion:** The current study illustrates that a notable number of patients had prevalence of superficial fungal infections, hence a prompt recognition of skin lesion and identification of these superficial fungal infections alarms us to undertake early diagnosis to improve the quality of life.

There were no ethical issues involved in this study

## ISSHID Abstract-80 *Leishmania donovani* Mevalonate kinase: an important factor in disease pathogenesis

### Tanvir Bamra, Ajay Kumar, Kumar Abhishek, Abhishek Mandal, Sushmita Das, Manjay Kumar, Rimi Mukherjee, Taj Shafi, Abhik Sen, Pradeep Das

#### Department of Molecular Biology, ICMR-RMRIMS, Patna- 800007, Bihar, India

**Background:***Leishmania donovani* causes visceral leishmaniasis in Indian subcontinent. Its pathogenesis is associated with its ability to get internalized in host cells which is attributed to Leishmania secretory proteins. Very few studies have addressed the role of secretory proteins in pathogenesis, especially in invasion. We investigated, one such protein, namely, mevalonate kinase (MVK), which is an enzyme of cholesterol biosynthesis pathway to explore its role in invasion.

**Methods:** Genomic PCR amplification was performed to verify if MVK is present in L. donovani. To examine if MVK is secreted out in extracellular milieu, the presence of MVK in outer milieu was analysed by Mevalonate kinase assay. Anti MVK antibody was obtained following cloning which was used to confirm the release of MVK by western blot. Further, to evaluate the role of LdMVK in invasion, RAW cells were treated with rLdMVK and parasite and number of internalized parasites were observed.

**Results**: PCR amplification confirmed that MVK is present in *L. donovani*. MVK activity was found in the *L. donovani* culture supernatant which suggested that it is released into the extracellular medium. This was further confirmed by Western blot. Treatment of RAW cells with rLdMVK and L. donovani parasites resulted in 2fold increase in internalized parasites.

**Conclusion**: In this study we have identified *L. donovani* MVK as a virulent factor that is released into the extracellular medium. We have also demonstrated that MVK plays an important role in the pathogenesis by helping in the initial phase of infection and inducing host invasion.

## ISSHID Abstract-82 Spotted Fever Rickettsiosis Presenting with Bilateral Anterior Uveitis and Retinitis: A case report

### Sohini Das^1^, George Abraham Ninan^1^, Smitha Jasper^2^, Minu George^2^, Ramya Iyadurai^1^

#### ^1^Department of Medicine, Christian Medical College, Vellore, India; ^2^Department of Ophthalmology, Christian Medical College, Vellore, India

**Background:** Spotted fever is a common rickettsial disease in India. It is caused by Rickettsia conorii, which demonstrates vascular tropism and causes endothelial injury. Ocular manifestations include multifocal retinitis and disc edema. Anterior uveitis as a presenting feature of spotted fever is uncommon.

**Case Presentation:** A 32-year-old man presented with fever, headache and myalgia for one week. He developed redness and pain in both eyes on Day 4 of illness. On examination, he was febrile, tachycardic, and had purpuric rash over palms and soles. Slit lamp examination showed bilateral non-granulomatous anterior uveitis. Fundus examination showed mild disc edema, scattered cotton wool spots, few hemorrhages and Roth’s spots bilaterally. There was no cardiac murmur, eschar or neck stiffness.

Investigations revealed thrombocytopenia and elevated transaminases. Malarial parasite smears, blood cultures, infective endocarditis workup, scrub typhus and dengue serology were negative.

Weil Felix test was positive for OX-19 and OX-2, consistent with spotted fever. Spotted fever IgM Elisa was also positive.

He was initiated on intravenous doxycycline. Fever subsided within two days. After defervescence, he was given oral doxycycline to complete one week of therapy. Topical steroids were given for anterior uveitis. Written informed consent for publication of case report and images was obtained from this patient

**Conclusion:** Differential diagnosis of spotted fever rickettsiosis should be considered in patients with febrile episode with rash and anterior uveitis. Clinicians should be vigilant about ocular manifestations of spotted fever. Prompt referral to an ophthalmologist for appropriate management and prevention of visual impairment is necessary.

## ISSHID Abstract-83 Standardization of TaqMan probe based Hexaplex qPCR Assay for Simultaneous Detection of Hepatitis A-G Viruses

### Priyanka Gupta, M Irshad, Sudip Kumar Datta

#### Department of Laboratory Medicine, All India Institute of Medical Sciences, New Delhi, India

**Background:** Viral hepatitis is associated with a number of viruses including Hepatitis A, B, C, D, E and G viruses. The accurate and early diagnosis is supposed to be beneficial in the management of viral hepatitis. Although various serological and molecular assays are available and currently in use by routine diagnostic laboratories, for detection of individual hepatitis viruses separately. However, these assays have several limitations. Present study was planned to develop a method for simultaneous detection of hepatitis A-G viruses associated nucleic acids using Multiplex qPCR.

**Methods:** This assay was developed using specific primers and TaqMan probes designed against the most conserved region of each hepatitis virus using bioinformatics tools. Multiplex qPCR was developed in different combinations for detection of six hepatitis viruses (A-G) using varying conditions including temperature, primer concentrations, and probe concentrations. All assays were optimized using standard plasmid control as a template for each virus. The plasmid control was prepared using pUC57 as a cloning vector. Standard curves were generated using a 10-fold serial dilution of positive control and used for quantification.

**Results:** The multiplex assay was found to be a sensitive, specific and potentially reproducible system for simultaneous detection of HAV-HGV. The detection limit for different viral genomes was found to be 880 copies/ml for HAV, 170 copies/ml for HBV, 132 copies/ml for HCV, 1700 copies/ml for HDV, 200 copies/ml for HEV, and 95 copies/ml for HGV.

**Conclusion:** The current assay offers a highly reliable and alternative molecular tool for the early diagnosis of viral hepatitis.

## ISSHID Abstract-84 Prevalence of oropharyngeal candidiasis among Human Immunodificiancy infected patients attending Government Hospital

### Darshana M V, Shymala R

#### Kodagu Institute of Medical Sciences, Madikeri, Karnataka, India

**Background:** HIV patients are at most risk of developing Oropharyngeal Candidiasis (OPC).Candida albicans is the most common organism causing OPC. Non albicans Candida are the emerging species. Candida species are getting resistance to anti-fungal drugs.

Aim is to isolate, characterize and anti-fungal susceptibility testing of Candida species.

**Methods:** Patients with curdy white patch in oral cavity were included in the study. Samples was collected in sterile swab and subjected for microbiological study. Candida species was characterized by gram stain, germ tube test and Dalamou-plate culture. 30 random samples were selected and anti-fungal susceptibility testing done for Fluconazole (10mcg) and Voriconazole (1mcg).

**Results:** Total 110 samples was collected, 76 showed Candida species. Prevalence of OPC is 69.09%. Mean age of the study population is 42 years. Commonest isolated is *Candida albicans* (77.63%) followed by *Candida tropicalis* (21.05%) and *Candida kefyr* (1.31%). Non albicans Candida were predominant in patients with CD4<200cells/mm3. Voriconazole sensitivity was 82.4% and Fluconazole showed 100% resistance among all isolates.

**Conclusion:***Candida albicans* is the commonest organism causing OPC. Candida tropicalis is second most common organism. Prevalence of Non albicans candida are significantly high in low CD4 count. Anti-fungal drugs getting resistant and they are not effective in treating OPC. All Candida species have got complete resistant to Fluconazole and more sensitive to Voriconazole.

## ISSHID Abstract-85 Improved Turnaround time for identification of Positive blood cultures by direct inoculation method in to VITEK 2 Compact systems

### Mullai Suresh, Devendiran Dhanasekaran, Banupriya Elumalai, Sripriya Dinesh

#### Anderson Diagnostics & Labs, Purasaiwalkkam centre, Chennai -600084

**Background:** Timely identification of positive blood culture is required for planning the treatment regimen for the patient with sepsis. Hence a rapid method was evaluated by direct seeding of positive blood cultures in to VITEK 2 systems for identification and antimicrobial susceptibility (AST).

**Methods:** A total of 100 isolates from positive bottles were directly inoculated into Vitek cards. A loop of the suspension was also inoculated onto blood agar for purity check. In brief, protocol followed for direct inoculation of vitek cards are, 3 milliliters of blood culture broth was transferred from the positive blood culture bottle into a serum separator tube (SST, Gel tube, yellow top, Becton Dickinson) and centrifuged at 3000 rpm for 10 min. The supernatant was discarded. Add 3 ml normal saline to it followed by brief vortexing. The turbidity of this inoculum was adjusted using normal saline to 0.5 McFarland and charged in to VITEK cards according to manufacturer’s instruction.

**Results:** Out of 100 isolates, 2 were misidentified by direct inoculation and there was no unidentified isolates. In AST 2% of isolates showed Very major error, No major error, 4% isolates had a minor error and 94% had essential MIC agreement between two methods.

**Conclusion:** Improvised TAT of approximately 12h from growth signal when compared to overnight subculture of bottles will have a better patient care and management. De-escalation of empirical therapy will reduce the pressure in antibiotic resistant era.

## ISSHID Abstract-86 Multi-omics approach to study the cellular events during HIV- *H. pylori* coinfection

### Vidhya Natarajan^1^, Preeti Moar^1^, Urvinder Kaur S^1^, Abhishek Kumar^2,3^, Ravi Tandon^1^

#### ^1^Laboratory of AIDS Research and Immunology, School of Biotechnology, Jawaharlal Nehru University, New Delhi, India; ^2^Institute of Bioinformatics, International Technology Park, Bengaluru, India; ^3^Manipal Academy of Higher Education (MAHE), Manipal, Karnataka, India

**Background:***Helicobacter pylori*, a gram-negative microaerophilic bacterium is reported to be more than 60% prevalent in Indian population. Therefore, it is very likely that HIV and H. pylori infection may co-exist. HIV infection results in the establishment of latent reservoirs which are the major barrier in HIV eradication. There are no studies elucidating the effect of H. pylori on HIV reservoir and pathogenesis. We seek to investigate the cellular events during HIV-H. pylori coinfection using omics approach.

**Methods:** We performed multi-omics analyses on *H. pylori* stimulated latently HIV-1 infected myeloid cells (U1 cells) and compared with unstimulated U1 cells using RNASeq and LC-MS analysis. Based on significant Differentially Abundance Protein (DAPs) and our data on Differentially Expressed Genes (DEGs), common proteins were selected. Gene Ontology (GO) for common proteins were predicted using GORILLA software and pathways were predicted using Reactome to identify the interactions between common DAPs.

**Results:** Our bioinformatic analysis showed 21 common significant gene/protein with similar expression profile from combined analysis of DAPs and DEGs. The HIV specific proteomic analysis confirmed significant expression of HIV Gag in H. pylori stimulated U1 cells. Functional analysis revealed IL1β, HCK and ADP-ribosylation factor-1 among others are involved in HIV specific host response and cytokine signaling.

**Conclusion:** The study suggests that *H. pylori* stimulation of latently HIV-infected U1 cells alters the protein expression leading to regulation of HIV specific proteins along with other host proteins which plays an essential role in H. pylori mediated reversal of HIV latency.

## ISSHID Abstract-87 Crusted scabies - A rare case series

### Vijayasankar Palaniappan, Gowtham Saravanan, Hima Gopinath, Karthikeyan kaliaperumal

#### Department of Dermatology, Venereology & Leprosy, Sri Manakula Vinayagar Medical College, Puducherry, India

**Background:** Crusted scabies is a rare highly contagious variant of scabies caused by uncontrolled proliferation of the mite Sarcoptes scabiei var hominis. The affected patients harbor millions of mites in their skin. Here we report three rare cases of crusted scabies with varying causes of underlying immunosupression

Case series : The first case was a 54-year-old diabetic male who had received multiple intramuscular dexamethasone injections for his generalised itching from a local practioner. The second case was a 62- year- old diabetic female with hemiparesis and third case was a 50- year old male with adenocarcinoma lung on chemotherapy and radiotherapy. All the three cases had non itchy erythematous to yellowish hyperkeratotic crusted plaques over their trunk, gluteal region and extremities. The examination of skin scrapings with mineral oil under microscopce demonstrated numerous mites, egg and scybala confirming the diagnosis of crusted scabies. The consent has been obtained from all the patients for the publication of their personal data

Discussion: Crusted scabies primarily affects immunocompromised, elderly, disabled and debilitated individuals. The problems with crusted scabies are its asymptomatic nature in many, mimics many other dermatoses and can easily trigger epidemiological outbreaks. Its complications include secondary bacterial infections, generalised lymphadenopathy, sepsis, erythroderma. It is necessary to hospitalise the patient to minimize the contacts, environmental decontamination, treat all the close contacts prophylactically. We treated all our patients with topical keratolytics, topical 5% permethrin cream daily for one week and oral ivermectin 12mg on day one, two and eight and they responded well to treatment:

**Conclusion:** Thus any crusted lesion in a moribund patient should arouse a suspicion of crusted scabies.

## ISSHID Abstract-89 Highly conjugated 1,10-Phenanthroline derivative bearing metal complexes towards tuberculosis: synthesis, characterization and antimycobacterial activity

### E.H.Edinsha Gladis^1,2^, K.Nagashri^1^, J. Joseph^2^

#### ^1^Dept. of Chemistry, Manonmaniam Sundaranar University, Tirunelveli, Tamilnadu; ^2^Dept. of Chemistry, Noorul Islam Centre for Higher Education, Kumaracoil, Tamilnadu

**Background:** In the world wide, the mycobacterium tuberculosis is one of the challenging communal health issues. The conventional anti-tuberculosis therapeutic approaches are lengthy, toxicity and create side effects to humans. Therefore, there is an imperative requisite to search for innovative anti-Tuberculosis agents with inventive molecular architectures that offers unique mechanistic action against mycobacterium strain. Metal(II) complexes containing 1,10-Phenanthroline derivatives afford versatile coordination behaviour which mimics the natural copper enzymes and enhancing pharmacological activities. The present research work was intensive on the synthesis, structural elucidation and antituberculosis activities of bioactive metal(II) complexes with the molecular formulae of [MIIL] where Ni(II), Cu(II), Zn(II) and Co(II); L = 1,10-Phenanthroline derivative.

**Method:** The MIC values were noticed for ligand and its metal complexes against M. tuberculosis strain, H37Rv using Microplate Alamar Blue assay (MABA) technique as compared with Ethambutol as standard.

**Results:** The observation was noted as MIC values of prepared metal complexes (3-10μg/ml) are greater than the drug (1μg/ml), Ethambutol.

**Conclusion:** Copper complexes showed higher anti-mycobacterial efficiency than other complexes and 1,10-Phenanthroline derivatives due to its lipophilic nature of copper complex (electron transfer route which makes in the development of oxidative stress or the disruption of electron transport phenomenon) and highly conjugate 1,10-Phenanthroline (ligand core) facilitates the diffusion into the cell membrane and leads to cell destruction. Based on our studies, we conclude that the readily available substrate and simple way to achieve Cu(II)-1,10-Phen complex provide selective and harmless lead molecule in the pursuit for new agents for the treatment of Tuberculosis.

## ISSHID Abstract-91 Emerging Viral Infections in India and Blood Safety

### Ravindra Prasad Thokala, Ashwin Anandan, Krishnamoorthy Radhakrishnan, Mahalakshmi Gopal

#### Department of Transfusion Medicine, Sri Ramachandra Medical college and Research Institute. Sri Ramachandra Institute of Higher Education and Research ,Chennai , Tamilnadu, India

**Background:** Blood transfusion can lead to transmission of infections that the donor harbours. In India, blood donors are screened for HIV, Hepatitis B, Hepatitis C, Malaria and Syphilis. Re-emerging and Emerging viral infections like Dengue, Chikungunya, H1N1 influenza, Zika , Ebola ,West Nile virus and Japanese encephalitis virus can be transmitted through blood transfusion and pose health risks.

**Methods:** A review of literature was done on Google database using the boolean logic. Articles were analysed for prevalence and evidence of transfusion transmission of viral infections.

**Results:** Fourty eight articles were included for review. Transfusion transmission of chikungunya has been a proven risk in countries like Thailand. Transmission of dengue through blood transfusion and transplantation has been reported from Singapore, Hong Kong and Germany. Transfusion transmission of Zika virus has been documented in Brazil and French Polynesia. No reports of transfusion transmission of Chikungunya, ZIKA and dengue could be found from India. Transfusion transmission of Ebola infection has not been reported anywhere in the world. Nipah virus infection has been reported in India, but no transfusion transmission had been documented .West Nile virus and Japanese encephalitis virus are widely prevalent in India but no transfusion transmission of these infections are documented.

**Conclusion:** Transfusion transmission attributes of HIV,HBV ,HCV warrant screening of all donor blood units .The Emerging viruses though has the potential to transmit via blood donation does not require screening tests at present due to lack of evidence for transfusion transmission in India

## ISSHID Abstract-93 The Impact of Dual Disease Burden on Patient Outcome: Association of Interleukin-10 Polymorphism in Pulmonary Tuberculosis with Type 2 Diabetes Mellitus

### Swathy Moorthy, Teena Koshy, Jahnavi

#### Sri Ramachandra Institute of Higher Education and Research (SRIHER), Chennai, India

**Background:** The dual disease burden of Type 2 Diabetes Mellitus (T2DM) and Pulmonary Tuberculosis (PTB) is rapidly accelerating. Several studies have established that host genetic factors play a role in determining host susceptibility to mycobacteria as well as the course of disease. There is evidence suggesting that there is a direct inter-relationship between Interleukin-10 (IL-10) and the severity of PTB, since IL-10 plays an important role in controlling inflammation. Certain single nucleotide polymorphisms within the promoter region of the IL-10 gene have been associated with altered levels of circulating IL-10. The aim of this study was to investigate the association between the IL-10 −592A/C polymorphism, PTB and T2DM; as individual diseases and co-epidemics in a South Indian population.

**Methods:** The study enrolled 30 patients diagnosed with PTB, 30 patients with T2DM and PTB and 30 patients with T2DM alone. DNA was isolated from blood samples and the IL-10 polymorphism was determined by polymerase chain reaction restriction fragment length polymorphism (PCR-RFLP) using a RsaI restriction endonuclease. The digested fragments were subsequently analysed by agarose gel electrophoresis.

**Results:** Analysis of genotype frequencies showed that IL-10 −592 C allele was predominant in all three groups. The heterozygote frequency in the PTB group was 3.3%; however, this was not statistically significant in comparison with the other two groups of patients.

**Conclusion:** Our findings suggest of IL-10 −592A/C may not be associated with PTB and T2DM in our population. Nevertheless, the findings of this study need to be corroborated in a larger population.

## ISSHID Abstract-94 Cholestatic Jaundice in A HIV positive patient - A Case Report

### Swathy Moorthy, Prarthana R, Srinivasan R, Manoj Prabhakar, K.Madhavan

#### Department of General Medicine, Sri Ramachandra Institute of Higher Education and Research (SRIHER), Chennai, India

**Background:** HIV Cholangiopathy is not an uncommon entity in the setting of AIDS. Though common in pre HAART era with an incidence of 26-46%, it is still occurring to date in AIDS patients with CD4 <100 and in presence of opportunistic infections or when they develop resistance to the first line ART drugs.

**Methods:** Here we report a 55 year old retropositive male after obtained his consent to present his clinical details. He was not on ART presenting with complaints of abdominal pain with loss of appetite, fatigue, high coloured urine, yellowish dicolouration of eyes and generalised itching for 3 months that increased over the last 10days. On examination, he was found to have right hypochondrial tenderness. Baseline evaluation showed features suggestive of cholestatic pattern of jaundice. He was found to be HBsAg positive, a low CD4 count 72, suggestive of a clinical possibility of HIV Cholangiopathy. There was no evidence of Opportunistic infections on further evaluation with blood, urine and stool culture and sensitivity. His fundus examination did not reveal any evidence of CMV retinopathy.

**Results:** The patient had dilated IHBR on USG abdomen while MRCP revealed multiple areas of irregularity, narrowing and beaded appearance involving the intrahepatic biliary radicals, short segment stricture involving distal common bile duct, dilated CBD and CHD and no evidence of calculi/mass which are characteristic features of HIV Cholangiopathy - thus confirming the diagnosis.

**Conclusion:** Treatment directed towards opportunistic infections usually does not improve cholangiographic abnormality. Though surgical management like ERCP sphincterotomy could be tried, most useful modality would be medically managing the patient with Ursodeoxycholic acid and ART.

## ISSHID Abstract-95 Prevalence of Cardiovascular risk factors among adult Patients Living with HIV - A Hospital based cross-sectional study from Puducherry, India

### Manikandanesan Sakthivel^1^, Venkatachalam Jayaseelan^2^, Palanivel Chinnakali^3^, Abdoul Hamide^4^

#### ^1^State Program and Technical Manager, CaP TB Project, SAATHII, India; ^2^Jawaharlal Institute of Post-graduate Medical Education and Research (JIPMER), Puducherry

**Background:** Cardiovascular diseases (CVD) are the most common cause of deaths due to Non-communicable Diseases (NCDs). Studies have shown increased prevalence of CVD among HIV patients due to interplay of traditional risk factors of CVD, HIV and anti-retroviral therapy. Though studies were available on prevalence of individual cardiovascular risk factors, comprehensive assessment of CVD risk factors had seldom been done previously. Hence, we aimed to determine the prevalence of selected cardiovascular risk factors among adult patients living with HIV (PLHIV).

**Methods:** This was a cross-sectional study conducted among all adult PLHIV (≥18 years) attending ART centre in a tertiary hospital from September 2016 to February 2018. Patients were surveyed with STEPS questionnaire to assess physical activity, alcohol and tobacco use and with Cohen’s perceived Stress Scale and Pittsburgh Sleep Quality Index. Weight, height, abdominal circumference and blood pressure were also measured along with biochemical investigations such as blood glucose, lipid profile and renal parameters. Prevalence was expressed as proportion and association between adherence and risk factors as prevalence ratio.

**Results:** Data were analysed for 316 patients. Mean (SD) age of the participants was 45 (9) years, 52% were males, 59% currently married and 73% currently employed. Prevalence of CVD risk factors found was as follows: current alcohol use-12.8%, current tobacco use-5.4% among males, hypertension-15.8%, diabetes-12.3%, dyslipidaemia-82.7%, impaired renal function-15.7%, abdominal obesity-34.2%, poor sleep quality-33.5%, very high stress level-38.8%, inadequate physical activity-74.4%.

**Conclusion:** CVD risk factors were high among PLHIV which advocates for health promotion activities and regular screening for CVD.

## ISSHID Abstract-100 A retrospective analysis of clinical spectrum of cutaneous tuberculosis from South India

### Mahalakshmi Veeraraghavan , Anuradha Priyadarshini , Adhikrishnan S, Sudha R, Murugan S

#### Dermatology Department, Sri Ramachandra Institute of Higher Education and Research, Chennai, India

**Background:** Tuberculosis continues to be a major infective disease and public health concern in India. Cutaneous tuberculosis constitutes about 1.4% of extrapulmonary tuberculosis.

**Methods:** The case records of all patients diagnosed to have cutaneous TB, between December 2017 to June 2019, was reviewed.

**Results:** Twelve cases of cutaneous TB were seen in 18 months duration. Age of the patients ranged from 8 years to 72 years, there were 7 males and 5 females. Disease duration ranged from 3 months to 1 year. There were 6 lupus vulgaris, 4 tuberculosis verrucosa cutis (TBVC), and 2 papulonecrotic tuberculids. Majority (10/12) of patients were asymptomatic. Pulmonary TB was present in the 2 patients with tuberculids. Tuberculids presented as disseminated lesions. The remaining 10 cases were not a/w systemic TB, the sites of involvement were lower limb in 4 patients, upper limb in 2 and face in 3, and trunk in 1 patient. In all the 12 cases the histopathological features was highly suggestive of TB, based on the findings of tuberculoid granulomas. Special stain for AFB was positive in one single case. Mantoux test was positive in 6/10 cases (excluding tuberculid cases). All 12 cases were HIV negative.

**Conclusion:** Lupus vulgaris and TBVC are the most common types of cutaneous TB. Albeit indolent, cutaneous TB may be associated with systemic TB and is an indicator of prevalence of TB in the community. A strong clinical suspicion and better diagnostic facility is needed for prompt diagnosis and treatment of these cases.

## ISSHID Abstract-102 Is Syphilis Making A Comeback ?

### Ashwin Anandan, Krishnamoorthy Radhakrishnan, Ravindra Prasad Thokala

#### Sri Ramachandra Institute of Higher Education and Research (SRIHER), Chennai, India

**Background:** Blood Transfusion Services is the vital part of health care system without which efficient medical care is impossible. The role of Blood Transfusion Service is to provide safe and effective blood and blood products to meet the patients need. In India, all donated units has to be screened for mandatory markers which includes HIV-1&2, Hepatitis B & C virus, Malaria and Syphilis as per regulations. This study was carried out with an objective of estimating the seroprevalence of Syphilis among voluntary blood donors at our institution

**Methods:** Informed consent was obtained from blood donors to screen their donated units for transfusion transmissible infections (TTI) as per National Blood Transfusion Council (NBTC) guidelines. Syphilis screening is perfomed using RPR (Rapid Plasma Reagin) test. RPR is a macroscopic Treponema flocculation test for the detection of antilipoidal antibodies. Descriptive statistics was used to estimate prevalence (January 2015 to July 2019).

**Results:** During the study period, a total of 54991 donors had donated blood of which 28 donors were positive for RPR. Seroprevalance for Syphilis was 0.05%.

**Conclusion:** All though transfusion transmissible Syphilis is not a major risk (as the spirochetes do not survive in blood at 4C for 72 hours) still the risk of concomitant HIV and high risk behavior exists. Hence careful and stringent blood donor selection with respect to high risk behavior and promiscuous activity is the need of the hour.

## ISSHID Abstract-103 Seroprevalence of HIV among blood donors

### Arpitha Puttegowda, Krishnamoorthy Radhakrishnan, RavindraPrasad Thokala, Ashwin Anandan, Panicker V K

#### Department of Transfusion Medicine, Sri Ramachandra Medical College and Research Institute, Sri Ramachandra Institute of Higher Education and Research, Chennai, Tamilnadu, India

**Background:** Transmission of infectious diseases through blood donation is a concern of blood safety as transfusion is an integral part of medicine and surgical therapy. One of the mandatory routine tests undertaken in a blood bank is screening of viruses including HIV. With any unit of the blood transfused there is a 1% chance of transfusion transmissible infections. Hence sophisticated screening and testing of blood donors is needed to reduce transfusion transmissible infections. The aim of this study was to know the seroprevalence of HIV among blood donors.

**Methods:** Donors who walked in for voluntary blood donation were selected based on standard criteria for donor fitness. Screening tests were conducted on every blood unit to exclude HIV infection. During the study period from January 2015 to July 2019, 54991 number of donor samples were screened. Screening was done using Automated ELISA analyser. Descriptive statistics was used to estimate prevalence.

**Results:** Thirty one donor samples were tested reactive for anti HIV1 & 2 out of 54991 samples screened. Seroprevalence of HIV was 0.056%

**Conclusion:** Prevalence of HIV among blood donors is much lesser than among general population. Blood transfusion is a potential route of transmission, although, risk may be reduced by vigorous screening of donors and donated blood. Currently all steps are taken to fight against transfusion transmitted infections like HIV. Educating people and creating awareness about voluntary blood donation is an important factor.

## ISSHID Abstract-104 Potential scope of a novel study tool for measuring motivational values of mother towards female child in scaling values among People Living with HIV/AIDS (PLHIV)

### Dinesh Kumar^1^, Richa Kalia^2^

#### ^1^Department of Community Medicine, Dr. Rajendra Prasad Government Medical College, Kangra, Himachal Pradesh, India; ^2^District Program Office, Department of Health and Family Welfare, Una, Himachal Pradesh

**Background:** Authors have structured and tested the scale to measure motivational values among women towards female child using structure proposed by Shalom H. Schwartz. The structure measured values for ten domains; power, achievement, hedonism, stimulation, self-direction, universalism, benevolence, tradition, conformation and security.

**Methods:** Questionnaire comprising of 59 questions was developed and data from 350 women were collected. Data was collected from women with children less than 5 year of age. Exploratory Factor Analysis (EFA) and assessment for Cronbach’s alpha were done to assess its internal validity and consistency.

**Results:** Total eight factors extracted maximum (64.7%) of variance with significant Barlett’s test of sphericity and high (0.80) Kaiser-Meyer-Olkin value. Questions with >0.45 factor loading were considered significant and out of total 59 questions following number of questions loaded successfully; 8 for power, (of 15), 2 for achievement (of 3), 2 for hedonism (of 2), 5 for self-direction (of 7), 4 for benovalence (of 5), 3 for tradition (of 3), 3 for conformity (of 4), and 3 for security (of 6). Inclusion of questions from domains of power subset (4 questions), achievement, self-direction, benovalence, and tradition observed with good (>0.80) internal consistency based on value of Cronbach’s alpha.

**Conclusion:** It is feasible to administer questionnaire to measure motivational values, wherein analysis has identified a total of 30 questions extracted majority of information from the dataset. Whereas, further reduction of questions from 30 to 18 improves the internal consistency of the scale.

## ISSHID Abstract-109 Intrameatal renal calculi mimicking a genital wart

### S.Adikrishnan, S.Sukesh Gautam, M.Krishnakanth, Gayathri Rajesh, S.Murugan

#### Department of Dermatology, Venereology and Leprosy, Sri Ramachandra Institute of Higher Education and Research, Porur, Chennai, India

**Background:** Condylomata acuminata caused by HPV is not an uncommon entity in clinical practice. They are the most common sexually transmitted viral diseases worldwide and are easily diagnosed and misdiagnosed in different circumstances. Urethral and intrameatal extension of warts are seen in 5% of men. On the other hand, urinary calculi which usually pass spontaneously while voiding, may get impacted rarely in the urethra during their passage and produce variable symptoms.

**Case Report:** A 20-year-old male presented with history of growth over the tip of the penis for 1 week associated with difficulty in micturition. There is a positive history of unprotected sexual contact with female commercial sex worker 2 months back. On examination, the patient had a verrucous papule impacting the urethral meatus. No other lesions elsewhere in the skin or genital or perianal region. The patient was investigated for HBsAg, anti-HCV, HIV Elisa and VDRL which were all nonreactive. Differential diagnoses considered were inverted papilloma of urinary tract and intrameatal condyloma acuminatum. The patient was then referred to urology for removal. To our surprise, the patient was found to have multiple dropped calculi from the urinary tract that were impacted in the urethra. The patient has given consent to the publication of their personal data.

**Conclusion:** Appearances are often deceptive” is a popular proverb that emphasises the importance of analysing things thoroughly before arriving at a conclusion. Silent calculus presenting at the tip of glans is an extremely rare entity. High index of suspicion is hence required to rule out this uncommon presentation.

## ISSHID Abstract-110 Seroprevalence of HCV among the blood donor population

### Mahalakshmi Gopal, Krishnamoorthy Radhakrishnan, Ravindraprasad Thokala, Ashwin Anandan, Panicker V.K

#### Department of Transfusion Medicine, Sri Ramachandra Medical College and Research Institute, Sri Ramachandra Institute of Higher Education and Research, Chennai, Tamilnadu, India

**Background:** Transfusion of blood and blood products which is considered as lifesaving modality, may lead to certain infectious complication in the recipients. The purpose of the study was to know the seroprevalence of anti-HCV among the blood donor population in a tertiary care hospital based blood bank, to evaluate the trends over the years Jan 2015- July 2019.

**Methods:** Blood units collected from all eligible blood donors who donated Whole Blood were subjected to mandatory screening by ELISA for antibodies to HCV. Data was retrieved from Jan 2015- July 2019 and total number of donors who were reactive for anti-HCV was noted. Descriptive statistics was used to estimate prevalence.

**Results:** Out of 54991 blood donor samples screened, 118 samples were reactive for anti-HCV. Seroprevalence was 0.214% for HCV.

**Conclusion:** HCV infection is associated with a high risk of chronicity which leads to liver cirrhosis and may progress to hepatocellular carcinoma. HCV was shown to be the known cause of 90% of Non-A Non-B (NANB) transfusion related hepatitis. NACO states that the prevalence of HCV among blood donors in 2015 in India is 0.34%. Currently no vaccine is available against HCV infection, Transfusion transmissible HCV infection is a major threat to safety of blood supply. This indicates the necessary to continue screening donated blood for HCV infection with highly sensitive and specific tests.

## ISSHID Abstract-113 Chemical modification of bioactive flavone derivatives with metal complexes towards antimycobacterial agents

### R.R. Krishna Jyothi, J. Joseph, Sharow Geeth Vincent

#### Department of Chemistry, Noorul Islam Centre for Higher Education, Kumaracoil 629 180, Tamilnadu, India

**Background** The WHO TB statistics indicated that 2.79 million peoples were affected by tuberculosis. The clinical success rate against tuberculosis only below 75% due to the resistance of body mechanism towards existing drug delivery systems. Hence, there is critical need for newer structural modifications in the existing bioactive molecule with advanced drug delivery features is a mandatory for the present condition worldwide. Literature studies revealed that heterocyclic ligand molecules with different substitutions and modifies structures offers varied anti-microbial activities and more activity against different strains of bacteria. The present work was intensive on the anti-tuberculosis efficacy of complexes of metals like Cu(II), Co(II), Zn(II) and Ni(II) with flavone derivatives.

**Methods:** The anti-tuberculosis study was carried out by MABA method towards H37Rv strain of mycobacterium tuberculosis. The standard ethambutol was used for comparison of efficiency of prepared compounds.

**Results:** Among the prepared complexes, greater inhibition of tuberculosis strain with the IC50 value 5.95μg/mL whereas other metal complexes showed slightly lower activities as compared with ethambutol.

**Conclusion:** The anti-mycobacterial activity of the prepared complexes was analysed. The potential efficiency of complexes are attributed to the electron withdrawing substituent present in the ligand molecule and also participate to enhance the lipophilic property followed by reduce the toxicity of complexes. Furthermore, the redox characteristics of copper complex may also diminishing the growth of microbes through different biological processes. Hence, it is confidence that the prepared copper complex of flavone derivatives may be prove to be excellent candidate for anti-TB

## ISSHID Abstract-115 Prevalence of pediatric tuberculosis in Chennai- A cross sectional study

### Suganthan R, Kalpana.s, Joseph Maria Adikalam, Srinivas G

#### Department of Epidemiology, The Tamil Nadu Dr.M.G.R.Medical University, Guindy, Chennai, India

**Background:** According to WHO estimates of 2015, 1.8 million people are estimated to have died from tuberculosis, of which 0.2 million were children. Often pediatric tuberculosis is underdiagnoses hence the real burden is not estimated correctly.Hence, this study was undertaken to estimate the prevalence of pediatric tuberculosis.

**Methods:** Pediatric cases from June 2018-June 2019 from all the 15 RNTCP centers were enrolled for this study. Case history was obtained from case sheets and from parents. Data regarding sociodemographic details, contact history, BCG vaccination status, Mantoux status, Chest X-ray, HIV status were collected. Ethical clearance was obtained from the Tamil Nadu Dr.M.G.R.Medical University.

**Results:** Totally 153 cases were enrolled for this study. Of which, Fourty four percentage of pulmonary tuberculosis and 55 percentage of extra pulmonary cases were registered in all 13 RNTCP centers in Chennai. Among 15 centers, in two centers, no pediatric cases were registered. Number of cases was high in 6th Zone (Pulianthopu). Among the total, 80 of them were female, and 109 belonged to hindu religion. Many of them were living in nuclear family (67%).68% of them were Mantoux Positive. Around 56% of them had contact with tuberculosis. 98% of them were BCG vaccinated. 98% of them were treated under the category I.

**Conclusion:** In this study, it was observed that extrapulmonary cases were more than pulmonary tuberculosis. This study could identify, number of family members is the associated risk factor for tuberculosis but other demographic profile is not associated with pediatric tuberculosis.

## ISSHID Abstract-117 Estimating Prevalence of Herpes Simplex Virus-2 Infection in Genital Ulcer Disease Patients using ELISA and PCR

### Gowsalya Saminathan, B. Rayvathy

#### Department of Microbiology, Dr. ALM Post Graduate Institute of Basic Medical Sciences, University of Madras, Taramani, Chennai, India

**Background:** Genital Herpes infection is a major Sexually Transmitted Infection with a high prevalence and is the most common cause of genital ulcer disease worldwide. Virus isolation in cell culture despite being the gold standard diagnostic method is a slow and labour intensive process. ELISA using the glycoprotein G (gG-2) purified from HSV-2 infected cells is capable of determining the HSV-2 type-specific antibodies thereby enabling the feasibility of specific IgG and IgM assay for precise detection and also the application of Polymerase Chain Reaction (PCR) offers a more rapid and sensitive technique for the identification of HSV-2. Hence with this background the current study was done to detect the prevalence of HSV-2 infection using ELISA and PCR among genital ulcer disease patients.

**Methods:** Serum and Genital swabs were collected from 60 Genital ulcer disease patients. Institutional Human Ethics Committee Approval was obtained. The serum was subjected to ELISA using Serolisa TM HSV-2 IgG & IgM ELISA Kit (K-1030 & K-1031). The DNA was extracted from the swab specimens using Nucleospin Virus Kit (MACHEREY NAGEL) according to manufacturer’s instruction and subjected to PCR using specific primers targeting glycoprotein G gene.

**Results:** The results revealed that IgG ELISA detected HSV-2 type-specific IgG antibodies in 45% of the samples and none of the samples showed IgM positivity. PCR was capable of detecting the HSV-2 DNA in 45% of the samples.

**Conclusion:** Thus this study estimates the prevalence of HSV-2 infection using ELISA and Conventional PCR in genital ulcer disease patients.

## ISSHID Abstract-119 Role of N-acetyl cysteine in HIV- 1 Infection induced oxidative stress and cell death in TZM-bl cells

### Pratiksha Jadaun, Leila Footoh Abadi, Smita S. Kulkarni

#### Division of Virology, ICMR-National AIDS Research Institute – Pune, India

**Background:** The N-acetylcysteine (NAC) is a thiol-containing free radical scavenger that is known to reduce oxidative stress. Studies have indicated an enhancement of oxidative stress/reactive oxidative species (ROS) in HIV infection, however, its role at the cellular level is not clearly understood and thus, the current study was undertaken.

**Methods:** TZM-bl cells were selected as a model for study. The cell viability was determined by MTT and ATPlite assays. The ability of NAC to inhibit the replicative capacity of HIV-1 primary isolates (R5, Indian subtype C and X4, Ugandan subtype D) was assessed. The generation of reactive oxygen species (ROS: superoxide anion and hydrogen peroxide) was measured using MitoSOX TM and DCF-DA probes and observed through confocal microscopy. The cell death was measured using Alexa Fluor® 488 Annexin V/ Apoptosis Kit.

**Results:** The NAC was found to be nontoxic (CC50: 18.30mM/mL) having the potential to inhibit HIV-1 replication (IC50: ≤9.5 mM/mL). The confocal studies indicated that virus-infected cells exhibited enhanced fluorescence due to increased ROS generation in the cells while NAC was found to abrogate this effect. Furthermore, NAC was found to reduce the necrotic population by 31.7% and apoptotic population by 0.6% as compared to untreated cells indicating that this might be due to the effect of NAC on ROS mediated cell death.

**Conclusion:** The study indicates free radical scavenging activity of NAC at the cellular level along with anti-HIV-1 activity and the use of NAC as an adjunct to ART in HIV management.

## ISSHID Abstract-121 Molecular characterization of extended spectrum β-lactamase and metallo-β-lactamase positive Enterobacteriaceae isolates

### Bowiya P, Janani N, Ishrath Razia R, Prabu D

#### Department of Microbiology, Dr.ALM PGIBMS, University of Madras, Taramani, Chennai-113, India

**Background:** Extended-spectrum β-lactamase (ESBL) and metallo- β-lactamase (MBL) positive Enterobacteriaceae isolates are found to be the serious threat in healthcare sectors. Potential dissemination of ESBL and NDM isolates create a therapeutic challenge in our country. Hence, the present study was conducted to determine the ESBL and MBL genotypes in Enterobacteriaceae isolates.

**Methods:** The clinical strains obtained from a tertiary care center were characterized by traditional microbiology techniques. Phenotypic detection of ESBL and MBL were performed as per Clinical Laboratory Standards Institute (CLSI) guidelines. Genotyping of ESBL (bla CTX-M, bla SHV ,bla TEM) and MBL (NDM) genes were characterized by multiplex PCR.

**Results:***E. coli* and *K. pneumoniae* were found to be the predominant strains among the Enterobacteriaceae group. The majority of the isolates (75%) were showing resistance to beta lactam antibiotics. Genotyping of ESBL and MBL showed positive for blaCTX-M (37%), blaSHV (37%), blaTEM (75%) and NDM (37%) gene, respectively, in our study isolates.

**Conclusion:** Our study shows the prevalence of ESBL and MBL resistance among the clinical isolates. Regular surveillance is required to control dissemination of resistant isolates among the population.

## ISSHID Abstract-123 Quality of life and stigmatization faced by active tuberculosis patients in Kancheepuram district, Tamilnadu - a qualitative study on physical, mental and social contexts

### S.Vidhyalakshmi, Shanthi Edward, R.V.M. Anantha Eashwar

#### Sree Balaji Medical College and Hospital, Chennai, Tamilnadu, India

**Background:** Tuberculosis (TB) remains a leading infectious cause of morbidity and mortality throughout the world and India has highest burden.Medication non‐compliance,one of the major drawbacks in the successful management of TB,is mainly due to drastic changes in routine life and stigmatization. Also drugs produce effects making them physically drained. The aim of the present study is to assess the life-quality and stigmatization against them.

**Methods:** A qualitative study using in-depth-semi-structured-interviews was conducted among TB patients in the field practice areas of a private medical college in Chennai, India. Data saturation was attained after interviewing 18 active TB patients. Interview included questions relating to symptoms, disease, treatment and stigma and Content analysis was performed.

**Results:** From this study, it is found that most of the patients when they developed symptoms, they had hesitance to visit doctor and tried to resolve by self-medication. After being diagnosed with TB, most of them did not disclose their condition to others for the fear of being isolated. When people came to know about their condition, they maintained distance, some even avoided them and many lost their jobs. Their routine life was also affected. During treatment, many due to adverse effects and some due to betterment of health thought of discontinuing the therapy. Their main concern was infection to their family and their condition affecting their children’s life.

**Conclusion:** Tuberculosis, a stigmatized disease, affects the quality of life of patient and family. Rehabilitation programs and counseling to be made for adherence to therapy and health education to reduce stigma.

## ISSHID Abstract-125 Molecular identification of *Streptococcus mutans* using 16s rRNA gene PCR-RFLP

### Iswarya A, Thota Akhil Raj, Thangam Menon, Sambhavi Bhagavatheeswaran,Vinu Ramachandran, Anandan Balakrishnan, Prabu D

#### Dr.ALM PGIBMS, University of Madras, Taramani, Chennai-113, India

**Background:***Streptococcus mutans* is known to be associated with human dental caries. Identification of *S.mutans* by conventional biochemical tests is often time consuming and inconsistent. The aim of the present study was to characterize strains of *S.mutans* using 16s rRNA gene PCR-RFLP.

**Methods:** Samples of oral rinse were collected using sterile phosphate buffered saline from 24 healthy individuals. Out of 24 samples tested, *S.mutans* was isolated from 11 samples and identified using Mitis-Salivarius agar and biochemical tests. DNA was extracted using boiling lysis method and 16s rRNA gene PCR was done with universal primers. The PCR products were subjected to restriction digestion using Hpa II enzyme. The restriction patterns were analyzed using Bio Rad Gel documentation system.

**Results:** All 11 strains of *S.mutans* were digested with Hpa II enzyme. 4 different restriction patterns were seen among the 11 strains studied.

**Conclusion:** 16s rRNA gene PCR-RFLP method was found to be a useful method for strain differentiation of *S.mutans.*

## ISSHID Abstract-126 Multidrug resistant *magA(K1),wzy(K2)* and Hypervirulent *(rmpA ) Klebsiella pneumoniae* causing pulmonary infection

### D.Danis Vijay^1^, S.Jayanthi^1^, N.Meenakshi^2^, S.H.ShifaMeharaj^1^, A.Sujhithra^3^, J.Perumal^1^

#### ^1^Department of Microbiology, Chettinad Hospital and Research Institute, CARE- Chettinad Academy of Research & Education, Kelambakkam, Tamilnadu, India. ^2^Department of Respiratory Medicine, Chettinad Hospital and Research Institute, CARE- Chettinad Academy of Research & Education, Kelambakkam, Tamilnadu, India. ^3^Department of Allied Health Science, Chettinad Academy of Research & Education, Kelambakkam, Tamilnadu, India

**Background:** Rapid spread of Multi Drug Resistant (MDR) and Hypervirulent *Klebsiella pneumoniae* (hvKP) strains with restricted treatment choices are the significant challenges. A strain with capsular mucopolysaccharide is known as hypervirulent *K. pneumoniae* (hvKP).The aim of the study to identify the cKP and hvKP strains causing pulmonary infections. Genotypic confirmation of K1,K2 serotypes and hvKP by multiplex PCR.

**Methods:** This prospective case control study was carried out in Chettinad Hospital and Research Institute from June 2018 - December 2018 after the Human Institutional Ethical committee approval. After phenotypic confirmation of hypervirulent *Klebsiella pneumoniae* the virulent genes, magA(K1), wzy (K2) and rmpA were identified by Multiplex PCR.

**Results:***Klebsiella pneumoniae* isolated from respiratory samples showed MDR. The string test was done for phenotypic confirmation of hvKP, n=54 were positive. Serotypic confirmation of hvKP strain were done with *magA*(K1) and *wzy*(*K2*) gene along with virulent gene rmpA by multiplex PCR. In 54 strains nearly 32 and 8 were K1and K2 positive respectively and 24 strains (44%) were positive for rmpA virulent gene , 18 and 6 in K1 and K2 positive serotypes respectively. Fourteen were negative for K1, K2 and rmpA. High prevalence of hvKP strains were detected from respiratory samples.

**Conclusion:** The knowledge of hypervirulent strains hvKP has to be propagated among the clinicians by the microbiologist and insist the need of stringent approach towards antibiotic stewardship.

## ISSHID Abstract-127 *blaKPC, blaNDM*, *blaVIM* detection among the Carbapenem resistant organisms (CROs) isolated in the Intensive care unit

### S.H.ShifaMeharaj^1^,S.Jayanthi^1^, D.Danisvijay^1^, A.Sujhithra^2^, J.Perumal^1^

#### ^1^Department of Microbiology, CHRI-Chettinad Hospital and Research Institute,CARE- Chettinad Academy of Research & Education, Kelambakkam, Tamilnadu, India; ^2^Department of Allied Health Science, Chettinad Academy of Research & Education, Kelambakkam, Tamilnadu, India

**Background:** Infections with Carbapenem resistant organisms (CROs) are known leading causes of mortality and morbidity among the critically ill Intensive care unit [ICU] patients. Overuse of the antimicrobials, prolonged stay and use of invasive devices are the most important factors. Objective of this study genotypic confirmation of carbapenemase encoding genes among CROs in the ICU.

**Methods:** The study was conducted in the Microbiology Department of Chettinad Hospital and Research Institute, Kelambakkam, Chennai, period of five months and ICU clinical samples (n=696) were processed as per standard guidelines. Phenotypic test like E-strip test, mCIM and Carba NP were performed. Multiplex PCR was performed to confirm the presence of blaKPC, blaNDM and blaVIM genes in CRO.

**Results:** Pathogens were grown in 189 ICU samples. Nearly, 68 isolates showed carbapenem resistance by antimicrobial susceptibility testing. Carbapenemase enzyme was detected in 46 and 50 strains by Carba NP and mCIM test respectively. Multiplex PCR was performed in 68 CRO isolates to amplify the specific genes. Among the CROs, 6 blaVIM, 13 blaKPC and 17 blaNDM positive and others n=32 were negative. *Klebsiella pneumoniae* was the predominant strain isolated in ICU with blaKPC (n=9,25%).

**Conclusion:** Effective and rapid molecular detection of the resistant strains directly from the ICU samples may prevent treatment failure and spread of nosocomial strains.

## ISSHID Abstract-128 Tuberculous pericarditis associated with leprosy: A rare combination

### Rani S, Prasannakumar, Jaisri A, Jaikhishen M

#### Department of Laboratory medicine, Southern Railway Head Quarters Hospital, Ayanavaram, Chennai , Tamilnadu, India

**Introduction:** Pericarditis is a rare manifestation of tuberculous disease that can be fatal even with proper diagnosis and treatment. Tuberculosis is a relatively common cause of clinically significant pericarditis in immunocompromised patients. We report a case of asymptomatic tuberculous pericarditis in a leprosy patient, an association that is rarely reported at present.

**Case Report:** A 59 year old male recently diagnosed with borderline tuberculoid leprosy, presented with fever and severe pain over left knee with pus discharge (Brodie’s abscess).During admission, chest X-ray showed cardiomegaly and patient had abnormal ECG findings. An Echocardiogram performed showed massive pericardial effusion and emergency pericardiocentesis done.

Gram stain of the aspirated pericardial fluid showed no organism and AFB smear was negative. Bacterial culture showed no growth and PCR Assay for Mycobacterium tuberculosis was positive. Culture on Lowenstein Jensen medium showed rough, tough and buff colonies suggestive of Mycobacterium tuberculosis at the end of 4 weeks and was confirmed by BACTEC MGIT 960 Analyzer. Sputum AFB smear was negative. ESR, LDH and ADA levels were increased. Cytology report showed predominantly lymphocytes. Based on laboratory investigations patient was started on ATT (Category 1 RNTCP), and improved clinically. Consent was obtained from the patient for the publication of his data and there was no conflict of interest during the case study.

**Conclusion:** Tuberculous pericarditis has a wide range of disease and may lead to cardiac tamponade. Pericardial tissue specimens should be obtained to provide definitive laboratory diagnosis. Culture remains the gold standard for laboratory confirmation of tuberculous pericarditis, and to perform drug-susceptibility testing and genotyping. This case highlights the importance of TB screening in patients with Leprosy

## ISSHID Abstract-131 Prevalence of fungal infections in a tertiary care hospital of South India

### Sinduja.S, Anupma Jyoti Kindo

#### Department of Microbiology, Sri Ramachandra Institute of Higher Education and Research, Chennai, India

**Background:** Fungal infections are the biggest evolving challenge in recent health care setting. Detection of fungi has exponentially increased leading to identification of vast range of fungi posing threat. Hence, understanding the prevalence of fungi will help us in getting more insight into approach to tackling various fungal disease with appropriate treatment. Newer diagnostics and therapeutics - identification of immune related diseases and various drugs being used as immunomodulators. Hence this study will help us in understanding the present scenario.

**Methods:** 664 samples from suspected fungal diseases were taken for the study received during the period of August 2018 - 19. Conventional mycological methods were performed that included direct microscopy, Gram staining and KOH mount, culture on Sabouraud’s dextrose agar, Oat meal agar and special media if required. Fungi were speciated by studying the morphology.

**Results:** Male predominance with identification of fungi from samples in decreasing order are Urine 325 (49%), Pus swab 132 (20%), BAL 86 (13%), Blood 86 (13%), Others 33 (5%). Candida tropicalis was the predominant isolate followed by candida albicans, Non albicans (other than tropicalis) , Aspergillus and newer pathogenic fungi.

**Conclusion:** Increasing incidence of fungal infections with isolation of newer pathogenic fungi in both immunocomprised and competent individuals needs prompt attention worldwide. Hence, Understanding the recent progression of fungi world would pave way for better outcomes.

## ISSHID Abstract-134 A severe gluteal infection caused by Apophysomyces species- a case report

### Harish M, Saravanan, Vidya S, Anupma Jyoti Kindo

#### Sri Ramachandra Institute of Higher Education and Research, Chennai, India

**Background:** Apophysomyces is a filamentous fungus occurring in soil and decaying vegetation as a common environmental contaminant. It is commonly found in tropical and subtropical regions. It causes infection in immunocompromised and healthy individuals as well.

Case Report: A 69-year-old male patient who is a known case of diabetes, was rushed to the emergency for management of acute kidney injury and right sided gluteal ulcer from an outside hospital. Patient had history of IM injection, following which he developed blackish discoloration of skin around the right gluteal area for which incision and debridement was done outside. On examining, the post debridement wound had unhealthy and necrosed ulcer involving the deeper muscles. There was white cotton like growth (fungal) all around the ulcer. The necrotic tissue and the cotton like growth were sent for gram stain, KOH mount, fungal culture, bacterial culture and histopathological examination for identification of the pathogen. Patient was started on Liposomal Amphotericin B and emergency wound debridement was done. Post-operative wound persistently had fungal growth and myonecrosis. Fungal culture revealed the presence of apophysomyces species and histopathology of tissue confirmed the angioinvasion. Tissue culture also had *Enterococcus faecalis* susceptible only to linezolid and vancomycin. Consent was obtained from the patient for publication of their personal data.

**Conclusion:** In patients with diabetes, mucormycosis is very common more so in uncontrolled diabetes and an inconspicuous wound can turn out to be infected with the member of the zygomycetes family. In advanced cases like this amphotericin B also becomes ineffective and patient can succumb to the infection due to its angioinvasive nature.

## ISSHID Abstract-137 In vitro neutralization of TGF-β in the cervical epithelial cells enhances HIV-1 release

### Jyotsna Gokavi, Shubhangi Bichare, Madhuri Thakar, Vandana Saxena

#### Immunology & Serology Division, ICMR-National AIDS Research Institute, Pune, Maharashtra, India

**Background:** Among females, the local immune responses like increased inflammation and proinflammatory cytokines at cervicovaginal mucosa (CVM) potentiates the risk of HIV acquisition and disease progression. Transforming growth factor-β (TGF-β) is an important cytokine which is shown to be associated with increased cervical viral shedding, however, the causal relationship is yet unknown. Hence we aimed to determine the effect of TGF-β on HIV shedding at CVM using in vitro assays.

**Methods:** Two cervical epithelial cell lines were used: ME-180 (lacking CD4 receptor) and TZM-bl (transgenic expressing CD4 and CCR5). For HIV infection, TZM-bl cells were infected with cell-free HIV (NL4.3) while ME-180 cells were co-cultured with HIV-infected T cells. At different intervals, levels of TGF-β and HIV-p24 were determined using qPCR and ELISA.

**Results:** ME-180 cells showed a significant increase in HIV release from day 3 onwards. Interestingly, TGF-β mRNA was significantly reduced in HIV infected ME-180 co-cultures than uninfected co-cultures. TZM-bl cells showed a significant increase in p24 levels by day 1 pi and also showed reduced expression of TGF-β gene in HIV-infected cells than uninfected cells. After blocking TGF-β using neutralizing antibodies, there was an increase in extracellular HIV-p24 level in ME-180 cells while neutralizing the TGF-β levels did not influence HIV-p24 release in TZM-bl cells.

**Conclusion:** These findings indicate HIV infection suppresses TGF-β expression in cervical epithelial cells and neutralizing TGF-β facilitates HIV shedding. It would be further interesting to understand the interplay of other immune cells at these mucosal surfaces in influencing the viral shedding.

## ISSHID Abstract-138 Occurrence of *Candida albicans* and Candida non-albicans in critical care unit patients and their antifungal susceptibility pattern in a tertiary care hospital, Mysore, Karnataka

### Swathi K, Sumana MN, Keerthi SK

#### Department of Microbiology, JSS Hospital, JSS Academy of Higher Education and Research, Mysore, Karnataka, India

**Background:** Candida species being a part of normal commensal flora, is capable of causing severe infections in human in both immunocompromised and immunocompetent patients. The prevalence of C. albicans and Non-albicans Candida (NAC) in critically ill patients and their treatment management along with emergence of antifungal resistance is a major challenge to the primary care providers.

**Methods:** This study was conducted for a period of 18 months. The clinical samples were collected from ICU patients and were cultured onto Sabouraud Dextrose Agar for isolation. Species identification and their antifungal susceptibility testing were carried out by the automated system, VITEK2 Compact.

**Results:** Non-albicans Candida (79.48%) were predominantly isolated than *Candida albicans* (20.51%). Among the NAC spp*, C. tropicalis* (68.98%) were significantly isolated followed by *C*. *parapsilosis* (6.50%); *C. glabrata* (5.42%); *C. guillerimondii* (4.55%); *C.famata* (2.81%); *C. krusei* (2.16%); *C. haemulonii* (1.73%); *C. ciferrii* (1.08%), *C. lusitaniae* (1.08%), *C. rugosa* (1.08%), *C*. *utilis* (1.08%); *C. lipolytica* (0.86%). Rate of occurrence of Candida spp. in blood sample is 2.08%, 9.97% in urine samples, 2.64% in Sputum samples, 1.91% in Endotracheal aspirates. Occurrence of Candida spp. were high in Surgical ICU (14.48%) followed by ICU (11.89%), Medical Care Unit (7.06%), Respiratory ICU (5.51%), Neonatal ICU (3.96%). *C. tropicalis* showed drug resistance to Flucytosine (6.28%), Fluconazole (18.55%), and Amphotericin-B (8%). C. albicans showed a higher drug resistance to Micafungin and Voriconazole than NAC spp.

**Conclusion:** Substantial increase of Non-albicans Candida in critical care units and their antifungal resistance can be managed by prompt detection, rational usage of antifungal drugs.

## ISSHID Abstract-139 Resistance and virulence profile of Coagulase negative Staphylococci isolated from Genital Tract Infections from Chennai, South India

### Anbarasi Kalaiselvan^1^, Esther Mary Selvam^2^, Padma Krishnan^1^

#### ^1^Department of Microbiology, Dr. ALM PG IBMS University of Madras, Chennai, Tamil Nadu, India; ^2^Department of Microbiology, ESIC Hospital, K.K. Nagar, Chennai, Tamil Nadu, India

**Background:** Coagulase Negative Staphylococci (CoNS) are the commensal bacteria of the urogenital region, a reason why CoNS are usually considered contaminant in genital samples. However, repeated isolation of the same tube coagulase negative staphylococci with significant colony count (>10^5^cfu/ml) from a symptomatic case is regarded a pathogen. Significance of CoNS as a pathogen in genital infections is due to the fact that it serves as a reservoir of virulence and antibiotic resistant genes which could facilitate transfer to more pathogenic strains of *Staphylococcus aureus*, thereby causing persistent infection and possible treatment failure. Thus, this work was designed to study the resistance and virulence profile of CoNS from genital infections.

**Methods:** 51 CoNS isolates obtained from a tertiary care centre in Chennai were included (Source-High vaginal swab-n=27, Endocervical swab-n=1, semen-n=16). Antibiotic susceptibility testing was followed by detection of resistance determinants ßlactams[mecA], macrolides [ermA,ermC,msrA], tetracyclines[tetK,tetM], aminoglycosides[aac(6′)-Ie-aph(2″),ant(4′)-Ia,aph(3′)-IIIa], trimethoprim [dfrA], mupirocin[mupA], fusidic acid[fusB,fusC,fusD] by PCR. Biofilm formation was phenotypically detected by tissue culture plate method (TCPM) followed by screening virulence genes-icaAD,aap,atlE,eno,ebpS,cna,bbp,IS256.

**Results:** Of the 51 CoNS isolates, *S.haemolyticus* (n=42,82.4%) was predominant followed by *S.epidermidis* (n=6,11.8%), *S.warneri* (n=2,3.9%)and *S.capitis* (n=1,1.9%) respectively. 31(60.7%) isolates were methicillin resistant. Highest resistance was found towards erythromycin (64.7%) followed by pristinamycin (50.9%) and ciprofloxacin (47%). Among the resistance determinants dfrA(41.2%) was predominant followed by tetK(27.5%), aac(6′)-Ie-aph(2″)(21.6%),aph(3′)-IIIa(21.6%), fusC(15.7%), fusB(11.8%), ermC(9.8%), mupA(7.8%),msrA(3.9%),tetM(1.9%) respectively. By TCPM, 1(1.9%) isolate showed strong and 15(29.4%) weak biofilm production. Overall, 15(29.4%) isolates harboured biofilm genes(1icaAD+aap+atlE,9aap,5aap+atlE). Amongst the adhesion genes eno (88.2%) was predominant followed by ebpS(35.3%) and cna(1.9%). IS256 was present in 28(54.9%) isolates.

**Conclusion**: The present study demonstrates high prevalence of resistance and virulence determinants among CoNS isolates. The study results highlight the pathogenic significance of CoNS from genital infections

## ISSHID Abstract-140 Resurgence of ‘The Great Imitator’- A Case Series of Secondary Syphilis

### Ibrahim Yahya, Anuradha Priyadarshini, Mahalakshmi V, Murugan S

#### Department of Dermatology, Venereology and Leprosy, SRIHER (DU), Chennai- 600116, India

**Background:** Syphilis is a STI caused by Treponema pallidum. Signs and symptoms can be variable through its 3 clinical stages. Syphilis has been known as “The Great Imitator"" as it can mimic many other diseases. Syphilis infection has a great impact on Indian population and therefore a better understanding of trends in its prevalence could help improve the efforts of public health measures in STI prevention and their integration with HIV prevention.

**Methods:** Case series of 11 cases of early infectious syphilis in a 1-year time period.

**Results:** Among the 11 cases presented, 7 of them were young males (1 boy), 3 were young females and 1 a transgender. Of the 3 females, 2 were pregnant. All patients presented with features of secondary syphilis; confirmed with reactive VDRL and positive TPHA. None of them were HIV positive. In this series of secondary syphilis cases, an early infectious stage of syphilis is a clear pointer towards resurgence of syphilis in recent times.

**Conclusion:** Syphilis, which was previously endemic in low income countries, has now re-emerged in several high-income countries. From a dermatological perspective- a suspicious mind, a detailed history, along with careful clinical examination by an astute clinician is required to make the diagnosis of syphilis. Secondary syphilis with its relatively innocuous presentations can be difficult to identify and if misdiagnosed or left undiagnosed leads to detrimental consequences in individual and community. The role of preventive measures to reduce prevalence of syphilis in pregnancy and in young cannot be overemphasised.

## ISSHID Abstract-141 A case of stubborn Cryptococcal meningitis in an immunocompetent patient

### P V Anto, N Maheedhar Reddy, Vidya Krishnan, Anupma Jyoti Kindo

#### Department of Microbiology, Sri Ramachandra Institute of Higher Education and Research, Chennai, India

**Background:** Cryptococcus is an opportunistic fungus, an important cause of CNS infections among immunocompromised patients, but it has only sporadically been reported in non-HIV positive persons.

**Materials and Method:** A 64-year-old female was brought to emergency with complaints of fever, severe head ache, vomiting with known case of uncontrolled diabetes mellitus. Blood and urine cultures were negative in early stage. Due to the CNS symptoms meningitis was suspected and CSF was collected. India ink preparation showed plenty of capsulated yeast cells. Cryptococcal latex agglutination test titre was more than 1:64. Culture was positive and nucleotide sequencing confirmed as Cryptococcus neoformans var grubii. The diagnosis was confirmed as cryptococcal meningitis. Patient was started on conventional Amphotericin B. The patient underwent normal serial lumbar punctures for control of intracranial pressure. MRI showed leptomeningeal enhancement with new onset of non-obstructive hydrocephalus. Consent was obtained from the patient for the publication of her data.

**Result:** After 13 days of antifungal treatment, India ink continued to show distorted cryptococcal yeast cells. The cryptococcal latex agglutination test titre remained the same as more than 1:64. However at this stage the culture was negative for Cryptococcus. In spite of the treatment patient succumbed to the infection.

**Conclusion:** This is an unusual case of non-responding cryptococcal infection despite the amphotericin B treatment. The CSF provides a rich medium to promote fungal growth and the local production of mannitol by the fungus may contribute to cerebral edema and interfere with phagocytic function. Combination therapy with fluconazole could have been tried, since fluconazole has a good penetration through the blood brain barrier.

## ISSHID Abstract-142 Study of Rubella infection Seropositivity in Antenatal Women attending obstetric clinic of Secondary and Tertiary care hospital in Perambalur

### Maheswari R, Suresh Chander

#### Microbiology Department, Dhanalakshmi Srinivasan Medical College & Hospital, Tamilnadu Dr MGR Medical University, Perambalur, India

**Background:** Rubella, is an acute, mild exanthematous illness caused by Rubella virus which belongs to family Togaviridae. Maternal infection during pregnancy causes great concern of public health importance, because it acts as a teratogen leading to frequent abortions, intra – uterine fetal deaths, still births and congenital rubella syndrome (CRS). Even though safe and effective vaccine against rubella is available, according to estimation over 100,000 infants are born with congenital rubella syndrome worldwide annually. Rubella infection is endemic in India, morbidity and mortality among live births is contributed mainly by CRS.

**Aim:** To study the Seropositivity of Rubella IgM and IgG antibodies in antenatal mothers attending the obstetric care clinic.

**Methods:** Cross sectional study conducted for six months from March 2018 to September 2018. Sera collected from 193 antenatal mothers irrespective of the trimesters belonging to 20 – 40 years of age. According to manufacturer’s instructions Rubella IgM and IgG antibodies were assessed by ELISA using Ratio – Diagnostics kits.

**Results:** 3.1% Seropositivity of IgM and 75.1% for IgG antibodies was observed and maximum Seropositivity seen among mothers of age 25-30 years.

**Conclusion:** All antenatal cases should be routinely screened for Rubella. Serosurveillance of Rubella should be considered among adolescent girls and women of child – bearing age before conception to avoid further delay in assessment during pregnancy, to prevent innumerable abortions, still births and CRS.Strong recommendations can be made for universal coverage of MR vaccine in early age group of children.

## ISSHID Abstract-147 Preliminary investigation on the effect of vanillic acid on preventing the pathogenesis of *Enterococcus faecalis* (ATCC 29212)

### Sherly Antony^1^, Vineetha Varghese^2^, Aswathi Biju^2^, Ceyona Chacko^2^, Prasanth Rathinam^3^

#### ^1^Department of Microbiology and Microbial Technology Laboratory, Pushpagiri Institute of Medical Sciences and Research Centre, Thiruvalla, Kerala, India; ^2^Department of Medical Microbiology, Union Christian College, Aluva, Kerala, India; ^3^Department of Biochemistry and Medical biotechnology Laboratory, Pushpagiri Institute of Medical Sciences and Research Centre, Thiruvalla, Kerala, India

**Background:** Dental plaque is a microbial biofilm (usually *Enterococcus faecalis*) that attaches to tooth surfaces, restorations and prosthetic appliances like dentures and bridges. Frequent build-up of biofilms can give rise to tooth decay, and periodontal problems like gingivitis and periodontitis. Understanding this need of dental plaque removal from the oral cavity, this study is an attempt to investigate the effect of Vanillic acid (plant derived flavouring agent) against biofilm formation and virulence factor production by E. faecalis.

**Methods:** The minimum inhibitory concentration (MIC) of Vanillic acid against *E. faecalis* (ATCC 29212) was determined as per standard CLSI guidelines and all further assays were performed at sub-MIC levels. Effect of Vanillic acid on the expression levels of virulence factor genes and cell to cell communication mechanism (quorum sensing) associated genes were also studied and statistically analysed using GraphPad Prism 5 Sofware.

**Results:** The MIC of Vanillic acid for *E. faecalis* (ATCC 29212) was 1024 μg ml−1 (6.0 mM). Vanillic acid tested at sub-MIC levels (3.0, 4.0 and 5.0 mM) exhibited biofilm inhibition and biofilm eradication properties, significantly at 3.0 mM levels (p<0.001). Similarly, among the tested levels of Vanillic acid, 3.0 mM (p<0.001) significantly reduced the mRNA levels of virulence factor determinants; GelE (encoding gelatinase), Esp (encoding enterococcal surface protein), Agg (encoding aggregation substance) and cell to cell communication mechanism (quorum sensing) associated genes FrsB and LuxS (p<0.001).

**Conclusion:** The present study confirmed the biofilm inhibition and anti-virulence property of sub-MIC levels of Vanillic acid against *E*. *faecalis* plausibly through QS inhibition (QSI) mechanism.

## ISSHID Abstract-148 Detection of Aspartyl Proteinases and Esterase activities of *Candida auris* isolated at a tertiary care hospital in Karnataka, South India

### Umamaheshwari S^1^, Shubha Gopal^1^, Keerthi SK^3^, Sumana MN^2^

#### ^1^Department of Studies in Microbiology, University of Mysore, Mysuru, Karnataka, India; ^2^Department of Microbiology, JSS Medical College, Mysuru, Karnataka, India

**Background:***Candida auris*, an emerging yeast has associated globally with life threatening invasive infections and has become the major concern for healthcare and scientific community due to multidrug-resistant profiles. The aim of this study was to determine the virulence of *C.auris* isolates by assaying in vitro secreted aspartyl proteinases (Saps) and esterase activities.

**Methods:** Twelve *C.auris* strains (10-clinical, 2-environmental) isolated during Dec2018–March2019, in tertiary care hospital in Mysuru, Karnataka, India, were included for the study. Saps and esterase activities were assessed by plate assays using yeast carbon base-bovine serum albumin (BSA) and Tween 80 opacity medium respectively. About 10μl of C.auris (107 CFU/ml) suspension were inoculated on both the medium and incubated at 37°C for 72hrs.

Cleavage of BSA by Saps resulted in a clearance zone for proteinase activity, whereas hydrolysis of the medium by lipolytic enzymes showed precipitation halo zone for esterase test. This activity is expressed according to the Pz index, i.e., ratio of colony diameter to the total diameter of the colony plus the clearance zone/precipitation halo. Pz score of one was considered negative, <1 as positive, and ≤0.63 as strong positive.

**Results:** All 12 *C.auris* strains analyzed in the study were found to possess extracellular proteolytic and esterase activity. Pz score of all C.auris were ≤0.63, indicating strong Saps and esterase producer.

**Conclusion:***C.auris* being the first yeast to be highly virulent and multidrug-resistant, necessitates to identify virulence factors and understand the molecular mechanism of pathogenesis to introduce new anticandidal agents and improve treatment strategies

## ISSHID Abstract-149 An unusual case of fatal colitis in HIV patient

### Sreya Alice Jacob, Sudha Madhavan, K.Vengadakrishnan

#### Department of Medicine, SRIHER , Chennai , India

Introduction: AIDS is an immunocompromised state highly susceptible to opportunistic infections. Diarrhea can cause significant morbidity in HIV patients and cryptosporidium accounts upto a third of diarrhea cases. Cryptosporidium is a protozoa belonging to the class apicomplexa and known to cause chronic, severe debililating diarrhea in immunocomprimsed individuals.Common species infecting humans include C.parvum and C.hominis.

Case report: A 60 year old male patient presented to the hospital with complaints of diarrhea of six months duration. Four days before admission his symptoms aggravated with associated vomiting and intermittent low grade fever. The patient was diagnosed to be HIV reactive 15 days ago with CD4 count of 43 while being evaluated for significant weight loss and was started on HAART. Clinical examination revealed pallor and chronic multiple violaceous plaques all over the body. Platelet count was 3000/cmm and peripheral smear showed macrocytic RBC, neutrophilia and thrombocytopenia. Stool AFB was negative for isospora, cyclospora and cryptospora. CT abdomen showed moderate ascites and possibility of infective colitis. Upper GI endoscopy showed diffuse erythema, biopsy of which revealed cryptosporidium. CMV PCR was negative. Colonoscopy revealed diffuse colitis. Bone marrow biopsy was suggestive of hypocellular marrow. During the course of stay the patient had malena, hematochezia, sudden drop of hemoglobin, repeated hypoglycaemia and metabolic acidosis. The patient was treated with HAART and Nitazoxanide.

Consent from next of kin of patient was obtained for the publication of patient’s data, as patient expired during course of treatment.

**Conclusion:** Cryptosporidiosis presenting with symptoms of fulminant colitis is rare but has a very high mortality rate.Immunocompromised individuals presenting with chronic diarrhea and colitis symptoms should be evaluated for cryptosporidiosis.

## ISSHID Abstract-150 Decline in the prevalence of HIV infection among clients attending Integrated counseling and testing center at a tertiary care center: Trend over the period of 14 years

### Sangita Bansode, Preeti Sangani, Varsha Pancholi, Prashant Javade

#### Navi Mumbai Municipal Corporation Hospital, Vashi, Mumbai, India

**Background:** HIV integrated counseling and testing centers play an important role in evaluating HIV/AIDS epidemic. We assessed changes in HIV prevalence over last 14 years in a tertiary hospital in Maharashtra highlighting the socio-demographic characteristics & risk behavior of clients.

**Methods:** This was a retrospective observational study done from 2004-2018. In all 1,26,649 clients including regular and high risk groups, excluding antenatal patients, either visited voluntarily or as referred were screened as per NACO guidelines. The results were analyzed and correlated with sex, age, occupation and route of transmission.

**Results:** A steady increase in occurrence of HIV positivity from 2005 to 2009 followed by steady decline from 2010 to 2018 (10.34% to 2.72%) was observed. Male:Female ratio was 1.34:1. Highest prevalence was observed amongst 35-49 years in both sexes (39.52%) and most common route of transmission was among heterosexuals (94.45%). In males majority had petty business (21.43%) where as in females, majority were housewives (30.61%) who were the partners of affected males. A significant decline in dropouts after pre-test counseling was noticed (17.39% to 0.24%). Despite lower proportion of direct walk-in ICTC attendees (14%) compared to referred, seroprevalence was noticed significantly higher amongst direct walk-in (23.98%) than the referred (2.3%).

**Conclusion:** Integrated counseling and testing centers (ICTC) are key entry points for HIV detection and prevention. Overall the study highlighted an increase in number of cases for HIV screening and simultaneous steady decline in HIV seroprevalence over more than a decade due to intensified and consolidated interventional services by NACO.

## ISSHID Abstract-151 Draft genome sequencing data of Blastocystis subtype 3 human isolate from xenic culture

### Shashiraja Padukone^1^, Amitbikram Mohapatra^2^, Abhishek K Shrivastav^2^, Jharna Mandal^1^, Ajay P Selvarathinam^1^, Nonika Rajkumari^1^, Vamsi M Ananthabotla^1^, Subhash Chandra Parija^1,3^

#### ^1^Department of Microbiology, Jawaharlal Institute of Postgraduate Medical Education and Research, Puducherry, India; ^2^Division of Bioinformatics, Bioserve Biotechnologies, Hyderabad, India; ^3^Sri Balaji Vidyapeeth, Pillayarkuppam, Puducherry, India

**Background:** Blastocystis an atypical stramenopile dwells in the large intestine of approximately one billion people across the globe and nine subtypes (STs) of Blastocystis (ST1 to ST9) infect humans. Blastocystis pathogenicity status is unclear. The previous study from Puducherry, India confirmed that Blastocystis ST3 is the most prevalent subtype. Here we explored the likelihood of retrieving the draft genome of Blastocystis ST3 grown in xenic culture.

**Methods:** It is an institutional ethics committee approved study protocol and informed consent was obtained from the participant. In this experiment, Blastocystis subtype 3 isolate was cultured in Jones’ medium from the stool sample of an asymptomatic healthy individual. Blastocystis cells were enriched by density gradient centrifugation and subjected to DNA extraction. Further, paired-end libraries were prepared using the NEBNext Ultra DNA II kit using the standard protocol and sequencing was performed on Illumina Nextseq 500 platform. More than 87% of the data passed the Q30 quality score. High-quality reads were mapped to the reference genome of Blastocystis ST7 CABX0100000.

**Results:** Upon running through contaminant screen a total of 10,429,904 sequence length with 10,257 contigs were recovered. The GC content was 52% and the processed reads were deposited in GenBank under the accession number QVNP00000000. (https://www.ncbi.nlm.nih.gov/nuccore/QVNP00000000.1) However, gene and protein annotation were not performed.

**Conclusion:** This is the first draft genome sequence and reference assembly of Blastocystis ST3 from India. It provides additional insights into the genome of Blastocystis ST3. Subsequent, gene annotation is necessary to unravel putative pathogenic genes.

## ISSHID Abstract-156 Autoantibodies among HIV-1 infected individuals and the effect of antiretroviral therapy on it

### Runal J Steve^1^, Diviya Alex^1^, Binesh Lal Y^2^, John A Jude Prakash^2^, Nitty S Mathews^3^, Veena Vadhini R^1^, John P Demosthenes^1^, Grace Rebekah J^4^, George M Varghese^5^, Rajesh Kannangai^1^

#### ^1^Department of Clinical Virology, Christian Medical College, Vellore, Tamil Nadu, India; ^2^Department of Clinical Microbiology, Christian Medical College, Vellore, Tamil Nadu, India; ^3^Department of Transfusion Medicine and Immunohaematology, Christian Medical College, Vellore, Tamil Nadu, India; ^4^Department of Biostatistics, Christian Medical College, Vellore, Tamil Nadu, India; ^5^Department of Infectious Diseases, Christian Medical College, Vellore, Tamil Nadu, India

**Background:** Antiretroviral therapy (ART) has led to a decline in autoimmune diseases but lacks studies on its effect on autoantibodies. This study aims to address the effect of ART on autoantibodies among HIV-1 individuals.

**Methods:** This is a cross sectional study with 100 paired HIV-1 infected ART naïve and experienced individuals and 100 age, sex matched healthy controls. Antinuclear antibody (ANA) detection by immunofluorescence assay (IFA), IgG anticardiolipin antibody (aCL), IgM and IgG beta 2 glycoprotein 1 (β2 GP1) antibodies by ELISA were performed. Results are expressed as median and percentage positivity, p value <0.001 is considered significant. The study was approved by the Institutional Review Board (IRB No 11129 dated 24.01.2018).

**Results:** In this study, 61 were males and 39 females. The median viral load of the treatment naïve samples was 4.34 Log copies/mL and median duration of treatment was 12 months. The percentage positivity of ANA was 5% among ART naïve and controls with a decrease to 2% post ART (p= 0.441). The positivity for aCL antibody was 15% among ART naïve with no positivity among treated or controls (p <0.001). IgM β2 GP1 was 4%, 1% and 3% among ART naïve, treated and controls respectively (p= 0.821). IgG β2 GP1 was 2% among ART naïve while none of the treated and controls were positive (p= 0.505).

**Conclusion:** ART has a significant effect on IgG aCL antibody and some effect on ANA, β2 GP1 IgM and IgG antibodies as evidenced by a decrease in percentage positivity following ART.

## ISSHID Abstract-157 Mucormycosis of gastrointestinal tract masquerading as gastroduodenal malignancy: Two case reports

### Supradeep.N, Shanmugam.D, Kadambari.D, Raj Kumar. N, Bhavana.S. Bhade

#### Jawaharlal Institute of Post-graduate Medical Education and Research (JIPMER)

**Introduction:** Mucormycosis is a rare, but fatal infection caused by angioinvasive fungi belonging to the order Mucorales. Mucormycosis of gastrointestinal tract is rare accounting for less than 7% of cases.

**Case 1:** A middle aged male, smoker and alcoholic, presented with several years’ history of epigastric pain and recent onset hematemesis and melena, associated with weight loss. He was poorly nourished, pale and had epigastric tenderness. Endoscopy revealed ulceroproliferative lesion in the first part of duodenum, biopsy from which had broad, ribbon-like, aseptate fungal hyphae consistent with zygomycosis. He underwent pancreaticoduodenectomy. Post-operatively he received amphotericin B but succumbed to organ failure after 47 days.

**Case 2**: An elderly male, smoker and alcoholic, presented with complaints of epigastric pain for 2-months, vomiting for 3-days and was tolerating only liquids at presentation. He gave history of loss of weight and appetite. Endoscopy showed an excavating ulcer in stomach and duodenum, histopathology revealing mucormycosis. During hospital stay he had massive hematemesis and was taken up for surgery where distal gastrectomy and cholecystojejunostomy was performed for gastric perforation along with complete transection of duodenum and sloughed off gallbladder. He was treated with lipoidal amphotericin B. He succumbed on post-operative day 2. Consent has been taken from the next of kin of both the deceased patient for publication of their personal data.

**Conclusion:** Though rare, duodenal mucormycosis is a fatal disease with high mortality rate and has very few case reports in literature. Our case series brings to notice this rare, fatal infection and discuss its presentation, diagnosis dilemma brought due to its non-specific symptoms, treatment aspect and the complications of treatment

## ISSHID Abstract-159 Magnitude of HIV infection among fifty plus years age group

### Sharvani.R, Hemavathi, Adnan Rafiullah

#### Microbiology Department, Sapthagiri Institute of Medical Sciences and Research Centre (RGUHS), Bangalore, India

**Background:** In a developing country like India the suspicion and screening for HIV is overlooked in 50 years and above age group population keeping in mind the notion that they are not sexually proactive. The present study is taken up to assess the magnitude of HIV infection among the population aged 50 years and above and also to assess their level of awareness towards safe sex practices and its importance.

**Materials and Method:** All referrals to PPP-ICTC who are 50years and above age group, from January 2014- December 2017 were analyzed.

**Results:** Out of 12558 referrals in this age group, 102 seropositive cases (64 males+38 females) were noted. The magnitude of positivity was 0.51 %( 2014), 0.81 %( 2015), 0.66 %( 2016), 1.2 %( 2017). 99% of transmission was sexual mode.69% were married and living with spouse, 27% single(widow/widower),3% separated not divorced,1% death .The spouse status showed 29% concordance , 40% discordance and unknown in 31%. Education level nil in 76.3% female and 43.7% male. None followed safe sex practices.

**Conclusion:** The study highlights that people aged 50 years and above are at equal risk for HIV infection as sex continues to play a very important role. As there are very few studies targetting the above age group there is paucity of data to understand age related changes in behaviour ,attitude etc to guide policy makers to reframe the strategies to avoid new infection and late diagnosis and also to subsume above age group into surveillance system.

## ISSHID Abstract-160 Incidence of blood groups as a predisposing factor for human HIV infections

### Deepika.L, Devi.G, Kalaivani.V, Elanchezhiyan.Manickan

#### Department of Microbiology, University of Madras, Taramani campus, Chennai, India

**Background:** Human Immunodeficiency Virus (HIV) which causes AIDS is the most devastating disease which is continued to be challenging for the management.Even though there are several modes of transmission have been repeatedly documented there are certain individuals who do not acquire the disease (natural resistance) or they show only minimum symptoms (elite controllers). Besides these, there are many other unknown mechanisms prevents the spread of HIV. In this study, we evaluated various blood groups and characterized the particular blood groups which may prone more for HIV infection.

**Methods:** For this study, we enrolled 300 healthy control and 300 HIV patients from various ART centers in and around Chennai. Blood samples were collected from each patient and as well as from control in anticoagulant Vacutainer.

HIV testing was done by using HIV gag p24 ELISA. Blood grouping were done by using standard protocols. Data was analyzed statistically by student’s t-test.

**Results:** In this study,when we analyzed the blood group that is more susceptible to HIV infection we found that blood group B was more prone to get HIV infection. Among the 300 HIV positive individuals, Blood group B was 49.6 ± 1.2, while when we compared the same among healthy controls (n=300), it was only 28.6 ± 1.2 and these values are statistically significant (student’s t-test, p<0.001). Similar comparison were done with A,O and AB blood group, the values were 9.6 ± 2.4, 35.6 ± 3.3, and 5.0 ± 0.81 for the HIV positive cases and for the Healthy control the values were 20.0 ± 3.7, 47.6 ± 5.4 and 3.6 ± 0.94 respectively. However, these differences were not statistically significant (p>0.5)

**Conclusion:** From this study, we are able to speculate that individuals with blood group B are more prone to HIV infections and may acquire HIV more readily than other Blood group individuals.

## ISSHID Abstract-161 T-cell epitope diversity analysis of *P. falciparum* from global clinical isolates

### Rajendra Mandage, Pragyan Acharya

#### Department of Biochemistry, All India Institute of Medical Sciences, New Delhi, India, 110029

**Background:***P. falciparum* is the causative agent of malaria in Africa, and in Asia. It evades immune response by displaying antigenic variations thereby preventing elimination of malaria. Therefore, malaria elimination can be achieved through an efficacious vaccine. However, vaccines that have undergone clinical trials have demonstrated upto 30-40% efficacy. In addition, the design of any vaccine with a global efficacy must take into consideration epitope variation among different parasite isolates. Hence, we present an extensive antigenic (T-cell epitope) variation analysis of *P. falciparum* to detect natural variation that might be involved in immune evasion.

**Methods:** 600 *P. falciparum* epitopes were retrieved form IEDB database and proteome sequences were downloaded from Genbank to detect conserved and non conserved epitopes across multiple isolates. A catalog of epitope variation was created using multiple sequence alignment (MSA) by mapping epitope sequences against the Plasmodium proteome.

**Results:** We have found extensive epitope variation in previously defined vaccine candidates such as circumsporozoite protein (CSP), Apical membrane antigen 1 (AMA1), MSP1, MSP2 and MSP4 proteins at many positions among different isolates. Notably, more than 40% of epitopes showed a distinct amino acid variation pattern. Using a population genomics approach and in silico methodologies, we have detected the variation in vaccine candidates of phylogenetically diverse P. falciparum clinical strains.

**Conclusion:** The presence of distinct polymorphisms in epitope regions may provide a route to escape from immune response during malaria infection. Therefore, understanding epitope variation/conservation would help in designing a better vaccine candidate to prevent Plasmodium falciparum infection.

## ISSHID Abstract-162 Transcriptional expression of superoxide dismutase genes in *Chlamydia trachomatis*-infected recurrent spontaneous aborters

### Ankita Ray^1^, Priya Prasad^1^, Banashree Das^2,3^, Sangita Rastogi^1^

#### ^1^Microbiology laboratory, ICMR-National Institute of Pathology, Sriramachari Bhawan, Safdarjung hospital campus, New Delhi, India; ^2^Department of Obstetrics & Gynecology, Vardhman Mahavir Medical College & Safdarjung hospital, New Delhi, India

**Background:** Recurrent spontaneous abortion (RSA) has multifactorial etiology and has physiological/psychological burden. *Chlamydia trachomatis* (Ct) is common STI leading to RSA and pathological mechanism behind Ct-associated RSA and oxidative stress (OS) is under investigation. Antioxidant genes such as superoxide dismutases (SOD) are activated to maintain antioxidant levels during pathogenic infection due to production of Reactive Oxygen Species (ROS). Aims were to study mRNA expression of Cu-Zn (SOD1) and MnSOD (SOD2) genes in urine of Ct-infected RSA patients by bioinformatic tools.

**Methods:** Subsequent to hospital ethics committee permission, urine samples were collected from pregnant women with history of RSA for Ct MOMP/plasmid detection by PCR. mRNA expression of SODs was quantitated by real-time PCR in Ct-positive versus uninfected RSA patients(controls). Data was statistically evaluated using Graphpad prism software while tabular text of GO/KEGG pathway was studied by STRING.

**Results:** Gene expression of SOD1 was downregulated (p<0.001) whereas SOD2 was upregulated (p<0.001) in Ct-infected RSA patients as compared to controls with fold-change of 0.5 and 1.4 respectively. GO analysis of SOD1/2 genes showed SOD gene family plays important role in ROS scavenging. STRING database revealed interaction with peroxiredoxin, glutathione peroxidase-1,3,5,7,8 and thioredoxin known to be involved in RSA.

**Conclusion:** Findings revealed lowered expression of SOD1 gene in response to Ct. This study increases our understanding of molecular basis of physiological processes such as removal of superoxide radicals, apoptotic signaling pathways, etc. It is evident that presence of Ct leads to production of ROS-generating OS involved in pathophysiology of gynecological ailment, viz.: RSA.

## ISSHID Abstract-163 A meta analysis on HCV association with autoimmune diseases

### Rahamathulla Syed, Shaik Roqhayya

#### Pathgene Healthcare Pvt Ltd, Tirupathi, Andhra Pradesh, India

**Background:** Hepatitis C Virus (HCV) is a hepatotropic and lymphotropic virus is not only associated liver infection but also with many diseases and syndromes which includes extra hepatic manifestations and auto Immune disorders in particular. HCV infection is responsible for Liver cancer and Cirrhosis which leads to about one million deaths throughout the world and has become a prominent public health problem. The main reasonin failing to produce proper medication against HCV infection is high variability and mutation rate in the genome sequence of HCV, which is responsible for triggering auto immune reactions producing auto antibodies.

**Methods:** This particular study is on the pathogenicity of HCV infection which leads to six types of auto immune disorders namely hematological, endocrine, renal, neurological, rheumatological and dermatological disorders. The study of these auto immune disorders found to be significant as they cause chronic inflammation followed by cancer and Cirrhosis leading to death.

**Results:** The current review on HCV infection with respect to Auto immune disorders is first of its kind for discussion and is more significant. HCV infection not only affects the liver, but also linked to extra hepatic manifestations and auto immune disorders.

**Conclusion:** There is experimental proof demonstration that HCV can undermine the immune system probably leading to the onset of autoimmunity. Autoimmune disorders may be due to dysfunction of both cell and humoral immunity. It is crucial to distinguish true auto immunity from HCV induced or mimicked disease since the significance of early treatment or both.

## ISSHID Abstract-165 Versatile coordination behaviour of metal complexes containing pyrazoline derivative imparts potential antimycobacterial molecule

### J.Joseph^1^, K.Nagashri^2^, C.Justin Dhanaraj^3^, G Boomadevi Janaki^4^

#### ^1^Department of Chemistry, Noorul Islam Centre for Higher Education, Kumaracoil, Tamil Nadu; ^2^Department of Pharmaceutical Chemistry, Manonmaniam Sundaranar University, Tirunelveli, Tamilnadu; ^3^Department of Chemistry, University College of Engineering, Nagercoil, Tamilnadu; ^4^Department of Chemistry, Vel Tech Rangarajan Dr. Sagunthala R&D Institute of Science and Technology, Chennai

**Background:** Tuberculosis is one of the most dreadful disease pushing problem on human health worldwide. The increasing prevalence of primary resistance to the current antibiotics and wide distribution of latent TB infection implies a growing need for new molecule with unique mode of action. Literature resources indicated that the integration of metal ions into the heterocyclic structural molecule offers structural diversity, variable oxidation states and thereby enhancing the efficacy against strains. The objective of this work was focused on the synthesis, characterization and antimycobacterial activity of metal complexes (Cu(II), Ni(II), Co(II) and Zn(II)) with pyrazoline derivative.

**Methods:** The MIC values were measured for metal complexes containing pyrazoline derivative against M. tuberculosis strain H37Rv using MABA method and compared with ethambutol (standard).

**Results:** Among the studied complexes, the Cu(II) complex showed higher inhibitory activity with 6.05 mg/mL whereas other complexes exhibited moderate activity with MIC value in the range 11.5–20 mg/mL, respectively as compared to standard, ethambutol.

**Conclusions:** In conclusion, the antimycobacterial activity of synthesized complexes was studied. The potential activity of metal complexes are may be due to the lipophilic nature of complex facilitates the diffusion into the cell membrane and subsequently induces cell destruction through the intracellular reduction of Cu(II) to Cu(I) species lead to the activation of oxygen which may be disastrous for microbial strain. The observed findings make the synthesised copper complex of pyrazoline derivative as a promising candidate for future investigations in the development of anti-TB drugs

## ISSHID Abstract-166 Community acquired pneumonia , now and then- a tertiary health care facility perspective

### Subrahmanyam DKS, Anandaraman R, Avinash U, Harish BN

#### Jawaharlal Institute of Post-graduate Medical Education and Research (JIPMER), Puducherry, India

**Background:** Community acquired pneumonia is a significant health problem. It is the fourth most common cause of death globally and 2nd most frequent cause for DALYs. Periodic assessment of the disease and understanding of the changing trends is essential for improving care.

**Methods:** This was an observational study conducted in JIPMER during the period from January 2015 - June 2016 in hospitalized patients aged >18 years with a diagnosis of pneumonia. Demographic, clinical, microbiological evaluation, and Urinary Antigen Tests for Streptococcus and Legionella was done. The results were compared with a study done in2004 – 2005 Descriptive statistics was performed where applicable and for significance testing a p<0.05 was taken .

**Results:** Eighty patients were studied. Their mean age was 48.63±14.1 years ,majority in the 40- 59 year age group. 51 patients (63.75%) were having comorbidities, most common was diabetes -24(30%) followed by chronic kidney disease -19(23.75%). Klebsiella was the commonest followed by Streptococcus and E. coli with overall microbiological yield of 45%. Streptococcal urinary antigen test was positive in ten but none by urinary Legionella antigen test. 75% of the *Klebsiella pneumonia* were resistant to Ceftriaxone. Mortality was 23.75%. Salient findings were compared with a previous study for changing trends.

**Conclusion:** Changing trends noted were increase in elderly population and comorbidities and ceftriaxone resistance in Klebsiella. Klebsiella is still the commonest organism in admitted patients and urinary legionella was negative in all.

## ISSHID Abstract-169 Truncated *mbtA* gene based real-time PCR for detection of *Mycobacterium avium* subsp. paratuberculosis

### Abinaya Kaliappan^1^, Bablu kumar^2^, Deepak kumar^3^, Ravi kant Agarwal^4^, Vikramaditya Upmanyu^5^, Gururaj^6^, P Das^2^

#### ^1^Department of Veterinary Microbiology, Indian Veterinary Research Institute, Bareilly, Uttar Pradesh, India; ^2^Division of Biological Products, Indian Veterinary Research Institute, Bareilly, Uttar Pradesh, India; ^3^Division of Veterinary Biotechnology, Indian Veterinary Research Institute, Bareilly, Uttar Pradesh, India; ^4^Division of Livestock Products Technology, Indian Veterinary Research Institute, Bareilly, Uttar Pradesh, India; ^5^Division of Biological Standardization, Indian Veterinary Research Institute, Bareilly, Uttar Pradesh, India; ^6^Animal Health Section, Central Institute for Research on Goats, Makhdum, Uttar Pradesh, India

**Background:** Paratuberculosis or Johne’s disease is a chronic granulomatous enteritis of ruminants, caused by Mycobacterium avium subsp. paratuberculosis (MAP). The pathogen is of zoonotic importance and it is known to cause Crohn’s disease in humans.The uniqueness of MAP is that the inability to produce mycobactin in-vitro due to truncation of mbtA gene by LSPp12 within the mycobactin operon of MAP. The goal of the study was to evaluate the truncated mbtA gene that could serve as an alternate diagnostic marker for specific detection of MAP.

**Methods:** The real-time PCR was optimized involving the truncated mbtA gene of MAP. The sensitivity and specificity of the assay was determined by comparison with standard molecular marker IS900. The test was validated with suspected fecal samples (n=50) and intestinal tissues (n=4) obtained from goat.

**Results:** Amplification results showed only the specific amplicon of MAP reference strains whereas other mycobacterial and non-mycobacterial species were not amplified. The detection sensitivity of truncated mbtA gene was found to be 21.3 fg of MAP DNA. The test detected 12 fecal samples and 1 intestinal tissue positive for MAP. The comparative evaluation of real-time PCR (truncated mbtA gene) with reference to that of IS900 PCR showed the kappa statistics value with overall sensitivity of 100% and specificity of 91.1%, respectively.

**Conclusion:** Truncated mbtA gene was able to detect MAP isolates in clinical samples with significant sensitivity and specificity. Therefore, this gene can be incorporated as additional molecular marker for diagnosis of MAP.

## ISSHID Abstract-170 Lymphangioma circumscriptum mimicking genital warts

### Sudha R, Krishnakanth M, Gayathri R, Harshatha S, Murugan S

#### Department of Dermatology, Sri Ramachandra Institute of Higher Education and Research (SRIHER), Chennai, India

**Background:** Lymphangioma circumscriptum (LC), a common form of cutaneous lymphangioma, characterized by persistent, numerous clusters of translucent vesicles that more often than not contains clear lymphatic fluid . The surface changes in our case showed verrucous changes mimicking genital wart. The awareness of this entity is important so that the patient is not subjected to unnecessary therapeutic intervention.

**Case History:** A 54 year old female presented with complaints of raised skin lesions over vulva since one year, associated with itching without any discharge. She was referred to our opd for suspicion of genital warts. No history of trauma.There was history of application of podophyllotoxin (0.5%) once in 3 weeks for 6 months with no significant remission but she developed several bouts of recurrent cellulitis in a region of vulval edema. She had h/o carcinoma cervix ( stage II) diagnosed 3 years back, and completed 10 cycles of sequential radio and chemotherapy. Examination revealed multiple confluent verrucous papular eruptions over right vulva of size 7x6 cms. Few discrete papules were present over anterior fourchette . Shave biopsy was done and features were consistent with lymphangioma circumscriptum. She was then referred to a surgeon and was advised vulvectomy. Patient has given consent to the publication of their personal data.

**Conclusion:** According to many published articles acquired Lymphangiomas affecting the vulva presents strangely as verrucous growths mimicking like genital warts. Herein, the combination of cancer cervix, radiotherapy with recurrent cellulitis resulted in local lymphatic obstruction , lymphoedema and symptomatic lymphangiectasis that mimicked genital warts.

## ISSHID Abstract-172 Development of LAMP assay for detection of *Neisseria gonorrhoeae*

### Geetika Arora, Daman Saluja

#### Medical Biotechnology Laboratory, Dr B R Ambedkar Center for Biomedical research, University of Delhi, Delhi

**Background:** Gonorrhoea is one of the most common sexually transmitted diseases caused by the bacterium Neisseria gonorrhoeae (NG). In majority of cases it is asymptomatic, hence Syndromic Case Management (SCM) becomes unreliable. In this study, a Loop mediated isothermal Amplification (LAMP) assay for detecting NG has been optimized which can emerge as a dependable Point-of-care (POC) diagnostic test, being rapid and cost effective.

**Methods:** LAMP primers (2 Pairs complimentary to 6 region) were designed using Primer Explorer V5 (http://primerexplorer.jp/lampv5e/index.html) online tool, for a segment in ORF-1 gene of N. gonorrhoeae. Assay was optimized using genomic DNA isolated from N. gonorrhoeae WHO P strain for reaction temperature, reaction time, primers concentration, Betain concentration and MgSO4 concentration, and Analytical sensitivity was determined.

**Results:** A 205 bp region of ORF-1 gene was amplified for identification of NG. Upon optimization, the reaction temperature of 62.5°C was finalised for a reaction carried out for 75 minutes. Concentration of outer primers was fixed at 0.2μM which worked best with 8 times more concentrated Inner primers. 3.2mM MgSO4 concentration along with 0.4M betain concentration further enhanced the intensity of lamplicons. The assay could detect up to 10 femtograms (4-5 pathogens) of genomic DNA of NG.

**Conclusion:** LAMP is a sensitive and reliable method for detecting ORF-1 gene of *N.gonorrhoeae*. Following clinical evaluation, it can potentially emerge as a rapid and cost effective method for diagnosing patients with gonorrhoea.

## ISSHID Abstract-173 Subtractive Proteomic Approach to Combat Lymphatic Filariasis Caused by *Brugia malayi*

### P.A. Abhinand, Saranya Anandan, Manikandan Mariappan, Mahalakshmi Vijayaraj, PK.Ragunath

#### Department of Bioinformatics, Sri Ramachandra Institute of Higher Education and Research, Chennai, India

**Background:** Filariasis is an infectious tropical disease caused by nematodes - *Wuchereria bancrofti* & *Brugia malayi*, transmitted through mosquitoes and affects 120 million people worldwide. B.malayi is responsible for nearly 10% of Filiariasis cases and endemic to Southeast Asia and Indonesia. Current treatment regime against B.malayi consists of administration of Diethylcarbamazine or Ivermectin along with Albendazole, these drugs are microfilaricidal but not effective against adult worms. There is an urgent need for renewed efforts to discover macrofilaricides and/or embryostatic agents in addition to addressing the emerging threat of resistance.

**Methods:** The entire genome of *B.malayi* was screened for suitable therapeutic biomarker target by employing a subtractive proteomic approach based on the rationale that those proteins which are indispensable to the survival of the pathogen with no known homolog in humans will be an ideal target.On this basis 2,3-Bisphosphoglycerate-Independent Phosphoglycerate Mutase (iPGM) of *B.malayi* was chosen.The structure iPGM protein was modeled by homology method. Modeled iPGM was subjected to virtual screening by docking against a library of phytochemicals with experimentally proven anti-helminthic activity.

**Results:** Catechin, Epicatechin, Proanthocyanidin and Andrographoside were found to possess good binding affinity towards iPGM while Chlorogenic acid showed the best binding capacity forming 26 hydrogen bonds with a moldock score of -127.722.

**Conclusion:** Our study indicates that Chlorogenic acid has good binding with iPGM in B.malayi and posses ideal drug like properties and can be considered as a novel candidate lead molecule for treatment of Lymphatic Filariasis after subjecting it to suitable in vivo and in-vitro studies.

## ISSHID Abstract-174 Evaluation of virulence potential of ESBL and MBL positive isolates using *Caenorhabditis elegans* infection model

### Janani N, Bowiya P, Ishrath Razia R, Sambhavi Bhagavatheeswaran, Vinu Ramachandran, Anandan Balakrishnan, Prabu D

#### Dr.ALM PGIBMS, University of Madras, Taramani, Chennai-113, India

**Background:** Extended spectrum beta-lactamase (ESBL) and metallo-beta-lactamase (MBL) positive isolates are the important cause of nosocomial infection and pose a significant therapeutic challenge. Caenorhabditis elegans has been used as a model host to study the virulence potential of human pathogens. Hence, the present study was aimed to investigate the virulence potential of ESBL and MBL positive isolates using nematode killing assay.

**Methods:** Two ESBL and one MBL positive clinical isolates were included in our study. E.coli strain OP50 was used as a control strain. L4 stage of C.elegans was selected for nematode killing assay. The nematode was seeded in NGM agar plates with ESBL and MBL isolates and incubated at 20°C. Daily scoring of live and dead worms were performed using stereomicroscope

**Results:** Nematode killing assay showed ESBL and MBL isolates having lower life span than the control strain. The mean survival time for nematodes fed with resistant isolates was 7 days. The resistant strains carrying blaCTX-M, blaSHV, blaTEM and NDM gene was found to survive longer than the other resistant strains.

**Conclusion:** Data from our study supports C. elegans could serve as an alternate animal model in studying the virulence potential of antimicrobial resistant isolates.

## ISSHID Abstract-175 *Mycobacterium Tuberculosis* masquerading as malignant gall bladder mass & presents in emergency – A case report

### Suneha Kumari, D Shanmugam, Supradeep N, B. H. Srinivas

#### Jawaharlal Institute of Post Graduate Medical Education and Research (JIPMER), Pondicherry, India

**Introduction:** India accounts for about a quarter of the world’s tuberculosis cases. Tuberculosis can affect any organ, but tuberculosis of gall bladdder is rare, and very few reports exist regarding the same and even few presenting in emergency.

**Case Summary:** A middle-aged male, presented with 2 days history of right upper abdominal pain, vomiting and fever, and recent onset jaundice. On examination, his abdomen was distended with tenderness and guarding in the right hypochondrium. Computerised tomographic scan showed discontinuity in body of gall bladder with a pericholecystic collection and multiple intraabdominal lymph nodes, few necrotic. On emergency laparotomy, gall bladder was found distended with thinned out fundus and thickened proximal body. Seropurulent fluid was present in pelvis with pus flakes which was sampled for GeneXpert and bacterial culture. Due to frozen Calot’s triangle, subtotal cholecystectomy was done. Subpyloric necrotic node was biopsied for histopathological examination. Post-operatively the histopathological report was suggestive of acute suppurative cholecystitis and lymph nodes showed features of granulomatous inflammation. Bacterial culture of intraabdominal pus had no growth whereas GeneXpert detected high levels of mycobacterium tuberculosis sensitive to rifampicin. He had an uneventful post-operative period and was discharged after one week with ATT. Consent has been taken from the patient before publication of his medical data.

**Conclusion:** Unless a high degree of suspicion is maintained, diagnosis of tuberculosis can be easily missed. As in this patient, the clinical picture was of malignant disease and although the biopsy was suggestive of granulomatous infection, the diagnosis could be confirmed and treatment initiated, by the judicious testing of intraabdominal pus for mycobacterium.

## ISSHID Abstract-177 A comparative study of hydrolytic enzymes production by Candida spp isolated from patients and healthy individuals

### Shubhangi S^1^, Anandhi Lakshmanan^2^, B Rayvathy^1^

#### ^1^Department of Microbiology, Dr.ALM Post Graduate Institute of Basic Medical Sciences, University of Madras, Taramani, Chennai, India; ^2^Department of Microbiology, ESIC medical college and PGIMSR, KK Nagar, Chennai, India

**Background:** Candida spp are commensals in healthy humans and may cause life threatening infections in immunocompromised conditions. The pathogenicity of Candida spp is attributed to certain virulence factors such as adherence, biofilm formation and the production of tissue damaging hydrolytic enzymes. This study was designed to detect and compare the hydrolytic enzyme production by Candida spp isolated from normal and diseased individuals and to evaluate the role of these enzymes in pathogenicity.

**Methods:** A total of 10 clinical isolates and 10 isolates from oral rinse samples of healthy volunteers were collected. Candida spp were speciated using standard phenotypic methods. Phospholipase, hemolysin, gelatinase and catalase enzyme production was assayed using Egg yolk agar plate method; Sabouraud chloramphenicol agar (SCA) with 5% blood and 3% glucose; phosphate buffer saline and 12% gelatin; and 3% Hydrogen peroxide respectively.

**Results:** Phospholipase production was observed in 50% clinical isolates and 60% healthy isolates, though the effect of enzyme activity was more in clinical isolates. Hemolysis and catalase production was observed in all clinical and healthy isolates. C.tropicalis and C.parapsilosis was found to have higher hemolytic activity among clinical isolates. 53.1% of clinical isolates were found to have gelatinase production whereas none of the healthy isolates had the ability.

**Conclusion:** The enzymatic activity was determined and compared among clinical and healthy Candida isolates and it was found that there was a significant difference in the production and activity of gelatinase and phospholipase enzymes whereas no such difference was observed in the hemolytic activity and catalase production.

## ISSHID Abstract-179 Homology modeling and immunosuppressant peptide docking of CCR5: an initiation towards the development of peptidomimetics compound to treat HIV

### Harish Chander. A, Aarthi Rashmi B, V.Saravana Kumar, J.Saleth Mary Serena, Vasanth Nirmal Bosco J, Vidya M, Roshan Asharaf M, Goutham Bhargavan KK, Apsara Unni, Priyadharshini K

#### Department of Bio Informatics, Sri Krishna Arts and Science College, Coimbatore, Tamilnadu, India

**Background:** Biomolecular identification to treat HIV a challenging task to execute. The challenge was addressed by Homology Modelling and Peptide based Docking for the design of peptidomimetics.

**Methods:** A Protein on the surface of White blood cells that is involved in the immune system- CCR5 is used for Homology modelling. The PDB structure of CCR5 (5UIW) was analysed and its FASTA sequence was retrieved. The CABS DOCK web server provides an interface for modelling protein – peptide interaction using a highly efficient protocol for the flexible docking of peptide – proteins, since the peptides are immunosuppressant that haver diverse mechanism to suppress the immune system, but generally they are targeted against specific signalling molecule. Thus the immune suppressant peptides GGTS and GGST are docked against the protein CCR5.

**Results:** The CABS dock server provides the cluster density and RMSD value of the protein- peptide model. The RAMPAGE server shows 98% of allowed regions for the protein peptide model. In case of H bonding interaction, GGST interacts with 5 amino acids of CCR5, where as GGTS interacts only with 3 amino acids.

**Conclusion:** Further these homology modelling and immunosuppressant peptide docking can be used for QSAR based structural studies and ADME studies.

## ISSHID Abstract-180 CD4 and CXCR4 stimulation dependent cytosolic translocation of Topoisomerase II isoforms during HIV-1 infection

### Sunnam Lokeswara Balakrishna^1^, Anand K Kondapi^2^

#### ^1^Department of Biotechnology, Indira Gandhi National Tribal University, Amarkantak, Madhya Pradesh, India; ^2^Department of Biotechnology and Bioinformatics, School of Lifesciences, University of Hyderabad, Hyderabad, Telangana, India

**Background:** In HIV-1 infected cells, at early hours, co-localization of Topoisomerase II isoforms (Topo IIα and Topo IIβ) with HIV-1 reverse transcriptase in the cytosol and abortive reverse transcription in the absence of these isoforms were reported. But, it was not clear about the translocation of these nuclear enzymes to cytosol. In this report, we are providing some basic evidence regarding the role of CD4 and CXCR4 stimulation in cytosolic recruitment of Topo II isoforms in Jurkat cells.

**Methods:** Each receptor specific antibodies were used to mimic HIV-1 binding and stimulation of CD4 and CXCR4 receptors. The translocation of the Topo II isoforms from nucleus to cytosol was documented by using immunofluorescence and confocal microscopy.

**Results:** A remarkable cytosolic localization of Topo IIβ alone was observed from the beginning upon CD4 cell surface receptor stimulation. This localization was temporal and reached maximum at 60 minutes (which was the last timepoint under observation). In contrast to this, upon the stimulation of CXCR4 receptor, a slower and weaker cytosolic translocation of Topo IIβ was observed with even distribution of Topo IIα in both cytosol and nucleus at 10 minutes. During the course of study, gradual retention of Topo IIα by nucleus was observed and was completed by the end of 60 minutes of post incubation.

**Conclusion:** Individual stimulation of CD4 and its co-receptor CXCR4 present on the Jurkat cell surface can differentially induce Topo II isoforms translocation from nucleus to cytosol.

## ISSHID Abstract-181 Complement proteins as surrogate markers for diagnosis of active tuberculosis and monitoring of treatment response

### Nancy Hilda J, Jayakrishna Pamarthi, Bharat G Venkataratna, Veronika Thulasingam, Srikanth P Tripathy, Luke Elizabeth Hanna

#### Department of HIV/AIDS, National Institute for Research in Tuberculosis, Chetpet, Chennai, India

**Background:** When *Mycobacterium tuberculosis* enters the host, our immune system activates the complement system comprising a cascade of plasma proteins that mark pathogens for destruction by phagocytes. This study aimed to analyze changes in levels of three important complement proteins, C3a, C5a and C5b-9 complex, associated with tuberculosis disease and response to anti-TB treatment (ATT).

**Methods:** Ten millilitres of blood was obtained from 20 healthy volunteers at one time point and 20 newly-diagnosed pulmonary TB patients at three time points (before ATT, at completion of ATT and 6 months after completion of ATT). Plasma samples were used to quantify complement proteins. Nonparametric Wilcoxon matched pair test and Mann-Whitney U test were performed to compute statistical significance.

**Results:** Tuberculosis patients had significantly higher levels of C3a, C5a and C5b-9 complex than healthy volunteers (median values (nanogram): 7961, 21, 837 vs 7406, 14, 500) respectively. While C5a and C5b-9 complex levels (Newly diagnosed Vs 6th month Vs 12th month: 21 vs 17 vs 17 & 837 Vs 692 Vs 729) declined significantly with treatment, C3a levels did not decline with treatment.

**Conclusion:** New non-sputum biomarkers for diagnosis of active tuberculosis are our highest priority for global tuberculosis control. We found elevated levels of complement proteins C3a, C5a and C5b-9 in active tuberculosis patients, clearly indicating an association with tuberculosis. Furthermore, levels of C5a and C5b-9 declined with treatment associating with treatment response. Addition of these proteins to the existing biomarker panels may provide added value for the diagnosis and monitoring of tuberculosis.

## ISSHID Abstract-183 Development of new chemical entities of metal complexes with flavone derivative against anti-malarial activity with unique molecular target

### Sharow G Vincent^1^, Joseph J1^1^ and K. Nagashri^2^, R.R. Krishna Jyothi^1^

#### ^1^Department of Chemistry, Noorul Islam Centre for Higher Education, Kumaracoil, Tamilnadu, India; ^2^Department of Pharmaceutical Chemistry, Manonmaniam Sundaranar University, Tirunelveli, Tamilnadu, India

**Background:** Malaria is one of the dreadful parasitic disease which has contributed greatly to health encumbrance of the global population.The great discovery of platinum complex, cisplatin-PtCl2(NH3)2 as anticancer drug motivate researchers to synthesis metal complexes as chemotherapeutic agents with improved therapeutic efficacy from existing drug molecule, chloroquine. The current work was concentrated on the synthesis, structural elucidation and pharmacological studies of metal complexes with flavone derivatives as pharmacophore towards antimalarial activity.

**Methods:** The prepared ligands and their metal complexes were tested for in vitro antimalarial activity against *P. falciparum* strain (parasite lactate dehydrogenase assay), Chloroquine as standard and DMSO as vehicle control. The selectivity index and β-Haematin inhibition were also performed.

**Results:** The flavone derivatives showed weak antiplasmodial activities (IC5018.5-22.8μM) while an enhancement of activity was observed upon chelation with metal ions (IC50 6.23-10.60μM).

**Conclusion:** The antimalarial activity of ligands and their complexes was screened against chloroquine resistant *Plasmodium falciparum* malaria parasite strain. Coordination of metal ions with pharmacophore which resulted in enhancing antimalarial activities with respect to the free ligands due to highly hydrophobic nature of ligands induced by the different structural moieties increases their lipophilicity characters and thereby facilitates their diffusion across the bilayer membrane. Among the studied metal complexes, the copper complexes displayed low cytotoxicity and high selectivity induces the β-haematin inhibition towards the malarial parasites. The observed results suggested that copper complexes as potential molecule to be further developed as novel antimalarial agents.

## ISSHID Abstract-185 Phenotypic detection of biofilm formation by clinical isolates of Candida sp.

### Pooja K R^1^, Rayvathy B^1^, Anandhi Lakshmanan^2^

#### ^1^Department of Microbiology, Dr. ALM PG IBMS University of Madras, Chennai, India; ^2^Department of Microbiology, ESIC Medical College and PGIMSR, Chennai, India

**Background:** Among yeasts, the genus Candida is currently the most common cause of fungal infection worldwide. It is a well-known opportunistic pathogen. One of it’s major virulence mechanism is it’s ability to form biofilm. The present study has been designed to phenotypically detect potential biofilm producing clinical Candida isolates using three different methods and compare their relative efficiency.

**Methods:** A total of 40 isolates were obtained from various clinical sources and were speciated by standard laboratory techniques. The biofilm formation was phenotypically detected by Congo Red Agar (CRA) method and Tube method (TM) and the findings were compared with the results of the standard semi-quantitative Microtitre Plate (MTP) method.

**Results:** Out of 40 clinical Candida isolates, 12 were *C.albicans* and remaining were non-albicans (19-*C.tropicalis*, 6 - *C.parapsilosis*, 3 – *C.krusei* ). Majority of the isolates were from urine (50%), followed by vaginal swab (40%), sputum (5%) and pus (5%) samples. Biofilm production by TM and CRA showed positivity of 65% and 52.5% respectively, while the standard MTP method detected only 15%. Biofilm formation was observed only among non-albicans Candida with *C.tropicalis* being the predominant biofilm producer and urine samples showed higher biofilm producing isolates.

**Conclusion:** The study phenotypically detects biofilm formation among clinical Candida isolates using three methods. TM and CRA lacked specificity (41.17% and 47.05% respectively) but their sensitivity was 100% and 50% respectively when compared with the gold standard MTP method. So, we conclude that TM is a better phenotypic test which can be used for initial screening.

## ISSHID Abstract-186 Determination of Minimal Inhibitory Concentration of Salmonella enteric fever isolates to Ciprofloxacin & its susceptibility to Azithromycin

### Sumana.M.N, Nivedita.R.D

#### Dept of Microbiology, JSSAHER, Mysore, India

**Background:** Enteric fever caused by Salmonella enterica is a systemic illness of GI tract especially in tropical countries. Antimicrobial therapy is generally indicated but resistance towards commonly used antibiotics has limited their therapeutic usefulness. Due to emerging resistance to traditional antimicrobial agents, Azithromycin is increasingly being used for the treatment of Salmonella infections. Therefore, this study was aimed to know the true pattern of ciprofloxacin resistance by determining the minimum inhibitory concentration (MIC) through E-test and to screen Azithromycin resistance among Salmonella enteric serovar Typhi using Kirby-Bauer disc diffusion technique.

**Methods:** Prospective study was conducted over a period of 3 months with the clinical samples yielding growth of Salmonella and screened for Ciprofloxacin and Azithromycin susceptibility.

**Results:** Of the 40 isolates that were screened 30(75%) were resistant followed by 8 (20%) showed intermediate sensitivity and remaining 2(5%) were sensitive to ciprofloxacin by MIC. 36 (90%) of the isolates were sensitive and 4 (10 %) were resistant to Azithromycin by disc diffusion technique.

**Conclusion:** Resistance among Salmonella enterica clinical isolates was high. Ciprofloxacin resistant strains limit the therapeutic use of fluoroquinolones. This study also observed resistance to Azithromycin. However, the disc diffusion results for Azithromycin resistant strains have to be confirmed using MIC break points and routine monitoring of such resistance is necessary in order to effectively and rationally manage enteric fever cases. Hence, this study suggests that Azithromycin can be used as a convenient alternative anti-microbial agent only when the sensitivity report confirms it to be sensitive.

## ISSHID Abstract-187 Molecular characterization of opportunistic pathogens in the hospital environment: a neglected source

### S.Jayanthi^1^, D.Danisvijay^1^, S.H.ShifaMeharaj^1^, A.Sujhithra^2^, J.Perumal^1^

#### ^1^Department of Microbiology, CHRI-Chettinad Hospital and Research Institute CARE- Chettinad Academy of Research & Education, Kelambakkam, Tamilnadu, India; ^2^Department of Allied Health Science, Chettinad Academy of Research & Education, Kelambakkam, Tamilnadu, India

**Background:** Hospital environment contains a diverse population of microorganisms. Some agents are airborne and hospital environment could be a potential source of transmission of infection. These agents could have been originated from patients, staff, visitors, ventilation air conditioning system, water and soil resources.

Aim: The air quality of hospital environment indoor, outdoor air and domestic water resources are evaluated to determine the microbial contamination. Molecular characterization of the opportunistic pathogens from the environment.

**Methods:** Air samples were collected using exposure plate impingement technique in Chettinad Hospital and Research Institute. Nutrient agar exposure plates were incubated at 37°C and observed for growth after 48hours. Fungal plates (SDA) were incubated at 25°C and 37°C, observed upto 7 days. Water samples were processed by multiple tube method, presumptive Coliform count (Faecal coliform), plate count method. Microorganisms were identified using standard microbiological procedure. Molecular confirmation of the opportunistic pathogens were done by 16srRNA and 18srRNA gene amplification.

**Results:** In our study, quality of the air and water were analysed. Kochs sedimentation formula was used for CFUs of the microbes grown. Maximum isolates were *Staphylococcus* sps, *Pseudomonas* sps, *Candida albicans* in the indoor environment. Micrococcus sps, Pseudomonas sps, Candida non-albicans from the outdoor air and *Escherichia coli*, *Klebsiella* species from water. Comparative analysis and molecular characterization of microbes from the indoor, outdoor air and water were done.

**Conclusion:** Surveillance study should focus on monitoring strict environment hygiene and to prevent cross transmission of infections.

## ISSHID Abstract-189 Echinococcosis - Diagnostic profiling of suspected patients in relation to Anti-Echinococcus IgG ELISA in a tertiary care hospital

### Ketan Priyadarshi, Uneza Husain

#### Department of Microbiology, Institute of Medical Sciences, Banaras Hindu University, Varanasi, Uttar Pradesh, India

**Background:** Echinococcosis (hydatid disease) is important parasitic zoonoses. Serological methods, in addition to imaging and histopathology, are used in diagnosis of hydatid disease.

**Methods:** Anti-Echinococcus IgG antibodies were investigated by ELISA in serum of 40 patients with suspected hydatid disease. Clinical, radiological/imaging, histopathological and other laboratory test findings were obtained.

**Results:** Anti-Echinococcus IgG seropositivity was 32.5%(13/40). Among 13 IgG positive cases, 8 were confirmed on both HPE & imaging, 2 on HPE, 2 on imaging & 1 was not confirmed by either HPE or imaging. 7 out of 27 IgG negative cases had final diagnosis as hydatid disease (3 confirmed by HPE, 3 by imaging & 1 by both HPE & imaging). Among 20 hydatid disease cases, organs involved were liver(15/20), lung(2/20), hepato-gastric ligament(1/20), pelvic soft tissue(1/20), ovary & adnexa(1/20) & spleen(1/20). Wet mount was positive for Echinococcus in 3/20 cases. Out of 20, 2 had high eosinophil% (29.2% & 14.8%) & 3 had mildly elevated eosinophil%. Out of 27 IgG negative cases with cystic lesions in which hydatid disease was kept possibility, 11 had simple cysts [brain(1), liver(7), kidney(1), intestine & peritoneum(1), joints(1) & spleen(2)]. 3 had liver abscess and 6 had malignancy (1ovary & adnexa cyst/tumor, 1nonSCC lung & bronchus, 1primary lung with metastatic liver AdenoCA/mesothelioma, 1non-invasive mucinous cystic neoplasm of liver, 1primary yolk sac tumour of liver & 1biliary cyst adenoCA of liver & GB).

**Conclusion:** Anti-Echinococcus IgG antibodies detection by ELISA can be used as adjunctive tool in diagnosis of Echinococcosis, aiding imaging & histopathological diagnosis.

## ISSHID Abstract-190 A nutshell story of three isolates of rare pathogen *Fonsecaea pedrosoi* in nasal specimen at a tertiary care hospital

### Uneza Husain, Ketan Priyadarshi

#### Department of Microbiology, Institute of Medical Sciences, Banaras Hindu University, Varanasi, Uttar Pradesh, India

**Background:** Fungal Rhinosinusitis (FRS) is the mycotic infection of nasal and paranasal sinuses. *Aspergillus* spp. has always been considered the primary etiological agent of FRS but dematiaceous fungi are increasingly being reported. North India has been identified as endemic zone for paranasal mycoses possibly due to dust and frequent sand storms in summer months.

**Methods:** About 150 nasal secretions or mucus samples obtained by flushing nasal cavity with saline from patients suspected of rhinosinusitis in otorhinolaryngology department were included in the study. The study was performed after approval from Institutional Ethics Committee approval and informed consent. Direct microscopic examination along with culture isolation was done on paired Brain Heart Infusion blood agar and Sabouraud dextrose agar with antibiotics and incubated at 25°C and 37°C. Identification was done by growth characteristics, Lactophenol cotton blue mount from growth and slide culture. Nested polymerase chain reaction using pan-fungal internal transcribed spacer (ITS) primers was performed on clinical samples and culture isolates followed by sequencing and confirmatory identification.

**Results:** Apart from *Aspergillus* spp. which is most commonly isolated pathogen in such samples, 3 isolates of *Fonsecaea pedrosoi* were obtained in culture. Results of molecular studies were also affirmative.

**Conclusion:***Fonsecaea pedrosoi* is a well-known pathogen causing chromoblastomycosis, but isolation of these fungi which affects the skin and subcutaneous tissue, in nasal samples is interesting revelation for exploration in mycological research. Clinical correlation is essentially the most important criteria to rule out contamination in such samples and has to be stringently followed for better patient care

## ISSHID Abstract-192 Gut dysbiosis drives systemic CD4 T cells activation in perinatally HIV-infected children

### Urvinder Kaur S^1^, Preeti Moar^1^, Anita Shet^2^, Himanshu D^3^, Ravi Tandon^1^

#### ^1^Laboratory of AIDS Research and Immunology, School of Biotechnology, Jawaharlal Nehru University, New Delhi, 110067, India; ^2^International Vaccine Access Center, Johns Hopkins School of Public Health, Baltimore, MD, 21205, USA; ^3^Department of Medicine, King Georges Medical University, Lucknow, 226003, India

**Background:** Persistent immune activation is a hallmark of progressive HIV infection. Immune activation persists despite antiretroviral treatment (ART) and correlates with a higher occurrence of non-AIDS morbidities in HIV-infected individuals. Gut microbial dysbiosis during chronic HIV infection in adults is associated with persistent systemic immune activation and inflammation. Previously, we showed altered gut microbial composition regardless of ART and its association with inflammation in perinatally HIV-infected children. Here, we study the level of immune activation and its association with gut microbial dysbiosis in them.

**Methods:** Frequency of activated T cells was analyzed in treatment naïve, ART-suppressed perinatally HIV-infected children and uninfected controls using flow cytometry. Multivariate analysis was performed to evaluate the association of systemic activated T cells with the relative abundance of significantly distinct genera in HIV-infected children.

**Results:** HIV-infected children had significantly higher CD38+ HLADR+ frequencies in CD4 as well as CD8 T cells compared to uninfected controls. Activated CD8 T cells remain elevated in HIV-infected children regardless of ART. Frequency of activated CD4 T cells showed positive association with Haemophilus and negative association with Ruminococcus and Bifidobacterium in HIV-infected children.

**Conclusion:** Our data suggest that the gut microbial dysbiosis in perinatally HIV-infected children contributes to immune activation. Members of Ruminococcus and Bifidobacterium genera include butyrate-producing species, which are essential for maintaining the gut epithelial barrier and preventing microbial translocation.

## ISSHID Abstract-193 Determination of Oxidative Stress and functional role of C-Reactive Protein in patients with tuberculosis

### Solomon Raj.J, Andrew Pradeep.M, Nandhini.R.S, Stella Mary.J

#### Department of Microbiology, The American College, Madurai, Tamil Nadu, India

**Background:** Tuberculosis is a global public health problem and it is second to HIV/AIDS as the leading infectious cause of death of adults worldwide. MTB is a recognized facultative intracellular bacteria that replicate and persists within macrophages. Mycobacteria can induce reactive oxygen species (ROS) production by activating phagocytes and although these are important part of the host defence against Mycobacteria, enhanced ROS generation may promote tissue injury and inflammation. ROS are highly toxic to all types of cells, but especially to lipids (fat cells) causing peroxidation. . C–reactive protein (CRP) is the prototypical acute phase proteins in humans. It is widely used as a marker of infection, and also to aid the differentiation between a bacteria and a viral origin. CRP is produced by the liver.

Methods A total of 50 tuberculosis patients attending Anti Tuberculosis Therapy in government Rajaji Hospital, Madurai were subjected in the present study. Ethical Committee constituted by Government Rajaji Hospital approved and gave ethical clearance for sample collection. Samples were collected in the informed consent of the patients. Reactive Oxygen Species of serum can be determined by Nitro Blue Tetrozolium reduction assay. C-Reactive Protein – Immuno Turbidimetric method.

**Results:** Qualification of CRP by immune Turbidimetric method Documents the presence of inflammation in Tuberculosis Patients. Present study Documents Increased level of Oxidative Stress and Lower level of Anti-oxidant status in Tuberculosis Patients.

**Conclusion:** Study suggests modulation of above parameters facilitates an effective therapeutic care and improvised host immune surveillance.

## ISSHID Abstract-194 Insights in to Comparative Docking Study of Selected Molecules Vs Standard HIV Drugs against HIV target enzymes

### Parakkot Ramkakrishnan Surya^1^, Ayyadurai Jerad Suresh^2^, Wilson Aruni^1^

#### ^1^School of Pharmacy, Sathyabama Institute of Science and Technology, Chennai-600019, India; ^2^Colleges of Pharmacy, Madras Medical College, Chennai, India

**Background:** The HIV/AIDS pandemic remains a major public health problem. Hence there is a need to continue the research for new chemical entities that can combat HIV safely and effectively.

**Methods:** Drug design predicts a binding interaction between a small molecule ligand and an enzyme protein. This results in activation or inhibition of the enzyme. Protein Ligand Docking is carried out by Argus lab® 4.0 software.

**Results and Discussions**: A series of novel molecules (Pyridine, Thiadiazole, Benzimidazole; and Acetyl thiophene derivatives) were designed and molecular docking studies were performed against three critical HIV target enzymes Reverse Transcriptase (RT), Protease Retro pepsin (PR) and Integrase (IN) with PDB id 3OXC, 4R5P and IFEJ respectively. All the molecules were screened for their docking score and protein ligand interactions. Based on the analysis of docking results, Benzimidazole derivatives showed good docking score compared to other series. Four standard HIV/AIDS drugs Zidovudine (NRTIs), Rilpivirine (NNRTIs), Saquinavir (PIs), Elvitegravir (IIs) also docked against the HIV proteins. The docking results of novel molecules and standard drugs against HIV proteins was compared and analyzed.

**Conclusions:** All the molecules may exhibit better anti HIV activity. The most active anti HIV agent perhaps could be the one which acts significantly well against all the critical enzymes rather than against one or two. The current study gives insights in to the development of new drugs against HIV/AIDS. Results of the comparative molecular docking studies of selected molecules Vs standard HIV drugs against HIV proteins lights up for future synthetic studies.

## ISSHID Abstract-196 Gonorrhea in Men having sex with men (MSM): A study from tertiary care centre, Bangalore, India

### Sneha K Chunchanur, Shwetha JV, Ambica R, Leelavathy B, Revathi

#### Bangalore Medical College and Research Institute, Bengaluru, India

**Background:** Gonorrhoea is second most prevalent STI. In MSM, extra genital infections which are asymptomatic lead to increased transmission rate. Drug resistance is also of concern.

**Methods:** 30 Consecutive MSM with or without urethral discharge attending STI clinic were recruited. The urethral swab was subjected to Gram stain, culture and real time PCR. Antimicrobial susceptibility was performed by CDS, CLSI and E test, WHO C and WHO K strains were used as control strains. The AST results were confirmed with apex regional STD Laboratory, VMMC & Safdarjung Hospital, New Delhi. The oropharyngeal swabs were subjected to real time PCR.

**Results:** Out of 30 MSM, predominant were between age group 21-30 years, 9 were bisexual, majority of them practiced both oral and anal sex , 10 were positive for one more STI.

Out of 23 urethral swabs, 18 were positive for Gram negative diplococci, 11 grew in culture, 20 were positive by real time PCR. Out of 14 oropharyngeal swabs, 10 were positive by real time PCR. AST results showed that all strains were resistant to penicillin, ciprofloxacin and tetracycline. All strains were sensitive to cefixime and ceftriaxone, 2 strains were resistant to azithromycin.

**Conclusion:** To the best of our knowledge, from India this is the first study to focus on gonorrhoea in MSM including molecular detection and antimicrobial susceptibility test which showed emerging resistance to azithromycin which is one of the drug prescribed by NACO under syndromic management; more such studies are required to strengthen national guidelines with appropriate data.

## ISSHID Abstract-199 Evaluation of the cytokine status in tuberculosis patients

### R. Gopinath^1^, Suthandira Munisamy^2^, Elanchezhiyan Manickan^1^

#### ^1^Department of Microbiology, Dr. ALM. Post Graduate Institute of Basic Medical Sciences, University of Madras, Taramani, Chennai, India; ^2^IRT Perundurai Medical College, Perundurai, Erode District, Tamil Nadu, India

**Background:** The encounter of immune cells with the Mycobacterium tuberculosis (MTB) bacilli leads to a cascade of several immune events. The development of initial cytokine milieu stimulated by the innate immune cells plays a vital role in TB. Later on with the progression of immune response, certain specific cytokine lineages are produced by the adaptive immune system and this includes the Th1 and Th2 lineages of cells equipped with specific functions. A Th1 and Th2 cell response determines the outcome of several disease conditions. Hence it is with this background the current study was performed to measure the cytokine status of TB patients in comparison with the controls.

**Methods:** A total of 300 serum samples were collected from TB patients (156 treatment naïve and 144 under therapy) and 100 age cum sex matched control subjects were collected. Institutional Human Ethics Committee Approval was obtained. These samples were subjected to evaluation of the levels of Th1 cytokines IFN-γ and TNF-α and Th2 cytokines- IL-10 and IL-6 by employing ELISA technique.

**Results:** Results revealed higher production of Th2 cytokines among untreated TB cases. In contrast treated TB cases showed a reversal of cytokine trend towards Th1 type cytokines. These differences in cytokine pattern among untreated versus treated patients were not biased with gender or age.

**Conclusion:** Cytokine niche plays a major role during TB. It is assumed that immune phenotype matures into Th2 during TB (non protective) and treatment with anti TB drugs reverses the cytokine phenotype back to Th1 type (protective).

## ISSHID Abstract-201 Hidradenitis Suppurativa Complicating HIV – A rare case report

### Murugan Sundaram, Adikrishnan.S, Krishnakanth.M, Sudha.R, Mahalakshmi.V, Anandan.S, Sukesh Gautam.S

#### Department of Dermatology, Venereology and Leprosy, Sri Ramachandra Institute of Higher Education and Research, Porur, Chennai, India

**Background:** Hidradenitis suppurativa (Acne inversa/Pyoderma fistulans significa/Verneuil’s disease) is a chronic suppurating infection that affects the apocrine glands of the axilla, groin and the perineum. There is the formation of multiple intradermal abscesses, which lead to the development of multiple sinuses, fistulae, and scarring of the skin. Herewith, we report a rare case of hidradenitis suppurativa in a HIV seropositive individual.

**Case Report:** A 35-year-old presented with complaints of swellings in both axilla associated with pain. History of recurrence of lesions present since one year. Patient gave history of unprotected extramarital contact with a commercial sex worker 2 years back. No h/o diabetes mellitus or tuberculosis. On examination, multiple tender nodules, pustules, abscesses with sinuses draining purulent material were present over both the axillae. Multiple healed scars were seen on the axillae. Axillary lymph nodes- firm, enlarged & discrete. Differential diagnosis considered were furunculosis, scrofuloderma and hidradenitis suppurativa and actinomycosis. Routine blood investigations were normal. VDRL was non-reactive, HIV ELISA & western blot was positive. The patient was initiated on ART and was treated with topical mupirocin and oral doxycycline for the axillary lesions. The patient has given consent to the publication of their personal data.

**Conclusion:** Association of HIV with a chronic skin condition like HS is a therapeutic challenge. Etiology may also have an endocrinal component, and HIV-associated endocinopathies may alter the course of the disease. Use of retinoids for management of HS in HIV-positive patients on ART needs regular monitoring. TNF-α inhibitors like Infliximab may be a safe and effective therapeutic option for severe refractory HS in HIV-positive patients.

## ISSHID Abstract-202 Bacteriological profile of endotracheal aspirates and their antibiotic susceptibility pattern in patients suspected of ventilator-associated pneumonia (VAP)

### Vaishali Kumar.S.M, Tejashree.A, Krishna Karthik.M, Vidyavathi.B.C, Satya Sai.B

#### Department of Microbiology, JSSAHER, Mysore, India

**Background:** Critically ill patients of intensive care units are always at greater risk for acquiring hospital-associated infections with MDRO. VAP is one of the most common nosocomial infections seen among the patients admitted in ICUs and it is most commonly associated with high morbidity and mortality. It is essential to have knowledge of local antimicrobial resistance patterns even before starting empirical therapy. Hence, this prospective observational study is aimed to analyze the common microorganisms associated with VAP and their antibiotic sensitivity profile for over a period of 6 months

**Methods:** This is a prospective observational study done on clinically suspected cases of VAP who were on a mechanical ventilator in the ICUs of our hospital over a period of 6 months. Microbiological profile and the sensitivity patterns of the isolates were analyzed.

**Results:** Of 1279 ET aspirates 594 yielded significant growth. The predominant organisms isolated were *Acinetobacter spp*. (38%), followed by *Klebsiella spp*.(26.26%), *Pseudomonas spp.* (17.3%), *S. aureus* (4%), *E. coli* (2.5%), *Candida spp*. (2.3%) and *Serratia spp*. (2%). MDR was predominantly observed in *Acinetobacter spp*. (14.3%) followed by *Klebsiella spp*.(4.4%), Pseudomonas spp.(3%) and which were sensitive to colistin (100%), minocycline (89.23%), tigecycline (82.16%). 4 (16.6%) isolates among S. aureus were MRSA.

**Conclusion:** A local antibiogram for each hospital, based on bacteriological patterns and susceptibilities is essential not only to initiate empiric therapy but also to prevent poor outcomes and help in framing the appropriate institutional antibiotic policy.

## ISSHID Abstract-203 Increasing trend of tigecycline resistance in clinical isolates of *Acinetobacter baumannii*

### Ajit R. Sawant^1^, Sudhakar Pagal^1^, Sheela Devi C^2^, Shashikala P^2^, Kanungo R^2^, K. Prashanth^1^

#### ^1^Department of Biotechnology, School of Life Sciences, Pondicherry University, Pondicherry, India; ^2^Department of Clinical Microbiology, Pondicherry of Institute Medical Sciences (PIMS), Pondicherry, India

**Background:***Acinetobacter baumannii* infections are global threat to public health due to limited treatment options and significant mortality. To determine trend of antibiotic resistance among *A. baumannii* isolates isolated over a period of time and its correlation with the presence of resistant determinants

**Methods:** A total of 240 *A. baumannii* clinical isolates isolated from a referral hospitals at different time points (2009 to 2018) were investigated. These isolates were subjected to antimicrobial susceptibility testing using agar and broth dilution followed by PCR screening for detection of resistant determinants.

**Results:** High percentage resistance was observed in *A. baumannii* isolates, wherein more than 75% of isolates were resistant to aminoglycosides and carbapenems, however, colistin is exception. PCR revealed high prevalence of β-lactamase *blaOXA*-23 (64%) and 16SrRNA methylase armA (82%). There is strong co-relation between the metallo-β-lactamase (*blaNDM*-1) and 16SrRNA methyltransferases (armA or rmtC) wherein isolates that are positive for blaNDM-1 also harboured armA or rmtC. Increasing percentage of resistance to tigecycline was observed among the isolates over a period of time. The MIC for tigecycline was 1.5mg/L in 2015 that was increased to 4mg/L in 2018. Total 58% isolates of year 2018 were showing resistance towards tigecycline (MIC = 4mg/L), which is a worrisome development. Use of efflux pump inhibitor CCCP with tigecycline resulted in two-fold decrease in MIC implicating active efflux pumps in tigecycline resistance.

**Conclusion:** The resistance towards tigecycline has steeply increased among the isolates of 2018 revealing the alarming rise of tigecycline resistance in very short span of time.

## ISSHID Abstract-204 Proportion of sexually transmitted infections among pregnant women and its outcome

### Hemavathi, Brunda Muniswamy, Sharvani.R

#### Sapthagiri Institute of Medical Sciences and Research Centre (RGUHS), Bangalore, India

**Background:** The worldwide burden of STI is estimated to be 350 million cases/year, most of which occurs in developing countries. STIs in pregnancy are associated with adverse pregnancy outcome. About 90% of HIV, hepatitis B, C (HBV, HCV) and syphilis infections in children result from mother-to-child transmission and can occur during pregnancy and peripartum. This study is to determine the proportion of these infections among pregnant women attending the antenatal clinic of our hospital and post treatment outcome.

Material and Method: Data from January 2014 to December 2018 was analyzed.

**Results:** Total number of pregnant women tested were 8287 , out of which 56(0.67%) were positive for any one STI- 0.25% were positive for HIV, 0.37% positive for HBV , and 0.01% and 0.03% were positive for HCV and syphilis respectively.

No mother to child transmission of HIV noted and all children are seronegative at the end of 18months follow-up.

**Conclusion:** To fulfill the WHO target to “eliminate mother-to-child transmission of HIV by 2030”, accelerated detection and treatment of all seropositive pregnant women has reduced other STI s also. The above study highlights the active intervention and follow-up is the road for success to achieve the WHO goal but, proportion of hepatitis B infection rate has increased in spite of good vaccine availability. Policy makers can consider awareness regarding hepatitis B infection and vaccination also to be incorporated along with the HIV awareness program. Vaccination may be made mandatory among adolescents.

## ISSHID Abstract-207 Molecular detection of virulence & resistance determinants in commensal & invasive strain populations of *S. epidermidis* and development of multiplex assay for rapid detection of invasive *S. epidermidis*

### Suresh Sah^1^, Satish kumar Amarnath^2^, K. Prashanth^1^

#### ^1^Department of Biotechnology, School of Life Sciences, Pondicherry University, Puducherry, India; ^2^ Manipal Cure and Care, Banglore, India

**Background:** In coagulase-negative staphylococci (CoNS), the most important member is Staphylococcus epidermidis. *S. epidermidis* is most abundant colonizer of human skin and is mainly responsible for chronic device-related infections and also known to evade host immunity. Specifically, exopolymers, such as the exopolysaccharide of *PIA* contributes to the formation of biofilms that inhibit the activity of human antimicrobial peptides and phagocytosis. Some important molecular factors namely *SdrG*, *AtlE* and *PBP2a* are implicated in this pathogen’s success by helping in colonization and immune evasion. This study evaluates the utility of detection of these factors through multiplex-PCR that might assist in quicker diagnosis *S. epidermidis* infection.

**Methods:** PCR was performed to detect different resistant and virulence determinants in *S*. *epidermidis* isolates. PCR detection of resistant (mecA) and virulent determinants (*atlE*, *sdrG*, *icaAB*) for 158 (110 clinical & 48 commensal) isolates of *S*. *epidermidis* was performed. Multiplex PCR was performed for the evaluation of the utility of such method for quicker diagnosis of *S*. *epidermidis* infection.

**Results:** Presence of virulence gene *icaAB* and resistant gene mecA were observed more in invasive strain category. Conversely, the presence of *altE* and *sdrG* was more in the commensal group of isolates. We have also successfully developed multiplex PCR wherein simultaneous amplification of four important genes and one species-specific gene achieved in single step.

**Conclusion**: The present study successfully used multiplex PCR assay for detecting some of the important virulence markers essential of pathogenic strain that can discriminate pathogenic invasive *S. epidermidis* strains from that of commensal contaminants.

## ISSHID Abstract-209 Anogenital warts on a rampage - 3 Challenging case reports

### Gayathri Rajesh, Harshatha S, Krishnakanth M, Murugan S

#### Department of Dermatology, Sri Ramachandra Institute of Higher Education and Research, Chennai, India

**Background:** Anogenital warts have a huge psychological impact on patients,as the treatment is difficult,requires time and multiple sessions with incomplete clearance. Hence,a treatment which is easy to use,with a good safety profile and low-recurrence rate is always desirable especially in difficult scenarios like pregnancy,HIV and in children.

Case Report: CASE 1– A 25-year old boy,presented with asymptomatic papulesover penis. O/E- multiple non-pruritic verrucous papules present over glans penis extending to the perianal region. He was on podophyllotoxin 0.5% lotion biweekly with oral retinoids 25mg for 4 months outside. Patient was resistant to management and hence we evaluated him further. Serology showed HIV positivity, CD4counts- 350. Sequential therapy with Cryotherapy, 5% imiquimod was started with oral retinoids 25mgdaily.

CASE 2 – A 27-year-old,8 weeks pregnant female presented with asymptomatic verrucous papules over vulva. On examination, multiple confluent verrucous papules over vulva extending into vagina and anal region. Serology-negative. Freezing touch cryotherapy done weekly once until remission.

CASE 3- A 14-year-old boy studying in hostel presented with aymptomatic lesions over penis. On examination, multiple verrucous papules over penis extending onto the anal area. History of exposure with fellow students was present. Serology and viral markers were negative. Radiofrequency ablation was done for bigger lesions along with 5% Imiquimod twice weekly and oral vitaminA 50000IU was started

Skin Biopsy was consistent with verruca vulgaris. All patients showed excellent results and no recurrence on follow-up. Consent was obtained from all the patients for publication of their data.

**Conclusion:** Hence using these therapeutic modalities early in the course of disease, can save the patient of psychological stress and physicians of long, repeated cumbersome treatment options.

## ISSHID Abstract-213 Profile of Infections in the 30 day post-operative period among Cardiothoracic surgery patients in a tertiary care centre in South India

### Vidya Krishna^1^, Srinivasan R^2^, Aditya Sanjeevi^2^, Periyaswamy .T^3^

#### ^1^Department of Infectious Diseases, Sri Ramachandra Institute of Higher Education and Research, Chennai, Tamil Nadu, India; ^2^Department of General Medcine, Sri Ramachandra Institute of Higher Education and Research, Chennai, Tamil Nadu, India; ^3^Department of Cardiothoracic Surgery, Sri Ramachandra Institute of Higher Education and Research, Chennai, Tamil Nadu, India

**Background:** Infections after cardiothoracic surgery occur in 5-21% of cases and add to morbidity, mortality and healthcare costs. Antimicrobial resistance rates are a matter of significant concern. We aimed to study the profile of postoperative infections after cardiothoracic surgeries in a tertiary care centre in South India.

**Methods:** Retrospective study of records of patients aged above 18 years of age who underwent cardio-thoracic surgery from January- December 2017. All culture positive infections that occurred in first 30 days post-surgery were included. Type of infections and organism profile were studied.

**Results:** During the study period, a total of 694 cardio-thoracic surgeries were performed. 54 cases were excluded (children under 18 years, missing records). Of the 640 cases, 41 patients (6.4%) developed culture positive infections in the first 30 days after surgery. 29 (71%) of these patients developed infection > 7 days and 7 (17%) of these required readmission. Type of infections were- SSI 30 (73%), Respiratory -5 (12%), UTI- 4 (10%) and Sepsis-2 (5%). 7 (17%) patients had polymicrobial cultures. Gram negatives caused 59% (n=30) and Gram positives caused 41% (n=21) of all infections. Among gram negatives, ESBL rates were 50% and CRO was 6.7%. Of gram positives, 42.8% were MR-CONS and 14.3 % were MRSA. One patient died due to VAP and MODS.

**Conclusion:** Majority of infections occurred after 7 days post-operative period. SSIs were the commonest infections and antibiotic resistance was seen around 60% of all infections.

## ISSHID Abstract-215 Metal complexes containing 2-aminobenzothiazole derivative as potential bioactive agents for anti-TB

### A. Suman^1^, J.Joseph^2^, K.Nagashri^3^

#### ^1^Department of Chemistry, Manonmaniam Sundaranar University, Tirunelveli, Tamilnadu, India; ^2^Department of Chemistry, Noorul Islam Centre for Higher Education, Kumaracoil, Tamil Nadu, India; ^3^Department of Pharmaceutical Chemistry, Manonmaniam Sundaranar University, Tirunelveli, Tamilnadu, India

**Background:***Mycobacterium tuberculosis* infects nearly one-third of the world’s population and kills about 1.3 million people annually. Treatment of drug-susceptible tuberculosis (TB) still uses therapeutic schemes that were introduced decades ago, and implies long-term treatment with isoniazid, rifampicin, ethambutol, and pyrazinamide. There is a growing need for new drug molecules with a novel mode of action for anti-TB. The objective of this work was focused on the synthesis, characterization and antimycobacterial activity of metal complexes (Cu(II), Ni(II), Co(II) and Zn(II)) with 2-aminobenzothiazole derivative.

**Methods:** The MIC values were measured for metal complexes containing 2-aminobenzothiazole derivative against M. tuberculosis strain H37Rv using MABA method and compared with ethambutol (standard).

**Results:** Among the studied complexes, the Cu(II) complex showed higher inhibitory activity with 12.36 mg/mL whereas other complexes exhibited moderate activity with MIC value in the range 14.6–24.50 mg/mL, respectively as compared to standard, ethambutol.

**Conclusions:** In conclusion, the antimycobacterial activity of synthesized complexes was studied. The potential activity of metal complexes are may be due to the lipophilic nature of complex facilitates the diffusion into the cell membrane and subsequently induces cell destruction. The other constituents, solubility, conductivity, dipole moment, and DNA binding may also contribute to the increased activity of the complexes. The observed findings make the synthesised copper complex of 2-aminobenzothiazole derivative as a promising candidate for future investigations in the development of anti-TB drugs may lead to a new paradigm in TB drug development.

## ISSHID Abstract-216 Outbreak of Measles in Barganwa village, Uttar Pradesh, India-May 2019

### Manjeet Singh Choudhary^1^, Valan Adimai Siromany^2^, Kevisetuo Anthony Dzeyie^3^, Ismeet Kaur^3^, Satyaprakash Singh^4^, Rajesh Yadav^2^, Binod Kumar Agrawal^4^, Pauline Harvey^3^

#### ^1^WHO-India EIS Officer, National Public Health Surveillance Project- World Health Organization, India; ^2^Centers for Disease Control and Prevention, India Country Office, New Delhi, India; ^3^National Public Health Surveillance Project – World Health Organization, India; ^4^Department of Medical Health and Family Welfare, Sonbhadra, Uttar Pradesh, India

**Background:** Measles continues to be a major health problem in India, largely affecting children aged <5 years. In 2018, India accounted for 11% (52,308) of measles cases reported globally. In April 2019 a traditional healer alerted Sonbhadra district health authorities about 5 suspected measles cases in Barganwa village, Uttar Pradesh state reported to have >85% immunisation coverage in the village based on administrative records.

**Methods:** We defined a probable case as new onset of fever and maculopapular rash with cough or coryza or conjunctivitis occurring between 1 February and 20 June 2019, in a resident of Barganwa. We reviewed the health workers register, outpatient records of govt.health facilities and conducted a house-to-house search in Barganwa to identify cases. We interviewed probable cases and collected information on clinical presentation, travel, vaccination and vitamin-A history through structured questionnaire. Census details were taken, and attack rate were calculated. Blood specimens collected from five probable cases were tested for anti-measles IgM by enzyme linked immunosorbent assay (ELISA).

**Results:** We identified 24 probable cases in Barganwa between 10 March-09 May 2019; attack rate 0.6%; median age 4 years (range 9 months-13 years); 63% were male. Ten children had diarrhoea, eight (33%) symptomatically treated, none hospitalized and no deaths. 21of the probable cases (88%) received MCV1, 58% received MCV2 dose through routine immunization; five (20%) received single vitamin-A dose post-rash onset. Three of the five blood specimens tested positive for anti-measles IgM.

**Conclusion:** A measles outbreak occurred in this community with vaccination coverage below the WHO recommended rates of 95% coverage with two MCV doses. Improvements in MCV1, MCV2, vitamin-A coverage, continue surveillance and plans for vaccination coverage survey in the district would be valuable in reducing measles cases in this area.

## ISSHID Abstract-217 Anti-candida activity of metal complexes with quinoline derivatives comprising different pharmacophore units as novel antifungal agent

### B. Ebenezer^1^, K. Nagashri^2^, A.Suman^1^

#### ^1^Department of Chemistry, Manonmaniam Sundaranar University, Tirunelveli, Tamilnadu, India; ^2^Dept of Pharmaceutical Chemistry, Manonmaniam Sundaranar University, Tirunelveli, Tamilnadu, India

**Background:** In worldwide, the fungal strains are caused diseases that induced a public health problem worldwide. Fungal infections caused by the genus Candida are becoming more frequent and are associated with high morbidity and mortality. The commercially or clinically available antifungal chemotherapeutic molecules are inadequate to treat life-threatening infections of bacterial and viral diseases. Therefore, there is an emerging necessity to develop novel with different pharmacopore units, efficacious, non-toxic with improved spectrum anticandida activity.

Method: The chemical modification on quinoline molecule with different structural core which yielded different ligands in appropriate yields. Subsequent reaction of these ligands with metal(II) acetate yielded metal(II) complexes and were characterised. All of the free ligands and their metal complexes against clinical isolate of fungal strain, C.albicans were screened (broth microdilution susceptibility protocol) and commercially available antifungal drugs, ketoconazole and Amphotericin B utilized as standards.

**Results:** The ligand didn’t exhibit anti candida activity. Among the studied complexes, the copper complex demonstrated anti-Candida activity (MIC = 20 μM) as compared with commercially available antifungal agent, ketoconazole (MIC = 25 μM)

**Conclusion:** The anti-candida activity of complexes may be due to increases in their lipophilic characters and thereby causing polarity reduction of the fungal membrane and enhancing antimicrobial activity. The antifungal efficiencies indicated that structural scaffolds such as the donor groups, sizes and the metallic ions in the complexes may be significantly influence their anticandida activities. Therefore, the effective copper complex with quinolone derivative showed broad-spectrum of activity and leads to drug candidates after further clinical analysis.

## ISSHID Abstract-218 Efficacy of anticancer drugs 5-Fluoro uracil and Gemcitabine on HIV-1 infections – A Drug Repurposing Approach

### M Divyabharathi^1^, J Rajesh^1^, M Sai Akilesh^1^, Ashish D Wadhwani^1^, P Selvam^2^

#### ^1^Department of Pharmaceutical Biotechnology, JSS Academy of Higher Education & Research – JSS College of Pharmacy, Ooty, Tamil Nadu, India; ^2^Aravindh Herbal labs (P) LTD., Rajapalayam – 626117, Tamilnadu, India

**Background:** Drug repurposing is identifying new use for existing drug with well-established drug profile. Due to lack of newer antiviral drugs and increase in resistance to current generation of antiviral drugs, drug repurposing offers strategies to treat viral infections. The objective was to repurpose the anticancer drugs 5-Fluoro Uracil and Gemcitabine for HIV infection and were chosen on the basis of their mechanism of action on cancer.

**Methods:** The study plan carried out to evaluate the ability of 5-Fluoro Uracil and Gemcitabine for anti-HIV activity by preliminary cytotoxicity studies followed by in vitro anti-HIV activity by neutralization assay.

**Results:** Cytotoxicity studies: 5-FU and Gemcitabine IC50 values showed cytotoxicity of 36 ± 4.95 μg/ml and 26±1.41μg/ml in TZM-bl cells respectively.

Invitro Anti HIV activity by neutralization assay

- Cell associated- In cell associated assay the drug 5-FU showed EC50 of 5.61 and 4.57 μg/ml against HIV-1 UG070 and HIV-1 VB59 primary isolates respectively. Gemcitabine showed EC50 of 4.51 and 3.19 μg/ml against HIV-1 UG070 and HIV-1 VB59 primary isolates respectively.
- Cell free - In case of cell free assays, the drug 5-FU showed EC50 of 4.7 and 4.57 μg/ml against HIV-1 UG070 and HIV-1 VB59 primary isolates respectively. Gemcitabine showed EC50 of 3.19 and 4.23μg/ml against HIV-1 UG070 and HIV-1 VB59 primary isolates respectively. This clearly indicates that the drugs have potent antiviral activity against HIV-1 UG070 and HIV-1 VB59 primary isolates

**Conclusion:** Our findings suggest that the anticancer drugs 5-Fluoro Uracil and Gemcitabine shows potent antiviral activity against HIV-1.

## ISSHID Abstract-219 Evaluation of the inhibitory effect of anticancer drugs 5-Fluoro Uracil and Gemcitabine on HSV - 1 & 2 Infections – A Drug Repurposing Approach

### M Divyabharathi, M Sai Akilesh, J Rajesh, Ashish D Wadhwani

#### Department of Pharmaceutical Biotechnology, JSS Academy of Higher Education & Research – JSS College of Pharmacy, Ooty, Tamil Nadu, India

**Background:** Due to lack of newer antiviral drugs and increase in resistance to current generation of antiviral drugs, drug repurposing offers an attractive strategy to treat viral infections. The objective of the study was to repurpose the anticancer drugs 5-Fluoro Uracil (5-FU) and Gemcitabine for HSV infection and were chosen on the basis of their mechanism of action on cancer.

Method: The study plan was carried out to evaluate the ability of 5-FU and Gemcitabine for anti-HSV activity by cytopathic effect inhibition assay, virucidal assay, MTT antiviral assay and plaque reduction assay.

**Results:** In Vitro cytotoxicity studies: 5-FU and Gemcitabine showed cytotoxicity of 136 ± 5.29 μg/ml and 78.67 ± 6.51 μg /ml in VERO cell culture.

In Vitro anti-HSV activity -

Cytopathic effect inhibition assay: 5-FU exhibited 100% cell protection against the cytopathic effects caused by the HSV-1 viruses, while showing only 50% protection against HSV-2, comparatively Gemcitabine exhibited less protection.

Virucidal assay: When the drugs were incubated with the viruses prior to adding to the plates, the cell protection decreases to 75% and 25% by 5-FU and gemcitabine respectively.

MTT antiviral assay: 5-FU and Gemcitabine exhibited 92.23% and 70.84% cell protection respectively against HSV-1.

Plaque reduction assay: The drugs exhibited a selectivity index of 6.15 for 5-FU and 2.16 for Gemcitabine which confirms antiviral activity of the drugs against HSV-1.

**Conclusion:** Our findings suggest that the anticancer drugs 5-Fluoro Uracil and Gemcitabine show potent antiviral activity against HSV-1 but minimal activity against HSV- 2.

## ISSHID Abstract-220 Engineered filament with anti-HIV drug for 3D Printing of personalized medicine

### Usha Nandhini V, Priya Dharshini K, Malarvizhi K, Ramyadevi D, Vedha Hari BN

#### School of Chemical & Biotechnology, SASTRA Deemed University, Thanjavur, Tamil Nadu, India

**Background:** Over 36 million people are affected by HIV, among which 1.8 million are children under the age of 15 years. Several limitations are reported in pediatric antiretroviral therapy such as patient incompatibility, high pill burden dose, drug resistance, and side effects. To overcome these factors, personalized medicine is recommended as one of the appropriate choices of drug administration. The 3D printing technique can be utilized to develop suitable unit dosage forms with required size and shape containing an optimum dose of the drugs according to the need of the individual patients.

**Methods:** The anti-HIV drug Efavirenz (200 mg) loaded 3D printing filament was developed by the ionic gelation technique using 5% sodium alginate, 3% gelatin, 3% hydroxypropyl methylcellulose, 3% microcrystalline cellulose and 3% PVA in presence of calcium chloride with acetic act as a cross-linking agent. The prepared filament was characterized for its physicochemical properties such as viscosity, thickness, diameter, shape, hardness, drug content, SEM, FTIR, TG-DSC to confirm the stability and suitability for fused deposition 3D printing.

**Results:** Efavirenz loaded filament was found to be linear in structure with a length of 102cm, a thickness of 38.5±4.775mm with 5.33 mg drug content. The FTIR analysis confirmed the stability of the drug in the filament. TG-DSC showed the melting of the filament between 190-200oC.

**Conclusion:** The anti-HIV drug-loaded filament was developed successfully for the first time, with required physicochemical properties suitable for the development of personalized medicine through 3D printing technology with the desired dose.

## ISSHID Abstract-221 Metal complexes with terpolymer ligand containing benzimidazole scaffold enhancing antimycobacterial efficiency

### N. Vijayan^1,2^, S. Johnson Raja^3^, R. Princess^4^

#### ^1^Research and Development Centre, Bharathiar University, Coimbatore, Tamil Nadu, India; ^2^Department of Chemistry, Solamalai College of Engineering, Madurai–625 020, Tamil Nadu, India; ^3^Department of Chemistry, The American College, Madurai, Tamil Nadu, India; ^4^Department of Biotechnology, Mepco Schlenk Engineering College (Autonomous), Sivakasi, Virudhunagar District, Tamil Nadu, India

**Background:** Tuberculosis is one of the most dreadful disease pushing problem on human health worldwide. In order to overcome the existing improper therapeutic strategies, the researchers motivated towards metal polymer frameworks for anti-TB. Several polymers and terpolymers and their metal complexes have been prepared and exhibited very pronounced antibacterial, antitumor, antiviral, antifungal, and drug delivery properties. In recent years, profound research efforts on the synthesis and characterization of metal-organic coordination polymers has led to significant advances in pharmacological activity. The objective of this work was concentrated on the synthesis, characterization and antimycobacterial activity of metal complexes (Cu(II), Ni(II), Co(II) and Zn(II)) with terpolymeric ligand (obtained from substituted benzimidazole, formaldehyde and aminophenol).

**Methods:** The MIC values were measured for metal complexes containing terpolymeric ligand against M. tuberculosis strain H37Rv using MABA method and compared with ethambutol (standard).

**Results:** The observation was noted as MIC values of prepared metal complexes (3-10 μg/ml) are greater than the drug (1 μg/ml), Ethambutol.

**Conclusions:** The copper complexes with terpolymer showed higher antimicrobial activity than other complexes and ligand due to higher thermal stability and lipophilic nature of complex facilitates the diffusion into the cell membrane & subsequently induces cell destruction through the intracellular reduction of Cu(II) to Cu(I) species lead to the activation of oxygen which may be disastrous for microbial strain. In the present investigations, the the prepared copper exhibited improved activity as compared with known standards and may also be used as antiTB agents after clinical assay.

## ISSHID Abstract-223 Cytotoxicity and in-vitro performance of Valacyclovir-Polycaprolactone nanocrystals as novel carriers for antiviral drug delivery

### Ramyadevi D, Veesam Gnana Kusuma, Vedha Hari BN, Shruthi S

**Background:** Virus infections have been recognized as outrageous conditions prevailing among wide populations, especially the human immune deficiency virus, herpes virus and influenza virus, which requires the most competent handling and healing resources. The entry of nanocarriers in drug delivery has potential effects in sustaining the drug action and targeting. The purpose of the study is to design nanoparticulate drug delivery for an antiviral pro-drug Valacyclovir to improve its bioavailability and half-life, and decrease the dosing frequency which ensures noteworthy improvement in the antiviral therapy.

**Methods:** Valacyclovir loaded polycaprolactone nanocrystals were developed by double emulsion method in presence of poly-vinyl alcohol as surfactant and optimized by 3^2 factorial design. In-vitro characterization, drug release and cytotoxicity (cell line) studies were carried out to check the efficiency of the designed nanoparticles.

**Results:** The nanocrystals were obtained in the size range of 90.2–536 nm with drug entrapment efficiency of 65–100%. The characterization studies by FT-IR, DSC and XRD revealed compatibility of drug with polymer and increased crystallinity with degree of crystallinity as 48° and melting temperature of 215°C. The in-vitro drug release profile showed sustained drug release of 49% at 8 h for the optimized formulation, which followed Higuchi model (R^2=0.9921) release kinetics. The in-vitro cytotoxicity studies of the nanoparticles on NIH-3T3 cells proved negligible toxicity at low dose and exhibited time and dose-dependent toxicity at 62.5–1000μg/mL concentrations.

**Conclusion:** The formulated nanoparticles proved its efficiency for sustained drug release without significant cytotoxicity, thereby suitable to provide improved anti-viral therapy.

## ISSHID Abstract-224 Innovative discovery of a novel agar infused with *Capsicum annuum* – Chilli agar

### Nirmala B, Suganthi P

#### Department of Microbiology, Dr.ALM PGIBMS, University of Madras, Taramani, Chennai, India

**Background:** Green chilli consists of protein, carbohydrate, fibre, calcium, potassium, sodium, ascorbic acid, and capsaicin. The objective is to find new cheap medium to grow bacteria and fungi using green chilli. This study shows the ability of the organism to grow in the minimal requirement of chilli and against capsaicin.

**Methods:** Chilli agar was made using oven dried green chilli powder with 2% agar. 1% and 2% chilli agar were used for bacterial and fungal cultivation respectively. The pH was adjusted to 7 for bacteria and 5.6±0.5 for fungi. Fungal and bacterial cultures were inoculated onto the medium and growth were observed. In addition, cresol red dye was added to the medium, which detects the alkaline byproducts of the organism by the change in the color of the medium due to change in the pH.

**Results:** Of 10 bacterial cultures (*E.coli, K.pneumoniae, B.subtilis, S.aureus, P.aeruginosa, p.vulgris, S.typhi h, S.dysentriae, E.faecalis, Citrobacter*) *E.coli, K.pneumoniae, B.subtilis, S.aureus, P.aeruginosa*, and *Citrobacter* were found to be grown.

And of 18 fungal cultures(*Aspergillus niger, A.fumigatus, A.flavus, A.terreus, A.brasiliensis, Mucor, Rhizopus, Curvularia, Alternaria, Penicillium, Fusarium, Syncephalastrum, Paecilomyces, Absidia, Candida albicans, C.tropicalis, C.krusei, C.parapsilosis*) all were found to be grown.

**Conclusion:** From this study, it is evident that Chilli agar can be used as an alternative mycological as well as bacteriological medium for the purpose of cultivating microorganisms

## ISSHID Abstract-226 Genotypic detection of vancomycin resistant Neisseria polysaccharae in acute febrile illness – First report from South India

### M.K.Janani^1^, K. Santhanalakshmi^1^, L. Dhanurekha^1^, B. Aarthy^2^, H.N. Madhavan^1^

#### ^1^Sankara Nethralaya Referral laboratory, unit of Medical Research Foundation, Chennai; ^2^SRM Institute of Medical Science (SIMS) Hospital, Chennai

**Background:** Nongonococcal, Nonmeningococcal Neisseriae are part of the normal flora in human. Frequently, the presence of these bacteria in the blood has been associated with severe infections. This study was conducted to ascertain the causative agent of the acute febrile illness (AFI) with fulminant sepsis and disseminated intravascular coagulation.

**Methods:** Three consecutive blood cultures were performed showed positive signal and the subsequent isolation of gram negative diplococci in culture. The phenotypic characterization was performed by conventional biochemical tests and commercially available Vitek 2 compact. 16S bacterial rRNA gene was amplified by the polymerase chain reaction (PCR) followed by Sanger sequencing. The sequence of the PCR products were validated with Bioinformatics methods such as NCBI-BLAST and metagenomics databases.

**Results:** All three blood cultures initiated upon admission were positive. The smear morphology resembled Neisseria meningitidis. However, the isolate was not identified by the Vitek2 and MALDI. The antibiogram also showed resistance to Vancomycin, Penicillin and co-trimoxazole. BLAST analysis with 16S rRNA showed a high similarity with Neisseria polysaccharea and the sequences were submitted in the genbank (accession numbers: MN394393, MN394394 & MN394395). Further, the comprehensive comparison with antibiotics resistance database revealed the presence of 16S rRNA mutation conferring resistance to spectinomycin.

**Conclusion:** The detection of normal respiratory flora (infrequent infectious agent) in the patient’s blood samples could be an alarming serious infection. A detailed interaction of these pathogens in the blood need to be further characterized to explore the targeted therapies and appropriate treatment.

## ISSHID Abstract-228 Dengue in pregnancy

### Nidhi Sharma^1^, Archana Prakash^2^, Shanthi Ethiraj^1^, Hussaina Sheikh^1^, John Smith^1^, Susan Jones^2^

#### ^1^Department of Obstetrics and Gynaecology, Saveetha University, Chennai, India; ^2^Department of Physiology, Saveetha University, Chennai, India

**Background:** Dengue infection at term gestation carries a risk of hemorrhage for both mother and newborn. There is also a serious risk of fetal death, premature birth and vertical transmission

**Case Study:** A 26years old pregnant lady, second gravida at 38 weeks of gestation, with previous caesarean section delivery presented with fever and cough for 3 days. On examination, her general condition was s

with temperature recording 102 degree F and on chest auscultation right basal crepitations were heard. She had haemoglobin 10.6 g/dl, total leucocyte count- 21,700/cubic mm, platelet count 3.66 lakhs/cubic mm. Dengue IgM was positive .Patient was monitored by tempreture, pulse, respiratory rate and input output charting. Intravenous paracetomol, antibiotics, normal saline and nebulisation were given. Elective caesarean section was done in afebrile phase after 48 hrs of stabalising, due to previous caesarean section. Patient delivered an alive, boy baby with APGAR 8/10, 9/10. Birth weight was 2.895 kgs. In postoperative period baby and mother were well. Informed written patient consent in local language was obtained for the publication of this case report.

**Conclusion:** Severe bleeding may complicate delivery and/or surgical procedures performed on pregnant patients with dengue during the critical phase, i.e. the period coinciding with marked thrombocytopenia with or without plasma leak. Prophylactic platelet transfusion has no role. This case has been reported for its management and fetal outcome. A high index of clinical suspicion is essential in any pregnant woman with fever during an epidemic. In this case study meticulous management resulted in good outcome.

## ISSHID Abstract-231 Protein profiles of *Sterptococcus mutans* isolated from oral cavity of adult coffee drinkers

### Thota Akhil Raj, Iswarya A, Thangam Menon, Prabu D

#### Department of Microbiology, University of Madras Taramani, Chennai, India

**Background:** The primary habitat of *Streptococcus mutans* is the oral cavity and it is known to play a role in the etiology of dental caries. Recent studies have shown that coffee consumption can alter the human microbiome. The present study was aimed to investigate the prevalence and protein profiles of *S.mutans* isolated from oral cavity of coffee drinking and non-drinking healthy adults.

**Methods:** A total of 24 oral rinse samples were collected from healthy adult population (coffee drinker (n=20) and non coffee drinker (n=4) ). Mitis Salivarius Agar was used to isolate and identify the *S.mutans* in the oral samples. Standard biochemical tests were performed to confirm *S.mutans*. Protein was extracted and estimated by Lowry’s method. Protein profiling was determined using SDS-PAGE analysis.

**Results:***S.mutans* was identified in 8 oral samples, of which 4 were from coffee drinkers and 2 were from non-coffee drinkers. SDS-PAGE analysis showed similar protein profiles for the *S.mutans* isolated from both the coffee drinkers and non –coffee drinkers.

**Conclusion:** In our study, *S.mutans* was identified in both coffee drinkers and non-coffee drinkers and isolates in both groups had similar protein profiles.

## ISSHID Abstract-233 Dengue serological profile and its geographical distribution from a tertiary care hospital, Mysuru, South India

### Monisha.B, M Raghavendra Rao, Krishna Karthik.M, Vidyavathi.B.C, Sowmya.G.S, Rashmi P Mahale

#### Dept of Microbiology, JSSAHER, Mysuru, India

**Background:** Dengue fever is one of the most common acute viral illness associated with considerable morbidity and mortality. Recently, there is an alarming rise of dengue in India.It is difficult to trace out the incidence of the infection which is circulating in Mysuru district, South India.Hence we have adopted a method to know the maximum number of cases reported.

**Methods:** Prospective study conducted over a period of 1 year (June 2018-July 2019)included a database obtained for 4172 patients of suspected dengue fever who were admitted to JSS Hospital, Mysuru.Dengue serology was done for all suspected cases by ELISA.Detailed demographic details were taken to construct geographical maps of disease and deprivation.

**Results:** Of 817 positive dengue cases, 584 were Ns1 positive (71.4%),150 were IgM positive (18.3%),and 38 were both Ns1 and Ig M positive(4.6%). Dengue positivity was considerably high during monsoon period (June to August)of the year 2018 and 2019.Majority of the clusters were observed in Krishnarajanagara taluk and urban areas of Mysuru district. And also the range of total leukocyte count, packed cell volume and platelet count were 5, 946 , 40.84 and 0.844 respectively.

**Conclusion:** The finding of increased number of dengue cases during the three months (June to august) of the year should be useful in hospital planning and management. In order to reduce the burden of dengue cases and its mortality rate, increased community awareness and vector control measures need to be strengthened during monsoon period (June to August), especially in Krishnarajanagara taluk and urban areas of Mysuru district where there was an outbreak .

## ISSHID Abstract-235 Small Molecule Inhibitors for HIV-1 Nef and Host Co-stimulatory receptor interaction

### Anusmrithi U. Sharma, Shweta Sharma, Taslimarif Saiyed, Parvinder Pal Singh, Ram Vishwakarma, Anandi S. Karumbati, Satyajit Mayor

**Background:** HIV/AIDS remains to be one of the world’s foremost health challenges. With anti-HIV-1 drugs rapidly becoming ineffective unless administered in multi-drug combinations due to viral resistance there is a need for alternative therapeutic strategy. HIV-1 Nef protein is expressed during early phases of viral replication and helps in maintaining a constant state of infection, thereby playing a pivotal role in pathogenesis and AIDS progression. In infected APCs, Nef protein binds and internalizes the host B7 co-stimulatory CD80/CD86 surface receptors, leading to loss of T-cell activation, hence circumventing immune clearance of the infected cells.

**Methods:** Small peptides mimicking the cytosolic tails of CD80/CD86 were able to reverse the Nef effects, hence establishing this protein-protein interaction as a potential target. Based on this target, small chemical molecule from a focused library were tested for disruption of Nef-CD80/CD86 interaction in biochemical assay as well as in phenotypic functional T-cell activation and viral assays.

**Results:** Active molecules belonging to 3 scaffold series namely AP, PA and BC were identified from biochemical and cell-based viral infection assays. To begin with, the ‘AP’ scaffold was selected to initiate Hit to lead optimization study and we came up with a few active compounds. In preliminary PK and solubility studies, these molecules showed very good solubility, AUC and Cmax values showing druggable potential. Currently lead optimization of molecules and understanding the structural biology aspects of the target are in progress.

Conclusions: Based on our studies, ‘AP’ series candidates show attractive therapeutic potential for HIV-1 drug discovery program.

## ISSHID Abstract-236 Fabrication of Drug Eluting Buccal Film by 3D Printing Technology Loaded With Anti-HIV Drug

### Priya Dharshini K, Ramyadevi D, Vedha Hari BN

#### School of Chemical & Biotechnology, SASTRA Deemed University, Thanjavur, Tamil Nadu, India

**Background:** In healthcare, 3D printing technique is primarily used in different fields such as multifunctional drug delivery systems, dental implants, skin/tissue regeneration, orthopaedics, etc. For the first time, coupling of two techniques namely, 3D printing and Dip Coating is investigated in the present study to produce oral disintegrating buccal films (ODFs) for anti-HIV therapy in paediatrics / geriatrics and bed ridden patients.

**Methods:** The ODFs loaded with anti-HIV drug Dolutegravir were prepared by fused deposition modelling (FDM) from polyvinyl alcohol filament with or without a coating layer. The ODFs were characterized for their physico-chemical properties using TG-DSC, FTIR and XRD analysis. The drug was incorporated in the hydroxy propyl methyl cellulose (HPMC-5cps) and Eudragit E100 solution for dip coating process to improve the drug content and provide immediate release effect.

**Results:** The drug release rate was dependent on the polymeric material of the ODF, thickness of the ODF and the presence of the coating layer. The drug content of the uncoated ODF was 4.0±0.02mg, whereas the coated ODF displayed 5.0±0.03, 6.0±0.02, 7.0±0.04mg of drug for 1.5mm, 3.0mm, 4.5mm thickness, respectively. The minimum disintegration time of the ODF in the simulated salivary fluid was 5 hours. The ODF without coating layer showed faster drug release rate compared to the coated ODF.

**Conclusion:** The Dolutegravir loaded oro-dissolving film was successfully developed by 3D printing technology and coated with polymers. 3D printed drug delivery system loaded with optimum dose of Dolutegravir will pave the way for personalized medication needed for the individual patient.

## ISSHID Abstract-237 Zinc complexes with 4-aminoantipyrine derivatives as potent antituberculosis agents

### C. Justin Dhanaraj

#### Department of Chemistry University College of Engineering, Nagercoil, Tamil Nadu, India

**Background:** One of the major causes of death in the world like HIV is Tuberculosis. Around one third of the world population is thought to be affected by the agent mycobacterium tuberculosis. It is identified as one of the leading infectious disease. Due to the resistance offered by M. tuberculosis to different drugs, development of novel anti TB drugs is of urgent priority in this crucial time. There is also alarming increase in several new cases with multi drug resistant TB. Owing to high biological activity of 4-aminoantipyrine derivatives, these may offers as potential candidate towards the treatment of tuberculosis.

**Methods:** Agar well diffusion method is adopted to evaluate the antimycobacterial activity. Standardized inoculums of the test organism were spread uniformly on the surface of the plates using sterile cotton swab. Mycobacterium smegmatis was compared with gentamycin as standard. DMSO was used as negative control.

**Results:** On comparing with MIC value of standard, the tested Schiff base and mixed ligand zinc complexes exhibited MIC values in the range 10-14 μg/ml, indicating high antimycobacterial activity

**Conclusion:** Among the metal complexes studied, the 4-amino antipyrine derived mixed ligand zinc complex exhibits higher antimycobacterial activity. The higher antimycobacterial activity of the mixed ligand zinc complex is due to the presence of additional biologically active pharmacophore. Drugs with different pharmacophore act at more than one target and effective for treating drug resistant infectious agents. So the 4-aminoantipyrine derived mixed ligand zinc complex may serve as promising agent to treat tuberculosis.

## ISSHID Abstract-239 Antimicrobial and antioxidant activity of Resveratrol Nano-dryemulsion developed by spray drying technique for improved efficacy against methicillin-resistant bacteria

### S.Punitha^1,2^ K.Ruckmani^1,2^

#### ^1^Department of Pharmaceutical Technology, University College of Engineering, BIT Campus, Anna University, Tiruchirappalli, Tamil Nadu, India; ^2^The National Facility on Drug Development (NFDD), University College of Engineering, BIT Campus, Anna University, Tiruchirappalli, Tamil Nadu, India

**Background:** Antimicrobial resistance has led to the evaluation of other potent microbial agents. Resveratrol possesses acclaimed health – beneficial effects and its use is limited due to low oral bioavailability. The objective of this study was to assess the antibacterial activity of resveratrol through dry nanoemulsion formulation and characterize its physicochemical properties

**Methods:** The o/w type Resveratrol Nanoemulsions (liquid) were prepared with capryol 90 as an oil (2.5 %), labrasol as a surfactant (11.7 %), ethanol as co-surfactant (5.8 %) and water (80 %). Subsequently spray-dried at 150° C. The spray-dried nanoemulsion was evaluated for its physicochemical properties, in-vitro release studies, TG-DSC analysis, SEM, XRD analysis. The antibacterial activities, performed by disc diffusion method using tetracycline as a standard against clinical isolates of methicillin-resistant S. aureus (MRSA), *E. coli* and *P.aeruginosa*. The antioxidant activity, performed by DPPH assay.

**Results:** Dry emulsion exhibits higher drug release rate due to reduced particle size < 100 nm, appeared with dents, agglomerated spherical shaped particles, lack of melting endothermic peak and absence of crystalline diffraction peaks. The plain Resveratrol nanoemulsion showed antibacterial activity at 1000 μg/mL concentration against MRSA bacteria. Also, the Resveratrol nano-dry emulsion showed improved antioxidant activity to that of the plain drug.

**Conclusion:** Spray drying technique improved Resveratrol solubility through a solid-state transition tor improved bioavailability. Since the clinical pathogens are more virulent, requires a higher concentration of Resveratrol to act against MRSA pathogens.

## ISSHID Abstract-240 Utility of Xpert® Carba –R in identifying carbapenem resistance in blood culture isolates in critically ill patients

### Rakshith.V^1^, Vidya Krishna^2^, Uma Sekar^3^

#### ^1^Department of General Medicine, Sri Ramachandra Institute of Higher Education and Research, Chennai, Tamil Nadu, India; ^2^Department of Infectious Diseases, Sri Ramachandra Institute of Higher Education and Research, Chennai, Tamil Nadu, India; ^3^Department of Microbiology, Sri Ramachandra Institute of Higher Education and Research, Chennai, Tamil Nadu, India

**Background:** Xpert ® CARBA R (Cepheid, Sunnyvale, USA) is a real-time PCR test for detection and differentiation of carbapenamase producing genes like KPC, NDM, VIM, OXA-48 and IMP in 48 minutes. It is well studied for detection of colonization with carbapenem resistant gram negative organisms (CROs) in rectal swabs/ feces to facilitate early isolation of such patients. However, there are fewer studies on the use of Xpert ® CARBA R in blood culture isolates to enable early identification of CROs to guide empiric treatment with Polymyxins. We aimed to study the utility of Xpert ® CARBA R to identify CROs in blood culture isolates.

**Methods:** Retrospective study of cases in whom Xpert Carba R had been done on gram negative blood culture isolates during the period between September 2018 to August 2019.

**Results:** Xpert ® CARBA R was done on 26 critically ill patients during the study period. Of the 28 blood culture isolates, 19 were CRE ( E.coli 8, Kleb. pneumoniae 11). Sensitivity and specificity of Xpert ® CARBA R to detect carbapenem resistance was 58.8% and 72.7% in all isolates and 83.3% and 71.4% in CRE isolates. In concordant cases, average turnaround time for Xpert CARBA R was 2.12 days vs culture at 3.75 days. Resistance genes in CRE were OXA-48 (9), NDM (3) and both(3).

**Conclusion:** Xpert Carba R detects carbapenem resistance in Enterobacteriaceae earlier and faster than cultures in critically ill patients and can help guide empiric therapy. It does not detect carbapenem resistance in non-enterobacteriaceae.

## ISSHID Abstract-244 Prevalence of Hepatitis C and Human immunodeficiency virus co-infection in India: A systematic review and meta-analysis

### Appakkudal R Anand^1^, Gladys Rachel^2^, Harinee R^1^, Aanand B Sonawane^2^

#### ^1^L & T Microbiology Research Centre, Vision Research Foundation, Chennai, India; ^2^HIV/AIDS Division, Department of Clinical Research, National Institute for Research in Tuberculosis, Chennai, India

**Background:** Though India contributes significantly to the worldwide burden of both Human Immunodeficiency Virus (HIV) and Hepatitis C virus (HCV), little is known about the extent of HCV-HIV co-infection in India. The aim of this review was to determine the prevalence of HCV in HIV-infected individuals in India and identify the main risk factors associated with co-infection.

**Methods:** We performed a systematic review of indexed (Pubmed and Embase) studies reporting HCV prevalence in HIV-infected populations in India. We estimated pooled prevalence with a DerSimonian-Laird random-effects model. Data were further stratified by region, risk group, HIV status and year of publication.

**Results:** We included 45 studies from all over India, comprising 51 separate cohorts. Among low-risk groups, the HCV prevalence in HIV-infected individuals was 3.15% overall, with the highest prevalence (6.18%) in blood donors. Among high-risk groups, the HCV prevalence in HIV-infected individuals was 67.36% overall, with the highest prevalence (71.86%) in injection drug users (IDUs). Meta-analysis revealed that the odds of HCV infection were 4.38 times higher in HIV-positive than their HIV-negative counterparts.

**Conclusion:** We noted a consistently higher prevalence of HCV in HIV-positive than HIV-negative individuals across all regions in India. However, the prevalence of HCV in HIV-positive populations was highly variable depending on the risk groups, with IDUs representing the major risk group. The study underscores the need for larger country-wide population surveys to assess the prevalence of HCV across different HIV-population groups in all regions.

## ISSHID Abstract-248 Intensified Tuberculosis case finding using expanded clinical algorithm among HIV infected patients attending an antiretroviral therapy centre in Puducherry

### Karthiga Vijayakumar^1^, Palanivel C^1^, Gautam Roy^1^, Gokul R^1^, Divya Nair2

#### ^1^Department of PSM, Jawaharlal Institute of Post-graduate Medical Education and Research (JIPMER), Puducherry; ^2^Program Officer,The INCLEN Trust International, New Delhi

**Background:** Tuberculosis is one of the leading causes of mortality among PLHIV. WHO recommends regular screening for active TB disease of PLHIV using expanded clinical algorithm (current cough, fever, night sweats and weight loss). With presence any one of the four symptoms, presumptive TB is diagnosed and subjected to TB evaluation. A longitudinal study was conducted to estimate the proportion of presumptive TB patients, to estimate the incidence of TB, to estimate the proportion initiated on anti-TB treatment (ATT) among patients diagnosed with TB and to assess reasons for drop-outs from diagnosis to initiation of ATT.

**Methods:** A record review was carried out at the ART centre of tertiary care hospital in Puducherry during July 2016 to June 2017.All PLHIV attending the ART centre were subjected to TB screening at every visit and if suspected then TB evaluation was done.

**Results:** Out of 850 PLHIV, 190(22.4%) participants had presumptive TB symptoms. Cough (91%) is the most common presumptive symptom. Of the 190 symptomatic individuals, 136 (62.8%) were referred for evaluation of TB and 34 were diagnosed with TB. The incidence density of TB was 6.4 (95% CI 4.6-9) 100 person-years. The reasons for drop outs were unaware of TB HIV coinfection, time constraints, confidentiality disclosure.

**Conclusion:** Though the incidence of TB among PLHIV was high not all symptomatic patients undergo TB evaluation procedure. Concomitant use of IPT along with ARV reduces the incidence of TB among PLHIV which should be implemented all over the country.

## ISSHID Abstract-249 Dermatophyte infection confined to the male external genitalia – an unusual presentation

### Sukesh Gautam S, Krishnakanth M, Cordelia Babitha, Anuradha Priyadarshini, Adikrishnan S, Gayathri Rajesh, Murugan S, Anandan S

#### Department of Dermatology, Venereology and Leprosy, SRIHER, Chennai, 600116, India

**Background:** Dermatophytosis of the penis and scrotum is regarded as rare since the days of Hebra, and the same opinion has been expressed till recently by others. Reduced eccrine sweat secretion in the penile skin have been proposed as mechanism for the relative resistance of dermatophyte infection in the male external genitalia. Few such cases have been reported from the tropical countries.

Case Report: A 24-year-old male presented with itchy lesion on the genitalia for one month. On examination, there was a polycyclic scaly patch involving the glans penis and prepuce with raised erythematous border and central clearing.

Another 28-year-old male presented with lesions on the penis since 1 week associated with itching. On examination the patient had two discrete erythematous well-defined patches over the penile shaft and the preputial skin each measuring approximately 3x3 cm.

Differential diagnoses considered were herpes genitalis, fixed drug eruption, plasma cell balanitis and irritant dermatitis. Skin scraping was done from the lesions of both the patients, scales collected, 10% KOH was added and fungal elements were visualised under light microscope. The patients have given consent to the publication of their personal data.

**Conclusion:** Dermatophyte infection of external genitalia without accompanying groin involvement is rare and is often misdiagnosed as other dermatoses. Currently tinea genitalis is also being looked upon as a minor sexually transmitted disease but enough evidence of the same is lacking. Hence a high index of suspicion is required to identify and treat the dermatophyte infection without much hassle.

## ISSHID Abstract-250 Unusual cause of unilateral lower limb gigantism

### Bushra Muna, Anuradha Priyadarshini, Sudha Rangarajan, Adikrishnan, Anandan.S, Krishnakanth, Mahalakshmi, Murugan

#### Department of Dermatology, Sri Ramachandra Institute of Higher Education and Research, Chennai, India

**Background:** Unilateral lower limb gigantism is a rare presentation to Dermatology OPD. The usual differentials include syndromes of the likes of Klippel-trenaunay syndrome. Here, we present a rare case of Subcutaneous Zygomycosis manifesting as unilateral gigantism.

Case report: A 55 year old female presented with painless swelling of right lower limb since 2years duration, starting with thigh & progressed both proximally and distally. A skin biopsy done previously shows granuloma for which patient was empirically treated with ATT for 6 months with no response. She had no co-morbidites.On examination, there was diffuse swelling of right lower limb extending 2cm above inguinal ligament to lower third of calf, induration felt on palpation,with well defined proximal and distal margins.There was no lymphadenopathy. Differential diagnoses considered were Subcutaneous zygomycosis, Diffuse large B cell lymphoma of leg,slow growing sarcoma. Incisional skin biopsy showed suppurative granuloma with foreign body giant cells and eosinophils. PAS &GMS were positive for broad, aseptate hyaline fungal hyphae. Fungal culture was sent. Patient was started on saturated solution of potassium iodide starting with 5 drops thrice a day and gradually incremented with monitoring of thyroid function.the patient responded well to treatment with 50% reduction of swelling over 6 weeks. Patient has given consent to the publication of their personal data

**Conclusion:** Subcutaneous Zygomycosis is endemic in India and is more so concentrated in South India; it is caused by *Basidiobolus ranarum*.Common presentation is subcutaneous indurated plaque which may however rarely resemble elephantiasis,onchocerciasis, tuberculosis and Burkitt’s lymphoma. Unilateral gigantism in this case is a rare presentation of zygomycosis and emphasizes the need for clinical suspicion.

## ISSHID Abstract-252 Phenotypic and genotypic characteristics of Carbapenem - resistant uropathogens in Diabetic and non Diabetic patients

### Aneesha S, P Suganthi, M Anandhi, Swathi M, Latha V

#### Department of Microbiology, Dr ALM PG IBMS, University of Madras, Chennai, India 600113

**Background:** Urinary tract infections (UTIs) are common and occasionally life threatening condition among diabetic patients. Antibiotics are prescribed empirically which may adversely affect antibiotic resistance so far. Carbapenem –resistant is one of the serious problems in diabetic patients. This study investigated the phenotypic and genotypic features of carbapenem resistant uropathogens in diabetic and nondiabetic.

**Methods:** Total 90 urine samples were collected from both diabetic and non-diabetic patients. All the samples were processed by standard microbiologic procedure. Antibiotic susceptibility test were done by Kirby-Bauer disc diffusion method. Carbapenem resistant isolates among MDR were tested to identify carbapenemase producing genes (*blaKPC, blaIMP,blaVIM,blaNDM* and *OXA-48*) by PCR method.

**Results:** Total 84 urine sample with significant UTI, 53 (63%)diabetic and 31(37%)non-diabetic were studied. Total106 isolates were obtained. 65(61.32%) isolates were diabetic and 41(38.6%)non-diabetic. The predominant isolate was *E.coli* 37 (35%).Prevalence of *E.coli* was 21% in diabetic and 14%in non-diabetic. Antibiotic susceptibility test showed that 13(12%) isolates were sensitive to Penicillin and all isolates (100%) were sensitive to colistin group. Total 61 (57.54%) isolates showed multi drug resistance (40.56%diabetic,16.98% nondiabetic) to different antibiotic groups.Gene identification result showed out of 8 carbapenem resistant isolates, 3(37.5%) harboured *blaOXA*-48. All these 3 strains from diabetic patients.

**Conclusion:** This study reported the prevalence of multidrug resistant isolates higher in diabetic than non-diabetic. *blaOXA*-*48* was present in diabetic MDR but not in non-diabetic. Screening of UTI is important for the treatment, prevention of renal complications and control of antibiotic resistance.

## ISSHID Abstract-254 Bioinformatics analysis of microarray data for identifying the differentially expressed host genes in Active tuberculosis (ATB) compared to Latent tuberculosis (LTB) infection

### Mohan R, Luke Elizabeth Hanna, Srikanth P Tripathy, Sudhakar N

#### Department of HIV/AIDS, ICMR – National Institute for Research in Tuberculosis (NIRT), Chetpet, Chennai – 600031. India

**Background:** Our study is aimed at identifying differentially expressed genes (DEG) in Active tuberculosis (ATB) and Latent tuberculosis (LTB) and analyzing the functional differences that result using Integrative bioinformatics analysis.

**Methods:** We reanalyzed the microarray dataset GSE37250 (Malawi and South African cohort) extracted from Gene Expression Omnibus (GEO). Differential host gene expression between ATB and LTB groups was analyzed using the BRB Array Tools. Functional annotation and pathway analysis were performed using DAVID bioinformatics tool and Reactome pathway analysis. Protein-protein interaction (PPI) was analysed using the STRING database and cytoscape.

**Results:** Microarray analysis identified 1117 DEG in the Malawi cohort and 1486 DEG in the South African cohort. Gene Ontology and pathway analysis revealed that the upregulated genes were involved in immune response, inflammatory response, antibacterial humoral response, platelet degranulation, complement coagulation cascades and phagosome function, while the downregulated genes were mainly related to Wnt signaling, inflammatory response, cytokine signaling and B-cell receptor signaling. The resultant PPI network was enriched significantly with p value <1.0E-16, where 76 nodes were connected to 151 edges. The following 19 nodes (FCAR, MCEMP1, CEACAM1, CEACAM8, CLEC4D, GPR84, TLR2, CASP4, CAMP, HP, NAIP, AIM2, SLPI, CR1, DEFA4, CASP5, FCGR2A, FCGR1A and OLFM4) were identified as densely connected regions and also as important hub proteins.

**Conclusion:** The study identified differentially expressed host response genes associated with ATB using bioinformatics analysis. The DEG need to be validated in ATB and LTB samples for potential diagnosis of TB and differentiation of ATB from LTBI and other pulmonary diseases.

## ISSHID Abstract-255 Recombinant Expression of UL111A gene of Human Cytomegalovirus

### P. Durgadevi, Khashpatika Ganesh, K. Lily Therese

#### L&T Microbiology Research Centre, Vision Research Foundation, Chennai, India

**Background:** Laboratory diagnosis of Human Cytomegalovirus (HCMV) from clinical specimens mainly depend on molecular techniques, as the conventional culture is difficult and it requires maintenance of primary cell culture in a well-established tissue culture laboratory. Thus, in case of ocular CMV infection, lab diagnosis is mainly based on Polymerase Chain Reaction (PCR). The availability of positive control for CMV PCR is scarce. This pilot study was to make readily available positive control DNA through the recombinant expression of target UL111A gene of HCMV.

**Methods:** The CMV DNA was isolated from the AD-169 standard strain and the CMV target gene UL111A was amplified by nested PCR (nPCR) using specific primers. The amplified gel product was purified and ligated into ‘pDrive vector’ and transformed by cloning into *Escherichia coli* XL-10 gold. The cloned colonies were selected using ampicillin resistance markers and the gene of interest was identified through restriction enzyme EcoRI.

**Results:** The presence of UL111A gene confirmed by nPCR amplification of first round 234 bp and second round 168 bp size by gel electrophoresis. The sensitivity and specificity of the amplified restriction digested product using CMV primers were confirmed by nPCR. Further, the desired amplified product of the targeted gene was isolated and concentrated for routine HCMV PCR to be used as positive control.

**Conclusion:** This successful pilot study resulted in the availability of “in-house positive control” for performing routine PCR for the detection of CMV in clinical specimens.

## ISSHID Abstract-257 Evaluation of the microbial infections of ear and their susceptibility pattern in a tertiary care hospital

### Anisha Sunil^1^, P.Kennedy Kumar^1^, K.S. Sridharan^1^, S.Neha^2^, L.Somu^2^

#### ^1^Department of Microbiology, Sri Ramachandra Institute of Higher Education and Research (SRIHER), Chennai, India; ^2^Department of Otorhinolaryngology, Sri Ramachandra Institute of Higher Education and Research (SRIHER), Chennai, India

**Background:** Nearly 0.065-0.33 billion people suffer from ear infections leading to loss of hearing in 60% of them. As the middle ear is in close proximity to the brain, infections can lead to intracranial complications. Inappropriate use of antibiotics in these situations can lead to multi drug resistant bacterial strains. Hence ,the knowledge of commonest bacteria causing these infections along with its susceptibility pattern remains a key to unravel the void left in otological microbiome.

**Methods:** A retrospective analysis of samples obtained from middle ear infections were analysed for a period of 4 months (2019) at the Department of Microbiology, SRIHER using Hospital information system. The results of microbiological profile and their susceptibility pattern were tabulated and statistically analysed.

**Results:** Out of 325 samples enrolled, 302 samples grew pathogens (GPC:122,GNB:186,Fungi:17, 23 of them grew more than 1 pathogen).The microbiological profile of 325 pathogens were : *Pseudomonas aeruginosa* 41.8%, *Staphylococcus aureus* 27.6%, CONS 7.6% ,*Candida* species 3.3%, *Klebsiella pneumoniae* 4.6%, *Proteus* species 4.6%,*Streptococcus* species 2.2%,*Escherichia coli* 1.8%, *Aspergillus* species 1.8%, *Acinetobacter* species 1.2%, *Enterobacter* species 1.2%, Citrobacter species 0.9%, *Morganella* species 0.6%, *Providencia* species 0.3%. Multi drug resistant strains were seen in 17 of GNB 9.1% (n=186), predominantly in *Pseudomonas* species 8.8%(n=136). Methicillin resistance among the *Staphylococcus* species was 22%(n=115), predominantly in CONS 64%(n=25). Pan-drug resistance was not reported.

**Conclusion:** Based on our study, a total of 43(13.2%) isolates were MDR strains, hence it is imperative to do a culture and sensitivity pattern of ear infections for efficacious management, thereby reducing further complications.

## ISSHID Abstract-259 Molecular characterization of k1 serotype Hypervirulent *Klebsiella pneumoniae* from clinical isolates

### M Anandhi, P Suganthi, Aneesha S, Swathi M, Latha V

#### Department of Microbiology, Dr.ALM PG IBMS, University of Madras, Taramani, Chennai, India

**Background:** One of the most important causative agent of hospital-acquired infection is *Klebsiella pneumoniae* which cause severe pneumoniae, urinary infection, septicaemia and wound infections. Increased use of antibiotics exposure of K.pneumoniae to different classes of antimicrobial agents leads to emergence of multidrug-resistant. Hypervirulent klebsiella pneumonia (hvkp) is evolved pathotype which cause organ or life threatening infection in healthy individual from community. *Klebsiella pneumoniae* synthesize capsules of 78 different serotypes of which 8 capsular serotype (k1-k8) occurs in hvkp. Of the 8 capsular serotype k1and k2 are most virulent as it prevent phagocytosis. Emergence of carbapenamase producing hvkp is the major public treat presently.

**Methods:** A total 66 *Klebsiella pneumoniae* isolates were included in this work. All the isolates were characterized by antimicrobial susceptibility testing, string test, gelatinase, blood hemolysis and biofilm production. The presence of specific serotype k1, magA gene were detected by polymerase chain reaction (PCR).

**Results:** Among 66 isolates of k.pneumoniae 23(35%) were carbapenem resistant in which 3(13%) were string test positive. Biofilm strongly produced by 5(22%), gelatinase produced by 3(13%), blood hemolysis test positive for 3(13%) and magA gene in two isolates (8.69% : n=23) these strains were MDR including carbapenem and chloramphenicol resistant.

**Conclusion:** Emergence of carbapenem resistant hypervirulent *Klebsiella pneumoniae* posses a serious public health treat which carries and spread asymptomatically life-threatening, invasive disease within the community.

## ISSHID Abstract-260 Unraveling Novel HIV-1 gp41Inhibitors Using In-silico Approaches

### Ishwar Chandra, Sanjeev Kumar Singh

#### Computer Aided Drug Design and Molecular Modeling Lab, Department of Bioinformatics, Alagappa University, Karaikudi, Tamil Nadu, India

**Background:** AIDS is a global epidemic infecting nearly 2 million people every year. The resistance and adverse effects to HAART, currently available therapeutics demand exploration of potent anti-HIV drugs. The binding of gp120 to cellular receptors conformationally changes gp41 into a fusogenic six-helix bundle (6HB) that inserts viral gene into the target cell by fusing the HIV-1 membrane with the cell membrane. The 6HB comprises of three C-terminal heptad repeat (CHR) and trimeric N-heptad repeat (NHR) of gp41 that exists in a coiled-coil and is a susceptible drug target.

**Methods:** Structure-based virtual screening (SBVS) with four small molecule databases like ZINC Natural Product Database (ZNPD), Specs, Asinex antiviral and NCI are employed for identification of potent hits for blocking the HIV-1 gp41 6HB protein functions. Enrichment calculation is used to validate the SBVS. Further, IFD, ADMET, binding free energy and MDS are employed to assess the binding affinity, drug-likeness, and stability of the compounds. All the experiments are conducted with Schrodinger’s software suite.

**Results:** The compound ZNPD_05998785 interacted with the key residues GLN567 in NHR; TRP631 and GLU634 in CHR regions of 6HB protein with docking score of -10.617 kcal/mol and binding free energy of -64.810 kcal/mol. Besides, interaction with GLN563 in NHR and THR639 in CHR regions are also observed. MDS of 60 ns revealed that the compound was stable and interacted with the critical residues of gp41.

**Conclusion:** The hit ZNPD_05998785 binds within the 6HB cavity of HIV-1gp41 protein which can be optimized to get a potential lead.

## ISSHID Abstract-261 Active case finding for Tuberculosis and experience of using mobile phone based data collection in rural, Puducherry – A mixed methods study

### Reenaa Mohan^1^, Ganapathy kalaiselvan^2^, Vinayagamoorthy Venugopal^3^, Vivekananda K^4^

#### ^1^Department of Community Medicine; ^2^Department of Community Medicine; ^3^Department of Community Medicine;^4^Department of Community Medicine

**Background:** Active case finding (ACF) is a key strategy to find them out that helps to achieve elimination of TB by 2025. This activity was to determine the yield of TB cases by community based ACF strategy and to explore the experience of using mobile phone-based application (Epicollect) for community data collection

**Methods:** Community based house-to-house survey was carried out to identify Presumptive TB cases as per the program definition among fourteen villages of Thirubhuvanai, Primary Health Centre by trained postgraduates, medical interns and medical social workers of Department of Community Medicine, Sri Manakula Vinayagar Medical College and Hospital, Puducherry. Mobile based application (Epicollect5) was used for survey. Presumptive TB cases were further followed to confirm the diagnosis of tuberculosis. Free listing and pile sorting was done among interns to explore their experience on Epicollect using Visual Anthropac software

**Results:** Totally 11,644 individuals were covered. Of them majority (75%) were adults and 8% were elderly. Diabetes and hypertension were the commonest comorbidities (12.6%). Tobacco and alcohol were the major addictions (7.8%). Three positive cases were detected from 77 TB suspects. Free listing identified 14 salient variables depending on the cut-off value of 0.083 (Smith’s Salience Score) and subjected to pile sorting. Cognitive map identified their experiences into three categories namely knowledge on TB screening, uses of Epicollect and paper based questionnaire

**Conclusion:** Well-planned ACF done in a systematic manner within the available resources, with proper referral system can help to detect missing TB cases

## ISSHID Abstract-264 Prevalence of *Candida auris* candidiasis in a tertiary care hospital in South India

### Anitha. S, Mary Kiran Danni, Anupma Jyoti Kindo

#### Department of Microbiology, Sri Ramachandra Institute of Higher Education and Research, Chennai, India

**Background:** Over 300 million people suffer from serious fungal-related diseases and fungi collectively kill over 1.6 million people annually. *Candida auris*, the superfungus first described in 2009, ranks fifth of the agents causing candidemia. It is intrinsically resistant to fluconazole and further resistance is shown to develop following antifungal exposure. In addition, difficulties in identification using conventional phenotypic and molecular techniques, the uncertain environmental niches, and the unclear mechanisms of spread have hindered control. Hence, establishing prevalence is vital to the development of appropriate screening and control strategies.

**Methods:** A total of 16 isolates of *Candida auris* were taken up for the study, during the period of one year from August 2018 to August 2019. Conventional mycological methods were performed including direct microscopy, culture and fermentation tests. DNA was amplified and sequenced using ITS1 and ITS4 primers, which amplified the ITS region of the ribosomal subunit. Sequences were aligned and GeneBank BLAST tool searches were performed for species identification. The sequence of the reference strains of candida auris were retrieved from GeneBank and included for the analysis.

**Results:** The genomic sequence of candida auris in the NCBI database were obtained. The data collected was used for creating phylogenetic tree. The evolutionary relationship of species can be assed using this phylogenetic analysis.

**Conclusion:***Candida auris* causes occult, multidrug resistant nosocomial outbreaks in hospitals with a high mortality. Molecular sequencing will help in confirming the species of candida and to improve the infection control surveillance, so that the infection can be curtailed.

## ISSHID Abstract-267 Fungal Infection in a post renal transplant patient with DIAPORTHE: A case report

### Suchitra S^1^, Vichitra K^1^. Lokeshwari G^2^, Balajee G^2^, Chandrasekaran V^2^, Anupama Jyoti Kindo^1^

#### ^1^Department of Microbiology, Sri Ramachandra Institute of Higher Education and Research, Chennai, India; ^2^Department of Clinical Microbiology, Gleneagles Global Health City, Chennai, India

**Background**: Here we report a case of infection caused by a rare fungus Diaporthe. The members of genus Diaporthe belong to Diaporthaceae family. They are filamentous, paraphyletic [4] fungi saprophyte on plants and trees. Diaporthe phaseolorum was first isolated in India from Bacopa monneiri (Brahmi) but it is the first case to be isolated and reported in India from human infection.

**Case:** A 47 year old from Assam went through post liver renal transplantation and was on immune suppressants. Four months after the transplant, the patient noticed a progressive swelling in the right leg . Excision biopsy was done from the swelling and sent in formalin for histopathological examination. Gram stain showed many pus cells and no organism. AFB smear and 40% KOH mount was negative. Fungal revealed growth of a filamentous phaeoid fungi. The isolate was amplified for ITS (18s rDNA) region using fungal specific primers. BLAST sequence analysis of 502bp was carried out, compared and identified as Diaporthe phaeseolorum .

Patient was started on oral itraconazole 100mg twice daily. Two months later while on treatment he presented with a recurrence of swelling in the same sight. The excised tissue grew the same fungi, itraconazole dose was increased to 200mg twice daily and advised regular follow up. Consent was obtained from the patient for the publication of their data

**Conclusion:** Rare fungal infections are emerging in immunosuppressed patients. The clinicians should be made aware to suspect fungal infections in this high risk group patients. This will avoid delay in starting antifungal therapy. As a result morbidity and mortality can be reduced.

## ISSHID Abstract-268 A case of disseminated hydatidosis in a 10 year old child

### Gupta M, Dhole T N, Kishore S

#### SGPGI, Lucknow, India

**Background:** Hydatid cyst disease is a zoonosis caused by cestodes belonging to the genus *Echinococcus*. Of the four species infecting humans, the two important ones are *E. granulosus*, which causes cystic echinococcosis (CE), and *E. multilocularis* (which causes alveolar hydatid disease). Infection in humans leads to the development of hydatid cyst(s) in liver, lungs, brain etc. and represents the metacestode (larval) stage of the parasite.

**Methods:** A ten yr old male presented with history of fever, headache and vomitting. MRI of brain showed well circumscribed mass lesion extending from left perit trigonal area upto posterior frontal cortex. Intra op Hyatid impression was made and cyst excised. Microbiological confirmation was made by demonstration of Scolices, brood capsules and hooklets

An USG abdomen showed hydatid cyst in right posterior liver segments and xray revealed cystic lesion in base of left lung. CECT showed two cysts 7.3x6.5x4.8 cm in segment 7 encroaching segment 8. Lung cyst measured 2.3 cm x2.2cm.. Informed consent was obtained from the parents for publication of this case report and accompanying images

Results Patient was started on 5 cycles of oral albendazole therapy (three weeks of albendazole and one week gap) with inadequate response and a PAIR-PD was planned. Patient was further given 5 cycles of albendazole therapy and monitored by serial ultrasound.

**Conclusion:** At the end of 14 months partial resolution of cavity was seen and child remained afebrile with no signs of recurrence

## ISSHID Abstract-270 The C-phycocyanin inhibits HIV-1 infection in vitro

### Pratiksha Jadaun, Leila Footoh Abadi, Smita S. Kulkarni

#### Division of Virology, ICMR-National AIDS Research Institute, Pune, India

**Background:** The C-phycocyanin (C-PC) is a phycobiliprotein having various therapeutic functions. However, the effect of C-PC, as a pure protein in anti-HIV studies remains unclear. Thus present study we conducted to evaluate its in vitro anti-HIV1 activity.

**Methods:** In the present study, TZM-bl cells were used for primary screening while confirmation was done in peripheral blood mononuclear cells (PBMCs). Cytotoxicity was determined by MTT assay. Based on CC50 value, sub-cytotoxic concentrations were taken for anti-HIV1 activity. In vitro C-PC activity against HIV-1 (R5 subtype C) was assessed in TZM-bl cells using luciferase gene assay while confirmation was done in activated PBMCs by p24 antigen ELISA assay. The generation of reactive oxygen species (ROS) was detected by the DCF-DA probe and observed through confocal microscopy.

**Results:** The C-PC was found low cellular cytotoxic. The C-PC showed dose-dependent anti-HIV1 activity.

The IC50 values in TZM-bl were ≤0.335 mg/mL while in PBMCs values were ≤ 0.085mg/mL. Further, our results depicted that there was increased fluorescence in virus-infected cells while C-PC treated cells had decreased fluorescence due to its antioxidant activity.

**Conclusion:** The C-PC was found to inhibit HIV-1 and reduce the enhanced ROS generation in HIV-1 infected cells, suggesting C-PC as a promising anti-HIV1 agent. We are here first to report its in vitro anti-HIV1 activity as a pure protein. Further studies are warranted to determine its mechanism of action.

## ISSHID Abstract-273 Fungal keratitis caused by Curvularia lunata

### Krishnapriya R^1^, Suhas Prabhakar^2^, Anupma Jyoti Kindo^1^

#### ^1^Department of Microbiology, Sri Ramachandra Institute of Higher Education and Research, Sri Ramachandra University, Porur, Chennai, India; ^2^Department of Ophthalmology, Sri Ramachandra Institute of Higher Education and Research, Sri Ramachandra University, Porur, Chennai, India

**Background:** Fungal keratitis is a common infection of the eye, mainly caused by *Aspergillus and Fusarium* species. Black moulds have been reported to cause fungal keratitis, but it is comparatively rare. Here we report a case of fungal keratitis caused by *Curvularialunata*.

**Case report:** A 55 year old female patient presented to the ophthalmology out patient department with complaints of redness, pain and watering in her left eye since two days, following fall of a foreign body in the eye. On examination, an ulcer with feathery margins, intact epithelium and stromal infiltration was seen on the inferior aspect of the cornea, extending towards the pupillary region. Scraping was taken from the ulcer and sent for culture and sensitivity, KOH mount, and gram staining. Patient was advised admission, but refused. She was treated with moxifloxacin and cyclopentolate eye drops, lubricants and advised to take the opinion of a cornea specialist. The KOH mount, culture plates, and normal saline mount pointed towards *Curvularia lunata*, hence the specialist added natamycin eye drops to the ongoing treatment. The ulcer had healed well by the fourth follow up. Written informed consent was obtained from the patient for the publication of her personal data

**Conclusion:** Keratitis, whether bacterial or fungal, if left untreated, may lead to perforation. Hence, it is very important to do a direct microscopy to diagnose the aetiology so that specific treatment can be started immediately. Culture helps to know the correct genus and species so that systemic fungal treatment, if required, can be chosen appropriately.

## ISSHID Abstract-274 Clinical profile and outcome of severe pneumonia in children under five

### Krithika P, Padmasani Venkat Ramanan, Shuba S

#### Department of Pediatrics, Sri Ramachandra University, Chennai, India

**Background:** Severe pneumonia continues to be an important cause of mortality and morbidity in children in our country. There is very little data in South India about the short and long term outcome of this condition in children. This baseline data is important to study the effectiveness of preventive strategies.

**Methods:** A retrospective study was done from the medical records of children aged 1 month – 5 years of age hospitalized with a diagnosis of severe pneumonia between Jan 2017 – August 2019. The clinical details, radiological findings and laboratory reports were noted from the hospital records. The data was entered in an excel sheet and statistically analyzed with SPSS software. Test of significance were done and p value was significant < 0.05. Odds ratio was calculated.

**Results:** During the study period, total of 90 cases of severe pneumonia were admitted. Out of these, 35(38.9%) < 1 year, 47 (52.2%)1- 3 years and 8 (8.9%) 4-5 years. The common clinical features were fever, in 66.7%, cough, in 100% and tachypnoea in 100%. Among the identified etiological agents, commonest were respiratory syncytial virus (n=23), followed by streptococcus pneumonia (n=7) followed by H1N1 (n=3). 85.6 % children successfully went home after treatment without sequelae. Children who died inspite of treatment were 11 (12.2%).

**Conclusion:** Severe pneumonia in underfive children is more often due to viral illnesses . The mortality continues to be high.

## ISSHID Abstract-278 Leprosy a diagnostic enigma

### Mathumathy. R, Anuradha Priyadarshini

#### Department of Dermatology, Sri Ramachandra Medical College & Research Institute, Porur, Chennai

**Background:** Leprosy is a chronic infectious disease caused by Mycobacterium leprae affecting peripheral nervous system, skin and certain other tissues. It is classified into a paucibacillary tuberculoid (TT) form, a multibacillary lepromatous (LL) form and three unstable intermediate forms, the borderline tuberculoid(BT), mid borderline(BB) and borderline lepromatous(BL) based on clinical features and immunity of host.

Case report: 47 year old male working in leather industry came with complaints of asymptomatic erythematous skin lissesion in moustache area for past one year; he gave h/o hair dye application. He was diagnosed to have contact dermatitis and treated with topical steroids in several other hospitals, which was not of much help and the lesion was gradually expanding. On examination he had an erythematous plaque of size 7cm x 4cm extending from left alae of nose to upperlip, with sparse moustache hair in affected area. Sensory examination, motor examination and peripheral nerve examination was normal. Slit skin smear was negative. Skin biopsy showed a well defined epitheloid cell granuloma, langhan giant cells and was consistent with Hansen’s disease in tuberculoid spectrum. Consent has been obtained from the patient for the publication of his personal data.

**Conclusion:** Even in the post elimination era, we continue to encounter such varied manifestations of leprosy which stresses the importance of having high index of suspicion to diagnose these patients early and treat them adequately to prevent deformities.

## ISSHID Abstract-284 Cold Autoimmune Hemolytic Anemia- Secondary to Varicella infection

### Pratheesh Chandran R^1^, Glennys Carvalho^1^, Dsilva AA^1^, Kevin Manuel^2^, Kingsley S^2^, Basheer A^1^

#### ^1^Department of General Medicine, Pondicherry Institute of Medical Sciences (PIMS), Puducherry, India; ^2^Department of Pathology, Pondicherry Institute of Medical Sciences (PIMS), Puducherry, India

**Background:** Cold agglutinin disease is a rare form of autoimmune hemolytic anemia caused by cold-reacting autoantibodies. Autoantibodies that bind to the erythrocyte membrane leading to premature erythrocyte destruction.It can be primary or secondary. Primary cold agglutinin disease is usually associated with monoclonal cold-reacting autoantibodies. Secondary cold agglutinin disease may be associated with either monoclonal or polyclonal cold-reacting autoantibodies. It can be caused by lymphoproliferative disorders or infections.

**Case report:** A 21 year old female presented with fever, yellowish discolouration of sclera for 2 days following a history of chicken pox a week ago. Lesions present were suggestive of chicken pox.

On initial evaluation she had anemia, indirect hyperbilirubinemia, raised LDH and peripheral smear showed eryhthrophagocyosis and RBC agglutination which reverted at room temperature.Her DCT was positive for C3d which further confirms the cold agglutination. Test for Donath-Landsteiner Antibody was negative thereby ruling our paroxysmal cold hemoglobinuria .All other work up to rule our secondary causes like virology and ANA were negative.

She received multiple warm transfusions. Since hemoglobin levels continued to fall she was started on steroids on day 3.From day six improvement trends was noted in her bilirubin and hemoglobin suggesting resolution of hemolysis. She was discharged on day ten with a tapering steroid schedule and followed up on OPD basis. Full resolution was noted after a week on OPD visit. An informed consent was obtained from the patient for publication of the case data.

**Conclusion:** Since the patient had varicella infection before heamolytic anemia, we came to conclusion of post varicella heamolytic anemia.Very few case reports are reported of this sort in literature.

## ISSHID Abstract-293 HPV in asymptomatic men in the Indian sub-continent

### Anantharam Raghavendran^1^, Arun Jacob George^2^, Ramani Manojkumar^3^, Antony Devasia^2^, Priya Abraham^1^

#### ^1^Department of Clinical Virology, Christian Medical College, Vellore, India; ^2^Department of Urology, Christian Medical College, Vellore, India; ^3^Department of Pathology, Christian Medical College, Vellore, India

**Background:** Unlike infection in women, less is known about HPV infection in men. HPV prevalence in men varies with sampling methods used, nature of tests applied, anatomical site(s) and ranges from 1.3%-73.9%. In India, there is a paucity of information on HPV prevalence in asymptomatic men, who can be reservoirs of infection.

**Methods:** A prospective recruitment of 140 men visiting the Urology clinic with symptoms unrelated to penile disease was done. The study was approved by Ethics committee and informed consent was obtained from particpants. Men with known HIV infection and other sexually transmitted infections (STI) were excluded. Penile swab was collected from urethral meatus and the penile shaft. A WHO validated HPV PCR reverse-hybridization method (PGMY-CHUV assay) was used to identify the HPV infection and genotype. Presence of HPV was correlated with known risk factors.

**Results:** All samples were checked for sufficient DNA prior to analysis. Among the 140 participants, 12 (8.6%) were positive for HPV. Ten (83%) harboured established high risk genotypes (Figure). All were heterosexuals. There was no significant association of HPV with smoking, alcohol use, number of partners, phimosis, circumcision, penile warts and condom use.

**Conclusion:** HPV prevalence in these “asymptomatic” men is lower than some populations but similar to an earlier Korean study. Low prevalence could be related to excluding men with HIV and STI. However, high percentage of risk HPV was detected in those positive for HPV. Large scale epidemiological studies are needed to assess the burden in symptomatic and asymptomatic men in India.

## ISSHID Abstract-296 Sequence analysis of the HIV - 1 envelope proteins in a single patient sheds light onto HIV compartmentalization

### Katamaneni Akhila^1^, Tunuguntla Naga Sai Priyanka^1^, Jennifer Ambrose^2^, Anna Rock^1^, Chirumamilla Krishna Prasad^1^, Wilson Aruni^1^, Daniel Alex Anand^1^

#### ^1^Department of Bioinformatics and The Center for Molecular Data Science and Systems Biology, Sathyabama Institute of Science and Technology, Chennai, India; ^2^PG and Research Department of Advanced Zoology & Biotechnology, Loyola College, Chennai, India

**Background:** Compartmentalization of HIV in the host is transient and correlates the points during infection when the virus is most likely to migrate within the compartments. Compartmentalized viral populations possess different phenotypic characteristics like drug-resistance, cellular tropism and level of pathogenesis.

**Methods:** With the authors’ permission, we used 110 envelope sequences of HIV-1 obtained a single patient from sequential blood sample and various organs obtained at autopsy and the HXB2 sequence as reference. The tools used in this study included Hyphy, MESQUITE, BEAST, BEAUti, Fig Tree, RDP3, and RAT.

**Results:** Of 198 positions, 27 positions were conserved. The Best model in prottest was: HIVw+I+G. The HyPhy Console provided a number indicating the probability that as many or fewer migration events occurred by chance. The probability was 0.9931 and is therefore considered statistically significant. Compared observed migration in Slatkin-Maddison between PBMC to Lymph (0.3151) and PBMC to spleen (0.2974) was higher which might indicate compartmentalization. MP, NJ and Fig trees showed comparable clustering together of Brain and CSF sequences, all liver sequences, lung and kidney sequences, bone marrow and lung, spleen, lung and lymph sequences. RDP3 and RAT ascertained cross – over events between – BM & lung, kidney & lung, lymph node & lung, spleen & lung sequences.

**Conclusion:** Compartmentalization strongly implicates that not all variants of the virus are equally capable of infecting a target cell or tissue and that cell- or tissue-specific adaptation of particular HIV variants is required for efficient replication within a given microenvironment

## ISSHID Abstract-297 Prevalence of *Candida albicans* in pregnant women in South Indian tertiary care hospital and association with preterm delivery

### Sheak Seyed Husaina, K.Jayashree, Nidhi Sharma

#### OBG Department, Saveetha Institute Of Medical and Technical Sciences, Saveetha University, Chennai, India

**Background:** Preterm birth is birth before 37completed weeks of gestation. Genitourinary infections are most frequent in causing preterm births. Preterm births are the main cause of neonatal mortality and morbidity. Our study aims at determining the prevalence of vaginal candidiasis in antenatal women and its association with preterm delivery.

**Methods:** Our study was a prospective observational study conducted in the department of OBG, Saveetha medical college and hospital between NOV 2018 to AUG 2019 over a period of 6 months. A total of 80 antenatal women were studied and after obstetric history taking and clinical examination, high vaginal swab for Gram staining and culture and sensitivity was done. Candida was detected by KOH mount.

**Results:** The prevalence of candida among antenatal women in our study was 31.25%. Majority of women were 22years years of age. Mean gestational age who has gone in for preterm labour are 34.3 +-1.2 weeks. Candida has significantly associated with 10.66 fold risk for preterm labor (odd’s ratio – 10.66) C.I. 3.39-33.54 statistically significant p value 0.0001. Mean birth weight is 2.6+-0.56 in group with candida positive as compared to 2.8+-0.45 in patients with no candida infection.

**Conclusion:** There is a significant association of candidiasis with preterm delivery. Relative risk of candidiasis in preterm delivery was found to be 5.14 [95% C.I. 2.14-12.33] p value 0.0002. The presence of candida was significantly associated with preterm birth. Early detection and treatment of candida will lead to reduced burden of prematurity.

## ISSHID Abstract-298 In-silico docking of brain endothelial receptor against Dengue virus envelope protein

### Durga Bethala, Shashikant Vaidya

#### Haffkine Institute for Training, Research and Testing, Mumbai, India

**Background:** Dengue serotype 2 virus is most predominant in India having wide neurological alterations as a clinical manifestations. The dengue envelope protein (DEP) on the surface of virus facilitates attachment to host cells. It is still unknown, that to which surface receptor of blood brain barrier (BBB) endothelial cells the dengue virus attaches, disturbing tight junction protein complex leading to dengue virus neuroinvasion into our central nervous system.In this study, we used a molecular docking approach to investigate the interactions between Japanese encephalitis virus (JEV) binding BBB receptors against DEP involved in virus entry mechanism.

**Methods:** The DEP ligands was retrieved from UniProt database (AAC59274.1, Phe247-Gly675) and designed using SWISSMODEL server (DEP, 3j27.1.C). All receptor structures were retrieved from the RCSB Protein Data Bank (PDB) database (https://www.rcsb.org). Energy minimization was done using Chiron server (Ramachandran, 2011) on DEP and receptor. The protein structures after energy minimization was validated using RAMPAGE (S.C. Lovell, 2002). Molecular docking was performed using PATCHDOCK and FIREDOCK (Wolfson HJ et al., 2002). The output file was visualized using Dassault Systèmes BIOVIA Discovery Studio Visualizer (v16.1.0.15350)

**Results:** The BBB receptor AX6 (-24.81), PLVAP (-38.2) and VLPAC (-49.60) displayed the lowest docking energy in kcal/mol towards DEP ligand. These receptor showed better docking scores than JEV envelope protein (AX6 -19.56, PLVAP -29.27 and VLPAC-45.13)

**Conclusion:** These results revealed that the PLVAP, ANXA6 and VP13C can be considered as a potential candidate for anti-dengue drug development during in-vitro and in-vivo study

## ISSHID Abstract-299 Are we dealing with melioidosis under the mask of tuberculosis? – A case series

### Malavika Kottarathil^1^, Sudhabharathi Reju^1^, Ramya Barani^1^,Vidhya Krishnaa^2^, Padma Srikanth^1^

#### ^1^Department of Microbiology, Sri Ramachandra Institute of Higher Education and Research, Porur, Chennai, India; ^2^Department of Peadiatric medicine, Sri Ramachandra Institute of Higher Education and Research, Porur, Chennai, India

**Background:** Melioidosis mimicks Tuberculosis and is often misdiagnosed clinically. India has now become endemic to Melioidosis. *Burkholderia pseudomallei* causes abscess in internal organs. Risk factors include diabetes, alcoholism, chronic pulmonary disease, glucocorticoid therapy. The objective of the study is to detect *Burkholderia pseudomallei* by PCR. In this report, we describe a case series of patients showing signs and symptoms similar to Tuberculosis.

**Case Series:**

A 48 year male, known T2DM, presented with multiple joint pain for 7 days involving right knee, right ankle, left knee and left ankle. Right ankle biopsy sample was tested.

A 30 year male, known case of T2DM, presented with fever and cough for 1 month, hepatitis and pancreatitis. Splenic aspirate sample was tested.

A 44 year male, known case of T2DM, presented with intermittent fever, chills, rigor, hepato-splenomegaly and prostatomegaly. Prostate biopsy was tested.

DNA was extracted from the collected samples and conventional nested PCR were carried out to detect the presence of 16S-23S spacer region of *B. pseudomallei* . The amplified product was detected by 2% agarose gel and the presence of 251 bp was considered positive. All three cases were positive for *Burkholderia pseudomallei*.

Culture was also performed and was found to be negative. Written informed consent was obtained from the study participants stating that they have no objection towards the publication of their personal data.

**Conclusion:** Patients suspected for tuberculosis should be screened for B. pseudomallei, especially when samples are negative for Mycobacterium tuberculosis by different methods. Often patients are exposed to antibiotics when they present with fever, hence the need for PCR for accurate diagnosis

## ISSHID Abstract-301 Novel association of dementia with Herpes Simplex Virus-1 (HSV 1)

### Shiny Queensty, Ramya Barani, Sathianathen, Shankar, Padma Srikanth

#### Sri Ramachandra Institute of Higher Education and Research, Chennai, Tamil Nadu, India

**Background:** Globally, 46.8 million people suffer from dementia. HSV1 undergoes periodic reactivation in which the newly formed viral particles reach the trigeminal ganglion; which in turn project to the brainstem and then to the thalamus to finally reach the sensory cortex; establishing a latent infection. Recent evidence suggests that HSV1 reactivation may result in dementia. The aim of the present study is to determine the presence of HSV1 IgM among participants with dementia.

Case report: Blood samples from eighteen study participants with dementia were collected from the participants in psychiatry, neurology & general medicine departments. Institutional ethical committee approval (IEC No: CSP-MED/19/MAR/51/31) was obtained prior to start of the study. Consents were obtained from all of them for the publication of their data. Serum samples were processed and analysed for quantification of HSV1 IgM by ELISA (IBL International, Hamburg, Germany). Lower detection limit of the assay was 10 U/ml. Among the eighteen samples, two tested positive for HSV1 IgM (Patient 1: 13 U/ml; Patient 2: 22 U/ml). Both patients (71 years/male, 76 years/male) presented with loss of recent memory, repeated mouth ulcers, fatigue, mood swings, language problems and trouble sleeping for 6 months and one year respectively. Mini Mental Scale Examination (MMSE) for both were 23/30 and 24/30 respectively, that is early stage of dementia. Since antiviral therapy has found to reverse the dementia; both patients were advised the same.

**Conclusion:** Early detection of HSV-1 will help to alert the clinicians about the association of HSV 1 with dementia, thus enabling them to initiate early therapy in a timely manner which will in turn benefit the patients.

## ISSHID Abstract-302 Co-infection of Hepatitis C virus (HCV) and Epstein Barr virus (EBV) in patient with nasopharyngeal carcinoma - A rare report

### R. Selvambigay, Ramya Barani, Manickavasagam, Padma Srikanth

#### Sri Ramachandra Institute of Higher Education and Research, Chennai, Tamil Nadu. India

**Background:** About 90% of the hepatitis is due to hepatotropic viruses. Herpes virus like EBV plays a major role in alteration in liver pathology and cause acute to chronic hepatitis. We represent the rare case of nasopharyngeal carcinoma with co-infection of EBV and HCV.

**Case report:** A 64-year-old male is known case of cancer in hypo pharynx T2N1M0 on concurrent chemotherapy (3 cycles) and radiotherapy (50Gy cycles). After one-week patient got admitted with complaints of nausea, vomiting, fever and decreased appetite. He is known case of diabetes mellitus on insulin and coronary artery disease on therapy. Had a previous history of jaundice and addiction to alcohol for 25 years. On evaluation, patient was found to be clinically icteric and diagnosed to have hepatitis C virus (378 IU/ml; Genotype 3). Liver enzymes and bilirubin showed increased level (SGOT-1556 IU /L; SGPT-1735 IU/L) following which restarted for RT. Cytomegalovirus blood was below detectable level (<50 copies/ml). Real time quantitative PCR targeting EBV-BNTp143 gene in blood was found to be negative but detected in saliva (13055912copies/ml). The lower detection limit of the assay was <100 copies/ml. Patient was started on antiviral therapy (Tab. Sovihep V 400mg). ENT reassessment showed satisfactory response of hypopharyngeal growth. Consent has been obtained from the concerned patient and explained for the publication of the details

**Conclusion:** Reactivation of EBV occurs during chemotherapy when the immunity is low and increase the level of liver enzymes. EBV and HCV co-infection promotes replication of HCV which may lead to hepatocellular carcinoma. Appropriate diagnosis and optimisation of drug protocol may help to reduce the severity of the disease

## ISSHID Abstract-303 Erythema Mutiforme in a HIV patient - A Clinician's dilemma

### S Harshatha , R Gayathri , S Murugan, S Adikrishnan , R Sudha , V Mahalakshmi, M Krishnakanth

#### Department of Dermatology, Sri Ramachandra Institute of Higher and Research, Porur, Chennai, India

**Background:** Erythema multiforme (EM) is best regarded as a self-limiting cytotoxicity dermatitis resulting from cell-mediated hypersensitivity most commonly to drugs or infection.We herewith report an unusual case of HIV-seropositive male patient with multiple target lesions over the body.

**Case Report:** A 45-year-old male presented to our dermatology opd with complaints of fever and rashes for 4 days which was sudden in onset and progressed to involve whole body involving palms and soles. He is a diabetic under metformin. Vitals were stable.Systemic examination was normal. Dermatological examination revealed an extensive maculopapular erythema with target and targetoid lesions over face, trunk and limbs. Oral and genital mucosa were normal. Baseline investigations were normal. Treatment consisted of oral acyclovir, azithromycin and antihistamines.But the rash was persistent with minimal improvement.Hence serology was done which revealed HIV positivity and ICTC counseling given.After further probing he gave history of unprotected sex with multiple unknown partners in last 5 months.

Skin biopsy features consistent with EM. HIV viral load revealed 1080000copies/mL, elevated CD8 counts,low CD4counts,reversal of CD4:CD8 which indicates acute stage of HIV infection. Lesions subsided after induction with intravenous steroids within 1 week. Patient has given consent to the publication of their personal data.

**Conclusion:** Our patient presented only with targetoid lesion without a background of acute infections,relevant drug history or malignancy.This case highlights the fact that detailed sexual history is mandatory for effective evaluation and management.Hence any patient with atypical rash with suspected viral prodrome should be serologically evaluated for HIV status.

## ISSHID Abstract-305 Evaluation of serological markers with nucleic acid based assay in diagnosis of Hepatitis C virus among chronic Kidney disease patients

### C.Rajasekaran^1^, S.Thasneem Banu^2^, U.Uma Devi^2^, David Agatha^3^, C.S.Sripriya^4^

#### ^1^Department of Microbiology, Kilpauk Medical College, Chennai, India; ^2^Institute of Microbiology, Madras Medical College, Chennai, India; ^3^Department of Microbiology, Chengalpattu Medical College, India; ^4^Department of Microbiology, Stanley Medical College, Chennai, India

**Background:** Hepatitis C virus (HCV) is a major cause of liver disease in patients with chronic kidney disease (CKD). The occurrence of HCV infection in CKD patients is much higher, compared to the general population. The objective of early diagnosis is to start the treatment at the earliest, prevention of complication, to reduce the infectivity and control of transmission. This study was done to evaluate the serological markers with nucleic acid based assay in diagnosis of HCV infection among CKD patients.

**Methods:** This cross section observational study was conducted in Madras Medical College & Rajiv Gandhi Government General Hospital, Chennai between SEP 2015 to AUG 2016. 82 CKD patients on hemodialysis, were subjected to: anti-HCV antibody detection by third generation ELISA, combined capsid antigen- anti-HCV antibody detection by ELISA and RT- PCR.

**Results:** Both ELISA tests were evaluated with gold standard RT-PCR. Sensitivity & specificity of HCV antibody ELISA & combined Ag-Ab ELISA were 63.63%, 100% and 81.81%, 100% respectively. Positive & negative predictive value of HCV antibody ELISA & combined Ag-Ab ELISA were 100% , 94.66% and 100% , 97.26% respectively. The p value <0.001, which was statistically significant.

**Conclusion:** Seroconversion to HCV antibody doesn’t occur early in CKD patients compared to other patients. In CKD patients, HCV serological window period is longer. In CKD patients with poor immune response, antibody ELISA alone is not sufficient for diagnosis of HCV infection. Combined Ag-Ab ELISA can be substituted as a diagnostic test where PCR testing facility is unavailable.

## ISSHID Abstract-307 Association of Hepatitis B virus in Type 2 Diabetes individuals

### Abhinaya Raguramachandran^1^, Monika Mani^1^, Shanthi Vijayaraghavan^2^, Ganesh P^2^, Padma Srikanth^1^

#### ^1^Department of Microbiology, Sri Ramachandra Institute of Higher Education and Research, Porur, Chennai; ^2^Department of gastroenterology, Sri Ramachandra Institute of Higher Education and Research, Porur, Chennai

**Background:** Hepatitis B virus infects hepatocytes where glucose metabolism occurs. There are 40-50 million chronic HBV carriers in India and in Tamil Nadu, about 4.8 million people have T2DM. The association between HBV infection and T2DM is not well established. The aim of this study is to research the association of HBV infection with T2DM. The viral load of HBV infected individuals with and without T2DM were measured. HBV infection may be a possible risk factor for T2DM.

Materials and **Methods:** Blood sample were collected from 30 HBsAg seropositive participants with T2DM and 30 without T2DM. DNA was extracted and real time PCR was performed to estimate the HBV viral load. DNA sequencing was performed targeting HBV polymerase gene (1300 bp) in participants with HBV DNA level above 2000 IU/mL.

**Results:** HBV genotype D2 was identified in all the participants enrolled in the study. Plasma HBV DNA levels of participants with T2DM were found to be higher 6+1.44 log10 IU/mL than the nonT2DM participants. Aminoacid substitutions in polymerase gene of reverse transcriptase domain was identified among the study participants including rtA7T (20%), rtN53D (60%), rtY54H (70%), rtH126R (67%), rtI226R (20%), rtL145M (13%), rtH133K (10%) in the polymerase gene. Among these, rtN53D and rtH126R were specific to genotype D.

**Conclusion:** From this study, we suggest screening for HBV genotypes among HBV infected persons with T2DM to reduce the risk of disease progression and start early appropriate therapeutic solutions.

## ISSHID Abstract-308 A case report of surgical site infection caused by atypical Mycobacterium following laparoscopic Hernia repair

### R. Preethy, D. Aruna, M. Kalyani

#### Department of Microbiology, Saveetha Medical College, Thandalam, Kanchipuram District, Tamil Nadu, India

**Background:** Port site infection is chronic, prevailing complication following laparoscopic surgery. Persistence of wound infections post operatively at surgical sites impair wound healing and patient recovery and if atypical Mycobacteria is the causative agent it becomes even more difficult to manage. The main aim of the study is to increase awareness of atypical Mycobacterial infections, prompt diagnosis, and treatment that may ultimately provide better care to patients.

**Case Presentation:** After getting informed consent from patient we report a case of surgical site infection with bilateral discharging wound sinus in a 54-year old male with history of fever and abdominal pain for six weeks following laparoscopic Hernioplasty. There was no history of any Co-morbid illness and no identifiable immunosuppressive condition. USG showed pus collection around the mesh on both sides, hence USG guided pus aspiration was done and Pus was sent to Microbiology department for routine bacteriological culture and sensitivity, AFB Staining and culture. Bacteriological culture showed no growth. AFB Staining was positive by immunofluorescence. Gene Xpert was negative, DNA-PCR confirmed *Mycobacterium fortuitum*. Wound Debridement, Re-exploration of mesh, along with Amikacin and Clarithromycin resulted in better Wound healing without any Complications. Consent was obtained from the patient for the publication of his data

**Conclusion:** There should be a high index of suspicion based on clinical presentation in patients presenting with delayed post- operative wound infections for the diagnosis of non-tubercular Mycobacteria as causative agents. Treatment involves surgical debridement and administration of combination drugs, which should be continued for a period that will ensure complete healing and prevent recurrence.

## ISSHID Abstract-311 Bullous pemphigoid and HIV - A Rare Association and its Therapeutic Challenge

### Vishu Priya D, Cordelia Babitha, Sivayogana R, Murugan S

#### Department of Dermatology, Sri Ramachandra Institute of Higher Education and Research, Chennai, India

**Background:** HIV related cutaneous manifestations are varied, and skin is one of the most common organ affected. Immunobullous disorder are rarely reported in HIV patients and the treatment is strenuous in view of dysfunctional immune system. Hence we report a rare association of HIV and Bullous pemphigoid.

Case Report: A 50 years female, known case of HIV positive on ART since 2002, presented with itchy fluid filled lesions over face and upper limbs for 10 days. On examination, she had multiple erythematous vesicles and tense bullae over upper limbs, trunk. Histopathology and Direct immunofluorescence were consistent with bullous pemphigoid. CD4 counts > 200 cells/cu mm. Patient was started on steroids and dapsone. Patient was counselled and was advised for surveillance in view of opportunistic infections. On subsequent visit, patient showed improvement. Patient has given consent to the publication of her personal data

**Conclusion:** Bullous pemphigoid should be entertained as a differential diagnosis in HIV patients presenting with fluid filled lesions. Several theories have been proposed for the development of autoimmunity in HIV infection, namely molecular mimicry to a self-antigen, persistent antigenic stimulation, and restoration of the immune system with ART. Treatment of immunobullous disorder in HIV patients includes long-term systemic immunosuppressants. But this poses a therapeutic challenge as the patient have an already dysfunctional immune system. The potential acceleration of opportunistic infections and AIDS-defining illnesses must be balanced against the benefits of treatment. Hence we report this case for its rarity.

## ISSHID Abstract-313 Identification of membrane associated proteins as disease targets in *Klebsiella pneumoniae* by Subtractive Genomics Approach

### Amalareshma A^1^, Sharmila A^1^, Syed Javid S^1^, Mumtaj P^2^, Sasikala S^1^

#### ^1^Department of Microbiology, Presidency College, Chennai-600005, India; ^2^Bioinformatics Infrastructure Facility Centre of DBT, Presidency College, Chennai-600005, India

**Background:** Many microbes turned into multi drug resistant organism over the years consumption of antibiotics. In the last decade, global consumption of antibiotics has significantly increased morbidity, mortality, and health care costs. In community-acquired infections, *Klebsiella* also major causative pathogen and become increasingly resistant to antibiotics. Infections by these stains has become very challenging to treat. The objective of the present study is to identify potential membrane associated proteins as disease targets in *K. pneumoniae* BA1625, which has involved in respiratory and urinary tract infections of human.

**Methods:** In this study, comparative analysis of genomes and proteomes of pathogen and human was made to filter non - homologous proteins in human. Then, essential proteins of the pathogen were studied. In order to annotate the function of the proteins, kegg pathway analysis was performed. Various bioinformatics softwares, databases and tools like CASTp, HIT, PROCHECK, ERRAT, RMSD, RAMPAGE were used for the prediction of sub- cellular localization and membrane proteins.

**Results:** Membrane proteins can serve as novel therapeutic targets, from our study we identified 13 proteins and involved in the specific pathways of *K. pneumoniae* BA1625.

**Conclusion:** Subtractive genomics analysis helps in providing information for a set of proteins which are essential to pathogen but are not present in the host. These target proteins might be considered to study further in detail to design inhibitor for *K. pneumoniae* BA1625.

## ISSHID Abstract-320 Elevated levels of circulating Type 2, regulatory and other anti-inflammatory cytokines in pulmonary tuberculosis and reversal after chemotherapy

### Kadar Moideen^1^, Kumar N P^1^, Ramalingam Bethunaickan^2^, Banurekha V V^2^, Dina Nair ^2^ and Subash Babu^1, 3^

#### ^1^Department of Immunology, National Institutes of Health—NIRT— International Center for Excellence in Research, Chennai, India; ^2^Department of Immunology, National Institute for Research in Tuberculosis, Chennai, India; ^3^LPD, NIAID, NIH, Bethesda, MD

**Background:** Pro-inflammatory cytokines are vital in immunity against tuberculosis (TB). However, the role of Type 2, regulatory and other anti-inflammatory cytokines is poorly understood.

**Methods:** To investigate the role of anti-inflammatory cytokines in pulmonary TB (PTB), we investigated the plasma levels of Type 2 (IL-4, IL-5, IL-13), regulatory (IL-10, TGFb) and other anti-inflammatory (IL-19, IL-27, IL-37) cytokines in PTB, latent TB (LTB) or healthy control (HC) individuals. Further, we investigated the plasma levels of these cytokines in PTB individuals following anti-tuberculosis therapy (ATT).

**Results:** PTB individuals exhibited significantly higher levels of IL-4, IL-13, IL-10, TGFb, IL-19 and IL-27 when compared to LTB and/or HC, whereas PTB individuals exhibited significantly lower levels of IL-5 and IL-37. Bilateral or cavitary disease status did not show significant difference in Type 2, regulatory or other anti-inflammatory cytokine levels when compared to unilateral or non-cavitary disease nor did the cytokines show any significant relationship with bacterial burdens in PTB indidviduals. Finally, following ATT, IL-4, IL-5 and IL-10 levels in the plasma were decreased significantly, while IL-13 and IL-37 levels were increased significantly.

**Conclusion:** Hence, our investigation reports that PTB is associated with enhanced levels of Type 2, regulatory and other anti-inflammatory cytokines, some of which are altered followed chemotherapy. Also, our data suggests that anti-inflammatory cytokines cannot be disease severity markers or bacterial burden markers in PTB.

## ISSHID Abstract-323 Assessment of HIV-1 inactivation following different concentrations of bleach by real time polymerase chain reaction

### Nithish Kumar Balasubramaniyan, Gopalsamy Sarangan, Yazhini Ravi, Monika Mani, Padma Srikanth

#### Department of Microbiology, Sri Ramachandra Institute of Higher Education and Research, Porur, Chennai, India

**Background:** Sodium hypochlorite is highly effective disinfectant against blood borne pathogens. Routinely 1% bleach is used in the spill kit to disinfect spills. Human Immunodeficiency Virus - 1 (HIV-1) is a blood borne pathogen. Real-time PCR is the most efficient method for detection of live and inactivated virus. The aim of the study is to determine the effect of different concentrations of bleach on HIV positive samples using Real Time PCR.

**Methods:** Different concentrations of bleach (1%, 2%, 4%, 6%, 8%and 10%) were used in the study against two samples which were previously tested, in which one had a high viral load (>100000 IU/ml) and another had low viral load (<10000 IU/ml). Equal volume of plasma samples and different concentrations of bleach were mixed and incubated at RT for 30 min. Quantitative Real time PCR was performed to assess the viral load.

**Results:** After treatment of specimens with bleach, low viral load sample (<10000 IU/ml) demonstrated undetectable viral load at all concentrations of bleach. However, in samples with high viral load, complete disinfection was not achieved in 1% concentration whereas other concentrations (2%, 4%, 6%, 8% & 10%) showed undetectable viral load. While using 1% concentration, 98% reduction (3000 IU/ml) of HIV RNA was achieved in high viral load sample (>100000IU/ml).

**Conclusion:** Our data suggests that sodium hypochlorite concentration >2% may be used as a potent disinfectant against blood borne pathogens. It may be essential to revise the current practice of bleach concentration (1%) for disinfection.

## ISSHID Abstract-325 Chronic immune activation among treatment naïve HIV/HBV coinfected individuals

### John P Demosthenes, Gnanadurai J Fletcher, Uday G Zachariah, George M Varghese, Susanne A Pulimood, Priya Abraham, Rajesh Kannangai

#### Christian Medical College, Vellore, Tamil Nadu. India

**Background:** Chronic immune activation is one of the recognized hallmarks of HIV infection. It is identified with CD38 and HLA-DR and cells that express these markers show reduced proliferative potential, signal transduction, and increased apoptotic potential. Chronic immune activation can affect both HIV and HBV pathogenesis and its outcome.

**Methods:** In this cross-sectional study, we have examined the chronic immune activation markers HLA-DR+ (DR) and CD38+ in HIV-1 monoinfected (n=36), HBV monoinfected (n=35), and HIV/HBV coinfected individuals (n=22) using flow cytometry and correlated its effect on HIV/HBV replication using quantitative real-time PCR.

**Results:** Among HIV/HBV coinfected individuals, there was a significant increase in HBV viral load (p=0.002), a higher proportion of HBeAg (p=0.0049) and lower CD4 counts (p=0.04), as compared to monoinfected groups. The frequencies of both CD4+ CD38+ HLA-DR+, CD8+ CD38+ HLA-DR+ in the HIV/HBV coinfection, were significantly higher than HBV and HIV monoinfected groups respectively (p < 0.0001, p < 0.0001) . The cytokines Granzyme, Fas-L, were higher in the HIV followed by HIV/HBV coinfection and HBV. IL-17 alone showed higher levels in HBV compared to HIV/HBV coinfection. The Liver fibrosis score APRI and FIB-4, were higher in the coinfected group compared with HBV monoinfected group (0.67 vs 0.25, p = 0.0085; 3.48 vs 0.98, p = 0.0026) respectively.

**Conclusion:** Our data suggest that HBV probably influences the immune activation of CD4+ and CD8+ T cells, and this might play a significant role in accelerating the disease outcome among HIV/HBV coinfected individuals.

## ISSHID Abstract-328 Analysis of Genome Wide Association Studies (GWAS) in HIV - 1 infection points out the viral connection to HIV- Associated Neurocognitive Disorders (HAND)

### Mary Vincy. J^1^, Nisha. S^1^, Jennifer Ambrose^2^, Wilson Aruni^1^, Daniel Alex Anand^1^

#### ^1^Department of Bioinformatics and The Centre for Molecular Data Science and Systems Biology, Sathyabama Institute of Science and Technology, Chennai, India; ^2^PG and Research Department of Advanced Zoology & Biotechnology, Loyola college, Chennai, India

**Background:** Susceptibility to HIV-1 and the subsequence clinical course after infection varies significantly between individuals due to host genetic variation. Genome-wide association studies (GWAS) have revealed interesting host factors that influence HIV infection, replication and pathogenesis.

**Methods:** The GWAS catalogue was downloaded from NHGRI-EBI. The pertinent parameters as HIV – 1 control, viral set point, HIV – 1 associated dementia, and asymptomatic neurocogntive impairment were tabulated.The reported mapped genes, associated SNPs their strongest risk alleles, their frequencies and associated statistics were obtained. Enrichment Analysis, gene ontology and reactome were used for insights.

**Results:** Of 152652 entries, 124 pertained to HIV. The loci and variants were mapped and 44/124 (35%) of them were in intergenic regions. 107 mapped genes with 114 unique SNPs. and their risk alleles were identified at intronic regions, 3’ and 5’ UTRs as well as at exonic and regulatory regions. rs2523608 was the only SNP found to be a link between control and set point and is found at 6p21.33 on the HLA B locus in the non – coding exon transcript variant. Enrichment and subsequent pathway analysis revealed that Reelin (RELN) was one of the most enriched. Abnormal Reelin expression in the brain is implicated in a number of neuropsychiatric disorders.

**Conclusion**: Reelin and Tat are associated with HAND and NeuroAIDS. Could the effects of Tat help identify a target for development of preventative therapies or adjunctive countermeasures for the treatment of the neurodegeneration and behavioural dysfunction associated with HIV infection is yet to be uncovered.

## ISSHID Abstract-329 Analysis of HBx gene among HBeAg seronegative HBV infected individuals

### Reethika Sudharsan^1^, Monika Mani^1^, Gopalsamy^1^, Shanti Vijayaraghavan^2^, Padma Srikanth^1^

#### ^1^Department of Microbiology, Sri Ramachandra Institute of Higher Education and Research, Porur , Chennai, India; ^2^Department of Gastroentrology, Sri Ramachandra Institute of Higher Education and Research, Porur, Chennai, India

**Background:** Hepatitis B virus (HBV) is one of most common etiological factor causing hepatocellular carcinoma (HCC). HBV X gene frequently undergoes mutation play an enigmatic role in HBV related HCC. Amino acid substitutions in X gene from HBeAg negative individuals with different clinical stages was identified.

**Methods:** HBV-infected HBeAg seronegative individuals (n=49) who consented were recruited in the study. DNA was extracted from plasma, amplified, and sequenced using primers targeting HBV X gene (540bp).

**Results:** Among the study participants, 47% were asymptomatic, 26.5% had chronic liver disease and 8% had HCC. Genotypes detected among the participants include D2, D3, A1, C2. D2 was predominant. The frequency of aminoacid substitution in the proapoptotic domain was higher including I127V(34%), K130M(34%), V131I(40%). The frequency of double mutation (K130M+V131I) and triple mutation (I127V+ K130M+V131I) were high (32% and 36%) in HBeAg negative participants as our laboratory already documented these mutations in HBeAg positive participants. Double mutations were detected in 75%, 36%, 25% among HCC, CLD and chronic infection respectively. Among these triple mutations was seen in 50% of individuals with HCC, 36% with CLD and 28% with asymptomatic chronic infection.

**Conclusion:** Identifying amino acid substitutions in X gene will be useful in predicting the risk of developing HCC. We suggest screening for HBx aminoacid substitutions especially in HBe Ag negative participants and inactive carriers to enable early treatment and prevent disease progression.

## ISSHID Abstract-332 Diagnostic importance of detection of Epstein Barr Virus (EBV) in different samples by Real-time quantitative PCR - A Case report

### Nanditha Srinivasan, Ramya Barani, Manickavasagam, Shankar, Padma Srikanth

#### Sri Ramachandra Institute of Higher Education and Research, Porur, Chennai, Tamil Nadu, India

**Background:** Epstein Barr virus (EBV), a lymphotropic virus causing diseases ranging from infectious mononucleosis to carcinoma and CNS infection. Detection of EBV is based on the presence of virus in cells or organs. This study represents the detection of EBV by real-time quantitative PCR in two patients.

Case report: CASE 1- A 64-year-old male, known case of hypopharynx cancer - T2N1M0 on concurrent radiotherapy(RT). After one-week, complaints of nausea,vomiting, fever and decreased appetite. Patient was clinically icteric,diagnosed with hepatitis C virus. Elevated liver enzymes and bilirubin level. Real time qPCR for EBV-BNTp143 gene was performed in blood which was negative; positive in saliva (13055912copies/ml) & started on antiviral therapy. Satisfactory response of hypopharyngeal growth was observed.

CASE 2- A 51-year-old male had complaints of loss of consciousness (5 -6 hours) and weakness of four limbs (one day); two episodes of seizures with tonic clonic movements of both upper limbs. Posterior disc bulge, indentation of mild thecal sac in MRI. CSF analysis: WBC–46 cells/cumm, RBC-28 cells/cumm, Polymorphs (75%)and Lymphocytes (25%). No growth in CSF culture. PCR for herpes viruses (HSV, VZV & CMV) was negative. Real-time q PCR for EBV was negative in CSF; positive in saliva (34,733 copies/ ml). Patient improved symptomatically. Consent from all patients were obtained for the publication of these findings to the journal

**Conclusion:** During active replication, virus can circulate in salivary epithelial cells and blood. We found that virus detected in saliva but not detected in blood or CSF. This study emphasizes the importance of screening of EBV in different samples when they are clinically suspicious for viral infection.

## ISSHID Abstract-333 A retrospective study on bacteria isolated from conventional blood culture

### Anusuya C, Tessa Antony, Uma Sekar

#### Department of Microbiology, Sri Ramachandra Institute of Higher Education and Research, Porur, Chennai, India

**Background:** Blood culture is a critical life-saving investigation in patients with bloodstream infection. The aim of blood culture is to isolate and identify the pathogen and provide the antibiotic susceptibility pattern. Contamination from skin flora can lead to false positive results and unnecessary treatment. This study evaluates the rate of contamination of blood culture, pathogens isolated and their susceptibility pattern.

**Methods:** A retrospective study of 469 blood samples that was received in the Microbiology laboratory of a tertiary care center for conventional blood culture was done over a period of one month. The growth of a known skin flora from a solitary specimen was taken as skin contaminant.

**Results:** There were 186(39.65%) samples from pediatric age group (<15 years) and 283 (60.3%) samples from adult patients. Bacteria was isolated from 39 samples (21 contaminants &18 pathogens)

The contamination rate was 4.47 % (n=21). Among the contaminants Micrococci accounted for 52.38% (n=11); diphtheroids 23.80% (n=5) and coagulase negative Staphylococci 23.08% (n=5) respectively.

Among the pathogens gram-negative bacteria accounted for 66.66 % (n = 12) and gram-positives 33.33% (n = 6) respectively. *Staphylococcus aureus* was the most frequently isolated organism 27.77% (n = 5) followed by *Salmonella* species 33.33% ( n=4), *Escherichia coli* and *Acinetobacter* species 25% (n=3) respectively

**Conclusion:** This study showed that blood culture contamination rate was 4.47%. There were no multidrug resistant bacteria isolated as the samples were received mainly from the wards and the outpatient department

## ISSHID Abstract-335 Opportunistic infections in people living with HIV/AIDS on Antiretroviral therapy at ART plus Centre, Udupi district

### Jessica D’Souza^1^, Shobha K. L.^1^, Ramachandra Kamath^2^

#### ^1^Department of Microbiology, Melaka Manipal Medical College, Manipal Academy of Higher Education, Manipal, India; ^2^Department of Community Medicine, Kodagu Institute of Medical Sciences, Kodagu, India

**Background:** Opportunistic infections in people living with HIV/AIDS (PLHA) is an added burden to the individual which requires timely diagnosis and appropriate treatment. Our objective was to assess the types of opportunistic infections among PLHA on antiretroviral Therapy (ART)

**Methods:** The study population included PLHA adults (n=450) receiving ART services at ART center in Udupi District Hospital, India. Ethical clearance was obtained (IEC257/2015 KMC Hospital, Manipal). The study was conducted using quality of life (WHO-QOL) questionnaires, and data from patient files

**Results:** Among the patients interviewed 60% were women of poor socioeconomic background, from rural areas (98.2%) and low level of education (no schooling, 17. 1%) with mean age of 43.6 years (9.2 SD). CD4 count was below 200 cells/μL in 5.6%. Among the patients 119(26.1%) had opportunistic infections of which pulmonary tuberculosis (15.1%) was the most common, followed by herpes zoster (4. 4%), oral candidiasis (1.8%) and extra pulmonary tuberculosis (1.5% cases). Quality of life was low, the spirituality/personal belief domain was least with mean score of 6.9(4.1SD)

**Conclusion:** Tuberculosis as the most common opportunistic infection among the HIV/AIDS patients which may be related to CD4 counts. These patients also suffer from low quality of life due to opportunistic infections.

## ISSHID Abstract-336 Role of real time PCR in screening for HCV among patients undergoing dialysis and relevance of genotype identification

### Chidurala Rahul, Monika Mani,Vigna Seshan RV, Padma Srikanth

#### Department of Microbiology, Sri Ramachandra University, Chennai, India

**Background:** In India, about 1in 10 have chronic kidney disease (CKD). Every year 1, 75,000 patients add upto end stage renal disease (ESRD). Treatment options are haemodialysis and renal transplant. Hepatitis C Virus (HCV) screening in patients undergoing haemodialysis is done by ELISA and RT-PCR. However discordance between both the techniques creates clinical dilemma. In this study we detected HCV by real time PCR and the circulating genotypes by DNA sequencing.

**Methods:** Blood samples were collected. RNA was extracted from the plasma and quantified using HCV Real time PCR Kit (Qiagen, Germany). The results were compared with HCV Ab-ELISA (CMIA). NS5B gene was amplified using one step RT-PCR kit (Qiagen, Germany) from the extracted RNA. DNA sequencing was performed and the bidirectional sequences were analyzed using HCV geno2pheno database

**Results:** In all, 171samples were screened for HCV of which 58 samples (34%) were positive by RT-PCR. Mean age of the study participants was 52.3+15 years. Among 58 individuals, 56 (96.5%) were positive by PCR and ELISA. Two (3.5%) HCV ELISA reactive samples were negative by PCR and three (5.2%) HCV antibody ELISA non-reactive samples were found to be positive by PCR. Genotyping was performed for 10 participants and genotype 3 was predominant (70%) followed by genotype 1(30%).

**Conclusion:** The study justifies the use of real time PCR for screening HCV, since false positives are possible by ELISA. Genotype 3 was predominant (70%) among the dialysis patients which is important for therapy.

## ISSHID Abstract-338 Serological study of Dengue fever and its correlation with hematological parameters in a tertiary care center

### Subalakshmi.S, Kalyani.M

#### Department of Microbiology, Saveetha Medical College and Hospital, Thandalam, Kanchipuram Dist, Tamilnad, India

**Background:** Dengue fever/Hemorrhagic fever/Shock syndrome are the dangerous forms of febrile illness that requires high vigilance from clinicians. Dengue viral serology is the useful method in the active surveillance of dengue fever.The study aims at correlating the Serological and hematological parameters of the patients with suspected dengue fever.

**Methods:** This cross sectional study was conducted at Saveetha Medical College from August 2018-July 2019. Serum samples received in Microbiology laboratory from 1127 patients suspected to have dengue clinically, were tested for dengue NS1 antigen, IgM and IgG antibody using Evolis Twin Plus Automated ELISA. Clinical history of the patients with the hematological parameters were analysed with serological test results.

**Results:** Among 1127 serum samples, 409 were found to be positive for one or more serological dengue markers. 69(9.61%) were positive for NS1 antigen, 221(30.7%) for IgM and 60(8.35%) for IgG respectively. 22 were found to have secondary dengue infection among which 2(9.09%) were positive for NS1 and IgG, 12(54.54%) for IgG and IgM and 8(36.36%) for all the three parameters. Regarding the haematological parameters, 72% were found to have thrombocytopenia within 5th day of fever, 62% had leucopenia, 31.8% patients had decreased hemoglobin and hematocrit was increased in one case. Aspartate transaminase levels were increased in 53.6% patients on biochemical analysis

**Conclusion:** Though there is no specific medical treatment for Dengue, the serological and haematological parameters help in monitoring clinical recovery. This study results can be used as a screening tool for early clinical response.

## ISSHID Abstract-340 Awareness about HIV pulse and daily drug regime tuberculosis among health care providers

### Sharvani R, Divya Bharathi M, Hemavathi

#### Microbiology department, Sapthagiri Medical College & Research Centre, Bangalore, India

**Background:** Always, awareness among the health care providers (HCP) is the key for implementation and success of any program. In India HIV&TB are two important burdens which we are facing. This study assesses the awareness among the HCP about HIV PULSE mobile application and daily drug regime (DDR) for TB as they are the new updates in the field of HIV&TB.

Materials and **Methods:** 90 health care providers in and around medical college hospital were interviewed using a questionnaire.

**Results:** Out of 90 HCP, some were treating & some were referring TB /HIV/both cases. In total 58.9%(53) were aware about only DDR for TB&only4.4%(4) aware about both. Among the 45 HCP who were treating TB,82%(37)were aware and following DDR,18%(8) unaware and prescribing thrice weekly regime. Among 22 HCP who were treating HIV, 95.5%(21) were unaware & only 4.5%(1) aware about HIV PULSE, but did not notify using this mobile application. Sources of awareness among 57 HCP were- own interest visiting online updates33.3%(19),attending CME ,training program28.1% (16)each,casual discussion10.5%(6).The mode by which 90 HCP prefer to get updated was via E-mail 35.6%(32) ,mobile23.3%(21)& least preferred CME8.9%(8).

**Conclusion:** In our study though awareness about TB updates is better than HIV, but to achieve the NSP targets “eliminating TB by 2025” even small amount of unawareness also needs to be addressed.HIV PULSE is a friendly,technology based application, awareness is important for its success. Hence there is a need for coordination between HCP and policy makers. Once program implemented, monitoring and evaluation is equally important.

## ISSHID Abstract-345 Crypted in the Immunocompromised Gut

### S Josephine, M.Susruthan, G.Barathi, G.A.Vasugi, S.Rajendiran

#### Department of Pathology, Sri Ramachandra Institute of Higher Education and Research, Chennai, Tamil Nadu, India

**Background:** Immunodeficiency is complicated by a wide spectrum of opportunistic infections, in which enteric protozoan parasites play a significant role. A suitable example to indicate the predilection for the immunocompromised and the correlation of morbidity with defects in immune response is Cryptosporidiosis. Commonly caused by *C.parvum*, it presents with diarrhoea, which may be resolved easily in immunocompetent people, but manifests with increased severity and chronicity in the immunocompromised.

Case reports: CASE 1:A 50 year old male who was diagnosed with HIV two years back, presented with complaints of chronic diarrhoea and severe weight loss, not responding to treatment. Duodenal biopsy revealed organisms resembling cryptosporidium. Also, imaging studies revealed cystic changes and calcification in the posterior segment of both lungs, indicating dissemination of infection.

CASE 2:An 8 yr old male who presented with recurrent fever, loose stools and abdominal pain was diagnosed with common variable immunodeficiency(CVID). Although stool tests, cultures and endoscopy were negative, colonic biopsy revealed a diagnosis of cryptosporidiosis.

Consent has been duly obtained from both the patient described in Case 1 and parents of 8-year-old boy in Case 2 for publication of their data.

**Conclusion:** GIT being the largest lymphoid organ is severely affected in immunodeficiency. In HIV, cryptosporidiosis is the most common cause of diarrhoea caused by parasites, the incidence and severity of which is dependent on the CD4 cell count. Persistent disease without treatment results in dissemination of the infection, sepsis and fatal outcome. In children with CVID, cryptosporidium associated diarrhoea is characterised by malabsorption and failure to thrive due to its chronicity and severity. Thus, diarrhoea in the immunocompromised should always raise a suspicion for cryptosporidiosis.

## ISSHID Abstract-348 Prevalence of Extended Spectrum Betalactamase (ESBL) and Metallobetalactamase (MBL) in *A. baumannii* isolates obtained from various clinical samples in a tertiary care hospital

### G. Kavitha, M. Kavitha, S. Rajesh, T. Sundararajan, R. Vidhyarani, S. Deepa, N. Subathra, D. Neelaveni

#### Deparment of Microbiology, GMKMCH, Salem, Tamilnadu, India

**Background:***Acinetobacter baunamii* is one of the ESKAPE pathogen, a group of clinically important predominantly health care associated organism that has the potential for substantial antimicrobial resistance. This study was conducted with an objective to find the prevalence of ESBL and MBL in *A. baumannii* isolates obtained from various clinical samples.

**Methods:** This is a cross sectional descriptive study conducted in the Department of Microbiology in GMKMCH Salem between June – August 2019.A total of 145 Acinetobacter spp were isolated from clinical specimens including blood - 59, pus-55 and urine -31. All the isolates were screened for ESBL production by double disc diffusion method and MBL production as per the CLSI guidelines 2019.

**Results:** Out 145 isolates of *Acinetobacter* spp among that in blood 32 (54%) , pus 38(69%) , urine 18(58%) were found to be ESBL producers. There were no MBL producers . Majority of the ESBL producers (59%) were isolated from blood stream infection in neonates admitted in NICU. This was followed by isolates (55%) from pus samples of patients admitted in surgery and orthopedics depts

**Conclusion:** Active surveillance of antibiotic resistance among *Acinetobacter* isolates provides guidance to the clinician to choose antibiotic for the empirical treatment depending on local antibiotic susceptibility pattern and to formulate antibiotic policy to maintain antibiotic stewardship. Early detection, compliance towards infection control practices on hand hygiene, aseptic care of IV line, contact isolation and recommendation of environment cleaning are the best defenses against these organisms.

## ISSHID Abstract-350 Nosocomial urinary tract infection due to *Trichosporon asahii*

### Yamini Anandan, Swati kumari, T. Premamalini, Anupma Jyoti Kindo

#### Department of Microbiology, Sri Ramachandra Institute of Higher Education and Research, Chennai, India

**Background:** Catheter associated urinary tract infections caused by fungi, have increased in recent times causing morbidity and mortality among hospitalized patients.There is difficulties on species identification as well as standardized sensitivity testing invitro.This causes limited information on epidemiology,diagnosis,treatment of trichosporon infections.We report two cases of UTI caused by *T.asahii* in severely ill patients in tertiary care set up.

**Methods:** Both the samples were collected by clamping the catheter. Gram stain revealed moderate pus cells, yeast cells, arthrospores. Urine sample inoculated on CLED agar revealed significant growth of>10^5dry wrinkled creamy white colonies after overnight incubation.Further subcultures were done on SDA incubated at 22°C and 37°C,corn meal agar,congo red agar,urea hydrolysis,sugar assimilation was done to identity the morphology and cultural characteristics. AFST was performed by both disk diffusion method using MHA with 2% glucose, methylene blue and MIC. For accurate identification of these isolates,we employed conventional PCR and sequencing.

**Results:** LPCB revealed septate hyaline hyphae with blastoconidia, arthroconidia and budding yeast cells.Subcultures on SDA revealed curdy white cerebriform growth with radial fissures which turned cottony on subsequent incubation consistent with that of Trichosporon species. AFST showed resistance only to fluconazole. PCR was performed with primer pair ITS1/ITS4 and T.asahii-specific TASF and TASR primers, yielded specific amplification of a DNA fragment at 430bp with genomic DNA from the reference cultures.

**Conclusion:** Natural barrier of skin and mucosa gets compromised by indwelling catheter leading to increased opportunistic infection including UTI.The use of longterm antibiotics in severely ill patients favour fungal development, particularly yeast.So high index of clinical and microbiological suspicion needed to control of Trichosporon infection

## ISSHID Abstract-355 The Unusual Invasion

### S. Anand , M.Rajkumar, Sathyamoorthy, Bhargav

#### Internal Medicine, Sri Ramachandra Institute of Higher Education and Research, porur , Chennai

**Background:** Abdominal Tuberculosis include involvement of the gastrointestinal tract, peritoneum, lymph nodes and/or solid organs.Abdominal TB comprises 5% of all TB cases worldwide.Conglomerate Abdominal lymphadenopathy is common.The abdominal lymph nodes encasing the great vessels of the abdomen is a rare finding and no such cases has been reported till date

Case Report: 24 year old male with 10 days h/o intermittent low grade fever with no other localising signs or symptoms.History of 10 kg weight loss in last 2 months.Clinical examination was normal except for right sided inguinal lymphadenopathy. Haemogram, electrolytes, RFT, LFT, CXR, ECG normal. Tropical fever work up negative, cultures sterile, viral markers non reactive. USG abdomen showed splenomegaly.CECT abdomen done showed generalised abdominal lymphadenopathy involving bilateral para-aortic(R-4.5 X2.7 cm,L-3.5x1.9cm), retrocaval and iliac regions(R-3.2x1.8cm,L-3.8x2.3 cm) along with bilateral psoas abscess.

The Unusual Presentation Was The Ivc Being Completely Encased And Severely Compressed By Nodes From L2 To Iliac Confluence

Aorta was also completely encased by nodes with no significant narrowing.

Biopsy from lymph node was suggestive of tuberculosis (AFB and gene Xpert was positive) , following which AntiTb Treatment was started.. Consent was obtained from the patient for the publication of his data.

**Conclusion:** In this patient there was abdominal tb lymphadenopathy which was significant enough to compress the large vessels like aorta and also ivc. Abdominal tb lymphadenopathy causing vessel encasement is a rare finding and management guidelines if it causes severe obstruction to blood flow is not available,similar case scenarios are not reported yet,hence i would like to present this case.

## ISSHID Abstract-356 Bacterial Profile of Urinary tract infection and Anti microbial Susceptibility pattern among adult patients in a tertiary care hospital

### Subhashini Ramadurai, Swati Kumari

#### Sri Ramachandra Institute of Higher Education and Research, Chennai, India

**Background:** Urinary tract infection is one of the most common communities acquired and hospital acquired infection in developing countries like India. UTI can occur in any age group and both sexes, but more common in women due to short urethra proximity to anus.

**Methods:** The study was a retrospective analysis of 233 urine culture reports belonging to the adult age group, in SRMC, Chennai in August 2019. The urine samples were cultured, the results were recorded from Central Lab.

**Results:** From 233 urine samples, 43 (18.4%) were positive for growth of organism in culture.

Age distribution among Females:

**Table:** 

(Years)	% Cases
21 - 40	40.9
41 - 60	22.7
61 - 80	36.4
> 80	-

Age distribution among Males:

**Table:** 

Age (Years)	% Cases
21 - 40	14.3
41 - 60	33.3
61 - 80	38.1
> 80	14.3

Male:Female was 0.9 : 1. Among female patients, commonly affected age group was the reproductive age group : 21-40 years; 61-80 years group in males. Most commonly isolated organisms were *E.coli* followed by *Klebsiella pneumoniae*, Pseudomonas spp, Enterococcus spp, etc. Antibiogram showed most of the isolates tested were resistant to commonly prescribed antibiotics. Carbapenems, Aminoglycosides and Nitrofurantoin were found to be effective antibiotics.

**Conclusion:** Study showed higher prevalence of UTI in reproductive age group female and the potential pathogens were Gram negative bacilli( resistant to most of the commonly used antibiotics used to treating them). Routine screening of women in reproductive age group attending the hospital should be done.

## ISSHID Abstract-358 Prevalance of extented spectrum beta lactamase (ESBL) producing *Escherichia coli* among various clinical samples in a tertiary care hospital

### V.Ramya, M.Kavitha, S.Rajesh, T.Sundararajan, R.Vidhyarani, S.Deepa, N.Subathra, D.Neelaveni, M.Gomathi

#### Department of Microbiology, GMKMCH, Salem, Tamilnadu, India

**Background:** ESBL producing *E. coli* are emerging pathogens in worldwide now a days. These isolates are resistant to Penicillins, I, II & III generation Cephalosporins and to Aztreonam. Increasing resistant organisms tends to make empirical treatment more difficult. Infections complicated by ESBL producing organisms will leads to increased morbidity and prolonged hospitalization.

**Methods:** This is a descriptive study conducted in GMKMCH Salem from June to August 2019. About 1200 samples including Urine, Pus & Blood were collected from hospitalised patients. Among total isolates ESBL producing *E. coli* were detected and confirmed by phenotypic method (Combined disk method – Cefotaxime 30μg and Cefotaxime-clavulanic acid) as per CLSI guidelines.

**Results:** 261 (21.8%) *E coli* isolates were isolated among total samples and 55% isolates from Urine, 39% from Pus & 6% from Blood. Out of total isolates 177 (67%) were ESBL producers and 65%, 74% & 50% of ESBL producers were isolated from Urine, Pus & Blood respectively. The highest range of ESBL producing *E. coli* isolates were isolated from ICU patients (86%).

**Conclusion:** The present study shows high burden of ESBL and increasing drug resistant *E. coli* isolates from a tertiary care hospital. A strong antimicrobial stewardship program, strict infection control practices and rationale therapy to minimize the development of resistant strains. Regular surveillance of ESBL detection and their susceptibility pattern are helpful to prevent treatment failure and thereby the decrease morbidity and mortality.

## ISSHID Abstract-359 Clinical rotavirus infection in neonatal intensive care unit from a tertiary care centre, Chennai, South India

### Sudhabharathi Reju^1^, Sribal Selvarajan^1^, Binu Ninan^2^, Prakash Amboiram^2^, Gunasekaran Palani^3^, Gagandeep Kang^4^, Padma Srikanth^1^

#### ^1^Department of Microbiology, Sri Ramachandra Institute of Higher Education and Research, Porur, Chennai, India; ^2^Department of Neonatology, Sri Ramachandra Institute of Higher Education and Research,Porur, Chennai, India; ^3^Former Director, King Institute of Preventive Medicine and Research, Guindy, Chennai, India; ^4^Director, Translational Health Science and Technology Institute, Faridabad, New Delhi, India

**Background:** Neonatal rotavirus infections have been associated with necrotising enterocolitis (NEC), abdominal distenstion, feeding intolereance, hematochezia and gastroesophageal reflux. However, it also causes asymptomatic infection in neonates. Rotavirus infection in neonates has not been studied extensively. This study is done to detect rotavirus among symptomatic and asymptomatic neonates and identify the commonly circulating strain in Chennai, South India.

**Methods:** Stool samples were collected from both symptomatic and asymptomatic neonates from August 2018 - November 2018, among those who were admitted for more than >48 hours in the neonatal intensive care unit (NICU). Samples were screened for rotavirus antigen using Premier Rotaclone. The samples which tested positive were subjected to conventional nested RT-PCR targeting VP7 and VP4 to identify the genotype.

**Results:** Rotavirus antigen was detected in 24.4% (n=21) of 86 neonates. Rotavirus infection was more common in first week of life (28.1%) followed by second week (18.5%). Rotavirus infection was observed in 30.5% of late preterm and term neonates and it was 11.1% among moderate and very preterm neonates. Neonates with gastrointestinal symptoms (diarrhoea, vomiting, NEC and feed intolerance) had 42.9% rotavirus infection. 33.3% and 28.5% of rotavirus positive neonates had a birth weight of 2-3kg and >3kg respectively.

**Conclusion:** Late preterm and term neonates show high risk for rotavirus infection. Neonates exhibiting gastrointestinal symptoms are at higher risk of rotavirus infection. Neonates are vulnerable and very sensitive host for rotavirus even in the first week of life. A single strain, G10 P [11] was found to circulate in NICU.

## ISSHID Abstract-363 Comprehensive detection of *Mycobacterium tuberculosis* by different laboratory methods

### Shiny Vincent, Sribal Selvarajan, Krithika Gopalakrishnan, Ramya Barani, Chockalingam Chandrasekar, Rajagopalan, Padma Srikanth

#### Sri Ramachandra Institute of Higher Education and Research, Porur, Chennai, India

**Background:** India accounts for 1/4th of the world’s TB cases. ‘THE END TB strategy’ vision is 90% reduction in TB incidence and 95% reduction in TB deaths by 2035. Molecular methods are underutilised in the detection of MTB. This study is done to detect Mycobacterium tuberculosis(MTB) by different laboratory methods to initiate treatment early.

**Methods:** Pulmonary samples (n=45) were collected from participants visiting DOTS centre and extra-pulmonary samples (n=5) from in-patients with a high index of suspicion for TB during 2016-2017. Samples were subjected to Kinyoun’s staining, LED-Fluorescent microscopy, Lowenstein- Jensen culture and real-time PCR targeting IS6110(HELINI, India) for detecting MTB and multiplex real-time PCR(SEEGENE, Korea) for detecting MTB and MDR-TB.

**Results:** Among n=45 pulmonary samples, MTB was detected in 97.8% by Kinyoun’s staining, 100% by fluorescent microscopy and 86.7% by culture. Real-time PCR detected MTB in 82.2% whereas multiplex PCR detected MTB in 100% and MDR-TB in 10% of samples tested. In n=5 extrapulmonary samples, MTB was detected in 60% by Kinyoun’s staining, 80% by fluorescent microscopy, 60% by real-time PCR. Multiplex PCR detected MTB in 80% and MDR-TB in 20%. Fluorescent microscopy and multiplex PCR had a sensitivity, accuracy of 96% and positive predictive value of 100%.

**Conclusion:** In this study. fluorescent staining and multiplex PCR is superior to AFB staining, culture and real-time PCR. We suggest the use of molecular tests for early diagnosis of MTB and MDR-TB. We need multiple laboratory tests to rule in early diagnosis of TB in order to prevent and control TB.

## ISSHID Abstract-365 Therapeutic monitoring of Antiretroviral drugs in patients living with Human Immunodeficiency Virus Infection co-infected with Tuberculosis

### Sabitha Panchagiri^1^, Veeresham Ciddi^2^, Chandrasekhar Valupadas^3^

#### ^1^Department of Pharmaceutical Sciences, University college of Pharmaceutical Sciences, Kakatiya University, Warangal, India; ^2^College of Pharmacy, Sri Ramachandra Institute of Higher Education and Research, Chennai, India; ^3^Department of General Medicine, ART Centre, MGM Hospital/Kakatiya Medical College, Warangal, India

**Background:** Increase in access to anti retroviral therapy (ART) therapy with amplified new infection yearly; demand the role of drug monitoring in HIV management especially in patients co-infected with Tuberculosis (PLHIV+TB).

**Methods:** A total of thirty one (n=31) patient’s dried blood samples for ARV drugs level were analyzed by liquid chromatographic method from PLHIV+TB on TLE (Tenofovir disoproxil fumarate+Lamivudine +Efavirenz) treatment for greater than three months with recorded good medication adherence (i.e. >98%) attending ART centre, Mahatma Gandhi Memorial Hospital (MGMH), Warangal, Telangana, during April 2018 to October 2018. Institutional ethics committee reviewed the study protocol and approved (KIEC/MGM/KMC/NCT/2016/P02).

**Results:** Of the total PLHIV+TB patients 64.52% were males and 35.48% were females. Duration (months) of the HIV infection and TB in these patients were 21.77±27.60 and 5.03±3.17 respectively. Among all, 64.52% (n=20) and 35.48% (n=11) were noticed with pulmonary and extra pulmonary TB respectively. Patients of this group showed 22.58% and 52% with EFV plasma levels below and above therapeutic levels respectively suggesting the dose adjustments to higher and lower doses respectively. It could only be possible with the availability of fixed dose combinations antiretroviral drug regimens with different doses. For the first time, present study also assessed the effect of number of pills for DOTS therapy on therapeutic levels of EFV and found no significant effect on EFV levels.

**Conclusion:** Present study results observed that the patients required either of increase or decrease in Efavirez dose to avoid treatment failure or toxicity respectively in future.

## ISSHID Abstract-367 Multi response genetic optimization for the estimation of decreased median drug resistance to NNRTIs in HIV

### Aishwarya Bai^1^, Balaji Rakshitha^1^, Lily Mercy^2^, Wilson Aruni^1^, Arun Pandiyan^3^, Piyush Bhanu^4^ , Daniel Alex Anand^1^

#### ^1^Department of Bioinformatics and The Centre for Molecular Data Science and Systems Biology, Sathyabama Institute of Science and Technology, Chennai, India; ^2^Department of Mechatronics, Sathyabama Institute of Science and Technology, Chennai, India; ^3^ITC Life Sciences & Technology Centre, Bangalore; ^4^XOME Life Sciences, Bangalore

**Background:** The prevalence of transmitted drug resistance (TDR) in antiretroviral therapy (ART)-naive individuals remains stable in most developed countries despite a decrease in the prevalence of acquired drug resistance. The susceptibility to all currently available NNRTIs was highly affected by transmitted NNRTI resistance mutations

**Methods:** 361 patterns of drug resistance mutations of NNRTI Susceptibility were obtained from the Stanford Drug Resistance Database. Drug – mutation combinations that contained all the median drug resistance for NVP, EFV, ETR, RPV and DOR were analysed. Regression analysis was done to measure the correlation and statistical significance.The data fit the quadratic regression curve with a minimal error value. Quadratic equations for each median drug resistance in terms of the mutated amino acid residue sites were generated and ANOVA was performed. In all the five equations R – Sq value were above 85%. Multiple combinations of mutations and their interaction were feasible.The objective function was set to minimize and multi – objective genetic algorithm solver was used. Solver was run to evaluate the fitness function to obtain a pool of optimized dataset called the pareto – optimal front which contains the function values for all the non – inferior solutions.

**Results:** Seventy optimal mutation combinations were obtained. Six out of nine positions were optimal. The mutations that contributed to high median fold drug resistance were in the positions – 100, 230 and 236.

**Conclusion:** Thus in this study, the combination of NNRTI mutations leading to median drug resistance were been identified.

## ISSHID Abstract-368 Investigation and Modeling of Nelfinavir specific protease inhibitor mutations and their patterns of Drug Resistance using Fuzzy and Neural network algorithms

Riya Joshi^1^, Gadiraju Divya Sai Ananya^1^, Riya Saju^1^, Rakesh. I^1^, Lily Mercy^2^, Arun Pandiyan^3^, Piyush Bhanu^4^, Wilson Aruni^1^, Daniel Alex Anand^1^

^1^Department of Bioinformatics and The Centre for Molecular Data Science and Systems Biology, Sathyabama Institute of Science and Technology, Chennai – 600119

^2^Department of Mechatronics, Sathyabama Institute of Science and Technology, Chennai – 600119

^3^ITC Life Sciences and Technology Centre, Bangalore.

^4^XOME Life Sciences, Bangalore

**Background:** Nelfinavir (NFV) was once widely prescribed HIV-1 specific protease inhibitor (PI).

**Methods:** 127 of 1453 patterns of major drug resistance mutations from HIV-1 Group M virus were studied. ML was implemented on the 127 datasets using ANFIS. 12 amino acid positions were considered for the mutations on HIV – 1 protease. Sugeno fuzzy inference system and neuro-adaptive learning techniques were employed. Hybrid optimization method was adopted during the cluster search which combines the forward and the back propagation methods of neural networks.

**Results:** The model did not follow linear or non – linear regression patterns. There were no significant outliers in the data and we observed interactions between the various NFV specific mutations in the same sequence contributing to the resistance. Mutual exclusivity in amino acid residue sites were found like when the 47th position was mutated with V, no mutation was observed at positions 30,48,50,76 and 88. When the 47th position was mutated with V, the 46th position is not mutated with L. Several combinations of mutations in the protease gene have been reported to demonstrate low protease activity and reduced viral fitness but the most incompatible pairs or combinations are yet to be discovered and the exact mechanism of incompatibility are also not yet understood.

**Conclusion:** The double mutations or combinations that completely eliminate infectivity and replication capacity need to be ascertained. Since the development of resistance to ARV are the rule over time, sequential use of different options in an appropriate manner is the vital.

## ISSHID Abstract-369 Elevation of cytokines leads to Multiorgan injury in severe dengue: A case report

### Nitya Rajaraman, Gopalsamy Sarangan, Monika Mani, Padma Srikanth

#### Depaartment of Microbiology, Sri Ramachandra Institute of Technology and Research, Porur, Chennai, India

**Background:** Dengue is reported from more than 150 countries and the mortality due to dengue in the age group 18-40 years is high. Cytokines play a significant role in viral immunopathogenesis. Cytokines destroys the vascular endothelial cells, increase capillary permeability and worsen the disease.

**Case Report:** A 32 year old male came with the complaints of fever with severe headache for 5 days, active gum bleeding and 3 episodes of melena during admission. He had a hospital stay for 8 days. On investigation patient had severe thrombocytopenia. Patient was found to be positive for Dengue NS1 antigen by ELISA and serotype was confirmed as DENV-2 by real-time PCR. This patient had increased expression of IL-10 (1007.31pg/ml) and IL-8 (1121.35pg/ml) when tested by quantitative ELISA. Non Th1/Th2 (IL-8) response also promotes Th2 responses (IL-10). Expression of non Th1/Th2 cytokines (IL-8) was found to be more in viremic phase of dengue since the patient had high dengue viral titre. Patient was managed by supportive therapy and blood transfusion. Patient had severe thrombocytopenia and he experienced altered sensorium and irritability. Patient succumb to death on 8th day due to MODS (multiple organ dysfunction syndrome) and septic shock. Consent has been given by the concerned patient for publication

**Conclusion:** Interplay of different inflammatory cytokines during a dengue (DEN) virus infection plays a role in either protection against disease or increase disease severity. Alteration in the levels of cytokines IL-10, IL-8 determines the disease spectrum either mild or severe such as dengue haemorrhagic fever as described in the above case which had multiorgan failure

## ISSHID Abstract-370 Does an Individual's Hygiene Affect Their Health?

### Varshini Veeramani, Krithika Balakrishnan, Sudhabharathi Reju, Padma Srikanth

#### Department of Microbiology, Sri Ramachandra Institute of Higher Education and Research, Porur, Chennai, India

**Background:** A plethora of infections- bacterial, fungal, or parasitical- are transmitted in unhygienic environments where the residents lack in maintaining sanitary conditions. Poor hygiene has led to many health problems all over developing countries such as India. It mainly affects the young children, which may deteriorate their health status. Though it affects the health badly, it is easily preventable.

**Methods:** The research design adopted for the study was a survey and the research approach adopted for the study was a non-experimental approach. 214 students from preschool to grade 5. The students were randomly selected from a school in the district of Mangadu. Data collected from structured interview questionnaire about hygiene and food habits.

**Results:** Although about 85% of the students were aware of the knowledge about proper sanitation, a majority of them lacked the application of it in their practical lives. Around 75-80% of the students, especially in the lower grade (preschool and 1st grade) did not follow it due to their normal behaviors and forgetfulness. This resulted in more frequent causes of illness and the occurrence of parasitical or bacterial infections.

**Conclusion:** The present study assessed the knowledge and practice of hygiene standards among school aged children. Since improper hygiene is the most common mode of transmission of diseases, students that lacked in cleanliness became sick more frequently and hence, took more “sick leaves” or absences. There is a positive correlation between knowledge and practice of personal hygiene among school aged children.

## ISSHID Abstract-371 A circulating pattern of rotavirus genotypes among children <5 years from a tertiary care center, south India

### Sudhabharathi Reju^1^, Sribal Selvarajan^1^, Ramachandran Padmanaban ^2^, Gunasekaran Palani^3^, Gagandeep Kang^4^, Padma Srikanth^1^

#### ^1^Department of Microbiology, Sri Ramachandra Institute of Higher Education and Research, Porur, Chennai, India; ^2^Department of Pediatrics, Sri Ramachandra Institute of Higher Education and Research, Porur, Chennai, India; ^3^Former Director, King Institute of Preventive Medicine and Research, Guindy, Chennai, India; ^4^Director, Translational Health Science and Technology Institute, Faridabad, New Delhi, India

**Background:** Rotavirus is the leading cause of acute gastroenteritis in children <5years of age. Globally rotavirus accounts to78000 deaths in children < 5 years. This study was done to determine the incidence of acute gastroenteritis caused by rotavirus among children and to identify the common circulating genotypes.

**Methods:** Stool samples were collected from the pediatric ward from Sep 2018 to Nov 2018. A total of 76 samples in children < 5 years were collected. Rotavirus antigen was detected by commercial enzyme immunoassay Premier Rotaclone (Meridian Bioscience INC., Cincinnati, USA). The samples which tested positive by ELISA were subjected to conventional VP7 & VP4 RT-PCR to identify the respective genotypes. The severity of disease was assessed using the Vesikari clinical severity scale. Socio-economic status was assessed using a modified Kuppuswamy scale.

**Results:** Rotavirus was observed in 28.9% of children admitted in our center. Rotavirus infection was higher among male children. The mean Vesikari score among rotavirus infected children was significantly higher (11.3+2.8) when compared to rotavirus un-infected children (9.1+2.3) [P=0.0008]. Rotavirus infected children were more common in the lower middle class (32.7%). The circulating genotypes identified in children < 5 years were G3P[8], G1P[8], G2P[4].

**Conclusion:** Rotavirus was detected in 28.9% of children < 5 years. G3P [8] has replaced G1 P[8] as the predominant genotype in our centre. Rotavirus diarrhoea was more severe than other causes of diarrhoea. There is a change in predominant genotype after the introduction of rotavirus vaccine and thus continuous surveillance of rotavirus genotypes is necessary.

## ISSHID Abstract-374 Multiplex PCR assay for detection of *Escherichia coli* O157:H7 and screening for non-pathogenic *E. coli*

### Rajnish Kumar^1^, Sujata Adhana^1^, Daman Saluja^2^, Balaram Pani^1^, Uma Chaudhry^1^

#### ^1^Bhaskaracharya College of Applied Sciences, University of Delhi, New Delhi, India; ^2^Dr. B. R. Ambedkar Center for Biomedical Research, University of Delhi, Delhi, India

*Escherichia coli* isolates have been long considered to be ideal laboratory workhorses. Strains such as Shiga toxin-producing *E. coli* and enterotoxigenic *E. coli* can cause severe foodborne disease. These are transmitted to humans primarily through consumption of contaminated foods*. E. coli* can act as an indicator for the presence of other pathogenic bacteria as it is always found to be present with other bacteria, and it is detected easily in foods such as fresh produce. Thus, *E. coli* detection in foods is one of the most useful hygienic criteria which can be exploited and extrapolated for detecting other pathogenic organisms as well.

We have developed a multiplex PCR method to differentiate pathogenic E. coli from non-pathogenic counterpart present in fresh produce. PCR using primers against unique gene that encodes beta-D-glucuronidase was used to identify *E.coli* accurately. The objective of the present study was to develop a rapid detection method for *E. coli* in food samples, using a combination of enrichment and PCR that can also differentiate pathogenic *E. coli* from non-pathogenic counterpart. Most of the samples that were analysed exhibited non-pathogenic *E. coli* and some samples mostly green leafy vegetables collected from local market were found to be positive for pathogenic E.coli. The test was standardized using a mixture of positive and negative cultures of *E. coli*. As low as 10ng of DNA sample was sufficient enough to be used for amplification purposes. This study showed that multiplex kit was highly specific and sensitive for detection of pathogenic *E.coli* samples.

## ISSHID Abstract-375 Polybacteremic secondary blood stream infection: A case report

### Anuniti Mathias, Padma Das

#### Department of Microbiology, All India Institute of Medical Sciences, Raipur, Raipur, India

**Background:** Polymicrobial bacteremia, i.e. bacteremia due to atleast two different organisms isolated from the same blood sample, can occur secondary to infections of gastrointestinal tract, respiratory tract, abcess or an occult site. CDC defines a secondary BSI as a BSI thought to be seeded from a site specific infection at another body site.

**Case Report:** A 19 year old female, known case of schizoaffective disorder for 2 years, was brought from outside hospital with high fever and confused mental state, vomiting, inability to talk and loose stools. Relevant specimens were sent and antibiotics started. CSF sent for culture was sterile. FNAC report was suggestive of TB, so anti-tubercular regimen was started. However, the patient continued to have high fever and developed altered sensorium and convulsions. The patient developed respiratory failure, thrombocytopenia, DIC and sepsis. Antibiotics upgraded and anti-fungal prophylaxis added. Urine culture sent on admission day 9 grew *E.faecalis* and *C.tropicalis*. ET aspirate sent on days 9 & 10 grew *A.baumannii*. Blood culture sent on day 10 showed growth of multiple bacteria, so a repeat paired blood specimen was sent on day 12 from which similar growth of *E.faecalis, A.baumannii* and Coagulase negative Staphylococcus was isolated. However, on day 12 she consecutively developed bradycardia and asystole from which she could not be revived. Consent was obtained from the parent of the deceased patient for the publication of patient’s data.

**Conclusion:** Polymicrobial BSIs are associated with higher mortality, hence identifying patients at risk for such infections and timely diagnosis by appropriate laboratory investigations such as paired blood culture and site specific cultures are important.

## ISSHID Abstract-376 Assessment of influenza virus viability on different surface and efficiency of laboratory disinfection procedure against Influenza virus

### Sudalai Eswaran, Gopalsamy Sarangan, Monika Mani, Padma Srikanth

#### Dept. of Microbiology, Sri Ramachandra Institute of Higher Education and Research, Porur, Chennai, India

**Background:** Most common cause of flu is Influenza A/H1N1 which belongs to Orthomyxoviridae. Transmission of influenza virus mainly happens through aerosols, droplets, fomites and contact with contaminated surface. We therefore measured the viability of Influenza A/H1N1 on the surface of two different materials and the efficiency of UV and 70% ethanol in influenza virus inactivation.

**Methods:** Samples with high and low viral load were included for the analysis. Surface viability was tested on stainless steel and wood by incubating the samples for 0, 3,5,10,15,30 min and overnight. Virus inactivation was analyzed by exposing to UV for 30min and 70% ethanol. RNA was extracted from the treated samples using Qiagen RNA extraction kit. Extracted RNA was amplified using one step RT PCR kit (Ag Path One Step RT-PCR kit) and assay mix (Applied Biosystems) with appropriate quality controls.

**Results:** Despite the RNA virus, RNA remains detectable at same level in high viral load sample extracted from the surface of stainless steel and wood after 12hours. However complete loss of RNA was observed in low viral load samples. Virus inactivation was achieved in low viral load following 30 mins UV exposure and 70% ethanol. In high viral load, we noticed a drop of viral RNA level which was evidenced by the difference in ct value from 20 to 27.

**Conclusion:** Current disinfectants are effective against samples with low viral load. However samples with high viral load requires a very effective disinfecting procedure to prevent the transmission of Influenza A/H1N1.

## ISSHID Abstract-378 Transfer of antibiotic-resistant bacteria (ARB) from food to human gut: Current understanding and a way forward

### Sujata Adhana, Rajnish Kumar, Balaram Pani, Uma Chaudhry

#### Bhaskaracharya College of Applied Sciences, University of Delhi, Dwarka, India

Non-pathogenic bacteria isolated from food has always been of low clinical relevance. Such bacteria should be proactively analyzed as these could be useful indicator organisms that serve as donors for antimicrobial resistance genes (ARGs) in the gut. Moreover, it is becoming important to understand that pathogenic/non-pathogenic terminology is strain-specific and also to the susceptibility of individual hosts.

In the present study, we evaluated the presence of microbes which are pathogenic vs non-pathogenic in fresh produce. Further investigation on these microbes genomic context was carried out for the presence and absence of antimicrobial genes. Soil, manure, and compost were also evaluated for the presence of pathogenic/non-pathogenic organisms and the presence of ARGs. It is hypothesized that these resistance genes are transferred from our food to the human gut. Our results revealed the presence of diverse bacterial communities in the fruits and vegetables that were collected from retail outlets of Delhi and NCR region.

The present study isolated bacteria that were found to be resistant to Ampicillin, Gentamicin, Tetracycline, Streptomycin, and Nalidixic acid. Multidrug-resistance strains possessing resistance genes (parC, gyrA, mecA, blaCTX-M, and blaTEM) were also detected. Further research should thus focus on clinically relevant antibiotics, should provide (or reject) evidence of transmission at the clonal level, and should clarify and quantify the involvement of commensals in ARG transfer to pathogens as well as in extraintestinal opportunistic infections after transmission by food. Future studies addressing these gaps would be more than welcome.

## ISSHID Abstract-381 Tuberculosis of the chest wall without Pulmonary Involvement-Case report

### Kartheeswari, Sageera Banoo

#### Department of Microbiology, DSMCH-Dr.MGR Medical University,Perambalur,India

**Background:** Tuberculosis of chest is usually seen in association with primary lung involvement. Here we present a case of Tuberculosis of chest wall, a rare entity without any primary foci in the lung or the ribs.

**Case Report:** A 60yrs old male Farmer, presented with painless swelling in the right anterior chest wall 3 month’s duration, which gradually increased in size. Patient is a known case of epilepsy on treatment. There was no H/O loss of appetite, loss of weight, NoH/O respiratory complaints, No H/O DM. O/E: There was a swelling on right anterior chest wall 10x7 cm in size. Ill defined, firm to hard in consistency, No tenderness. Blood investigation of patient showed Hb-10.8gm%.TC-5700 cells/cu.mm, RBS-92mg/dl, HIV- Non reactive. FNAC of right anterior chest wall swelling showed necrotizing lesions with suppuration. Incision and drainage was done.4-5ml of grayish tinged pus was squeezed out. Pus sent for Gram staining, AFB staining, culture and sensitivity and CBNAAT in Microbiology department.Gram Stain showed few pus cells, occasional RBC’s, No organisms seen. In Fluorescent-staining Acid Fast Bacilli_ positive Grade 1+,Pus cultures both aerobic and anaerobic culture showed no growth after 48hrs of Incubation. CBNAAT TB result showed M.tuberculosis and Rifampicin sensitive. Chest X-Ray PA view of lung-No abnormality detected.So this was a microbiologically confirmed extra pulmonary tuberculosis, Anti-tuberculosis drug was started CAT IATT (4pills /day) patient responds well to treatment and on follow up. Consent was obtained from the patient for the publication of his data.

**Conclusion:** A 6 months ATT regimen may be appropriate for chest wall tuberculosis patients whose lesions have been excised completely.

## ISSHID Abstract-382 Distribution of Candida spp. in hospital acquired blood stream infection and study of their virulence factor

### Neetu Srivastava, Padma Das, Archana B Wankhade, Anudita Bhargava, Ujjwala Gaikwad, Sanjay Negi

#### Department of Microbiology, All India Institute of Medical Sciences, Raipur, India

**Background:** Bacteria have been the traditional etiology of nosocomial blood stream infections(BSI) but an increase in the incidence of candidaemia is being observed over the last two decades in different parts of the world. It may be due to increasing use of broad spectrum antibiotics, central venous catheters (CVC) and indwelling devices.

**Methods:** A retrospective study was done from May 2018 to July 2019. 25 Candida species isolated from hospital acquired infection blood culture were identified to species level by Vitek-2 compact system(BIOMERIEUX). Anti-fungal susceptibility done against AmphotericinB, Caspofungin, Fluconazole, Voriconazole, Micafungin and Flucytosine in VITEK2 compact system.Biofilm formation on the surface of wells of microtiter plates was studied by naked eye observation and spectrophotometric test.Virulence factors like haemolysin, lipase, proteinase and esterase were detected.

**Results:** Out of 25 isolates., 9 *C. parapsilosis*(36%), 6 *C. tropicalis*(24%), 5 *C. glabrata*(20%), 2 *C. krusei*(8%) and single isolates of *C. albicans*(4%), *C. auris*(4%) and *C. pellioloa*(4%) were seen. Fluconazole(16%) was found to be most resistant followed by Caspofungin(12%), Micafungin(12%) and Amphotericin B(8%) . Among the 25 isolates subjected for biofilm production, 7(28%) were positive.

**Conclusion:** Candidaemia is increasing in nosocomial blood stream infections with changing anti-fungal susceptibility. Biofilm is responsible for increasing antifungal resistance and virulence factor is responsible for pathogenesis. The changing epidemiology of candida infection, therefore, highlights the need for monitoring the distribution, susceptibility, virulence factor detection and biofilm production of Candida spp. in order to give proper therapy and optimal outcome.

## ISSHID Abstract-384 In vitro and in vivo effect of phytosterols isolated from Sida Cordifolia L. extracts against HSV-1 infection

### Ashish Wadhwani^1^, Selvam P^2^, Viral Patel^3^, Masahiko Kurokawa^4^, Vijayan Pottekad^1^

#### ^1^Department of Pharm. Biotechnology, JSS Academy of Higher Education & Research, JSS College of Pharmacy, Ooty, Tamil Nadu, India; ^2^Aravindh Herbal Labs (P) Ltd., Rajapalayam, Tamil Nadu, India; ^3^Mitra Biotech, Bangalore, Karnataka, India; ^4^Department of Pharmacology, Kyushu University of Health and Welfare, Nobeoka, Miyazaki, Japan

**Background:** The currently available antiviral drugs leads to the problem associated with antiviral resistance and there comes the need for the search of new antiviral substances. Medicinal plants plays significant role in drug discovery and keeping it in mind one such medicinal plant Sida Cordifolia L was selected and phytosterols were isolated from the plant for the investigation against HIV-1 infection in vitro/in vivo.

**Methods:** A total of six extracted were prepared from selected plant and evaluated from phytochemical property. Based on the activity in vitro, phytosterols were isolated by solvent crystallization method and tested. The cytotoxicity was carried out with vero culture by tetrazolium bromide (MTT) assay and antiviral activity was carried out by CPE, MTT antiviral and plaque assay and in vivo by therapeutic efficacy in cutaneous HSV-1 infection in Balb/C mice.

**Results:** The isolated compound stigmasterol protected the cells from HSV-1 infection by 96±1.02% where as the beta sitosterol protected by 95 ± 1.22 %. The activity was confirmed by plaque reduction assay where the selectivity index was found to be 5.31. The in vivo test was performed at a dose of 750 mg/kg/day and at this dose the skin lesions (P<0.05) were delayed significantly and the mean survival time (P<0.001) was prolonged when tested against HSV I wild type virus 7401. Acyclovir was used as standard drug from both the studies.

**Conclusion:** The finding showed that the stigmasterol isolated from the mentioned plant has good antiviral activity and may be developed as drug against HSV-I infection.

## ISSHID Abstract-385 Ocular Tuberculosis is the key to diagnose pulmonary tuberculosis

### Kalpana R

#### Sankara Eye Hospital, Chennai, India

**Background:** Tuberculosis is one of the commonest infectious diseases prevailing in our Indian population. In a few incidences ocular tuberculosis is one of the presenting features of diagnosing intrapulmonary tuberculosis.

**Case Report:** A 49 female who presented to our ophthalmology OPD with complaints of defective vision in the right eye. Patient’s visual acuity in right eye was 6/60 and left eye was 6/9. Patient presented with right eye vitritis along with choroidal tuberculoma and disc edema. Systemic examination was done and sputum AFB and quantiFERON-TB GOLD was positive and hence diagnosed as intrapulmonary tuberculosis. Antituberculosis treatment was started and patient improved symptomatically. An informed consent was obtained from the patient to publish her personal data.

**Conclusion:** In ocular tuberculosis early diagnoses and prompt intiation of treatment can prevent complete visual impairment. This report thus concludes ocular tuberculosis is one of the differential diagnosis for unilateral loss of vision. Screening all suspected tuberculosis patients and family members for ocular tuberculosis helps in preventing complications.

## ISSHID Abstract-389 Screening of Anti-Parvovirus IgG by ELISA -A pilot study from tertiary care centre

### Broodlin Selvini S.S^1^, Kavipriya.S^1^, Swathi kumari^1^, Ramya Barani^1^, Sudha bharathi R^1^, Jayakumar. M^2^, Padma Srikanth^1^

#### ^1^Department of Microbiology, Sri Ramachandra Institute of Higher Education and Research, Chennai, Tamil Nadu, India; ^2^Department Nephrology, Sri Ramachandra Institute of Higher Education and Research, Chennai, Tamil Nadu, India

**Background:** Parvo virus B19 is ssDNA virus, It causes wide spectrum of disease ranging from erythema infectiosum in children to complex diseases like acute symmetric polyarthropathy in adults, persistent anemia in immunocompromised patient, transient aplastic crisis, spontaneous abortion and hydrops fetalis in pregnant women, graft rejection in transplant recipients. This study is to screen the parvovirus IgG among study participants.

**Methods:** Blood sample was collected from study participants. Parvovirus IgG detection was done by using Parvovirus IgG ELISA Kit (Euroimmun, Germany). Kit standards were used to obtain a standard curve. The results of the samples were interpreted as IU/ml.

**Results:** A total of 65 study participants were enrolled for detection of parvovirus B19. Of which 68% (n=44) were male and 32% (n=21) were female. Among them, 62% (n=40) of participants were tested positive for IgG which showed equal distribution among both female (62%) and male (61.4%). Majority of IgG positive cases (47%) were elderly patient followed by adult (37%) and lowest in children (16%). Majority of IgG was detected in patients from nephrology 65% (n=28/43), 57% (n=4/7) in urology and 53% (n=8/15) from gastroenterology.

**Conclusion:** High index of clinical suspicion and prompt diagnosis of Parvovirus B19 is needed to prevent complications like severe anemia and pure red cell aplasia. Parvovirus B19 directed treatment options are currently limited and optimized therapeutic strategies is required.

## ISSHID Abstract-391 Ocular Manifestations of Tuberculosis- A Clinical Study

### Deepa R, Anuradha P

#### Department of Ophthalmology, Saveetha Medical College, Chennai, India

**Background:** Tuberculosis, one of the major public health threats with high morbidity and mortality. Ocular tuberculosis is one of the extra pulmonary type of tuberculosis and is usually not associated with clinically evident pulmonary tuberculosis.

**Aim of the study:** To evaluate the prevalence of ocular manifestations of tuberculosis in pulmonary tuberculosis patients attending chest medicine OPD of our hospital. Study design- A Cross sectional prospective study, which included 93 sputum positive patients for a period of one year from June 2018 to June 2019

**Materials and methods:** Investigations like sputum AFB, CXR, Mantoux test, ESR and Quantiferon-Tb gold were done to confirm pulmonary tuberculosis .A complete ocular evaluation was done which included visual acuity, anterior segment examination and dilated fundus examination.

**Results:** Among the study population, there were 62 males(67%) and 31 females(33%) and maximum prevalence of ocular tuberculosis was observed in the age group of 41-60 years, followed by 21-40 years. Of the total 93 patients, 8 (8.60%) patients were diagnosed with ocular tuberculosis and their visual acuity was less than 6/18, among which anterior uveitis and vitritis were present in 10% of the patients, choroidal tubercule, choroidal scar and vasculitis were present in 20% of the patients. All patients were started on ATT drugs and on follow up after 1 month, visual acuity of patients with ocular tuberculosis improved.

**Conclusion:** Thus early diagnosis and treatment of ocular tuberculosis will reduce the cause of preventable blindness in our Indian population.

## ISSHID Abstract-400 Detection of Cytomegalovirus by real time PCR among pediatric patients in different samples

### amya Dharnidhar^1^, Kousalya V^1^, Ramya Barani^1^, Monika Mani^1^, Krithika Gopalakrishnan^1^, Padmasani LN^2^, Rajakumar PS^2^, Krishnarathinam Kannan^3^, Padma Srikanth^1^

#### ^1^Department of Microbiology, Sri Ramachandra Institute of Higher Education and Research, Chennai, Tamil Nadu, India; ^2^Department of Pediatrics, Sri Ramachandra Institute of Higher Education and Research, Chennai, Tamil Nadu, India; ^3^Department of Oncology, Sri Ramachandra Institute of Higher Education and Research, Chennai, Tamil Nadu, India

**Background:** Cytomegalovirus (CMV) is a leading cause of congenital infection resulting in hearing loss and systemic infection. Virus latency and reactivation can cause compartmentalized disease and transient viremia. CMV was reported in urine and saliva among pediatric patients in the absence of CMV viremia. To improve the detection rate of CMV among pediatric patients we used multiple samples to rule out CMV infection by real time PCR.

Methodology: Pediatric patients suspected for CMV infection were included. Both blood/urine/saliva was collected and participants who consented to give more than one samples were included. DNA was extracted and quantitative real time PCR (R-gene kit) was performed targeting ppUL83. The lower detection limit is <50 copies/ml.

**Results:** A total of 119 study participants were enrolled. In all, 54% (n=64) were male 46% (n=55) female. Samples were collected from infants (n=24) neonate (n=48); pediatric (n=47). About 24% (n=29) had CMV infection. In single sample testing, CMV was detected in 11 participants, including blood (n=6), urine (n=4); saliva (n=1). In four participants CMV was detected in all three samples. Five participants were found to be positive for CMV in urine only. CMV was detected only in blood in two patients. Five participants were found be positive for CMV in paired sample (B&U-1; B&S-2; U&S-2).

**Conclusion:** Detection of such infection by real-time PCR using specific gene is lifesaving in children with impaired immunity. Therefore detection of CMV in urine and saliva in first two weeks of life may help to prevent other complications.

## ISSHID Abstract-401 Drug-resistant genes in bacteria causing polymicrobial infections in HIV patients from Southern India

### Marimuthu Ragavan Rameshkumar^1^, Narasingam Arunagirinathan^1,2^, Balasubramaniam Senthamilselvan^1^, Chinnambedu Ravichandran Swathirajan^3^, Sunil SuhasSolomon^3,4^, Ramachandran Vignesh^3,5^, Saravanan Shanmugam^3^, Pachamuthu Balakrishnan^3^

#### ^1^Department of Microbiology and Biotechnology, Presidency College (Autonomous), Affiliated to University of Madras, Chennai, Tamil Nadu, India; ^2^Central Research Laboratory, Meenakshi Academy of Higher Education and Research (Deemed to be University), Chennai, Tamil Nadu, India; ^3^Infectious Diseases Laboratory, Y.R. Gaitonde Centre for AIDS Research and Education, Voluntary Health Services Hospital Campus, Chennai, Tamil Nadu, India; ^4^Department of Medicine, Johns Hopkins University School of Medicine, Baltimore, Maryland, United States; ^5^Preclinical Department, Faculty of Medicine, Universiti Kuala Lumpur Royal College of Medicine Perak (RCMP UniKL), Ipoh, Malaysia

**Background:** HIV infected individuals have high risk of polymicrobial infections (PIs) caused by more than one bacterial pathogens especially in urinary and respiratory tracts due to their immune deficiency. High exposure for prophylactic antibiotic therapy is influencing the emergence of drug-resistant bacteria which become a serious threat and also limit the effective antimicrobial treatment.

**Methods:** Bacterial strains from polymicrobial infections in HIV patients were isolated and identified using conventional culture and identification methods. Antibiotic susceptibility of the bacterial isolates was screened using Kirby-Bauer disc diffusion method and the occurrence of drug-resistant genes was analyzed using polymerase chain reaction method.

**Results:** Among 26 HIV patients having polymicrobial infections, 52 bacterial strains were isolated and 69.2% of PIs were caused by two different Gram-negative bacteria and remaining 30.8% were by two different Gram-negative and Gram-positive bacteria. High rate of PIs was found in urine samples (50%) followed by pus (30.8%) and sputum (11.5%) samples. CD4 cell count of the studied HIV patients was ranged from 34 to 743cell/mm3. *Klebsiella pneumoniae* and Pseudomonas aeruginosa (23.1%) combination caused highest level of PIs followed by *K.pneumoniae* and *Staphylococcus aureus* (15.4%), *K.pneumoniae* and *Proteus mirabilis* (11.5%), and *Escherichia coli* and *K.pneumoniae* (11.5%). Molecular study reveals that sul2(76.7%) was the most detected gene among bacteria causing PIs followed by TEM (62.3%),CTX-M(60.5%), OXA (46.5%), IntI1 (34.9%), sul1 and DfrA7(30.2%), SHV (20.9%), DfrA7 (11.6%), NDM-1 and CIT-M(4.6%) and IntI2, ACCM and DfrA5 (2.3%).

**Conclusion:** Dissemination of multiple drug-resistant genes among bacteria causing PIs might limit the antibiotic treatment options in HIV patients.

## ISSHID Abstract-405 Prevalence of needle stick injuries in health care workers in a Government tertiary care hospital

### M.K.Ismath Jahan, M.Kavitha, S.Nirmala, Subathra, V.Aruna, Divya, S.Raj Arul Mercy, Selvarani

#### Department of Microbiology, Government Mohan Kumaramangalam Medical College Hospital, Salem, Tamilnadu, India

**Background:** Accidental needle stick injuries (NSI’s) are an occupational hazard for health care workers (HCW’s).HCW’s are at increased risk when they work with hollow -bore needles.

**Methods:** This is a retrospective study of NSI’s in HCW’s of GMKMCH, Salem from June 2013 to August 2019.The HCW’s who sustained in NSI were Doctors, CRRI’s, Post Graduates, Lab technicians, DMLT Students, housekeeping staff, staff nurse and nursing students.

**Results:** A total of 117 needle stick injuries were reported. Of them,38(32.4%) were nursing students, 23(19.65%) were housekeepers,19(16.23%) were CRRI, 14(11.96%) were lab technicians,10(8.54%) were nurses,6(5.12%) were post graduates, 4(3.41%)were surgeons and three(2.56%) were others. Among the postgraduates, MD pathology had sustained NSI during FNAC. Nursing students and housekeepers were a significantly larger proportion of staff sustaining NSIs (P < 0.001). Among the HCW’s with NSIs, 84 (71.7%) were with work experience of less than 1 year. In our institution with 1500 HCW’s, approximately 8% are in their first year of service at any given time. Based on this, the proportion of NSIs among those with a work experience of less than 1 year is significantly higher (P < 0.001).

**Conclusion:** A regular training activity was practiced to address the issue of NSI to MBBS Graduates during foundation course, to CRRI during induction program emphasizing segregation at the point of generation and use of PPE. For house-keeping staff training was given in their own language. Trainings were conducted for nursing students during classes with senior matrons to mentor them.

## ISSHID Abstract-406 Elucidating the Antibiofilm effect of bioactive metabolites from *Streptomyces diasticus* Strain against *Candida albicans*

### Siddharthan Seema^1^, Rajamohamed Beema Shafreen^1,2^

#### ^1^Molecular and Nanomedicine Research Unit, Centre for Nanoscience and Nanotechnology (CNSNT), Sathyabama Institute of Science and Technology, Chennai, India; ^2^Department of Biotechnology, Science Campus, Alagappa University, Karaikudi, India

**Background:***Candida albicans* is regarded to be one of most important causative agent among the Candida species to cause biofilm-mediated infection in human. Thus biofilm mediated invasive fungal infections in immunocompromised patients and emergence of resistance to the most common antifungal drugs requires development of new antifungal agents. Therefore, objective of the present study is to explore anti-biofilm activity of *S. diasticus* (SS5) extract against *C. albicans* which is of clinical importance.

**Methods:** The CV assay, light microscopy, Confocal scanning electron microscopy (CLSM) and scanning electron microscopy (SEM) were employed to determine the inhibitory effect of *S. diasticus* extract on biofilm. Toxicity studies were carried out using zebrafish embryos. Characterization of bioactive fraction was carried out using FTIR and GC-MS.

**Results:** Bioactive fraction from extract at 30μg/ml showed 83% inhibition of *C. albicans* biofilm formation. This was evident from different microscopic analysis. Light and CLSM studies revealed the reduction of biofilm formation in the presence of SS5 extract. Cell membrane ruptured was observed by SEM. From in vivo toxicity studies carried out using zebrafish embryos in the presence of bioactive fraction showed 80 % of survival rate.

**Conclusion:** These results suggest that bioactive fractions from *S. diasticus* extract showed antibiofilm activity against *C. albicans* and furthermore the fraction was characterized using FTIR and GC-MS to identify the bioactive compounds responsible for biofilm inhibition. The bioactive compound identified from *S. diasticus* extract could be considered as a potential source for antibiofilm drug development against *C. albicans* biofilm.

## ISSHID Abstract-407 Serological marker in HBeAg positive and negative individuals

### Sweatha Kumar, Monika Mani, Gopalsamy Sarangan, Shanthi Vijayaraghavan, Padma Srikanth

#### Sri Ramachandra Institute of Higher Education and Research, Chennai, Tamil Nadu, India

**Background:** In India 40 million people are infected with Hepatitis B virus (HBV). HBV causes self-limiting hepatitis to acute, chronic hepatitis, cirrhosis and Hepatocellular carcinoma. Several serological markers are available to understand disease severity in infected individuals. HBeAg is a surrogate marker for active viral replication that guides clinicians to make therapeutic decisions. In India, two-third of infected individuals are HBeAg negative. ALT and HBV DNA levels are significantly lower in HBeAg negative patients. Few HBeAg negative patients have persistent viremia and have active liver disease. In this study we compared the plasma HBV DNA levels between HBeAg negative and HBeAg positive individuals.

**Methods:** Blood samples were collected.30 HBeAg negative and 30 HBeAg positive individuals. DNA was extracted from plasma and quantified by real time PCR. Results like HBeAg, SGOT and SGPT were obtained from hospital in-patient management system. Statistical analysis was performed using Medcalc software.

**Results:** Among HBeAg negative participants, the mean age was 36+5.1 years, mean plasma HBV DNA level was 4+1.16 log10IU/mL, SGOT was 109+226 U/L, SGPT was 131+344 IU/mL. In all, 42% had chronic liver disease. Among HBeAg positive participants, mean age was 46+15.8 years and mean plasma HBV DNA level was 6+1.26log10IU/mL, SGOT-481+1392 U/L, SGPT-315+893 U/L. Also 37% had chronic liver disease.

**Conclusion:** HBV DNA level was higher among HBeAg positive individuals. However, we noticed a slightly higher percentage of chronic liver disease among HBeAg negative individuals, suggests that measuring plasma HBV DNA level can be a reliable marker to assess clinical outcome.

## ISSHID Abstract-408 Developing an improved process for extraction of highly effective phytochemicals from the aromatic plant *Nigella sativa* (Black cumin) and to exploit its potential in ayurvedic formulations research

### Kalkatanur Ganesan Vanitha1, Madheshwar Rajha Viknesh, Natesan Sudhakar

#### Microbiology Department, Muthayammal College of Arts, Rasipuram, Namakkal district, TamilNadu, India

**Background:** To inhibit Antimicrobial resistance (AMR) bacteria, natural products are recommended widely by experts and users. Ancient literature reveals a traditional system called Śodhana (purification/detoxification) can influence the phytochemical, pharmacological, and toxicological profile of the plant drugs and thereby useful in increasing safety profile and efficacy of the drugs. Nonetheless, it is not widely practiced by the Pharma Industries, It is worthwhile to analyze and adopt Śodhana process for the development of more active PDA and employ for modern drug development.

**Methods:** We performed an antibacterial study on black cumin powder extracted with different solvents like Methanol, ethanol, hydro alcohol, Aqueous and the essential oil extracted from black cumin were subjected to Agar well diffusion assay(Kirby Bauer method,1960) against multidrug-resistant bacterial strains Escherichia coli, Staphylococcus aureus, Klebsiella pneumonia and also tested for their antifungal activity against pathogenic filamentous aflatoxin-producing fungi Aspergillus sp and Penicillium sp.

**Results:** Our experimental results reveal that Lime treatment shows strong inhibitory activity against all the tested microbes than the plain Black Cumin extract and antibiotics currently used. Also, Lime treated black cumin ethanol extract possess strong inhibitory activity against both the fungi Aspergillus Sps and Penicillium Sps significantly than the commercial fungicides Fluconazole & ketoconazole ( mg/ml) that are used as a positive control.

**Conclusion:** It is evident from our study that prior treatment with Lime is enriching the potential of Black Cumin extract as a whole by inhibiting the growth of pathogens.

## ISSHID Abstract-411 Diagnosis of new smear negative (NSN) Pulmonary Tuberculosis by XPERTMTB (CBNAAT)

### M.Kavitha, M.K.Ismath Jahan, Thentral, S.Raj Arul Mercy, S.Nirmala, A.V.Kanchana, Divya, Thirunavukkarasu

#### Department of Microbiology, Government Mohan Kumaramangalam Medical College Hospital Salem, Tamil Nadu

**Background:** Our study is intended to identify the number of NSN-CBNAAT positive cases and their resistance pattern and to analyse the advantage of direct screening in PLHIV using CBNAAT in Salem DTC.

**Methods:** Study Design: Descriptive study of cases enrolled during our routine diagnostic algorithm under revised national TB control Program. Study Period: January to August 2019.

**Results:** A total of 79 cases (59%) were smear negative and CBNAAT positive. All the NSN cases were sensitive to Rifampicin. There was no resistance identified. The HIV-TB co infection rate from 2015 to 2019 was 9.24%(107), 7.98%(173), 8.36%(137), 6.55%(98) and 5.21%(30) respectively. The overall HIV-TB co-infection rate is 7.75%(545).

**Discussion:** Our study shows that XPERT MTB (CBNAAT) has detected MTB in 50% of new smear negative cases (NSN). NSN patients who are CBNAAT positive (59%) are all sensitive to Rifampicin. They still can transmit infection (17%). PLHIV-TB co-infection has decreased from 9.24% to 5.21% due to direct CBNAAT testing for all HIV patients in our DTC. Early diagnosis and treatment has played a major role. It ruled out smear negative cases in PLHIV as well.

**Implications:** To motivate the young generation to join hands to END TB, a relay CME was conducted in our institution by the MBBS students where all the 100 students made 1 minute presentation on various topics in TB to the juniors. MBBS interns were involved in judging the presentations along with senior faculty with a high level of commitment.

## ISSHID Abstract-L14 Immune effector role of serum selenium among patients with Tuberculosis

### Monish. I, Andrew Pradeep. M, Nandhini.R.S, Stella Mary.J

#### Department of Microbiology, The American College, Madurai, Tamil Nadu, India

**Background:** Tuberculosis is a global public health problem caused by Mycobacteruim tuberculosis (MTB) and one among the leading infectious disease with high prevalence of mortality. Selenium is an essential trace element in all species including humans, which regulates the body physiological mechanism among the adult globally.Selenium has been recognized to play a vital role in both cellular and humoral mediated immune response and also evidenced with regulating the levels of interleukin during the susceptibility of bacterial and viral infection.

**Methods:** A total of 50 tuberculosis patients attending Anti Tuberculosis Therapy is Revised National Tuberculosis Control Program of Madurai Division in government Rajaji Hospital, Madurai were subjected in the present study. Ethical Committee constituted by Government Rajaji Hospital approved and gave ethical clearance of sample collection. Samples were collected in the informed consent of the patients.

**Result:** In the present study gender wise association of selenium with reference to *P* value was 0.1 which infers extremely significant correlation of selenium in the mechanism of immune regulation among the TB patients. This influence may be due to the activity of seleno enzymes in association with oxidative imbalance leading to the host vulnerability to resist co-infection thereby challenges immune competence.

**Conclusion:** Study concludes that selenium supplementation need to be emphasized in modulating the overall well being of the immune response and minimizing the risk of oxidative damage among patients with tuberculosis.

## ISSHID Abstract-L15 Bacteriological profile and antibiotic susceptibility pattern of urinary tract infection in children of tertiary care centre

### Akila Krishnan, Swati Kumari

#### Department of Microbiology, SRIHER

**Background:** UTI is a common bacterial illness and a leading cause of morbidity in paediatric population. UTI in young children present with non specific sign and symptom that can be misdiagnosed.

**Methods:** Retrospective analysis was done among paediatric age group (0-16 years) in SRIHER, Chennai. Results were recorded from cultured urine samples from central laboratory.

**Results:** Out of 308 urine samples, 47 (15.2%) were positive for growth of organism in culture. Boy:girl ratio was 1:2.2 .Most affected age group were infants. Most commonly isolated organism was E.coli (68%) followed by *Klebsiella pneumoniae* and Enterococcus.

Antibiogram showed overall resistance to ampicillin and cephalosporins but mostly sensitive to amikacin, nitrofurantoin, cotrimoxazole.

**Conclusion:** The presentation of paediatric UTIs varies widely and its knowledge along with risk factors and susceptible antibiotics is necessary to prevent future complications"

## ISSHID Abstract-L16 A potpourri of infection: When Fungi, Actinomycete and Bacteria coexist in the same host

### Isabella Princess B

#### Apollo Speciality Hospitals, Vanagaram, Chennai

**Background:** Common infections encountered in CKD patients are due to defective cell mediated immunity which can be related to immunosuppressant therapy. Among the list of infections, Cryptococcal sepsis is an uncommon occurrence especially in HIV negative individuals. We encountered one such patient who presented with Cryptococcosis along with other coexisting infections.

**Case report:** An elderly CKD patient with underlying DM, hypertension and CAD presented with fever, altered sensorium, seizure like episode, hypotension. He was drowsy, disoriented and unresponsive, vitals were stable. A clinical diagnosis of sepsis was made for which blood cultures were sent. Procalcitonin was elevated (≥2 and <10 ng/ml). After 72 hours of incubation, Gram stain of blood showed Gram positive spherical budding yeast cells. Capsulated yeast were appreciated on India ink. Cryptococcus neoformans was identified by automated identification system (Vitek 2 compact). He developed secondary polymicrobial bloodstream infection with Enterococcus faecalis and *Acinetobacter baumannii*. His chronic non healing leg wound grew *Nocardia brasiliensis*. His viral serology for HIV antibodies and HBsAg were negative.

**Conclusion:** Uncommon associations of Cryptococcosis in HIV negative individuals have been rarely documented in literature. Documented conditions other than HIV predisposing to cryptococcosis are immunosuppressant therapy and hepatic cirrhosis. There are no published reports of Cryptococcosis in patients with CKD. Apart from this, our patient had a rare combination of multiple infections in multiple sites of the body. It is therefore wise to consider polymicrobial infections in patients with CKD since this mandates wider and specific antimicrobial therapy.

## ISSHID Abstract-L18 Extended spectrum Beta Lactamase coding genes mediated resistance in *Klebsiella pneumoniae*

### P. John Thomas^1^, N.Soundharya^1^, D.Jayarajan^2^

#### ^1^PG Research Department of Microbiology, St.Joseph’s College of Arts and Science, Cuddalore, Tamilnadu, India; ^2^Department of MLT, Divine Mother College, Puducherry, India

**Background:** Lower respiratory infections accounted for 2.74 million deaths making them the fifth leading cause of death and leading infectious cause of death worldwide, according to data from the Global Burden of disease study. Due to the extensive use of broad spectrum antibiotics, the respiratory infections caused becomes severe complicated issues in hospitals nowadays. In addition ESBL producing strains also prevailed leads to resist beta lactamase antibiotics, thus the emergence of ESBL producing pneumonia strains limits therapeutic options contributing to the overall high mortality rates in immuncompromised patients

**Methods:** The present study evaluated the prevalence of ESBL coding gene(TEM) mediated resistance in hospitalized patients byRFLP and RAPD PCR.cDNA. 16SrRNA.

**Results:** 26 isolates of klebsiella pneumoniae were identified by 16SrRNA method and all the isolates were screened for ESBL production. Eighty percent of the isolates were found ESBL producers upon double disk synergy test. The ESBL production was further confirmed for the prevalence of ESBL encoding TEM genes which were identified in sixty percent isolates. TEM genes contributes resistance to combat therapeutic measure. All the isolates were Biofilm producers.

**Conclusion:** This study reveals that there was a strong correlation between Biofilm mechanism and TEM genes were observed during the study.

## ISSHID Abstract-L19 Reporting of notifiable diseases in a multispeciality hospital "the what, when, how and why?

### Sreevidya Subramanian, Senthur Nambi P, Debashree Banita Samal, Prema K, Sameeha Shroff, Kavitha. S

#### Apollo Speciality Hospitals, OMR Chennai, Tamil Nadu, India

**Background:** This study was undertaken in a multispeciality hospital to analyse the various notifiable diseases reported over a period of 4 years and to find out the most common diseases notified and prevalent in each season. It gives us insight into the demography and disease trend.

**Methods:** This is a hospital record based retrospective study. The data collected was tabulated using simple descriptive statistics and depicted as charts and tables

**Results:** The total number of cases reported was 6450. Only 9 diseases out of the 22 notifiable diseases listed by the government were reported. H1N1 (37.82%) was the most commonly reported disease followed by dengue (21.77%), acute febrile illness (17.18%) and acute gastroenteritis (16.58%). HIV (0.20%) and measles (0.13%) were least reported. The age group most commonly affected is 13-39 years.For all the reported diseases there is male predominance. Of total samples tested for notifiable disease in one month, 45.43% of the results were positive for notifiable infection. Dengue was more preponderant from mid August to end of October. Similarly H1N1 cases were usually seen from mid November till end of February. This was the general trend in the last four years.

**Conclusion:** There is an urgent need for the surveillance and study of notifiable diseases in order to strengthen global health security.No notifiable disease should be overlooked or undermined. The disease trends help us in emergency preparedness and action plans taken in the healthcare sector and keeping all investigation kits ready to diagnose the communicable diseases

## ISSHID Abstract-L20 Asperillosis: The Black Plague Killing the Immunosupressed

### Srikrishnan S, Priyadarshee Pradhan, Sampath Kumar

#### Dept. of Forensic Medicine and Toxicology, Sri Ramachandra Medical College and Research Institute, Chennai, India

**Introduction:** Systemic mycosis is an uncommon disease which affects patients whose immune system is compromised whose prevalence ranges from 0.98% to 10.4%. The factors attributing to fungemia are prolonged use of antibiotics, immunosuppressive agents, parenteral nutrition and failure of vital organs. The most trivial genesis for invasive mycosis is due to aspergillosis. The increase in drift has been acclaimed subsequently after organ transplantation, chemotherapy, and other antecedents of immunosuppression.

**Case Report:** We report the case of a 49-year-old male who was hospitalized due to acute exacerbation and worsening of chronic kidney disease. He was managed intensively and discharged with advice of weekly twice and regular hemodialysis. After few weeks, he was brought dead to our hospital with history of breathlessness and sudden collapse. Up on request, an autopsy was performed and vital organs such as heart, lungs and kidneys were examined, dissected and was sent for histopathological examination. Meanwhile history was recorded from the relatives, older admissions in various hospitals and medical records were perused which confirmed the diagnosis of aspergillosis which also corroborated with the findings from autopsy, which itself is a rare quintessence. Presence of peculiar and incidental findings in the 4th rib of the patient indicates a secondary infection of Tuberculosis. Consent was obtained from the next of kin to the patient for publication of the patient’s data, as the patient had expired.

**Conclusion:** This case highlights that a continual stay in the critical care unit in a tertiary center is a major source of cross and nosocomial infection which is a potential risk factor for aspergillosis. The diagnosis was based on the gross examination during the autopsy and it was further clarified by histo-pathological and microscopic examination of the organs.

## ISSHID Abstract-L7 Prevalence of oral lesions in HIV / AIDS individuals

### Karthik S, Preetha S, Vishnupriya V, Ashwin Andrews E, Ranganathan K, Umadevi K Rao, Elizabeth Joshua, Rooban T

#### Department of Oral and Maxillofacial Pathology, Ragas Dental College and Hospital, Chennai, India

**Background:** Diagnosis, prognosis and follow up of the course of the disease is facilitated by oral lesions in HIV infection / Acquired Immuno Deficiency Syndrome (AIDS). In this study, we compare the prevalence of oral lesions in HIV infected individuals before and after 2003

**Aims & Objectives:** To compare the prevalence of oral lesions in HIV infected individuals over a period of 14 years (between 2000 -2003 and 2004-2014)

**Materials and Methods:** The cohort comprised of 6055 HIV infected individuals presenting to Y.R.Gaitonde Centre for AIDS Research and Education (YRG CARE). The oral lesions were diagnosed based on EC Clearinghouse criteria (1993).Group A comprised of 1000 patients presenting with oral lesions from 2000-2003. Group B comprised of 5055 patients presenting with oral lesions after 2004-2014.

**Results:** In our study, the most predominant lesions observed in Group A were gingivitis, pigmentation followed by candidiasis. In Group B, a decline in the prevalence of gingivitis, pigmentation and candidiasis due to access to AntiRetroviral Therapy and patient awareness but an increase in leukoplakia was observed. In the prevalence of oral lesions between gender, an increase in gingivitis was observed in both the gender in group B when compared to Group A

**Conclusion:** The drop in the prevalence of oral lesions in HIV infected individuals can be attributed to increase in the use of Highly Active Anti Retroviral Therapy (HAART) regimen. Oral lesions are an important sign of immunosuppression and their diagnosis and effective management protocol may help in better quality of life of those patients.

## ISSHID Abstract-L8 Prevalence of Vitamin D deficiency among ART-naive HIV patients

### Umi Solekhah Intansari, Yanri Wijayanti Subronto, Adika Zhulhi Arjana, Usi Sukorini

#### Faculty of Medicine, Nursing, and Public Health, Universitas Gadjah Mada, Yogyakarta, Indonesia

**Background:** Vitamin D plays an important role as affects as an immunomodulatory agent. Research on the association of vitamin D and immune activation and immunodeficiency is limited, especially in Indonesia which is a tropical country with sufficient sunshine. This study aims to know the prevalence of vitamin D deficiency in human immunodeficiency virus (HIV) infection

**Methods:** This study is the fourth report from ongoing research titled "Role of Vitamin D in Human Immunodeficiency Virus Infection: Relationship Analysis of Vitamin D Levels and Immune activations" funded by the Research and Community Service Directorate, Research Strengthening and Developing the General Directorate of Research, Technology, and Higher Education Ministry, Indonesia. This was an observational study with a cross-sectional design. Subjects were taken consecutively in the ward or in the clinic of Dr. Sardjito. Subjects of this study were the rest of the blood sample of the study subjects who include in HATI study. Inclusion criteria included adult patients, all sexes, and diagnosed with HIV infection, either with or without antiretroviral therapy. Pregnant patients and not willing to follow the study were excluded.

**Results:** Sixty subjects followed this study with various clinical stage. Twenty-three subjects (38.3%) were vitamin D deficient with a cut off value of 20 ng/mL. The higher proportion was subjects with severe infection (stage 3 or 4). The proportion of subjects with a normal level of vitamin D was higher in mild groups (stage 1 or 2).

**Conclusion:** Patients with a severe infection of HIV tend to be vitamin D deficient

## ISSHID Abstract-P-1 Creeping Crypto in the crypt of gut – a case report

### Zenina R. Andrews^1^, Deepika Nair^1^, G.A. Vasugi^1^, Rithika Rajendran^1^, Rajendiran S^1^, Leena D. Joseph^1^, Dhaarani J^2^, Shanmuganathan S^3^

#### ^1^Department of Pathology, Sri Ramachandra Medical College, Chennai, Tamil Nadu, India; ^2^Department of Paediatrics, Sri Ramachandra Medical College, Chennai, Tamil Nadu, India; ^3^Department of Medical Gastroenterology, Sri Ramachandra Medical College, Chennai, Tamil Nadu, India

**Background:***Cryptosporidium* is an apicomplexan parasite of public health and veterinary importance that causes a respiratory and gastrointestinal illness (cryptosporidiosis) that primarily involves watery diarrhoea with or without a persistent cough. Unlike other waterborne pathogens, *the organism* is chlorine-resistant. This unique characteristic explains why it is one of the most common cause of illness in pool swimmers. The infection is prone to become severe or life-threatening in immunocompromised individuals.

**Case Report:** An eight-year old, developmentally normal male child with a known case of common variable immunodeficiency presented with a history of fever, passage of loose stools and colicky-abdominal pain lasting more than two weeks. On examination, the abdomen was soft, no tenderness or palpable organomegaly was noted. Upon evaluation, CBC showed leucopenia, while other routine blood parameters analysed were within the normal range. Stool routine and culture revealed no abnormality. There was no response to intravenous antibiotics for ten days.

During the course of the hospital stay, the child developed skin lesions suggestive of vasculitis with erythema nodosum. An endoscopy performed in view of tuberculosis and inflammatory bowel disease revealed a normal mucosal study. Biopsies were taken from the antrum, ileum, colon and rectum. To our surprise, the colonic biopsies revealed 2-5 μm basophilic round bodies protruding from the apex of enterocytes within the cell membrane. The patient is on anti-parasitic medication (Nitazoxanide) and regular intravenous immunoglobulin. Informed consent has been taken from parents for publication of their personal data.

**Conclusion:** High index of suspicion is required to detect and determine the atypical infection and atypical manifestation of common diseases in a patient with primary immunodeficiency.

## ISSHID Abstract-P-2 Acute kidney injury in a child with complicated malaria

### Preethi, Sangeetha G, Monika, Ramachandran P, Shuba S, Rajakumar PS, Dinesh kumar J, Subbarao P, Mahalakshmi R

#### Department of Paediatrics, Sri Ramachandra Institute of Higher Education & Research, Chennai, Tamil Nadu, India

**Background:** Malaria is the most prevalent endemic disease all around the world especially in the tropical region. Here we present a child with complicated malaria and acute kidney injury (AKI).

**Case report:** A 7 years old boy with Down’s syndrome presented with fever for a week. He also had abdominal pain and malena for 2 days. On examination, he was pale, icteric, edematous, stunted with hepatomegaly (5cm) and abdominal distension. His investigations revealed severe anaemia, thrombocytopenia, elevated blood urea nitrogen, creatinine, leucocyte dehydrogenase and liver enzymes. His complement C3 was low. Peripheral smear clinched the diagnosis of falciparum malaria. Diagnosis of complicated malaria with AKI was entertained. Though he had seizures, his sensorium was normal, hence did not consider cerebral malaria. In view of anuric AKI, haemodialysis (HD) was initiated. He was treated with injection artesunate. He was anuric for 11 days, needed 5 cycles of HD and renal functions recovered after 2 weeks. Acute kidney injury in malaria is multifactorial includes hemodynamic dysfunction, hypovolemia, vasoconstriction, haemolysis, immune response and hepatic dysfunction. This child had haemolysis and hepatic dysfunction. Requirement of dialysis had been reported in 46 to 76% of complicated malaria and 64% had complete renal recovery. Informed consent has been taken from parents for publication of their personal data.

**Conclusion:** Complicated malaria is a medical emergency and should be treated urgently and aggressively. In associated AKI, early initiation of dialysis gives better outcome.

## ISSHID Abstract-P-3 An unusual case of hyperleucocytosis in an infant

### Gracelin Jeyarani, Shuba, Vidya Krishna

#### Department of Paediatrics, Sri Ramachandra Institute of Higher Education and Research, Chennai, India

**Background:** Hyperleukocytosis refers to a total white cell count greater than 50,000/microL. Leukemoid reaction is defined as a variable degree of leucocytosis with immature precursors and can be due to severe bacterial infections. We describe here a case of a 6 months old boy who presented with a marked leukocytosis with septic shock.

**Case report:** A 6 month old male child born to 3rd degree consanguinous marriage with history of previous sibling deaths presented to ER in septic shock. Child received antibiotics and fluid boluses following which he improved. Initial investigations showed marked hyperleukocytosis (Max value- 1,04,300/microL). Peripheral smear did not show any blast cells. Baby had history of slightly delayed falling of umbilical cord (on day 10) and omphalitis in the neonatal period. Blood cultures were sterile. Child had an umbilical induration which developed into an enterocutaneous fistula. In view of suspected immunodeficiency (LAD), Flowcytometry was done and CD 18 and CD 11 were deficient. Hence diagnosis of LAD type 1 was confirmed.

**Discussion:** LAD-1 is characterized by recurrent bacterial and fungal infection. Despite striking neutrophilia, neutrophils will have significant defect in adhesion. Diagnosis is established by flowcytometric measurements of CD11b/CD18.Early allogenic haematopoietic stem cell transplantation is the treatment of choice. Children with severe degree of Beta 2 integrin deficiency may die in infancy, moderate deficiency may have relatively long survival. Informed consent has been taken from parents for publication of their personal data.

**Conclusion:** Although modern flowcytometry studies have facilitated the diagnosis, the practitioners must be aware of the clinical features that point towards this rare entity.

## ISSHID Abstract-P-4 Incidence and outcome of central line associated bloodstream infection in children treated for cancer

### Balaji T K^1^, Dhaarani Jayaraman^1^, Julius Xavier Scott^1^, Latha M S^1^, Shruti T K^2^, Prakash Agarwal^3^, Madhu R^3^, Ranjith Karthekeyan^4^, Uma Sekar^5^, Vidya Krishna^2^

#### ^1^Paediatric haemato-oncology division, Department of Paediatrics, Sri Ramachandra Institute of Higher Education and Research, Chennai, Tamil Nadu, India; ^2^Department of Paediatrics, Sri Ramachandra Institute of Higher Education and Research, Chennai, Tamil Nadu, India; ^3^Department of Paediatric surgery, Sri Ramachandra Institute of Higher Education and Research, Chennai, Tamil Nadu, India; ^4^Department of Cardiac Anaesthesia, Sri Ramachandra Institute of Higher Education and Research, Chennai, Tamil Nadu, India; ^5^Director, Central Laboratory Services, Sri Ramachandra Institute of Higher Education and Research, Chennai, Tamil Nadu, India

**Background:** Central line associated bloodstream infection leads to prolonged morbidity and even mortality especially in children with cancer. We conducted a retrospective study to analyze the incidence and outcome of central line associated bloodstream infection (CLABSI) in our paediatric haemato-oncology unit during the period of September 2018 to August 2019.

**Methods:** This is a retrospective study of children treated for cancer who required central venous access (port line, PICC line and Central venous line) for chemotherapy during the period of September 2018 to August 2019. Children who developed CLABSI were analysed for type of organism involved and outcome in terms of salvageability of central line.

**Results:** In 125 new cases admitted, 51 children required central venous access for chemotherapy. Out of 51 central venous lines placed 20 (42%) developed CLABSI. The most common organism involved being Klebsiella with 5 out of 20 central lines (25%). Three blood culture grew multiple organisms. With systemic antibiotic therapy, antibiotic lock, weekly dressing and usage of bionectar, we salvaged 16 out of 20 (80%) central lines. One child expired due to sepsis.

**Conclusion:** During our study period we found that 42% of the children developed CLABSI which is a significant number. We were able to salvage 80% of central lines with prompt intervention.

IEC approval has been obtained from institution ethics committee.

## ISSHID Abstract-P-5 Fulminant subacute Sclerosing Panencephalitis in a toddler

### Samyuktha Sivakumar, Shuba S, Shruthi TK, John, Rajakumar PS

#### Department of Paediatric Medicine, Sri Ramachandra Institute of Higher Education and Research, Chennai, India

**Background:** Subacute Sclerosing Panencephalitis (SSPE) is a late complication of Measles infection and usually occurs several years after Measles infection. Here we report a case of SSPE in a toddler due neonatal Measles infection.

**Case Report:** An 18 month old, developmentally normal, female child presented with fever for 15 days, inability to sit and stand from lying position and head lag since day 3 of fever. Child was given intravenous antibiotics and fluids, but did not improve. She suffered from intermittent episodes of jerky movements of all four limbs lasting a couple of minutes, without loss of consciousness; Child was active after that. Child developed repeated seizures and respiratory distress. Hence child was intubated and ventilated. Mother gave history of Measles infection of both mother and child when the child was 11 days old. Measles Immunoglobulin M antibody in cerebrospinal fluid and serum was positive. Magnetic Resonance Imaging of brain and spine showed bilateral frontal and right parietal white mater demyelinating changes. Diagnosis of Subacute Sclerosing Panencephalitis was made. Intravenous Methylprednisolone, Topiramate and Clonazepam were started and Intrathecal Interferon injection was advised. Child had extubation failure and needed prolonged hospital stay. Due to financial constraints, parents decided to discharge the child against medical advice. IEC approval has been obtained from institution ethics committee. Consent has been taken from the parents for the publication of child’s data

**Conclusion:** SSPE can occur early in Measles infections occurring in infancy.

## ISSHID Abstract-P-6 Clinical Profile and Predictors of Outcome for Paediatric Scrub Typhus at a Tertiary Care Hospital in South India

### Kiruthika VM, Shruthi TK, Shuba S, Rajakumar PS, Krithika P, Balaji TK

#### Department of Paediatric Medicine, Sri Ramachandra Institute of Higher Education and Research, Chennai, India

**Background:** Scrub typhus is an important cause of acute undifferentiated fever in India. It is the most common Rickettsial infection caused by Orientia tsutsugamushi transmitted by bite of larval stage of Leptotrombidium mite. Untreated cases can have mortality rates as high as 30-35%.

The objective was to study the demographical data, clinical profile and predictors of outcome for scrub typhus in children who were admitted to SRIHER Hospital, Chennai, South India.

**Methodology:** In this retrospective study, children diagnosed with scrub typhus based on IgM ELISA, between January 2012 and June 2019 were included. Demographical data and clinical profile were analysed using SPSS software and predictors by univariate analysis.

**Results:** A total of 120 patients were identified, among them 84 (Males-42, Females- 42) satisfied the inclusion criteria. 80 patients (95%) had complete recovery, 4 patients (5%) died of multiple complications. Months September to December had higher incidence, common among 12-18 age group and year 2012 had maximum cases. Eschar was seen in 37% cases. Deranged Liver function & Thrombocytopenia were common. Shock (p=0.0005 & odds ratio=13.9)(95% confidence interval-C.I=1.59-127), ARDS (p=0.001 & odds ratio= infinity)(C.I=NIL), AKI (p=0.00 & odds ratio= infinity)(C.I=NIL), DIC (p=0.001 & odds ratio= infinity)(C.I=NIL) and MODS (p=0.001 & odds ratio=59)(C.I=8.45-412) were significantly associated with prolonged hospitalisation and increased mortality.

**Conclusion:** Paediatric scrub typhus should be suspected in any case with fever for more than 5 days and children with hypotension, hypoxemia, oliguria with azotaemia and bleeding tendencies at admission have guarded prognosis.

IEC approval was obtained from institution Ethics Committee.

## ISSHID Abstract-P-7 Infections and malnutrition

### Reshma Ramesh, Padmasani Venkatramanan, Sriram Hariharan

#### Department of Paediatrics, Sri Ramachandra Institute of Higher Education and Research, Chennai, Tamilnadu, India

**Background:** Severe acute malnutrition (SAM) is known to be associated with increased risk of infections and poorer outcomes. The current study describes the infections identified in children with SAM hospitalized with fever without focus and their outcome as compared to normally nourished children.

**Methods:** This case control study was done in a teaching hospital in South India with the permission of the Institutional Ethics Committee. Children with SAM (WHO criteria), hospitalized between May 2019 and August 2019 with fever < 7 days without an obvious focus (FWF) were compared with age and diagnosis matched normally nourished hospitalized children. Statistical analysis was done and the Odd’s ratio for having a serious bacterial infection was calculated for each group.

**Results:** Twenty seven children with FWF in each group were included. 25.9% children with SAM had bacterial growth in blood culture and 44.4% in urine culture as compared to 1 (3.7%) and 3 (11.1%) in normally nourished children respectively. This difference was statistically significant (OR = 1.17 and OR = 1.32 respectively). 7.4% of malnourished children had enteric fever as compared to 3.8% of normally nourished children. The Average Length of Stay (ALOS) was also significantly more in children with SAM hospitalized with viral fever, urinary tract infection and enteric fever.

**Conclusion:** In under 5 children presenting with fever without focus, children with SAM were more often found to have serious bacterial infections. The ALOS was significantly higher in children with SAM as compared to age matched, normally nourished children

IEC approval has been obtained from institution ethics committee.

## ISSHID Abstract-P-8 Factors influencing severity of illness in children with Bronchiolitis

### Kadiyala Apurva, Kaveri S, Padmasani Venkatramanan

#### Department of Paediatrics, Sri Ramachandra Institute of Higher Education and Research, Chennai, Tamilnadu, India

**Background:** Bronchiolitis is a common cause for respiratory illness (LRI) in young children. The aim of this study was to identify the factors associated with its severity among hospitalised children.

**Methodology:** The prospective study was done with the approval of the Institutional Ethics committee from September 2016 to August 2017 among children hospitalised with bronchiolitis. Children with comorbidities were excluded. Severity was described using the maximum clinical severity score ( between 0 to 8 ) during hospitalization based on SpO2 ( > 95% = 0 , 91-95%=1, <91% = 2) , Retraction (None = 0 , Present =1, Present with nasal flare = 2 ), Ability to feed ( Normal= 0, Reduced = 1, Requires IVF = 2) and Respiratory rate ( <45/min=0 , 45-60/min =1, >60/min = 2). Mild severity was taken as a score < 3, moderate as 3-6 and severe as > 6. Multivariate analysis was done to identify the independent risk factors

**Results:** Out of the 150 children in our study, 35 (23.3%) had a score of < 3, 69 (46%) had 3 to 6 and 46 (30. 7%) had > 6. The independent risk factors for moderate to severe bronchiolitis (Score > 3) were RSV positivity (OR 1.46) , preterm birth (OR1.57) , passive smoking (OR 1.64) and family history of asthma (OR 1.63).

**Conclusion:** Children with these risk factors should be monitored for severe disease. Anticipatory guidance to parents to avoid smoking is important since it is the only preventable factor.

IEC approval has been obtained from institution ethics committee.

## ISSHID Abstract-P-9 Pyogenic Liver abscess, complicating Urinary Tract Infection in a toddler

### Subiksha K, Latha Ravichandran, Sarala Premkumar, Elayaraja Sivaprakasam

#### Department of Paediatrics, Sri Ramachandra Institute of Higher Education and Research, Chennai, Tamil Nadu, India

**Background:** Pyogenic liver abscess is a disease characterised by solitary or multiple collections of pus within the liver. The most common causative organisms are *Escherichia coli* and Klebsiella. Pyogenic liver abscess, in a child with urinary tract infection (UTI) is a rare complication.

**Case report:** A 3 year old female child came to SRIHER with complaints of fever for 5 days which was low grade and intermittent. She was diagnosed with congenital right dysplastic kidney. The child was given amikacin for UTI in a nearby hospital as urine culture grew Escherichia coli, but fever continued. She was referred here for further management. There was a previous episode of UTI 1 year back.

Systemic examination was normal. Blood investigations showed anaemia and leucocytosis. Urine culture grew *E. coli*. USG KUB showed right dysplastic kidney. Child was given piperacillin tazobactam for UTI, but fever spikes continued and the child did not improve. Further investigations were done. USG abdomen showed cystic lesion in the liver. CECT abdomen revealed a large abscess in caudate lobe of liver. Pus from liver abscess (pigtail drainage) showed sterile necrotising inflammatory process. AFB and PAS stains were negative.

Patient was started on Cefaperazone Sulbactam and Metronidazole. Repeat urine culture showed no growth and IV antibiotics was continued for 14 days. Patient clinically improved and was discharged on oral antibiotics. Informed consent has been taken from parents for publication of their personal data.

**Conclusion:** Any child with primary infection not responding to appropriate antibiotics, a search should be made for secondary septic focus and must be treated early.

## ISSHID Abstract-P-10 A rare case of *Salmonella typhimurium* induced AKI and rhabdomyolysis

### Divya R, Sangeetha G, Umapathy P, Shuba S, Rajakumar PS, Shruthi TK, Krithika P, Ram Mohan

#### Department of Paediatrics, Sri Ramachandra Institute of Higher Education & Research, Chennai, Tamil Nadu, India

**Background:** Salmonella typhimurium infection usually presents with acute enteritis. However, rhabdomyolysis with acute kidney injury is a rare presentation in non-typhoidal salmonella species induced septicaemia (NTS). The incidence of such invasive NTS infections is extremely low, even in endemic areas.

**Case Report:** A previously healthy immunocompetent 3year old female child came to ER with high grade fever, vomiting, multiple episodes of diarrhoea for 2 days and an episode of seizure. On presentation she was drowsy with a GCS of 8/15. She was febrile, tachypnoeic, tachycardic with cold peripheries and feeble peripheral pulses. She was intubated and mechanically ventilated. In view of hypovolemia and suspected septic shock, she received fluid boluses, started on ionotropic support and IV antibiotics. Investigations revealed severe metabolic acidosis, deranged renal function and increased creatine phosphokinase suggestive of anuric AKI secondary to sepsis and rhabdomyolysis. Subsequently she developed microangiopathic haemolytic anaemia, deranged coagulation profile and elevated transaminases. She was initiated on peritoneal dialysis and her urine output improved gradually with RFT showing an improving trend. Her blood culture showed Salmonella typhimurium and the diagnosis of non-typhoidal Salmonella species induced septicemia with Multiple Organ Dysfunction Syndrome (MODS) and acute kidney injury secondary to rhabdomyolysis was reached. Informed consent has been taken from parents for publication of their personal data

**Conclusion:** An immunocompetent child presenting with S. typhimurium septicemia resulting in MODS is rare. Also, rhabdomyolysis in such cases is highly unusual. A high index of suspicion for early recognition of rhabdomyolysis, extensive fluid replacement and appropriate treatment with antibiotics may help to reduce significant morbidity and mortality.

## ISSHID Abstract-P-11 A rare complication of a common cold – A case report on extra pulmonary manifestation of *Mycoplasma Pneumoniae*

### Sakti Priya, Pinnaka Subbarao, Dinesh kumar J, Sangeetha G, Ramachandran P

#### Department of Paediatrics, Sri Ramachandra Institute of Higher Education and Research, Chennai, India

**Background:***Mycoplasma pneumoniae* is an important etiological agent in Community Acquired Pneumonia (CAP) It is frequently seen in children 3 to 15 years of age. *Mycoplasma pneumoniae* may present with varied extra pulmonary manifestation.

**Case report:** A 5 year old child presented with fever and cough for 6 days. On examination, she was febrile, tachypnoeic with sub costal and intercostal retractions.

Chest X ray showed left lower lobe opacity. Cultures were sent and child was started on empirical amino penicillin. On day 3 of admission child persisted to have fever with no clinical improvement. CT showed left lower lobar consolidation and bilateral pleural effusion. Antibiotics were escalated in view of increasing oxygen requirement. Mycoplasma serology was positive and started on macrolides.

On day 10 of admission, she developed erythema and fissuring of lips with discoloration of extremities. Direct Coombs test, cold agglutination test (1:256), ANA, anti-cardiolipin antibody were positive. Suspecting small vessel vasculitis, she was started on enoxaparin and aspirin. Discolouration of toes improved within a couple of days.

Extra pulmonary manifestations of Mycoplasma Pneumonia can happen in up to 25% of patients. They include haematological, small vessel vasculitis, cardiovascular, dermatological, neurological and renal manifestations. Pathogenesis of the extrapulmonary manifestations are not clear but probably due to direct /indirect type. Treatments for complications include corticosteroids and immunomodulator therapy. Informed consent has been taken from parents for publication of their personal data.

**Conclusion:** One should always keep the differential diagnosis of extra pulmonary manifestations of *Mycoplasma pneumonia* in a child with respiratory symptoms and vasculitis manifestations.

## ISSHID Abstract-P-12 Clinical profile of Tuberculosis in Adolescents from South India

### Premkumar P^1^, Sarala Premkumar^1^, Latha Ravichandran^1^, Pulkit Malhotra^1^, Gayathri T^2^

#### ^1^Department of Paediatrics, Sri Ramachandra Institute of Higher Education and Research, Chennai, Tamil Nadu, India; ^2^Department of Allied Health Sciences, Sri Ramachandra Institute of Higher Education and Research, Chennai, Tamil Nadu, India

**Background:** Tuberculosis (TB) is a major infectious disease causing significant morbidity and mortality amongst adolescents. The aim of the current study is to find the clinical profile of Tuberculosis in adolescents (10-18yrs) from a tertiary care hospital in Chennai, Tamil Nadu.

**Methods:** The study was done from our hospital records with study period from July 2017 to June 2019 including adolescents with Tuberculosis, belonging to both upper and lower middle socioeconomic status.

**Results:** Overall 80 adolescents with tuberculosis were analysed (46.3%- male) with early adolescents (10-13 yrs- 26.3%), mid adolescents (14-15yrs- 35%) and late adolescents (16-18yrs- 38.8%). The most common presenting complaint was fever (60%) followed by loss of weight (47.5%) and cough (40%). Mantoux test was negative in 76.2% and sputum AFB was negative in 95.2% of early adolescents (P value <0.05). History of contact with tuberculosis was seen in only 16.3% of adolescents with Genexpert positivity in 28.8%. ESR was elevated in 63.8% of adolescents. Pulmonary Tuberculosis was seen in 50% (40/80) cases (52.5% sputum positive and 47.5% sputum negative) with predominance in late adolescents (64.5%). The extra pulmonary manifestation was present in 50% (40/80) of cases with predominance in early adolescents (15/21) 71.5%. We had 7 cases of MDR (multi drug resistant) TB (8.75%) and 2 cases of HIV positivity (2.5%).

**Conclusion:** The two important factors that we have to consider during adolescence are TB-HIV co-infection and MDR TB. The adolescents should be routinely screened for tuberculosis, so that disease transmission can be prevented.

IEC approval has been obtained from institution ethics committee.

## ISSHID Abstract-P-13 Acute leukoencephalopathy with restricted diffusion (ALERD) as a complication of Dengue infection

### Devaram Sowmya^1^, Ranjith kumar Manokaran^2^, Padmasani Venkat ramanan^1^, Shuba S^1^_,_ Rajakumar PS^1^

#### ^1^Department of Paediatrics, Sri Ramachandra Institute of Higher Education and Research, Chennai, Tamil Nadu, India; ^2^Paediatric Neurology Division, Department of Neurology, Sri Ramachandra Institute of Higher Education and Research, Chennai, Tamil Nadu, India

**Background:** Dengue is a common viral infection worldwide and its neurotropism is well known. Neurological complications previously described include encephalopathy, encephalitis, immune-mediated syndromes and dengue-associated stroke. We present here a child with acute leukoencephalopathy with restricted diffusion (ALERD) triggered by dengue infection. This is the first report of this association to the best of our knowledge.

**Case Report:** A 9 month old girl presented with complaints of loose stools for three days, fever for one day, seizure followed by status epilepticus. She was intubated and seizures were controlled with four antiepileptics. Evaluation for cause of fever revealed Dengue infection. Her biochemical, metabolic, electrolyte parameters, CSF analysis were normal. On day five of illness she again developed left focal seizures followed by left hemiparesis and persistent drowsiness. MRI brain showed areas of restricted diffusion in the deep cerebral white matter of bilateral frontal, right parietal lobes and bilateral thalami without any signal changes in the corresponding T2 weighted/FLAIR images establishing the diagnosis of ALERD, with a classic central sparing pattern. Child was treated with IVIG following which neurological symptoms improved.

ALERD is a clinico-radiological diagnosis. Previously termed acute encephalopathy with biphasic seizures and late restricted diffusion (AESD), it develops in association with a systemic or CNS viral or bacterial infections. It is hypothesised that hypercytokinemia causing neuronal damage plays an important role. Informed consent has been taken from parents for publication of their child’s personal data.

**Conclusion:** Many novel treatable causes of acute encephalopathy are emerging. Identification and prompt treatment of the cause is invaluable to improve outcomes.

## ISSHID Abstract-P-14 HHV-6 encephalitis in infants – documentation of rare entity

### Silky Agrawal^1^, Balasubramanian S^2^, Dhanalakshmi K^1^, Sumanth A^2^

#### ^1^Department of Paediatric Infectious diseases, Kanchi Kamakoti Childs Trust Hospital, Chennai, India; ^2^Department of Paediatrics, Kanchi Kamakoti Childs Trust Hospital, Chennai, India

**Background:** Human herpes virus 6 (HHV – 6) is one of the newly identified herpes group of virus. A population-based study in USA states that 77% of children had primary HHV-6 infection by 24 months of age. Similar study in Japan showed 86% seroprevalence. Most children are asymptomatic and most common clinical presentation in symptomatic is Roseola (Exanthem Subitum). HHV-6 infection and association with febrile seizure is increasingly recognised but underreported or remains undiagnosed in India. HHV-6 encephalitis is rare manifestation but can have permanent neurologic sequelae. CSF analysis may be normal or suggestive of aseptic meningitis; PCR assay for HHV-6 DNA is reasonably sensitive and specific.

**Case Report:** We present 3 cases of HHV-6 encephalitis, all presenting with short febrile illness and multiple episodes of seizures. Diagnosis was confirmed within hours of presentation by CSF viral DNA-PCR assay and antibiotics empirically started were stopped. All were immunocompetent and recovered without antiviral therapy, one case had difficult to control seizures requiring multiple anti-epileptics (AEDs). There was no mortality and two cases are seizure free off AEDs. This is probably first case series of HHV-6 encephalitis to be published in India to our knowledge.

**Conclusion:** This case series highlights HHV-6 as important cause to be considered in infants with encephalitis and multiple seizures. CSF PCR study is a rapid test with good sensitivity and specificity and is of significant value in antibiotic stewardship.

Informed consent has been taken from parents.

