# Correction to: Abstract-400 Detection of Cytomegalovirus by real time PCR among pediatric patients in different samples’

**DOI:** 10.1186/s12879-020-05119-y

**Published:** 2020-07-06

**Correction to: BMC Infectious Diseases (2020) 20:324**

**https://doi.org/10.1186/s12879-020-05038-y**

Ramya Dharnidhar^1^, Kousalya V^1^, Ramya Barani^1^, Monika Mani^1^, Krithika Gopalakrishnan^1^, Padmasani LN^2^, Rajakumar PS^2^, Krishnarathinam Kannan^3^, Padma Srikanth^1^

^1^Department of Microbiology, Sri Ramachandra Institute of Higher Education and Research, Chennai, Tamil Nadu, India

^2^Department of Pediatrics, Sri Ramachandra Institute of Higher Education and Research, Chennai, Tamil Nadu, India

^3^Department of Oncology, Sri Ramachandra Institute of Higher Education and Research, Chennai, Tamil Nadu, India

After publication of this supplement [1], it was brought to our attention that an author’s name has been erroneously spelled. It should be read “Ramya Dharnidhar’” instead of “amya Dharnidhar’”.

Background: Cytomegalovirus (CMV) is a leading cause of congenital infection resulting in hearing loss and systemic infection. Virus latency and reactivation can cause compartmentalized disease and transient viremia. CMV was reported in urine and saliva among pediatric patients in the absence of CMV viremia. To improve the detection rate of CMV among pediatric patients we used multiple samples to rule out CMV infection by real time PCR.

Methodology: Pediatric patients suspected for CMV infection were included. Both blood/urine/saliva was collected and participants who consented to give more than one samples were included. DNA was extracted and quantitative real time PCR (R-gene kit) was performed targeting ppUL83. The lower detection limit is < 50 copies/ml.

Results: A total of 119 study participants were enrolled. In all, 54% (*n* = 64) were male 46% (*n* = 55) female. Samples were collected from infants (*n* = 24) neonate (*n* = 48); pediatric (*n* = 47). About 24% (*n* = 29) had CMV infection. In single sample testing, CMV was detected in 11 participants, including blood (*n* = 6), urine (*n* = 4); saliva (*n* = 1). In four participants CMV was detected in all three samples. Five participants were found to be positive for CMV in urine only. CMV was detected only in blood in two patients. Five participants were found be positive for CMV in paired sample (B&U-1; B&S-2; U&S-2).

Conclusion: Detection of such infection by real-time PCR using specific gene is lifesaving in children with impaired immunity. Therefore detection of CMV in urine and saliva in first two weeks of life may help to prevent other complications.
